# The tuatara genome reveals ancient features of amniote evolution

**DOI:** 10.1038/s41586-020-2561-9

**Published:** 2020-08-05

**Authors:** Neil J. Gemmell, Kim Rutherford, Stefan Prost, Marc Tollis, David Winter, J. Robert Macey, David L. Adelson, Alexander Suh, Terry Bertozzi, José H. Grau, Chris Organ, Paul P. Gardner, Matthieu Muffato, Mateus Patricio, Konstantinos Billis, Fergal J. Martin, Paul Flicek, Bent Petersen, Lin Kang, Pawel Michalak, Thomas R. Buckley, Melissa Wilson, Yuanyuan Cheng, Hilary Miller, Ryan K. Schott, Melissa D. Jordan, Richard D. Newcomb, José Ignacio Arroyo, Nicole Valenzuela, Tim A. Hore, Jaime Renart, Valentina Peona, Claire R. Peart, Vera M. Warmuth, Lu Zeng, R. Daniel Kortschak, Joy M. Raison, Valeria Velásquez Zapata, Zhiqiang Wu, Didac Santesmasses, Marco Mariotti, Roderic Guigó, Shawn M. Rupp, Victoria G. Twort, Nicolas Dussex, Helen Taylor, Hideaki Abe, Donna M. Bond, James M. Paterson, Daniel G. Mulcahy, Vanessa L. Gonzalez, Charles G. Barbieri, Dustin P. DeMeo, Stephan Pabinger, Tracey Van Stijn, Shannon Clarke, Oliver Ryder, Scott V. Edwards, Steven L. Salzberg, Lindsay Anderson, Nicola Nelson, Clive Stone, Ngatiwai Trust Board, Clive Stone, Jim Smillie, Haydn Edmonds

**Affiliations:** 1 Department of Anatomy, University of Otago, Dunedin, New Zealand; 2 LOEWE-Center for Translational Biodiversity Genomics, Senckenberg Museum, Frankfurt, Germany; 3 South African National Biodiversity Institute, National Zoological Garden, Pretoria, South Africa; 4 School of Life Sciences, Arizona State University, Tempe, AZ USA; 5 School of Informatics, Computing, and Cyber Systems, Northern Arizona University, Flagstaff, AZ USA; 6 School of Fundamental Sciences, Massey University, Palmerston North, New Zealand; 7 Peralta Genomics Institute, Oakland, CA USA; 8 School of Biological Sciences, The University of Adelaide, Adelaide, South Australia Australia; 9 Department of Ecology and Genetics – Evolutionary Biology, Evolutionary Biology Centre (EBC), Uppsala University, Uppsala, Sweden; 10 Department of Organismal Biology – Systematic Biology, Evolutionary Biology Centre (EBC), Uppsala University, Uppsala, Sweden; 11 Evolutionary Biology Unit, South Australian Museum, Adelaide, South Australia Australia; 12 Amedes Genetics, Amedes Medizinische Dienstleistungen, Berlin, Germany; 13 Museum für Naturkunde Berlin, Leibniz-Institut für Evolutions- und Biodiversitätsforschung an der Humboldt-Universität zu Berlin, Berlin, Germany; 14 Department of Earth Sciences, Montana State University, Bozeman, MT USA; 15 Department of Biochemistry, University of Otago, Dunedin, New Zealand; 16 European Molecular Biology Laboratory, European Bioinformatics Institute, Hinxton, UK; 17 Section for Evolutionary Genomics, The GLOBE Institute, Faculty of Health and Medical Sciences, University of Copenhagen, Copenhagen, Denmark; 18 Edward Via College of Osteopathic Medicine, Blacksburg, VA USA; 19 Center for One Health Research, Virginia–Maryland College of Veterinary Medicine, Blacksburg, VA USA; 20 Institute of Evolution, University of Haifa, Haifa, Israel; 21 Manaaki Whenua - Landcare Research, Auckland, New Zealand; 22 School of Biological Sciences, The University of Auckland, Auckland, New Zealand; 23 School of Life and Environmental Sciences, The University of Sydney, Sydney, New South Wales Australia; 24 Biomatters, Auckland, New Zealand; 25 Department of Vertebrate Zoology, National Museum of Natural History, Smithsonian Institution, Washington, DC USA; 26 The New Zealand Institute for Plant and Food Research, Auckland, New Zealand; 27 Departamento de Ecología, Facultad de Ciencias Biológicas, Pontificia Universidad Católica de Chile, Santiago, Chile; 28 Department of Ecology, Evolution, and Organismal Biology, Iowa State University, Ames, IA USA; 29 Instituto de Investigaciones Biomédicas ‘Alberto Sols’ CSIC-UAM, Madrid, Spain; 30 Division of Evolutionary Biology, Faculty of Biology, Ludwig-Maximilian University of Munich, Planegg-Martinsried, Germany; 31 Centre for Genomic Regulation (CRG), The Barcelona Institute for Science and Technology, Universitat Pompeu Fabra (UPF), Barcelona, Spain; 32 School of Biological Sciences, University of Canterbury, Christchurch, New Zealand; 33 Global Genome Initiative, National Museum of Natural History, Smithsonian Institution, Washington, DC USA; 34 Austrian Institute of Technology (AIT), Center for Health and Bioresources, Molecular Diagnostics, Vienna, Austria; 35 AgResearch, Invermay Agricultural Centre, Mosgiel, New Zealand; 36 San Diego Zoo Institute for Conservation Research, Escondido, CA USA; 37 Department of Organismic and Evolutionary Biology and the Museum of Comparative Zoology, Harvard University, Cambridge, MA USA; 38 Department of Biomedical Engineering, Johns Hopkins University, Baltimore, MD USA; 39 School of Biological Sciences, Victoria University of Wellington, Wellington, New Zealand; 40 Ngatiwai Trust Board, Whangarei, New Zealand

**Keywords:** Conservation biology, Phylogenetics, Comparative genomics, Genome evolution

## Abstract

The tuatara (*Sphenodon punctatus*)—the only living member of the reptilian order Rhynchocephalia (Sphenodontia), once widespread across Gondwana^1,2^—is an iconic species that is endemic to New Zealand^2,3^. A key link to the now-extinct stem reptiles (from which dinosaurs, modern reptiles, birds and mammals evolved), the tuatara provides key insights into the ancestral amniotes^2,4^. Here we analyse the genome of the tuatara, which—at approximately 5 Gb—is among the largest of the vertebrate genomes yet assembled. Our analyses of this genome, along with comparisons with other vertebrate genomes, reinforce the uniqueness of the tuatara. Phylogenetic analyses indicate that the tuatara lineage diverged from that of snakes and lizards around 250 million years ago. This lineage also shows moderate rates of molecular evolution, with instances of punctuated evolution. Our genome sequence analysis identifies expansions of proteins, non-protein-coding RNA families and repeat elements, the latter of which show an amalgam of reptilian and mammalian features. The sequencing of the tuatara genome provides a valuable resource for deep comparative analyses of tetrapods, as well as for tuatara biology and conservation. Our study also provides important insights into both the technical challenges and the cultural obligations that are associated with genome sequencing.

## Abstract (web summary)

The approximately 5-Gb tuatara (*Sphenodon punctatus*) genome assembly provides a resource for analysing amniote evolution, and highlights the imperative for meaningful cultural engagement with Indigenous communities in genome-sequencing endeavours.

## Main

**Fig. 1:**
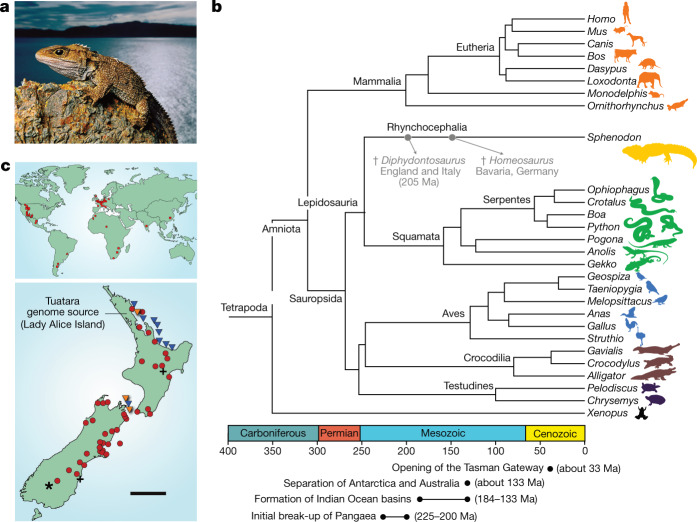
The phylogenetic significance and distribution of the tuatara. **a**, The tuatara, (*S. punctatus*) is the sole survivor of the order Rhynchocephalia. **b**, **c**, The rhynchocephalians appear to have originated in the early Mesozoic period (about 250–240 million years ago (Ma)) and were common, speciose and globally distributed for much of that era. The geographical range of the rhynchocephalians progressively contracted after the Early Jurassic epoch (about 200–175 Ma); the most recent fossil record outside of New Zealand is from Argentina in the Late Cretaceous epoch (about 70 Ma). **c**, The last bastions of the rhynchocephalians are 32 islands off the coast of New Zealand, which have recently been augmented by the establishment of about 10 new island or mainland sanctuary populations using translocations. The current global population is estimated to be around 100,000 individuals. Rhynchocephalian and tuatara fossil localities are redrawn and adapted from ref. ^1^ with permission, and incorporate data from ref. ^2^. In the global distribution map (**c**, top); triangle = Triassic; square = Jurassic; circle = Cretaceous; and diamond = Palaeocene. In the map of the New Zealand distribution (**c**, bottom); asterisk = Miocene; cross = Pleistocene; circle = Holocene; blue triangle = extant population; and orange triangle = population investigated in this study. Scale bar, 200 km. Photograph credit, F. Lanting.

The tuatara is an iconic terrestrial vertebrate that is unique to New Zealand^2^. The tuatara is the only living member of the archaic reptilian order Rhynchocephalia (Sphenodontia), which last shared a common ancestor with other reptiles at about 250 million years ago (Fig. 1); this species represents an important link to the now-extinct stem reptiles from which dinosaurs, modern reptiles, birds and mammals evolved, and is thus important for our understanding of amniote evolution^2^.

It is also a species of importance in other contexts. First, the tuatara is a *taonga* (special treasure) for Māori, who hold that tuatara are the guardians of special places^2^. Second, the tuatara is internationally recognized as a critically important species that is vulnerable to extinction owing to habitat loss, predation, disease, global warming and other factors^2^. Third, the tuatara displays a variety of morphological and physiological innovations that have puzzled scientists since its first description^2^. These include a unique combination of features that are shared variously with lizards, turtles and birds, which left its taxonomic position in doubt for many decades^2^. This taxonomic conundrum has largely been addressed using molecular approaches^4^, but the timing of the split of the tuatara from the lineage that forms the modern squamates (lizards and snakes), the rate of evolution of tuatara and the number of species of tuatara remain contentious^2^. Finally, there are aspects of tuatara biology that are unique within, or atypical of, reptiles. These include a unique form of temperature-dependent sex determination (which sees females produced below, and males above, 22 °C), extremely low basal metabolic rates and considerable longevity^2^.

To provide insights into the biology of the tuatara, we have sequenced its genome in partnership with Ngātiwai, the Māori *iwi* (tribe) who hold *kaitiakitanga* (guardianship) over the tuatara populations located on islands in the far north of New Zealand. This partnership—which, to our knowledge, is unique among the genome projects undertaken to date—had a strong practical focus on developing resources and information that will improve our understanding of the tuatara and aid in future conservation efforts. It is hoped that this work will form an exemplar for future genome initiatives that aspire to meet access and benefit-sharing obligations to Indigenous communities.

We find that the tuatara genome—as well as the animal itself—is an amalgam of ancestral and derived characteristics. Tuatara has 2*n* = 36 chromosomes in both sexes, consisting of 14 pairs of macrochromosomes and 4 pairs of microchromosomes^5^. The genome size, which is estimated to be approximately 5 Gb, is among the largest of the vertebrate genomes sequenced to date; this is predominantly explained by an extraordinary diversity of repeat elements, many of which are unique to the tuatara.

## Sequencing, assembly, synteny and annotation

**Extended Data Fig. 1:**
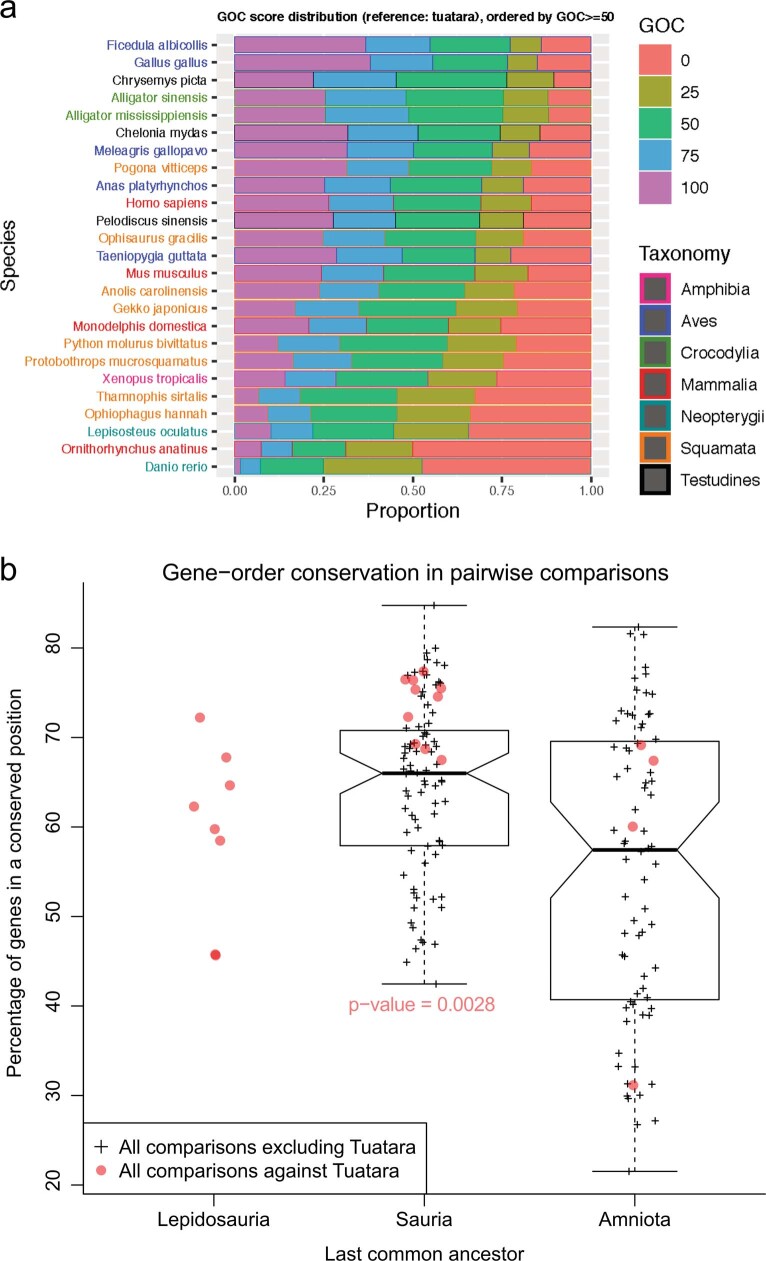
Gene order conservation. **a**, Gene-order conservation score distribution using the tuatara as a reference. Species are ordered by the proportion of top-scoring orthologues ≥ 50. **b**, Gene-order conservation versus divergence time. For the three taxonomic groupings (Lepidosauria, Sauria and Amniota), we analysed the percentage of genes that are found in a conserved position across all pairs of genomes. Pairwise comparisons involving tuatara are shown in plain red circles (respectively, *n* = 8, *n* = 10, and *n* = 4), and comparisons that do not involve tuatara are black (box plot and + signs; respectively, *n* = 0, *n* = 80 and *n* = 72). The conservation of gene order between tuatara, birds and turtles is significantly higher (one-sided, two-sample Kolmogorov–Smirnov test, *P* value = 2.8 × 10^−3^, *D* = 0.575) than that observed between squamates, birds and turtles. As the tuatara is the only remaining rhynchocephalian, there is no control distribution for the Lepidosauria ancestor. Box plot coordinates (minimum, first quartile, median, mean, third quartile, maximum) are 42.46%, 57.93%, 66.00%, 64.50%, 70.66% and 84.76% for the Sauria box plot, and 21.52%, 40.81%, 57.43%, 55.17%, 69.46% and 82.34% for the Amniota box plot.

**Extended Data Fig. 2:**
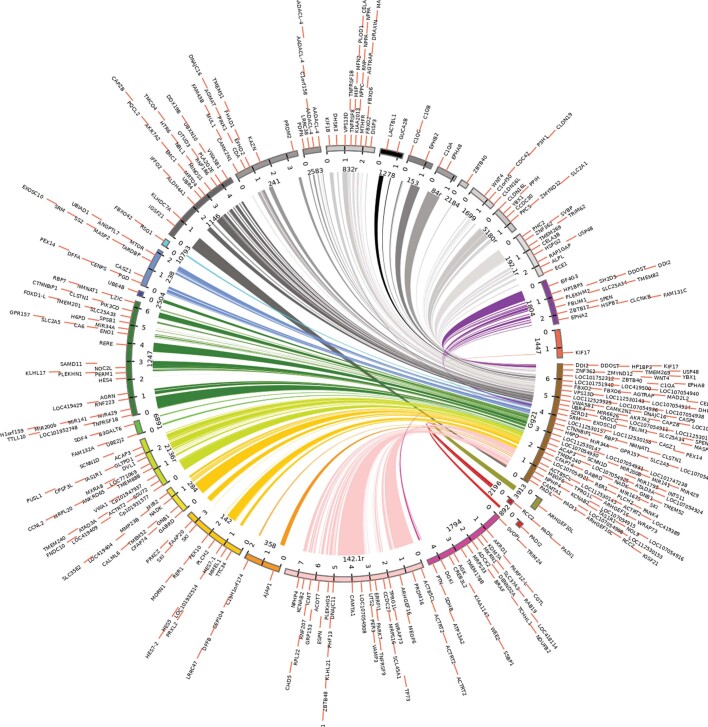
Co-linearity between *Gallus gallus* chromosome 21 and tuatara contigs. Circos plot highlighting the gene-order conservation observed between chicken chromosome 21 (assembly GRCg6a) and multiple tuatara contigs. The gene names shown derive from the chicken assembly.

Our tuatara genome assembly is 4.3 Gb, consisting of 16,536 scaffolds with an N50 scaffold length of 3 Mb (Extended Data Table 1, Supplementary Information 1). Genome assessment using Benchmarking Universal Single-Copy Orthologs (BUSCO)^6^ indicates 86.8% of the vertebrate gene set are present and complete. Subsequent annotation identified 17,448 genes, of which 16,185 are one-to-one orthologues (Supplementary Information 2). Local gene-order conservation is high; 75% or more of tuatara genes showed conservation with birds, turtles and crocodilians. We also find that components of the genome, of 15 Mb in size and larger, are syntenic with other vertebrates; protein-coding gene order and orientation are maintained between tuatara, turtle, chicken and human, and strong co-linearity is seen between tuatara contigs and chicken chromosomes (Extended Data Figs. 1, 2).

## Genomic architecture

**Fig. 2:**
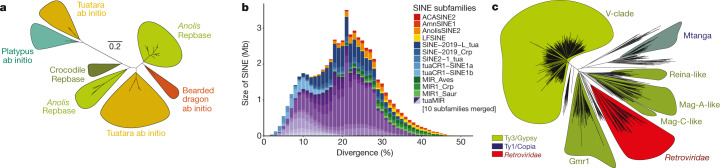
Analysis of the repeat landscape in the tuatara genome identifies unique repeat families, evidence of recent activity and a greater expansion and diversity of repeats than any other amniote. **a**, A phylogenetic analysis on the basis of the reverse transcriptase domain of L2 repeats identifies two L2 subfamilies; one typical of other lepidosaurs and one that is similar to platypus L2. This phylogeny is based on L2 elements >1.5-kb long with a reverse transcriptase domain of >200 amino acids. **b**, Landscape plot of SINE retrotransposons suggests the tuatara genome is dominated by MIR sequences that are most typically associated with mammals; the tuatara genome is now the amniote genome in which the greatest MIR diversity has been observed. Only SINE subfamilies that occupy more than 1,000 bp are shown. Definitions of the abbreviations of the SINE subfamilies follow: ACASINE2, *Anolis carolinesis* SINE family; AmnSINE1, Amniota SINE1; AnolisSINE2, *A. carolinesis* SINE2 family, LFSINE, lobe-finned fishes SINE; SINE−2019−L_tua, tuatara SINE; SINE-2019_Crp, *Crocodylus porosus* SINE; SINE2-1_tua, tuatara SINE2; tuaCR1-SINE1a and b, tuatara CR1-mobilized SINEs; MIR_Aves, avian MIR sequence; MIR1_Crp, *C. porosus* MIR sequence; MIR1_Saur, Sauropsida MIR sequence; tuaMIR, tuatara MIRs. **c**, The tuatara genome contains about 7,500 full-length, long-terminal-repeat retro-elements, including nearly 450 endogenous retroviruses that span the five major retroviral clades. A Ty1/Copia element (Mtanga-like) is especially abundant, but Bel-Pao long-terminal-repeat retro-elements are absent. At least 37 complete spumaretroviruses are present in the tuatara genome.

At least 64% of the tuatara genome assembly is composed of repetitive sequences, made up of transposable elements (31%) and low-copy-number segmental duplications (33%). Although the total transposable element content is similar to other reptiles^7^, the types of repeats we found appear to be more mammal-like than reptile-like. Furthermore, a number of the repeat families show evidence of recent activity and greater expansion and diversity than seen in other vertebrates (Fig. 2).

L2 elements account for most of the long interspersed elements in the tuatara genome (10% of the genome), and some may still be active (Supplementary Information 4). CR1 elements—the dominant long interspersed element in the genomes of other sauropsids^8^—are rare. CR1 elements comprise only about 4% of the tuatara genome (Fig. 2a, Supplementary Table 4.1), but some are potentially active (Supplementary Fig. 4.4). L1 elements, which are prevalent in placental mammals, account for only a tiny fraction of the tuatara genome (<1%) (Supplementary Table 4.1). However, we find that an L2 subfamily that is present in the tuatara, but is absent from other lepidosaurs, is also common in monotremes^9^ (Supplementary Figs. 4.3–4.5). Collectively, these data suggest that stem-sauropsid ancestors had a repeat composition that was very different from that inferred in previous comparisons using mammals, birds and lizards^7^.

Many of the short interspersed elements (SINEs) in the tuatara are derived from ancient common sequence motifs (CORE-SINEs), which are present in all amniotes^10^; however, at least 16 SINE subfamilies were recently active in the tuatara genome (Fig. 2b, Supplementary Information 5). Most of these SINEs are mammalian-wide interspersed repeats (MIRs), and the diversity of MIR subfamilies in the tuatara is the highest thus far observed in an amniote^11,12^. In the human genome, hundreds of fossil MIR elements act as chromatin and regulatory domains^13^; the very recent activity of diverse MIR subfamilies in the tuatara suggests these subfamilies may have influenced regulatory rewiring on rather recent evolutionary timescales.

We detected 24 newly identified and unique families of DNA transposon, which suggests frequent germline infiltration by DNA transposons through horizontal transfer in the tuatara^14^. At least 30 subfamilies of DNA transposon were recently active, spanning a diverse range of cut-and-paste transposons and polintons (Supplementary Figs. 5.1, 5.2). This diversity is higher than that found in other amniotes^15^. Notably, we found thousands of identical DNA transposon copies, which suggests very recent—and/or ongoing—activity. Cut-and-paste transposition probably shapes the tuatara genome, as it does in bats^15^.

We identified about 7,500 full-length, long-terminal-repeat retro-elements (including endogenous retroviruses), which we classified into 12 groups (Fig. 2c, Supplementary Information 6). The general spectrum of long-terminal-repeat retroelements in the tuatara is comparable to that of other sauropsids^7,15^. We found at least 37 complete spumaretroviruses, which are among the most ancient of endogenous retroviruses^16^, in the tuatara genome (Fig. 2c, Supplementary Figs. 6.1, 6.2).

The tuatara genome contains more than 8,000 elements related to non-coding RNA. Most of these elements (about 6,900) derive from recently active transposable elements, and overlap with a newly identified CR1-mobilized SINE (Fig. 2b, Supplementary Information 7). The remaining high-copy-number elements are sequences closely related to ribosomal RNAs, spliceosomal RNAs and signal-recognition particle RNAs.

Finally, a high proportion (33%) of the tuatara genome originates from low-copy-number segmental duplications; 6.7% of these duplications are of recent origin (on the basis of their high level of sequence identity (>94% identity)), which is more than seen in other vertebrates^9^. The tuatara genome is 2.4× larger than the anole genome, and this difference appears to be driven disproportionately by segmental duplications.

Overall, the repeat architecture of the tuatara is—to our knowledge—unlike anything previously reported, showing a unique amalgam of features that have previously been viewed as characteristic of either reptilian or mammalian lineages. This combination of ancient amniote features—as well as a dynamic and diverse repertoire of lineage-specific transposable elements—strongly reflects the phylogenetic position of this evolutionary relic.

**Fig. 3:**
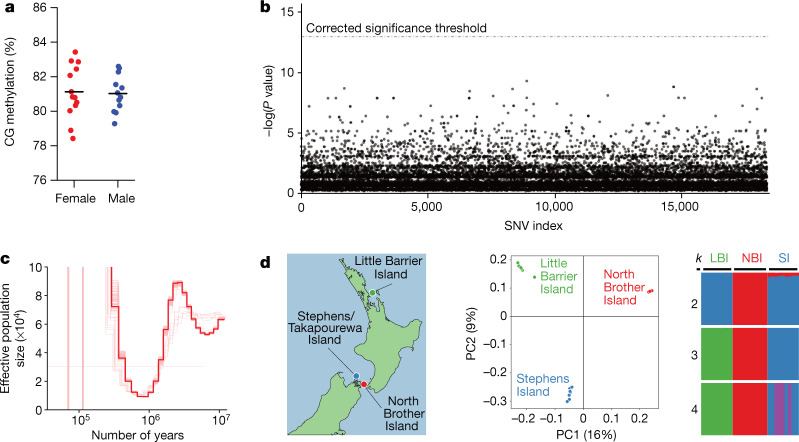
Analysis of sex differences, demographic history and population structure. **a**, Methylation levels in the tuatara genome are high (mean 81%), but show no significant differences among the sexes (female *n* = 13, mean = 81.13, s.d. = 1.55; male *n* = 12; mean = 81.02, s.d.−1.07). The black horizontal line represents the mean in each dataset. **b**, No single-nucleotide variant (SNV) is significantly differentiated with respect to sex in the tuatara genome. Each point represents a *P* value from a test of sexual differentiation for a single SNV. The dashed line represents the threshold for statistical significance after accounting for multiple testing (*n* = 28; 13 males and 15 females). *P* values calculated using Fisher’s exact test, two-tailed test and corrected for multiple testing using the Bonferroni method. **c**, Pairwise sequential Markovian coalescent plot of the demographic history of tuatara using a mutation rate of 1.4 × 10^−8^ substitutions per site per generation and a generation time of 30 years. **d**, We examined the three known axes of genetic diversity in tuatara: northern New Zealand (Little Barrier Island (LBI) (*n* = 9)) and two islands in the Cook Strait (Stephens Island (SI) (*n* = 9) and North Brother Island (NBI) (*n* = 10)), using genotype-by-sequencing methods. Principal component (PC) analysis and structure plots demonstrate substantial structure among tuatara populations, and strongly support previous suggestions that the tuatara on the North Brother Island are genetically distinct and warrant separate management.

Our low-coverage bisulfite-sequencing analysis found approximately 81% of CpG sites are methylated in tuatara (Fig. 3a)—the highest reported percentage of methylation for an amniote. This pattern differs from that observed in mouse, human (about 70%) and chicken (about 50%), and is more similar to that of *Xenopus* (82%) and zebrafish (78%). One possible explanation for this high level of DNA methylation is the large number of repetitive elements found in tuatara, many of which appear recently active and might be regulated via DNA methylation.

**Extended Data Fig. 3:**
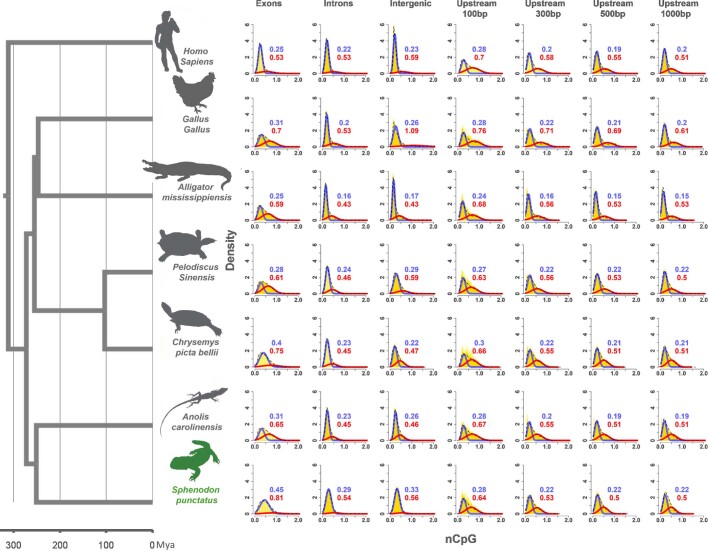
Normalized CpG distributions for tuatara and other vertebrates. Tuatara show a statistically significant bimodal normalized CpG distribution in all genomic regions examined (gene promoters, exons, introns and intergenic sequences). Explicit testing using mixed models uncovered global normalized CpG bimodality across all taxa, indicating that bimodality is ancestral and highly conserved across vertebrates irrespective of sex-determining mechanism. Further details are provided in Supplementary Information 8. Silhouettes are hand-drawn from the original photographs of A. Eckley, except for *Anolis* (D. Hobern, CC-BY-2.0), crocodile and hen (designed by Creazilla, https://creazilla.com/).

The low normalized CpG content of the tuatara suggests its genome has endured substantial historic methylation^17^. The tuatara has a significantly bimodal distribution of normalized CpG (Extended Data Fig. 3) in all of the genomic regions we examined, a similarity it shares with other reptiles that have temperature-dependent sex determination^17^. The low normalized CpG count of the tuatara in non-promoter regions may result from methylation silencing of repeat elements, and the bimodality of normalized CpG promoters suggests dual transcriptional regulation (Extended Data Fig. 3, Supplementary Information 8).

**Extended Data Fig. 4:**
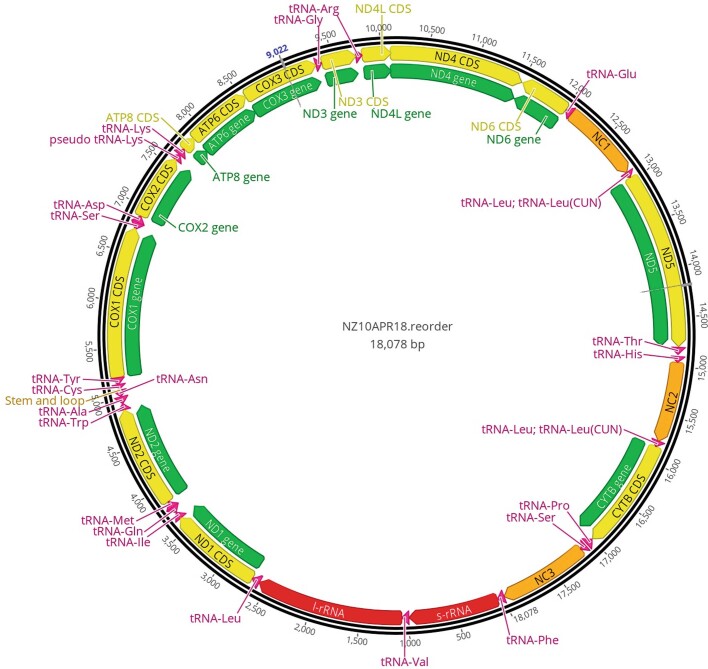
The mitochondrial genome of the tuatara. In the Lady Alice Island reference tuatara, this molecule is 18,078 bp, containing 13 protein-coding, 2 rRNA and 22 tRNA genes, standard among animals and contradicting previous reports that 3 genes (*ND5*, t*RNA*^*Thr*^ and *tRNA*^*His*^) were absent. Three non-coding areas with control region (heavy-strand replication origin) features (NC1, NC2 and NC3), and two copies of *tRNA*^*Leu(CUN)*^ adjacent to NC1 and NC2 possess identical or near-identical sequence, possibly as a result of concerted evolution. A stem-and-loop structure is observed in the region encoding tRNA^Asn^ and tRNA^Cys^, which may supplement for the origin of light-strand replication (O_L_) normally found in this location. The *tRNA*^*Lys*^ gene is duplicated with the first copy, possibly a pseudo-gene. The *tRNA*^*Cys*^ gene encodes a tRNA with a D-arm replacement loop. The gene and structure order is: *tRNA*^*Phe*^, *12S* rRNA, *tRNA*^*Val*^, *16S* rRNA, *tRNA*^*Leu(UUR)*^, ND1, *tRNA*^*Ile*^, *tRNA*^*Gln*^, *tRNA*^*Met*^, ND2, *tRNA*^*Trp*^, *tRNA*^*Ala*^*, tRNA*^*Asn*^, O_L_-like structure, *tRNA*^*Cys*^, *tRNA*^*Tyr*^, *COI*, *tRNA*^*Ser(UCN)*^, *tRNA*^*Asp*^, *COII*, pseudo-*tRNA*^*Lys*^, *tRNA*^*Lys*^, *ATP8*, *ATP6*, *COIII*, *tRNA*^*Gly*^, ND3, *tRNA*^*Arg*^, *ND4L*, *ND4*, *ND6*, *tRNA*^*Glu*^, NC1, *tRNA*^*Leu(CUN)*^ copy one, *ND5*, *tRNA*^*Thr*^, *tRNA*^*His*^, NC2, *tRNA*^*Leu(CUN)*^ copy two, *CYTB*, *tRNA*^*Pro*^, *tRNA*^*Ser(AGY)*^ and NC3.

The mitochondrial genome in the tuatara reference animal is 18,078 bp in size, containing 13 protein-coding, 2 ribosomal RNA and 22 transfer (t)RNA genes, a gene content typical among animals (Extended Data Fig. 4). This contradicts previous reports^18^ that the tuatara mitochondrial genome lacks three genes: *ND5*, *tRNA*^*Thr*^ and *tRNA*^*His*^. These genes are found—with an additional copy of *tRNA*^*Leu(CUN)*^ and an additional non-coding block (which we refer to as NC2)—in a single segment of the mitochondrial genome. Three non-coding areas (NC1, NC2 and NC3) with control-region (heavy-strand replication origin) features, and two copies of *tRNA*^*Leu(CUN)*^ adjacent to NC1 and NC2, possess identical or near-identical sequences that are unique to the tuatara mitochondrial genome. These three non-coding regions may be a result of concerted evolution.

## Genomic innovations

As befits the taxonomic distinctiveness of the tuatara, we find that its genome displays multiple innovations in genes that are associated with immunity, odour reception, thermal regulation and selenium metabolism.

**Extended Data Fig. 5:**
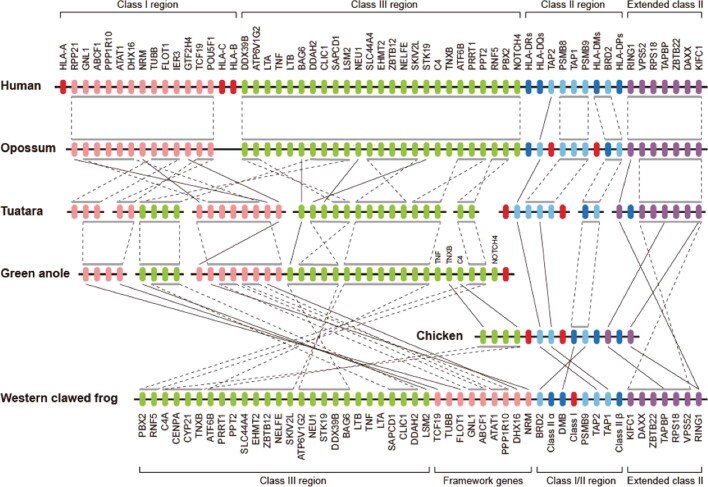
Comparative analysis of the MHC core region. Only genes that were annotated in the tuatara genome were included in the analysis. Orthologues between species are connected by a solid line; the grey bars above and below genes indicate syntenic blocks and are linked by dashed lines between species. *Anolis* MHC class I/II and extended class II regions are not shown, owing to the high degree of genome assembly fragmentation in these regions. Red, class I genes; pink, class I region framework genes; green, class III genes; dark blue, class II genes; light blue, class II region framework genes; purple, extended class II genes.

Genes of the major histocompatibility complex (MHC) have an important role in disease resistance, mate choice and kin recognition, and are among the most polymorphic genes in the vertebrate genome. Our annotation of MHC regions in the tuatara, and comparisons of the gene organization with that of six other species, identified 56 MHC genes (Extended Data Fig. 5, Supplementary Information 9).

Of the six comparison species, the genomic organization of tuatara MHC genes is most similar to that of the green anole, which we interpret as typical for Lepidosauria. Tuatara and other reptiles show a gene content and complexity more similar to the MHC regions of amphibians and mammals than to the highly reduced MHC of birds. Although the majority of genes annotated in the tuatara MHC are well-conserved as one-to-one orthologues, we observed extensive genomic rearrangements among these distant lineages.

The tuatara is a highly visual predator that is able to capture prey under conditions of extremely low light^2^. Despite the nocturnal visual adaptation of the tuatara, it shows strong morphological evidence of an ancestrally diurnal visual system^19^. We identified all five of the vertebrate visual opsin genes in the tuatara genome (Supplementary Information 10).

**Extended Data Fig. 6:**
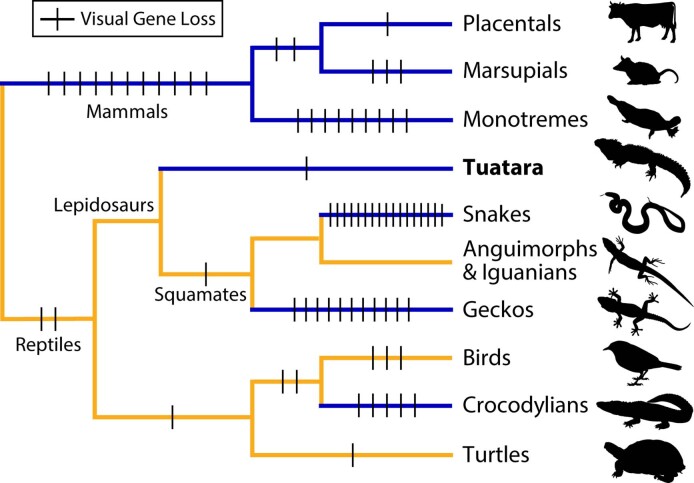
Phylogenetic tree of amniotes depicting inferred visual gene losses. Lineages are coloured based on a rough approximation of their ancestral activity pattern (blue, nocturnal; yellow, diurnal). The tuatara lineage has experienced some of the lowest rates of gene loss despite a nocturnal ancestry, which in other lineages is associated with increased gene loss. Supplementary Table 10.1 provides a summary of genes lost per branch. The complete table of visual genes analysed and their presence or absence from the various groups is available at http://www.doi.org/10.5281/zenodo.2597599. Silhouettes were designed by Creazilla, www.creazilla.com.

Our comparative analysis revealed one of the lowest rates of visual-gene loss known for any amniote, which contrasts sharply with the high rates of gene loss observed in ancestrally nocturnal lineages (Extended Data Fig. 6). Visual genes involved in phototransduction showed strong negative selection and no evidence for the long-term shifts in selective pressures that have been observed in other groups with evolutionarily modified photoreceptors^20^. The retention of five visual opsins and the conserved nature of the visual system also suggests tuatara possess robust colour vision, potentially at low light levels. This broad visual repertoire may be explained by the dichotomy in tuatara life history: juvenile tuatara often take up a diurnal and arboreal lifestyle to avoid the terrestrial, nocturnal adults that may predate them^2^. Collectively, these results suggest a unique path to nocturnal adaptation in tuatara from a diurnal ancestor.

Odorant receptors are expressed in the dendritic membranes of olfactory receptor neurons and enable the detection of odours. Species that depend strongly on their sense of smell to interact with their environment, find prey, identify kin and avoid predators may be expected to have a large number of odorant receptors. The tuatara genome contains 472 predicted odorant receptors, of which 341 sequences appear intact (Supplementary Information 11). The remainder lack the initial start codon, have frameshifts or are presumed to be pseudogenes. Many odorant receptors were found as tandem arrays, with up to 26 genes found on a single scaffold.

**Extended Data Fig. 7:**
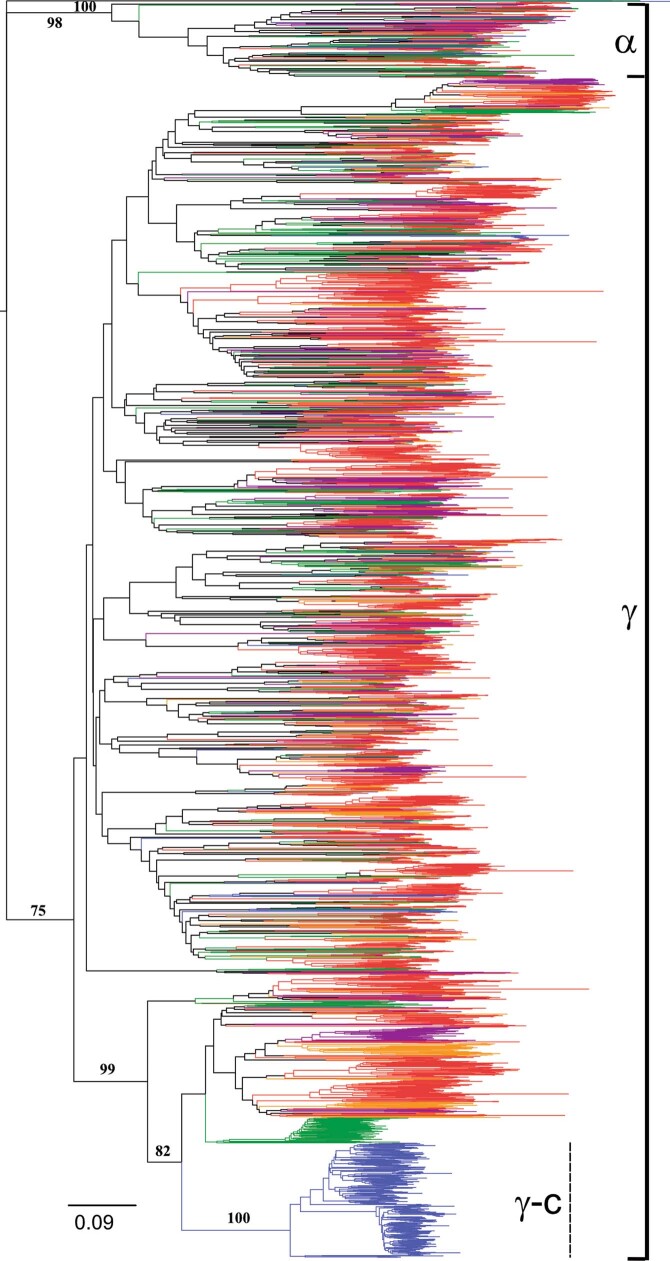
The evolutionary history of odorant receptors in terrestrial sauropsids. The relationship among odorant receptors was inferred using the neighbour-joining method. The unrooted tree contains 3,213 amino acid sequences. Branches are coloured according to the following categories: green, tuatara; blue, birds (*G. gallus* and *Taeniopygia guttata*); red, snakes (*Notechis scutatus*, *Ophiophagus hannah, Protobothrops mucrosquamatus*, *Pseudonaja textilis*, *P. bivittatus* and *Thamnophis sirtalis*); orange, lizards (*A. carolinensis* and *Pogona vitticeps*); and purple, gecko (*Gekko japonicas*). Bootstrap support values above 75% (1,000 replicates) are indicated for major branch splits relating to the different odorant receptor groups and branches leading to the species-specific odorant receptor expansions in birds (group γ–c) and tuatara (*).

The number and diversity of odorant receptor genes varies greatly in Sauropsida: birds have 182–688 such genes, the green anole lizard has 156 genes, and crocodilians and testudines have 1,000–2,000 genes^21^. The tuatara has a number of odorant receptors similar to that of birds, but contains a high percentage of intact odorant receptor genes (85%) relative to published odorant receptor sets from the genomes of other sauropsids. This may reflect a strong reliance on olfaction by tuatara, and therefore pressure to maintain a substantial repertoire of odorant receptors (Extended Data Fig. 7). There is some evidence that olfaction has a role in identifying prey^2^, as well as suggestions that cloacal secretions may act as chemical signals.

**Extended Data Fig. 8:**
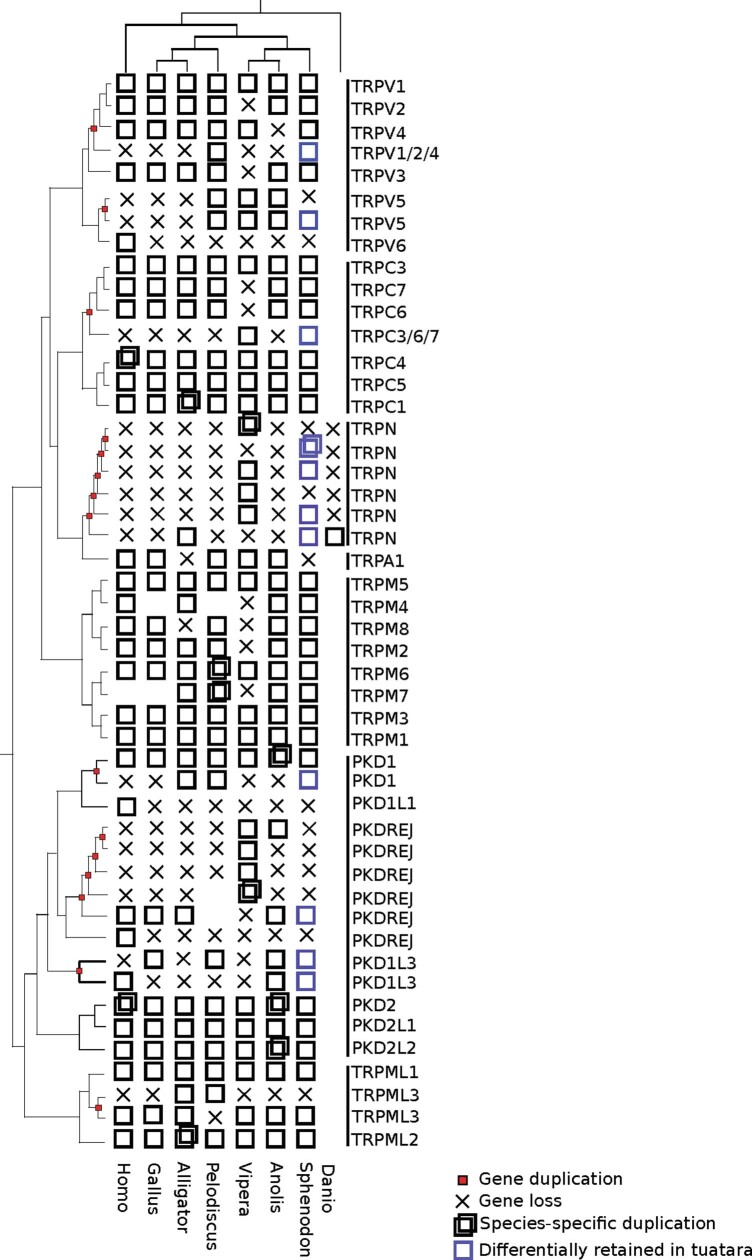
The repertoire of the TRP genes identified in tuatara. We compared the TRP genes identified in tuatara (*S. punctatus)* to those found in other six vertebrate species: lizard (*A. carolinensis*), viper (*Vipera berus*), turtle (*Pelodiscus sinensis*), alligator (*Alligator mississippiensis*), chicken (*G. gallus*), and human (*Homo sapiens*). Small red squares on nodes indicate gene duplications, duplicated boxes indicate species-specific duplications and blue boxes indicate differential gene retentions in tuatara. Crosses indicate genes that were lost after duplication and empty spaces genes that have most probably been lost, but are not yet confirmed.

The tuatara is a behavioural thermoregulator, and is notable for having the lowest optimal body temperature of any reptile (16–21 °C). Genes that encode transient receptor potential ion channels (TRP genes) have an important role in thermoregulation, as these channels participate in thermosensation and cardiovascular physiology^22^; this led us to hypothesize that TRP genes may be linked to the thermal tolerance of the tuatara. Our comparative genomic analysis of TRP genes in the tuatara genome identified 37 TRP-like sequences, spanning all 7 known subfamilies of TRP genes (Extended Data Fig. 8, Supplementary Information 12)— an unusually large repertoire of TRP genes.

Among this suite of genes, we identified thermosensitive and non-thermosensitive TRP genes that appear to result from gene duplication, and have been differentially retained in the tuatara. For example, the tuatara is unusual in possessing an additional copy of a thermosensitive TRPV-like gene (*TRPV1/2/4*, sister to the genes *TRPV1*, *TRPV2* and *TRPV4*) that has classically been linked to the detection of moderate-to-extreme heat^22^—a feature it shares with turtles. A strong signature of positive selection among heat-sensitive TRP genes (*TRPA1*, *TRPM* and *TRPV*) was also observed.

In general, these results show a high rate of differential retention and positive selection in genes for which a function in heat sensation is well-established^22^. It therefore seems probable that the genomic changes in TRP genes are associated with the evolution of thermoregulation in tuatara.

Barring tortoises, tuatara are the longest lived of the reptiles—probably exceeding 100 years of age^2^. This enhanced lifespan may be linked to genes that afford protection against reactive oxygen species. One class of gene products that affords such protection is the selenoproteins. The human genome encodes 25 selenoproteins, the roles of which include antioxidation, redox regulation, thyroid hormone synthesis and calcium signal transduction, among others^23^.

We identified 26 genes that encode selenoproteins in the tuatara genome, as well as 4 selenocysteine-specific tRNA genes; all of these appear to be functional (Supplementary Information 13). Although further work is needed, the additional selenoprotein gene (relative to the human genome) and the selenocysteine-specific tRNA genes may be linked to the longevity of tuatara or might have arisen as a response to the low levels of selenium and other trace elements in the terrestrial systems of New Zealand.

Tuatara has a unique mode of temperature-dependent sex determination, in which higher temperatures during egg incubation result in males^2^. We found orthologues for many genes that are known to act antagonistically in masculinizing (for example, *SF1* and *SOX9*) and feminizing (for example, *RSPO1* and *WNT4*) gene networks to promote testicular or ovarian development, respectively^24^. We also found orthologues of several genes that have recently been implicated in temperature-dependent sex determination, including *CIRBP*^24^ (Supplementary Information 17, Supplementary Table 17.2). Tuatara possess no obviously differentiable sex chromosomes^5^, and we found no significant sex-specific differences in global CG methylation (Fig. 3a) and no sex-specific single-nucleotide variants between male and female tuatara (Fig. 3b). On a gene-by-gene basis, sex-specific differences in methylation and gene expression patterns probably exist, but this remains to be investigated.

## Phylogeny and evolutionary rates

Our phylogenomic analyses, which incorporated both whole-genome alignments and clusters of single-copy orthologues (Supplementary Information 14, 15) recapitulated many patterns that have been observed in the fossil record and corroborated during the genomic era (Fig. 1). After their appearance about 312 million years ago^25^, amniote vertebrates diversified into two groups: the synapsids (which include all mammals) and the sauropsids (which include all reptiles and birds). We obtained full phylogenomic support for a monophyletic Lepidosauria, marked by the divergence of the tuatara lineage from all squamates (lizards and snakes) during the early part of the Triassic period at about 250 million years ago, as estimated using a penalized likelihood method (Fig. 1, Supplementary Information 14–16).

**Extended Data Fig. 9:**
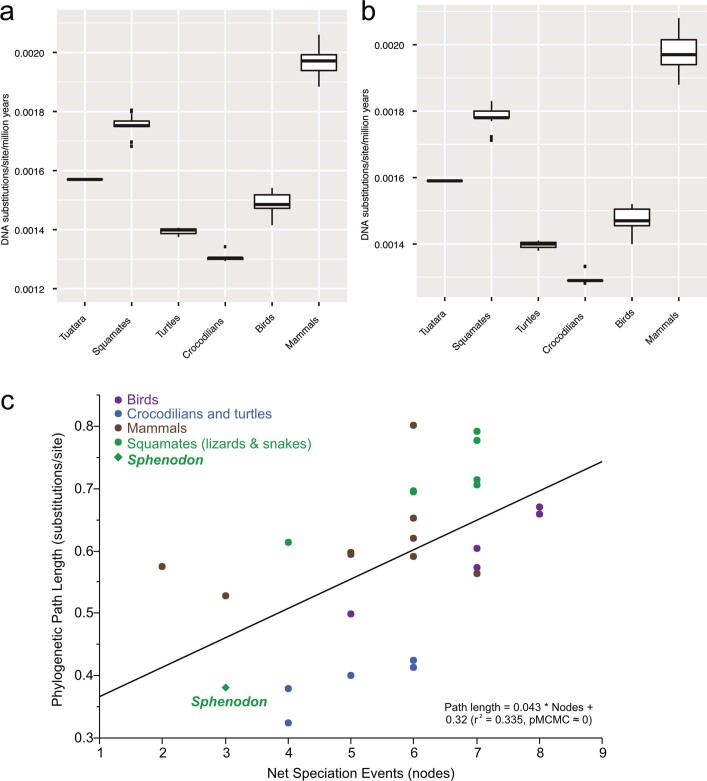
Estimated DNA substitution rates of amniote clades on the basis of fourfold-degenerate sites and a test for evidence of punctuated evolution. **a**, Box plots showing distribution of estimated substitution rates by clade using semiparametric penalized likelihood in r8s with fossil constraints from ref. ^36^ and ref. ^37^ for tuatara (*n* = 1, 0.00159), squamates (*n* = 13, minimum = 0.00171, maximum = 0.00183, median = 0.00178, 25th percentile = 0.00178, 75th percentile = 0.00183), turtles (*n* = 3, minimum = 0.00138, maximum = 0.00141, median = 0.00140, 25th percentile = 0.00139, 75th percentile = 0.00140), crocodilians (*n* = 5, minimum = 0.00128, maximum = 0.0133, median = 0.00129, 25th percentile = 0.00129, 75th percentile = 0.00129), birds (*n* = 11, minimum = 0.0014, maximum = 0.00152, median = 0.00147, 25th percentile = 0.00146, 75th percentile = 0.0015) and mammals (*n* = 15, minimum = 0.00188, maximum = 0.00208, median = 0.00197, 25th percentile = 0.00194, 75th percentile = 0.00201). **b**, Box plots showing distribution of estimated substitution rates with median time to most recent common ancestor estimates from www.timetree.org for tuatara (*n* = 1, 0.00157), squamates (*n* = 13, minimum = 0.00168, maximum = 0.00180, median = 0.00175, 25th percentile = 0.00178, 75th percentile = 0.00177), turtles (*n* = 3, minimum = 0.00138, maximum = 0.00141, median = 0.00140, 25th percentile = 0.00139, 75th percentile = 0.00140), crocodilians (*n* = 5, minimum = 0.00129, maximum = 0.0134, median = 0.00130, 25th percentile = 0.00130, 75th percentile = 0.00130), birds (*n* = 11, minimum = 0.00142, maximum = 0.00154, median = 0.00149, 25th percentile = 0.00147, 75th percentile = 0.00152) and mammals (*n* = 15, minimum = 0.00188, maximum = 0.00206, median = 0.00197, 25th percentile = 0.00194, 75th percentile = 0.00199). **c**, A test for evidence of punctuated evolution. The process of punctuated genome evolution predicts that the amount of evolution in the genome of a given species should correlate with the net number of speciation events. We used Bayesian phylogenetic generalized least squares to regress the total phylogenetic path length (of fourfold-degenerate sites) on the net number of speciation events (nodes in a phylogenetic tree). We find strong evidence for punctuated evolution, which accounts for 33.5% (*r*^2^; 95% credible interval = 0.34 to 0.38) of deviation from the molecular clock at fourfold-degenerate sites.

The rate of molecular evolution in the tuatara has previously been suggested to be paradoxically high, in contrast to the apparently slow rate of morphological evolution^26,27^. However, we find that the actual divergence in terms of DNA substitutions per site per million years at fourfold degenerate sites is relatively low, particularly with respect to lizards and snakes; this makes the tuatara the slowest-evolving lepidosaur yet analysed (Extended Data Fig. 9a, b). We also find that in general amniote evolution can be described by a model of punctuated evolution, in which the amount of genomic change is related to the degree of species diversification within clades^28,29^. The tuatara falls well below this trend, accumulating substitutions at a rate expected given the lack of rhynchocephalian diversity (Extended Data Fig. 9c, Supplementary Information 16). This suggests that rates of phenotypic and molecular evolution were not decoupled throughout the evolution of amniotes^30^.

## Patterns of selection

In two sets of analyses, we find that most genes exhibit a pattern of molecular evolution that suggests that the tuatara branch evolves at a different rate than the rest of the tree (Supplementary Information 17, Supplementary Table 4). Approximately 659 of the 4,284 orthologues we tested had significantly different *ω* values (ratios of non-synonymous to synonymous substitutions, dN/dS) on the tuatara branch relative to the birds and other reptiles we tested (Supplementary Information 17). Although none of these orthologues had *ω* values suggestive of strong positive selection (that is, >1), the results do indicate that shifts in patterns of selection are affecting many genes and functional categories of genes across the tuatara genome, including genes involved in RNA regulation, metabolic pathways, general metabolism and sex determination.

## Population genomics

Once widespread across the supercontinent of Gondwana, Rhynchocephalia is now represented by a single species (the tuatara) found on a few islands offshore of New Zealand (Fig. 1c). Historically, tuatara declined in range and numbers because of introduced pests and habitat loss^2^. They remain imperilled owing to their highly restricted distribution, threats imposed by disease and changes in sex ratios induced by climate change that could markedly affect their survival^31^. Previous work has found that populations in northern New Zealand are genetically distinct from those in the Cook Strait, and that the population on North Brother Island in the Cook Strait might be a distinct species^3^. Although subsequent studies have not supported species status for the population on North Brother Island^32^, it is managed as a separate conservation unit.

We used the tuatara reference genome to perform ancestral demographic and population genomic analyses of this species. First, we investigated genome-wide signals for demographic change using a pairwise sequentially Markovian coalescent method (Supplementary Information 18). Our reconstructed demography (Fig. 3c) reveals an increase in effective population size (*N*_e_) that is detectable around 10 million years ago, a marked decrease in *N*_e_ about 1–3 million years ago and a rapid increase in *N*_e_ between 500 thousand years ago and 1 million years ago. These events correlate well with the known geological history of New Zealand^33^, and may reflect an increase in available landmass subsequent to Oligocene drowning, a period of considerable climatic cooling that probably reduced tuatara habitat and the formation of land bridges that facilitated population expansion.

Our population genomic analyses examined the major axes of genetic diversity in tuatara^32,34^, and revealed substantial genetic structure (Fig. 3d, Supplementary Information 19). Our genome-wide estimate of the fixation index (*F*_ST_) is 0.45, and more than two-thirds of variable sites have an allele that is restricted to a single island. All populations have relatively low genetic diversity (nucleotide diversity ranges from 8 × 10^−4^ for North Brother Island to 1.1 × 10^−3^ for Little Barrier Island). The low within-population diversity and marked population structure we observe in the tuatara suggests that the modern island populations were isolated from each other sometime during the Last Glacial Maximum at about 18 thousand years ago.

Our results also support the distinctiveness of the North Brother Island tuatara, which has variously been described as *S. punctatus* or *Sphenodon guntheri*^3,32^. This population is highly inbred and shows evidence of a severe bottleneck, which most probably reflects a founder event around the time of the last glaciation^34^. It is not clear whether the distinctiveness we observe is due to changes in allele frequency brought about by this bottleneck, or is reflective of a deeper split in the population history of tuatara. Regardless, this population is an important source of genetic diversity in tuatara, possessing 8,480 private alleles. Although we support synonymization of *S. punctatus* and *S. guntheri*^32^, the ongoing conservation of the North Brother Island population as an independent unit is recommended.

## A cultural dimension

The tuatara is a *taonga* for many Māori—notably Ngātiwai and Ngāti Koata who are the *kaitiaki* (guardians) of tuatara. We worked in partnership with Ngātiwai *iwi* to increase knowledge and understanding of tuatara, and aid in the conservation of this species in the long term. Ngātiwai were involved in all decision-making regarding the use of the genome data by potential collaborators; for each new project we proposed, we discussed the benefits that might accrue from this work and how these could be shared. The need to engage with—and protect the rights of—Indigenous communities in such a transparent way has seldom been considered in the genome projects published to date, but is a mandated consideration under the Nagoya Protocol (https://www.cbd.int/abs/). Our partnership is a step towards an inclusive model of genomic science, which we hope others will adopt and improve upon. Although each partnership is unique, we provide a template agreement (Supplementary Information 20) that we hope will be useful to others.

## Discussion

The tuatara has a genomic architecture unlike anything previously reported, with an amalgam of features that have previously been viewed as characteristic of either mammals or reptiles. Notable among these features are unusually high levels of repetitive sequences that have traditionally been considered mammalian, many of which appear to have been recently active, and—to our knowledge—the highest level of genome methylation thus far reported. We also found a mitochondrial-genome gene content at odds with previously published reports that omitted the *ND5* gene^18^; this gene is present, nested within a repeat-rich region of the mitochondrial DNA.

Our phylogenetic studies provide insights into the timing and speed of amniote evolution, including evidence of punctuated genome evolution across this phylogeny. We also find that, in contrast to previous suggestions that the evolutionary rate for tuatara is exceptionally fast^26^, it is the slowest-evolving lepidosaur yet analysed.

Our investigations of genomic innovations identified genetic candidates that may explain the ultra-low active body temperature, longevity and apparent resistance to infectious disease in tuatara. Further functional exploration will refine our understanding of these unusual facets of tuatara biology, and the tuatara genome itself will enable many future studies to explore the evolution of complex systems across the vertebrates in a more complete way than has previously been possible.

Our population genomic work reveals considerable genetic differences among tuatara populations, and supports the distinctiveness of the North Brother Island tuatara.

Finally, this genome will greatly aid in future work on population differentiation, inbreeding and local adaptation in this global icon, the last remaining species of the once globally dominant reptilian order Rhynchocephalia.

## Methods

No statistical methods were used to predetermine sample size. The experiments were not randomized and investigators were not blinded to allocation during experiments and outcome assessment.

A full description of the methods can be found in the Supplementary Information.

### Sampling and sequencing

A blood sample was obtained from a large male tuatara from Lady Alice Island (35° 53′ 24.4′′ S 174° 43′ 38.2′′ E) (New Zealand), with appropriate ethical permissions and *iwi* consultation and support (Supplementary Information 20). Total genomic DNA and RNA were extracted and sequenced using the Illumina HiSeq 2000 and MiSeq sequencing platforms (Illumina) supported by New Zealand Genomics (Supplementary Information 1).

### Genome, transcriptome and epigenome

Raw reads were de novo-assembled using Allpaths-LG (version 49856). With a total input data of 5,741,034,516 reads for the paired-end libraries and 2,320,886,248 reads of the mate-pair libraries, our optimal assembly used 85% of the fragment libraries and 100% of the jumping libraries (Supplementary Information 1.4). We further scaffolded the assembly using Chicago libraries and HiRise (Supplementary Information 1.3).

We assembled a de novo transcriptome as a reference for read-mapping using total RNA derived from the blood of our reference male tuatara, and a collection of transcriptomic data previously collected from early-stage embryos^35^. In total, we had 131,580,633 new 100-bp read pairs and 60,637,100 previous 50-bp read pairs. These were assembled using Trinity v.2.2.0 (Supplementary Information 1.4).

Low-coverage bisulfite sequencing was undertaken using a modified post-bisulfite adaptor tagging method to explore global patterns of methylation in the genome for 12 male and 13 female tuatara (Fig. 3d, Supplementary Information 1.5).

### Repeat and gene annotation

We used a combination of ab initio repeat identification in CARP/RepeatModeler/LTRharvest, manual curation of specific newly identified repeats, and homology to repeat databases to investigate the repeat content of the tuatara genome (Supplementary Information 1.6). From these three complementary repeat identification approaches, the CARP results were in-depth-annotated for long interspersed elements and segmental duplications (Supplementary Information 4), the RepeatModeler results were in-depth-annotated for SINEs and DNA transposons (Supplementary Information 5), and the LTRharvest results were in-depth-annotated for long-terminal-repeat retrotransposons (Supplementary Information 6).

For the gene annotation, we used RepeatMasker (v.4.0.3) along with our partially curated RepeatModeler library plus the Repbase sauropsid repeat database to mask transposable elements in the genome sequence before the gene annotation. We did not mask simple repeats at this point to allow for more efficient mapping during the homology-based step in the annotation process. Simple repeats were later soft-masked and protein-coding genes predicted using MAKER2. We used anole lizard (*A. carolinensis*, version AnoCar2.0), python (*Python bivittatus*, version bivittatus-5.0.2) and RefSeq (www.ncbi.nlm.nih.gov/refseq) as protein homology evidence, which we integrated with ab initio gene prediction methods including BLASTX, SNAP and Augustus. Non-coding RNAs were annotated using Rfam covariance models (v.13.0) (Supplementary Information 7).

### Orthologue calling

We performed a phylogenetic analysis to infer orthology relationships between the tuatara and 25 other species using the Ensembl GeneTree method (Supplementary Information Tables 2.1, 2.2). Multiple-sequence alignments, phylogenetic trees and homology relationships were extracted in various formats (https://zenodo.org/record/2542571). We also calculated the gene order conservation score, which uses local synteny information around a pair of orthologous genes to compute how much the gene order is conserved. For each of these species, we chose the paralogue with the best gene order conservation score and sequence similarity, which resulted in a total set of 3,168 clusters of orthologues (Supplementary Information 2, Table 2.3).

### Gene tree reconstructions and substitution rate estimation

We constructed phylogenies using only fourfold-degenerate-site data derived from whole-genome alignments for 27 tetrapods, analysed as a single partition in RAxML v.8.2.3. Using the topology and branch lengths obtained from the best maximum likelihood phylogeny, we estimated absolute rates of molecular evolution in terms of substitution per site per million years and estimated the divergence times of amniotes via the semiparametric penalized likelihood method in r8s v.1.8 (Supplementary Information 14.5).

We also generated gene trees on the basis of 245 single-copy orthologues found between all species using a maximum-likelihood-based multi-gene approach (Supplementary Information 15). Sequences were aligned using the codon-based aligner PRANK. The FASTA format alignments were then converted to PHYLIP using the catfasta2phyml.pl script (https://github.com/nylander/catfasta2phyml). Next, we used the individual exon PHYLIP files for gene tree reconstruction using RaxML using a GTR + G model. Subsequently, we binned all gene trees to reconstruct a species tree and carried out bootstrapping using Astral (Supplementary Information 15, Supplementary Fig. 15.1).

### Divergence times and tests of punctuated evolution

We inferred time-calibrated phylogenies with BEAST v.2.4.8 using the CIPRES Science Gateway to explore divergence times (Supplementary Information 16.1). We then used Bayesian phylogenetic generalized least squares to regress the total phylogenetic path length (of fourfold-degenerate sites) on the net number of speciation events (nodes in a phylogenetic tree) as a test for punctuated evolution (Supplementary Information 16.2).

### Analysis of genomic innovations

We explored the genomic organization and sequence evolution of genes associated with immunity, vision, smell, thermoregulation, longevity and sex determination (Supplementary Information 8–13). Tests of selection were undertaken across multiple genes, including those linked to metabolism, vision and sex determination using multispecies alignments and PAML (Supplementary Information 17).

### Population genomics

Demographic history was inferred from the diploid sequence of our tuatara genome using a pairwise sequential Markovian coalescent method (Supplementary Information 18). We also sampled 10 tuatara from each of three populations that span the main axes of genetic diversity in tuatara (Supplementary Information 19, Table 19.1), and used a modified genotype-by-sequencing approach to obtain the SNVs that we used for population genomic analysis, investigations of loci associated with sexual phenotype and estimates of genetic load (Supplementary Information 19).

### Permits and ethics

This project was undertaken in partnership with Ngatiwai and in consultation with other *iwi* who are *kaitiaki* of tuatara (Supplementary Information 20). Samples were collected under Victoria University of Wellington Animal Ethics approvals 2006R12; 2009R12; 2012R33; 22347 and held and used under permits 45462-DOA and 32037-RES 32037-RES issued by the New Zealand Department of Conservation.

### Reporting summary

Further information on research design is available in the Nature Research Reporting Summary linked to this paper.

## Online content

Any methods, additional references, Nature Research reporting summaries, source data, extended data, supplementary information, acknowledgements, peer review information; details of author contributions and competing interests; and statements of data and code availability are available at 10.1038/s41586-020-2561-9.

## Supplementary information

Supplementary Information This file contains supplementary data and analyses supporting the main paper.

Reporting Summary

## Figures and Tables

**Extended Data Table 1:**
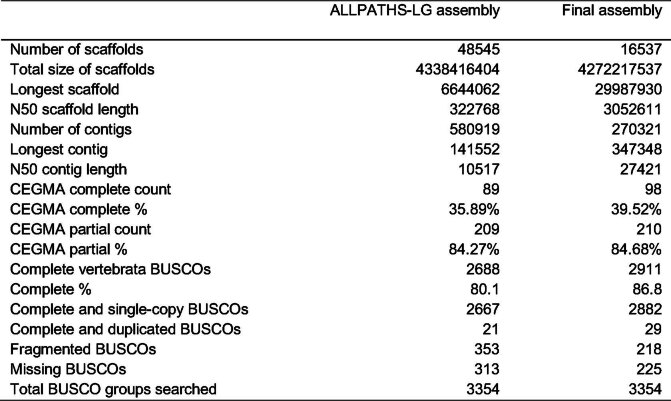
Assembly statistics and quality metrics for the tuatara genome

## Data Availability

The Tuatara Genome Consortium Project whole-genome shotgun and genome assembly are registered under the umbrella BioProjects PRJNA418887 and PRJNA445603, which are associated with BioSamples SAMN08038466 and SAMN08793959. Transcriptome read data are submitted under SRR7084910 (whole blood), together with previous data (SRR485948). The transcriptome assembly is submitted to GenBank with ID GGNQ00000000.1. Illumina short-read, Oxford Nanopore and PacBio long-read sequences are in the Sequence Read Archive accessions associated with PRJNA445603. The genome assembly (GCA_003113815.1) described in this paper is version QEPC00000000.1 and consists of sequences QEPC01000001–QEPC01016536. Maker gene predictions are available from Zenodo at 10.5281/zenodo.1489353. The repeat library database developed for tuatara is available from Zenodo at 10.5281/zenodo.2585367.
