# Publisher Correction: The tuatara genome reveals ancient features of amniote evolution

**DOI:** 10.1038/s41586-020-2661-6

**Published:** 2020-08-18

**Authors:** Neil J. Gemmell, Kim Rutherford, Stefan Prost, Marc Tollis, David Winter, J. Robert Macey, David L. Adelson, Alexander Suh, Terry Bertozzi, José H. Grau, Chris Organ, Paul P. Gardner, Matthieu Muffato, Mateus Patricio, Konstantinos Billis, Fergal J. Martin, Paul Flicek, Bent Petersen, Lin Kang, Pawel Michalak, Thomas R. Buckley, Melissa Wilson, Yuanyuan Cheng, Hilary Miller, Ryan K. Schott, Melissa D. Jordan, Richard D. Newcomb, José Ignacio Arroyo, Nicole Valenzuela, Tim A. Hore, Jaime Renart, Valentina Peona, Claire R. Peart, Vera M. Warmuth, Lu Zeng, R. Daniel Kortschak, Joy M. Raison, Valeria Velásquez Zapata, Zhiqiang Wu, Didac Santesmasses, Marco Mariotti, Roderic Guigó, Shawn M. Rupp, Victoria G. Twort, Nicolas Dussex, Helen Taylor, Hideaki Abe, Donna M. Bond, James M. Paterson, Daniel G. Mulcahy, Vanessa L. Gonzalez, Charles G. Barbieri, Dustin P. DeMeo, Stephan Pabinger, Tracey Van Stijn, Shannon Clarke, Oliver Ryder, Scott V. Edwards, Steven L. Salzberg, Lindsay Anderson, Nicola Nelson, Clive Stone, Ngatiwai Trust Board, Jim Smillie, Haydn Edmonds

**Affiliations:** 1 Department of Anatomy, University of Otago, Dunedin, New Zealand; 2 LOEWE-Center for Translational Biodiversity Genomics, Senckenberg Museum, Frankfurt, Germany; 3 South African National Biodiversity Institute, National Zoological Garden, Pretoria, South Africa; 4 School of Life Sciences, Arizona State University, Tempe, AZ USA; 5 School of Informatics, Computing, and Cyber Systems, Northern Arizona University, Flagstaff, AZ USA; 6 School of Fundamental Sciences, Massey University, Palmerston North, New Zealand; 7 Peralta Genomics Institute, Oakland, CA USA; 8 School of Biological Sciences, The University of Adelaide, Adelaide, South Australia Australia; 9 Department of Ecology and Genetics – Evolutionary Biology, Evolutionary Biology Centre (EBC), Uppsala University, Uppsala, Sweden; 10 Department of Organismal Biology – Systematic Biology, Evolutionary Biology Centre (EBC), Uppsala University, Uppsala, Sweden; 11 Evolutionary Biology Unit, South Australian Museum, Adelaide, South Australia Australia; 12 Amedes Genetics, Amedes Medizinische Dienstleistungen, Berlin, Germany; 13 Museum für Naturkunde Berlin, Leibniz-Institut für Evolutions- und Biodiversitätsforschung an der Humboldt-Universität zu Berlin, Berlin, Germany; 14 Department of Earth Sciences, Montana State University, Bozeman, MT USA; 15 Department of Biochemistry, University of Otago, Dunedin, New Zealand; 16 European Molecular Biology Laboratory, European Bioinformatics Institute, Hinxton, UK; 17 Section for Evolutionary Genomics, The GLOBE Institute, Faculty of Health and Medical Sciences, University of Copenhagen, Copenhagen, Denmark; 18 Edward Via College of Osteopathic Medicine, Blacksburg, VA USA; 19 Center for One Health Research, Virginia–Maryland College of Veterinary Medicine, Blacksburg, VA USA; 20 Institute of Evolution, University of Haifa, Haifa, Israel; 21 Manaaki Whenua - Landcare Research, Auckland, New Zealand; 22 School of Biological Sciences, The University of Auckland, Auckland, New Zealand; 23 School of Life and Environmental Sciences, The University of Sydney, Sydney, New South Wales Australia; 24 Biomatters, Auckland, New Zealand; 25 Department of Vertebrate Zoology, National Museum of Natural History, Smithsonian Institution, Washington, DC USA; 26 The New Zealand Institute for Plant and Food Research, Auckland, New Zealand; 27 Departamento de Ecología, Facultad de Ciencias Biológicas, Pontificia Universidad Católica de Chile, Santiago, Chile; 28 Department of Ecology, Evolution, and Organismal Biology, Iowa State University, Ames, IA USA; 29 Instituto de Investigaciones Biomédicas ‘Alberto Sols’ CSIC-UAM, Madrid, Spain; 30 Division of Evolutionary Biology, Faculty of Biology, Ludwig-Maximilian University of Munich, Planegg-Martinsried, Germany; 31 Centre for Genomic Regulation (CRG), The Barcelona Institute for Science and Technology, Universitat Pompeu Fabra (UPF), Barcelona, Spain; 32 School of Biological Sciences, University of Canterbury, Christchurch, New Zealand; 33 Global Genome Initiative, National Museum of Natural History, Smithsonian Institution, Washington, DC USA; 34 Austrian Institute of Technology (AIT), Center for Health and Bioresources, Molecular Diagnostics, Vienna, Austria; 35 AgResearch, Invermay Agricultural Centre, Mosgiel, New Zealand; 36 San Diego Zoo Institute for Conservation Research, Escondido, CA USA; 37 Department of Organismic and Evolutionary Biology and the Museum of Comparative Zoology, Harvard University, Cambridge, MA USA; 38 Department of Biomedical Engineering, Johns Hopkins University, Baltimore, MD USA; 39 School of Biological Sciences, Victoria University of Wellington, Wellington, New Zealand; 40 Ngatiwai Trust Board, Whangarei, New Zealand

**Keywords:** Conservation biology, Phylogenetics, Comparative genomics, Genome evolution

Correction to: *Nature* 10.1038/s41586-020-2561-9 Published online 05 August 2020

In this Article, owing to an error during the production process, the text: “a rapid increase in *N*_e_ between 500 thousand **million** years ago and 1 million years ago.”, should have read: “a rapid increase in *N*_e_ between 500 thousand years ago and 1 million years ago.”. The original Article has been corrected online.

