# Supplementary material for: The tuatara genome reveals ancient features of amniote evolution
Source: Nature. 2020 Aug 5;584(7821):403–9. doi: 10.1038/s41586-020-2561-9 (PMC7116210; doi:10.1038/s41586-020-2561-9)
Supplement: Supplementary file 1 — This file contains supplementary data and analyses supporting the main paper. [file 41586_2020_2561_MOESM1_ESM.pdf]

natureresearch

https://doi.org/10.1038/s41586-020-2561-9

Supplementary information

The tuatara genome reveals ancient features of amniote evolution

In the format provided by the authors and unedited

Supplementary Information

The tuatara genome reveals ancient features of amniote evolution

Neil J. Gemmell[1]*, Kim Rutherford[1], Stefan Prost[2,3], Marc Tollis[4,5], David Winter[6], J. Robert Macey[7], David L. Adelson[8], Alexander Suh[9,10], Terry Bertozzi[8,11], José H. Grau[12,13], Chris Organ[14], Paul P. Gardner[15], Matthieu Muffato[16], Mateus Patricio[16], Konstantinos Billis[16], Fergal J. Martin[16], Paul Flicek[16], Bent Petersen[17], Lin Kang[18], Pawel Michalak[18-20], Thomas R. Buckley[21,22], Melissa Wilson[4], Yuanyuan Cheng[23] , Hilary Miller[24], Ryan K. Schott[25], Melissa D. Jordan[26], Richard D. Newcomb[26], José Ignacio Arroyo[27], Nicole Valenzuela[28], Tim A. Hore[1], Jaime Renart[29], Valentina Peona[9,10], Claire R. Peart[9,30], Vera M. Warmuth[9,30], Lu Zeng[8], R. Daniel Kortschak[8], Joy M. Raison[8], Valeria Velásquez Zapata[28], Zhiqiang Wu[28], Didac Santesmasses[31], Marco Mariotti[31], Roderic Guigó[31], Shawn M. Rupp[4], Victoria G. Twort[21,22], Nicolas Dussex[1], Helen Taylor[1], Hideaki Abe[1], Donna M. Bond[1], James M. Paterson[32], Daniel G. Mulcahy[33], Vanessa L. Gonzalez[33], Charles G. Barbieri[7], Dustin P. DeMeo[7], Stephan Pabinger[34], Tracey Van Stijn[35], Shannon Clarke[35], Oliver Ryder[36], Scott V. Edwards[37], Steven L. Salzberg[38], Lindsay Anderson[39], Nicola Nelson[39], Clive Stone[40], Ngatiwai Trust Board[40]

1. *Allan Wilson Centre, Department of Anatomy, University of Otago, PO Box 56, Dunedin 9054, New Zealand*
2. *LOEWE-Center for Translational Biodiversity Genomics, Senckenberg Museum, 60325 Frankfurt, Germany*
3. *South African National Biodiversity Institute, National Zoological Garden, Pretoria, 0184, South Africa*
4. *School of Life Sciences, Arizona State University, 427 E Tyler Mall, Tempe, AZ 85281 USA*
5. *School of Informatics, Computing, and Cyber Systems, Northern Arizona University, 1295 S Knoles Drive, Flagstaff, AZ 86011 USA*
6. *School of Fundamental Sciences, Massey University, Private Bag 11 222, Palmerston North, 4442, New Zealand*
7. *Peralta Genomics Institute, Chancellor's Office, Peralta Community College District, 333 East 8th Street, Oakland, CA 94606*
8. *School of Biological Sciences, The University of Adelaide, Adelaide, SA 5005, Australia*
9. *Department of Ecology and Genetics - Evolutionary Biology, Evolutionary Biology Centre (EBC), Uppsala University, Norbyvägen 18D, 75236 Uppsala, Sweden.*
10. *Department of Organismal Biology – Systematic Biology, Evolutionary Biology Centre (EBC), Uppsala University, Norbyvägen 18D, SE-75236 Uppsala, Sweden*
11. *Evolutionary Biology Unit, South Australian Museum, Adelaide, SA 5000, Australia*
12. *Amedes Genetics, Amedes Medizinische Dienstleistungen GmbH, Jägerstr. 61, 10117 Berlin, Germany*
13. *Museum für Naturkunde Berlin, Leibniz-Institut für Evolutions- und Biodiversitätsforschung an der Humboldt-Universität zu Berlin. Invalidenstraße 43, 10115. Berlin, Germany*
14. *Department of Earth Sciences, Montana State University, Bozeman, MT 59717*
15. *Department of Biochemistry, University of Otago, PO Box 56, Dunedin 9054, New Zealand*
16. *European Molecular Biology Laboratory, European Bioinformatics Institute, Wellcome Genome Campus, Hinxton, Cambridge CB10 1SD, UK*
17. *Section for Evolutionary Genomics, The GLOBE Institute, Faculty of Health and Medical Sciences, University of Copenhagen, Øster Farimagsgade 5, 1353 København K, Denmark*
18. *Edward Via College of Osteopathic Medicine, Blacksburg, VA 24060, USA*
19. *Center for One Health Research, Virginia–Maryland College of Veterinary Medicine, Blacksburg, VA 24060, USA*
20. *Institute of Evolution, University of Haifa, Haifa, 3498838, Israel*
21. *Manaaki Whenua - Landcare Research, Private Bag 92170, Auckland, New Zealand*
22. *School of Biological Sciences, The University of Auckland, Private Bag 92019, Auckland, New Zealand*

23. *School of Life and Environmental Sciences, The University of Sydney, Sydney, NSW 2006, Australia*
24. *Biomatters Ltd, Level 2, 18 Shortland Street, Auckland 1010 New Zealand*
25. *Department of Vertebrate Zoology, National Museum of Natural History, Smithsonian Institution, Washington DC 20560, USA*
26. *The New Zealand Institute for Plant & Food Research, Private Bag 92169, Auckland, New Zealand*
27. *Departamento de Ecología, Facultad de Ciencias Biológicas, Pontificia Universidad Católica de Chile, Avenida Libertador Bernardo O'Higgins 340, Santiago, Chile.*
28. *Iowa State University, Department of Ecology, Evolution, and Organismal Biology, 251 Bessey Hall, Ames IA, 5011 USA.*
29. *Instituto de Investigaciones Biomédicas 'Alberto Sols' CSIC-UAM. Arturo Duperier 4. 28029-Madrid. Spain.*
30. *Division of Evolutionary Biology, Faculty of Biology, Ludwig-Maximilian University of Munich, D-82152 Planegg-Martinsried, Germany*
31. *Centre for Genomic Regulation (CRG), The Barcelona Institute for Science and Technology, Barcelona, Catalonia, Spain, Universitat Pompeu Fabra (UPF), Barcelona, Catalonia, Spain*
32. *School of Biological Sciences, University of Canterbury, New Zealand*
33. *Global Genome Initiative, National Museum of Natural History, Smithsonian Institution, 1000 Constitution Ave., Washington, DC 20560, USA*
34. *AIT Austrian Institute of Technology, Center for Health and Bioresources, Molecular Diagnostics, Muthgasse 11, 1190 Vienna, Austria*
35. *AgResearch, Invermay Agricultural Centre, Private Bag 50034, Mosgiel 9053, New Zealand*
36. *San Diego Zoo Institute for Conservation Research, Escondido, CA 92027 USA*
37. *Department of Organismic and Evolutionary Biology and the Museum of Comparative Zoology, Harvard University, Cambridge, MA 02138 USA*
38. *Department of Biomedical Engineering, Johns Hopkins University, Baltimore, MD 21205 USA*
39. *School of Biological Sciences, Victoria University of Wellington, PO Box 600, Wellington 6140, New Zealand*
40. *Ngatiwai Trust Board, 129 Port Road, PO Box 1332, Whangarei, New Zealand*

Table of contents

1 TUATARA GENOME SEQUENCING, ASSEMBLY AND ANNOTATION 8

1.1 Sampling and sequencing 8

1.2 Assembly using Allpaths-LG 8

1.3 Scaffolding the assembly with HiRise 9

1.4 Transcriptome 9

1.5 Epigenome 9

1.6 Repeat annotation 10

1.7 Gene annotation 10

2 ORTHOLOG CALLING 12

2.1 Summary 12

2.2 Methods 12

2.3 Tables 13

2.4 Figures 16

2.5 Download links 17

2.5.1 Source code 17

2.6 Command-lines 18

3 ENSEMBL ANNOTATION 19

3.1 Summary 19

3.2 Methods 19

3.2.1 Ensembl gene prediction 19

3.3 Figures 21

3.4 Tables 22

4 AB INITIO REPETITIVE DNA ANNOTATION OF THE TUATARA GENOME 23

4.1 Introduction 23

4.2 Results 23

4.2.1 Repeat coverage in Sphenodon punctatus 23

4.2.2 Classification of L2 elements in the tuatara genome 25

4.2.3 Tuatara L2 do not cluster with chicken CR1 26

4.2.4 Phylogenetic analysis of tuatara L2 compared to other vertebrates 27

4.2.5 Tuatara L2 may still be active 30

4.2.6 Possible horizontal transfer of L2 between monotreme and tuatara 33

4.2.7 Phylogenetic analysis of tuatara CR1 compared to other vertebrates 35

4.2.8 Potential active CR1 elements in the tuatara genome 37

4.2.9 Divergence rate of CR1 elements in the tuatara genome 38

4.2.10 Unclassified (un-annotated) consensus sequences are probably segmental duplications 39

4.3 Discussion 46

4.3.1 Segmental duplications in the tuatara genome 46

4.3.2 Significance of the LINE retrotransposons in the tuatara genome 47

4.4 Materials and methods 48

4.4.1 Reference genomes: repeat identification, annotation 48

4.4.1.1 Ab initio repeats identification and annotation 48

4.4.2 Analysis of LINE elements in the tuatara genome 49

4.4.3 Resolving L2 classification 49

4.4.4 Dendrogram construction from LINE nucleotide sequence alignments 49

4.4.5 Phylogenetic analysis of L2 elements using RT domain sequences 50

4.4.6 Potential horizontal transfer of L2 elements between tuatara and platypus 50

4.4.7 Divergence rate of CR1 elements in the tuatara genome 50

4.4.8 Identification of novel repeat sequences from the tuatara genome 51

4.4.9 Problems with RMD derived consensus sequences 51

5 REPEAT ANNOTATION: SINES AND DNA TRANSPOSONS 54

5.1 Methods 54

5.2 Results and discussion: DNA transposons 55

5.3 Results and discussion: SINE retrotransposons 57

6 DIVERSITY OF LTR RETROELEMENTS IN THE TUATARA GENOME 68

6.1 Introduction 68

6.2 Results 68

6.3 Discussion and conclusions 70

6.4 Methods 70

7 TUATARA GENOME: NON-CODING RNA ANNOTATION AND ANALYSIS 72

7.1 Introduction 72

7.2 Methods 72

7.3 Figures 73

7.4 Tables 74

8 THE nCpG DISTRIBUTION OF THE TUATARA GENOME AND ANALYSIS OF PATTERNS AND EVOLUTION OF CpG CONTENT IN VERTEBRATES 76

8.1 Introduction 76

8.2 Methods 77

8.2.1 Data source 77

8.2.2 Data processing 77

8.3 Results and discussion 77

8.4 Figures 80

8.5 Tables 81

9 EVOLUTION OF GENOMIC ORGANIZATION OF THE MHC 83

10 MOLECULAR EVOLUTION OF TUATARA VISUAL SYSTEM 85

10.1 Introduction 85

10.2 Methods 85

10.3 Results and discussion 86

10.3.1 Visual gene loss in tuatara and amniotes 86

10.3.2 Visual pigments and photoreceptors of tuatara 87

10.3.2.1 Selective pressures of tuatara phototransduction genes 88

10.3.2.2 Evolution of the tuatara visual system 88

10.4 Figures 90

10.5 Tables 93

11 ODORANT RECEPTORS 97

11.1 Methods 97

11.2 Results 97

12 TRANSIENT RECEPTOR POTENTIAL (TRP) ION CHANNELS GENES IN SPHENODON PUNCTATUS 100

12.1 Background 100

12.2 Methods 101

12.3 Results and discussion 101

12.4 Figures 104

12.5 Tables 108

13 SELENOPROTEINS 109

13.1 The tuatara selenoproteome 109

13.2 Multiple tRNASec gene copies 109

14 THE TUATARA GENOME AND A COMPARISON OF DNA SUBSTITUTION RATES ACROSS AMNIOTES 113

14.1 Introduction 114

14.2 Whole genome alignments 114

14.3 Data extraction 115

14.4 Phylogenetic analysis 115

14.5 Substitution rate estimation 115

14.6 Results 116

15 PHYLOGENETIC ANALYSIS OF VERTEBRATE SINGLE-COPY ORTHOLOGS 123

16 ANALYSIS OF DIVERGENCE TIMES AND A TEST FOR PUNCTUATED EVOLUTION 126

16.1 Divergence times 126

16.2 Punctuated evolution 126

17 PATTERNS OF SELECTION ON TUATARA ORTHOLOGS 129

17.1 Methods 129

17.1.1 Positive selection on the tuatara lineage 129

17.1.2 Patterns of molecular evolution at sex determining genes 129

17.2 Results 130

17.2.1 Positive selection on the tuatara lineage 130

17.2.2 Patterns of molecular evolution at sex determining genes 131

17.3 Discussion 135

18 RECONSTRUCTION OF THE DEMOGRAPHIC HISTORY OF THE TUATARA 136

18.1 Methods 136

18.2 Results 136

18.3 Discussion 136

19 POPULATION GENOMICS ANALYSES 138

19.1 Methods 138

19.1.1 Sample collection and library preparation 138

19.1.2 Read mapping and genotyping 138

19.1.3 Functional annotation of SNVs 139

19.1.4 Population genomic analysis 139

19.1.5 Differentiation with respect to sex 139

19.2 Results 140

19.2.1 Both SNV datasets support strong population structure 140

19.2.2 No loci are differentiated by sex 140

19.2.3 No evidence for excess genetic load in any population 140

20 A CULTURAL DIMENSION 146

20.1 GENOME RESEARCH AGREEMENT TEMPLATE 146

21 REFERENCES 153

1 TUATARA GENOME SEQUENCING, ASSEMBLY AND ANNOTATION

1.1 Sampling and sequencing

A blood sample was obtained from a large male tuatara from Lady Alice Island (35°53'24.4"S 174°43'38.2"E), New Zealand, with appropriate ethical and iwi permissions under permit NO-32037-RES issued by the New Zealand Department of Conservation. Total genomic DNA was extracted using proteinase K digestion and phenol-chloroform extraction, while RNA was prepared using Trizol extraction. Sequencing was undertaken using the Illumina HiSeq 2000 and MiSeq sequencing platforms (Illumina, San Diego, CA, USA). The sequencing libraries consisted of paired end (PE) libraries with insert sizes of 140 bp, 375 bp, 500 and 575 bp and three mate paired (MP) libraries with insert sizes of 2.3 kbp, 4.75 kbp and 8 kbp. The paired end libraries were prepared using the Illumina TruSeq PCR-Free DNA library kit, while the mate pair libraries were prepared using the Illumina TruSeq DNA library kit as per manufacturer's instructions. These libraries were normalised and pooled across 32 lanes on an Illumina HiSeq2000 using 2 x 100 bp paired-end sequencing at New Zealand Genomics Ltd., Dunedin. We further supplemented these data with additional Illumina TruSeq and Kappa DNA libraries, with insert sizes of 400 bp and 480 bp, respectively. These libraries were normalised and pooled across five Illumina MiSeq 2 x 250 bp runs by New Zealand Genomics Ltd., Dunedin, New Zealand.

1.2 Assembly using Allpaths-LG

Raw reads were de novo assembled using Allpaths-LG version 49856[36]). Allpaths-LG was run with several libraries with different insert sizes; overlapping paired-end libraries (PE) of 100 bp with an insert size of 180 bp, paired-end reads with insert size of 350 bp and 550 bp respectively, and several mate pair libraries (MP) with insert sizes of 5 kb, 8 kb and 9 kb. The overlapping paired-end reads were added as fragment libraries, and the 350 bp and 550 nt insert sized libraries and all MP libraries (>180 nucleotide insert size) were added as jumping libraries. Libraries with an insert size up to 550 nt were set with an inward read orientation; all others were set to be oriented away from each other in an outward direction. With a total input data of 5,741,034,516 reads for the paired-end libraries and 2,320,886,248 reads of the mate pair libraries several setups were tested for the optimal proportion of input data. Based on assembly statistics an optimal setup was found using 85% of the fragment libraries and 100% of the jumping libraries. PrepareAllPathsInputs.pl was therefore run with FRAG_COVERAGE=85. On the emergement assembly we used GapCloser[37] and then scaffolded the assembly using our transcriptome (see below) using L_RNA_Scaffolder[38].

1.3 Scaffolding the assembly with HiRise

We scaffolded the Allpaths-LG de novo assembly using HiRise, a software pipeline designed specifically for using proximity ligation data to scaffold genome assemblies[39]. We supplemented our shotgun libraries generated with 44X coverage of Chicago library reads (based on 1–50 kb pairs). Shotgun and Chicago library sequences were aligned to the draft input assembly using a modified SNAP read mapper (http://snap.cs.berkeley.edu). The separations of Chicago read pairs mapped within draft scaffolds were analyzed by HiRise to produce a likelihood model for genomic distance between read pairs, and the model was used to identify and break putative misjoins, to score prospective joins, and make joins above a threshold. After scaffolding, shotgun sequences were used to close gaps between contigs.

1.4 Transcriptome

A de novo transcriptome was assembled as a reference for read mapping and expression quantification using total RNA derived from the blood sample of our reference male using a standard RNA-Seq pipeline[40], and a collection of prior transcriptomic data collected from early stage embryos[41]. In total we had 146,739,908 read pairs comprising 131,580,633 new 100 bp read pairs and 15,159,275 prior 50 bp read pairs[41]. These were assembled using Trinity v2.2.0[42,43] running default parameters. To reduce redundancy in the assembled transcripts, CD-HIT-EST v4.6.6[44] was used to collapse contigs with at least 98% identity (e.g., representing alternative alleles from the same locus).

1.5 Epigenome

Low-coverage BS-seq was undertaken using a modified post-bisulfite adapter tagging (PBAT) method[45]. Briefly, 100 ng of purified DNA was subjected to bisulfite conversion using the EZ Methylation Direct Mag Prep kit (Zymo, D5044). Converted DNA underwent first strand synthesis with a biotin labelled adapter sequence possessing seven random nucleotides at its 3' end (BioP5N7, biotin- ACACTCTTTCCCTACACGACGCTCTTCCGATCTNNNNNNN). The product of first strand synthesis was captured using streptavidin-coated Dynabeads (Thermo, 11205D) and magnetic immobilisation. Double stranded DNA was created using the immobilized first-strand as a template and an additional adapter also possessing seven random nucleotides at its 3' end (P7N7, GTGACTGGAGTTCAGACGTGTGCTCTTCCGATCTNNNNNNN). Unique molecular barcodes and sequences necessary for binding to Illumina flow-cells were added to libraries by PCR using 1X HiFi HotStart Uracil+ Mix (KAPA, KK2801 and 10 μM indexed Truseq-type oligos, with thermal cycling as follows: 13*(94°C, 80 sec; 65°C, 30 sec; 72°C, 30 sec).

Successive rounds of paired-end 150 bp sequencing was performed on an Illumina MiSeq. Raw reads were subsequently trimmed in two steps using TrimGalore v0.4.0

(http://www.bioinformatics.babraham.ac.uk/projects/trim_galore) with stringency set to 2. First, adaptor sequences were removed and 10 bp was trimmed from the 5' end to remove nucleotide bias associated with production oligos; reads that became shorter than 105 bp were discarded to avoid bias. In the second step low-quality base calls (Phred score <20) were removed.

Trimmed reads were mapped using Bismark v0.14.3[46] with the --pbat option. Global methylation levels were calculated from Bismark reports by dividing methylated cytosine calls by total cytosine calls in both CG and non-CG contexts.

1.6 Repeat annotation

We initially annotated repeats in the assembly using RepeatModeler to generate a de novo repeat library http://repeatmasker.org/RepeatModeler.html[47]. Next, we used a combination of *ab initio* repeat identification in CARP/RepeatModeler/LTRharvest[47–49], manual curation of specific newly identified repeats, and homology to repeat databases to investigate the repeat content of the tuatara genome. From these three complementary repeat identification approaches, the CARP results were in-depth annotated for LINEs and segmental duplications (Supplementary Materials 4), the RepeatModeler results were in-depth annotated for SINEs and DNA transposons (Supplementary Materials 5), and the LTRharvest results were in-depth annotated for LTR retrotransposons (Supplementary Materials 6). Our manually curated repeat library (https://doi.org/10.5281/zenodo.2585367) was subsequently used to mask repeats in the assembly using RepeatMasker v4.0.3 (http://www.repeatmasker.org/RepeatMasker.html)[50].

1.7 Gene annotation

We used RepeatMasker[50] along with our manually curated de novo repeat database (above) to mask tandem elements in the genome sequence, but did not mask simple repeats at this point to allow for more efficient mapping during gene annotation. Simple repeats were later soft-masked during the gene annotation using MAKER2[51]. Protein-coding genes were predicted using MAKER2[51], using anole lizard (*A. carolinensis*, version AnoCar2.0), python (*P. bivittatus*, version bivittatus-5.0.2,) and RefSeq (www.ncbi.nlm.nih.gov/refseq) as protein homology evidence, which we integrated with ab-initio gene prediction methods including BLASTX, SNAP[52] and Augustus[53]. The SNAP HMM file was generated by training the anole lizard gene sequences. An Augustus model file was generated by training the tool on the OrthoDB set of 3,026 core genes of vertebrates from the genome completeness assessment tool BUSCO[54]. Predicted genes were subsequently used as query sequences in a blastx database search of the NR database (the non-redundant database, http://www.ncbi.nlm.nih.gov/). Blastx alignments with e-value greater than 1e-10 were discarded, and the top hit was used to annotate the query genes. Non-coding RNAs were annotated using Rfam covariance models (v13.0)[55] with Infernal (v1.1)[56] and tRNAscan-SE (v1.3.1)[57], with default score thresholds and parameters.

2 ORTHOLOG CALLING

Matthieu Muffato, Mateus Patricio

Correspondence to: muffato@ebi.ac.uk, mateus@ebi.ac.uk

2.1 Summary

We used the Ensembl method[58] to infer orthology relationships between the tuatara genome and another 25 species. Using sequence similarity and the conservation of local gene order, we could extract 3,168 groups of orthologs with exactly one representative gene in each species.

2.2 Methods

We performed a phylogenetic analysis to infer orthology relationships between the tuatara and another 25 species (see Supplementary Information Tables 2.1 and 2.2) using the Ensembl method[58], whose infrastructure had to be expanded to cater for externally annotated genomes (e.g. RefSeq annotations, see Supplementary Information Table 2.2). The method is composed of three main steps: (1) a clustering stage, where proteins are clustered into families using hcluster_sg based on NCBI BLAST+ e-values, (2) an alignment stage, where we compute a multiple sequence alignment of each family with M-Coffee or Mafft, and (3) a phylogenetic-tree inference and reconciliation stage using TreeBeST (the reconciliation with a species tree allows calling duplication and speciation events). The species-tree employed in this analysis was the NCBI taxonomy[59]. The computation took in total more than 900 CPU-days on the EMBL-EBI compute cluster and was orchestrated by the Ensembl Hive workflow management system[60].

We then computed an independent evidence score named "Gene Order Conservation" (GOC) score. The GOC score uses local synteny information around a pair of orthologous genes to compute how much the gene order is conserved. Supplementary Information Figure 2.1 is the distribution of the GOC scores (using tuatara as a reference) on all species, coloured according to the taxonomy and ordered by the proportion of orthologs that score 50 or higher. Fifty is chosen as a threshold here to allow genes at scaffold extremities to be selected (their GOC score being limited to 50 by construction). It shows that birds, turtles and crocodilians are the closest species (synteny-wise) to tuatara. Squamates are generally further away, with the exception of the central bearded dragon *Pogona vitticeps* which exhibits a very high level of synteny. The position of the four snakes reminds us that synteny is not necessarily correlated to evolutionary distance, but rather is a reflection of the rearrangement rate. Supplementary Information Figure 2.2 shows the pairwise conservation of synteny, highlighting the relative closeness of some clades (birds and testudines for instance) and the divergence of others (snakes).

An initial phylogenetic analysis was carried out on clusters of orthologs that have exactly one copy in all genomes, but these proved to be scarce (128 ortholog groups). This is due to the incompleteness of each assembly and genome used in the analysis, and the discrepancy between the phylogenetic history of some gene families and the species tree. In order to circumvent that, we looked at the 3,040 groups that span all species but have paralogues in at least one of them. Assuming that rearrangements are more likely to happen to a group of contiguous genes, rather than genes in isolation, we decided to use the GOC score as a selector for high-confidence orthologs[61]. For each of those species, we chose the paralogue with the best GOC score and sequence similarity. This way, we could use in total 3,168 clusters of orthologs with exactly 1 representative gene per species (see Supplementary Information Table 2.3). Data are available at: 10.5281/zenodo.2542570.

2.3 Tables

Supplementary Information Table 2.1 List of the genomes found in Ensembl

**Species name**	**Assembly name**	**Ensembl gene-set version**
Gallus gallus	Galgal4 (GCA_000002315.2)	2011-12-Ensembl
Meleagris gallopavo	UMD2 (GCA_000146605.1)	2010-09-Ensembl
Anas platyrhynchos	BGI_duck_1.0 (GCA_000355885.1)	2009-09-Ensembl
Taeniopygia guttata	taeGut3.2.4	2008-08-Ensembl
Ficedula albicollis	FicAlb_1.4 (GCA_000247815.1)	2012-05-Ensembl
Pelodiscus sinensis	PelSin_1.0 (GCA_000230535.1)	2011-11-Ensembl
Anolis carolinensis	AnoCar2.0 (GCA_000090745.1)	2010-09-Ensembl
Monodelphis domestica	BROADO5 (GCF_000002295.2)	2007-02-Ensembl
Homo sapiens	GRCh37 (GCA_000001405.14)	2010-07-Ensembl

Ornithorhynchus anatinus	OANA5 (GCF_000002275.2)	2007-01-Ensembl
Danio rerio	GRCz10 (GCA_000002035.3)	2014-09-Ensembl
Lepisosteus oculatus	LepOcu1 (GCA_000242695.1)	2012-01-Ensembl
Xenopus tropicalis	JGI_4.2 (GCA_000004195.1)	2010-09-Ensembl
Mus musculus	GRCm38 (GCA_000001635.5)	2012-01-Ensembl

Supplementary Information Table 2.2. List of non-Ensembl genomes

**Species name**	**Annotation source**	**Download link (GFFs and FASTAs)**
Sphenodon punctatus	augustus_maker	http://doi.org/10.5281/zenodo.1489354
Alligator mississippiensis	refseq	ftp://ftp.crocgenomes.org/pub/ICGWG/Genome_drafts/alligator.current/
Alligator sinensis	refseq	ftp://ftp.ncbi.nlm.nih.gov/genomes/all/GCF/000/455/745/GCF_000455745.1_ASM45574v1
Chelonia mydas	refseq	ftp://ftp.ncbi.nlm.nih.gov/genomes/all/GCF/000/344/595/GCF_000344595.1_CheMyd_1.0
Chrysemys picta	refseq	ftp://ftp.ncbi.nlm.nih.gov/genomes/all/GCF/000/241/765/GCF_000241765.3_Chrysemys_picta_bellii-3.0.3
Gekko japonicus	refseq	ftp://ftp.ncbi.nlm.nih.gov/genomes/all/GCF/001/447/785/GCF_001447785.1_Gekko_japonicus_V1.1
Protobothrops mucrosquamatus	refseq	ftp://ftp.ncbi.nlm.nih.gov/genomes/all/GCF/001/527/695/GCF_001527695.2_P.Mucros_1.0

Python molurus bivittatus	refseq	ftp://ftp.ncbi.nlm.nih.gov/genomes/all/GCF/000/186/305/GCF_000186305.1_Python_molurus_bivittatus-5.0.2
Thamnophis sirtalis	refseq	ftp://ftp.ncbi.nlm.nih.gov/genomes/all/GCF/001/077/635/GCF_001077635.1_Thamnophis_sirtalis-6.0
Ophiophagus hannah	refseq	ftp://ftp.ncbi.nlm.nih.gov/genomes/all/GCA/000/516/915/GCA_000516915.1_OphHan1.0
Ophisaurus gracilis	gigascience	http://gigadb.org/dataset/100119
Pogona vitticeps	gigascience	http://gigadb.org/dataset/100166

Supplementary Information Table 2.3 Statistics on the clusters of orthologs

Total number of ortholog clusters		23,111
Clusters with less than 26 species		19,943
Clusters with all 26 species present	*Total*	3,168
	Clusters with exactly 1 gene per species	128
	Clusters with paralogues	3,040

2.4 Figures

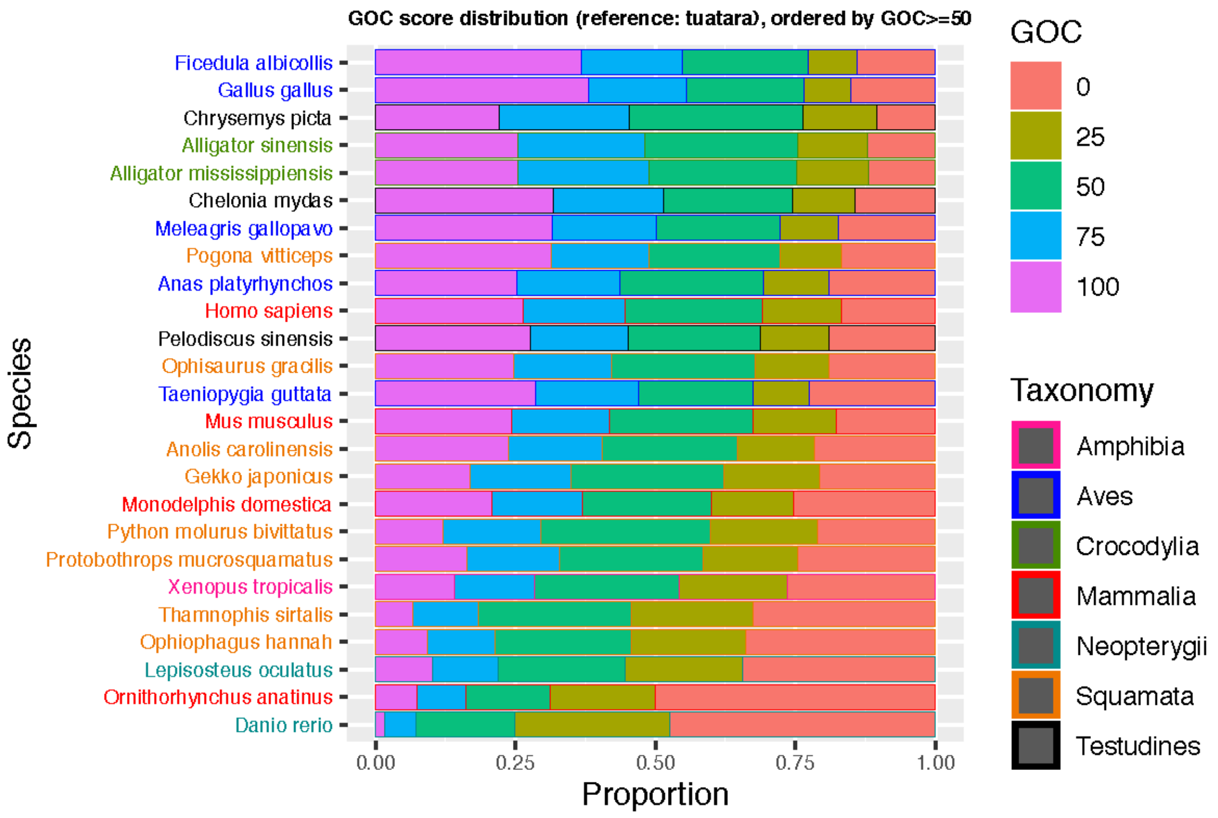

Supplementary Information Figure 2.1 Gene Order Conservation score distribution using the tuatara as the reference. Species are ordered by the proportion of top-scoring orthologs ≥50.

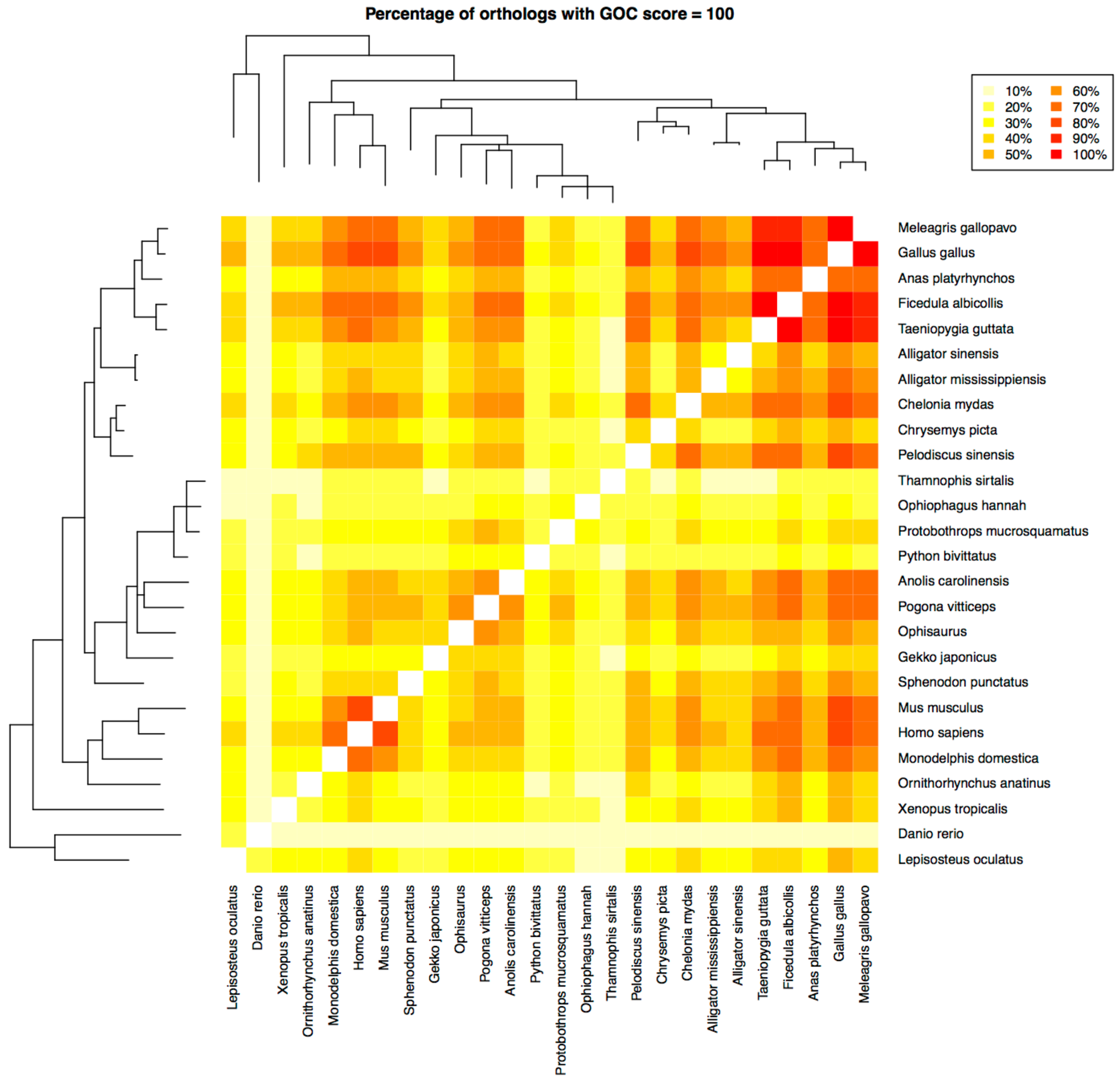

Supplementary Information Figure 2.2 Heat map representing the proportion of top-scoring orthologs between each pair of species

2.5 Download links

2.5.1 Source code

- Ensembl pipeline to compute trees and orthologs: https://github.com/Ensembl/ensembl and https://github.com/Ensembl/ensembl-compara branch “release/87”

- Ensembl Hive workflow management system https://github.com/Ensembl/ensembl-hive branch "version/2.3"
- Plotting script: https://github.com/Ensembl/ensembl-compara/blob/release/89/scripts/homology/plotGocData.r

2.6 Command-lines

- Ensembl pipeline to compute trees. After setting up paths and server locations in `ensembl-compara/modules/Bio/EnsEMBL/Compara/PipeConfig/Example/TuataraProteinTrees_conf.pm`
```
$ init_pipeline.pl
Bio::EnsEMBL::Compara::PipeConfig::Example::TuataraProteinT
rees_conf
```
  And then run the pipeline with the *beekeeper.pl* command-line that is advertised.
- Ensembl pipeline to extract Fasta, XML, TSV and EMF files from the gene-tree database.
```
$ init_pipeline.pl
Bio::EnsEMBL::Compara::PipeConfig::DumpTrees_conf -
member_type protein -clusterset_id default
```
- Extraction of clusters of orthologs
```
$ perl ensembl-
compara/scripts/examples/homologyForPhylogeny.pl -
compara_url mysql://user@server:port/database_name -
species_set_file /path/to/file_with_all_species_names -
outdir /path/to/output_dir/ -species_threshold 26 -debug 1
> /path/to/debug_output_file
```
- Generation of the plots, reference species should be defined on reference_species.dat file, one species per line.
```
$ Rscript ensembl-compara/scripts/homology/plotGocData.r
/your_source_dir/tuatara.tree /your_source_dir/
reference_species.dat
```

3 ENSEMBL ANNOTATION

Konstantinos Billis, Fergal J. Martin

Correspondence to: kbillis@ebi.ac.uk, fergal@ebi.ac.uk

3.1 Summary

Ensembl annotation, undertaken independent of the gene set annotated using MAKER2 (Supplementary Section 1.7), identified 17628 protein-coding genes, 767 pseudogenes and 910 noncoding RNAs.

3.2 Methods

3.2.1 Ensembl gene prediction

The tuatara genome assembly (version ASM311381v1) was annotated with the Ensembl gene annotation system[62]. Protein-coding gene models were annotated by combining alignments of UniProt[63] and RNA-Seq models generated from the publicly available blood sample (SRR7084910). The genome was repeat-masked with RepeatMasker, using the RepBase library (parameters: -species vertebrates) and using a custom library generated with RepeatModeler, and Dust[64]. Protein-coding models were generated by aligning reptile, bird, mammal and other vertebrate protein sequences with experimental evidence from UniProt protein existence levels 1 (evidence at the protein level) and 2 (evidence at the transcript level). These alignments were made to the repeat-masked genome in a splice aware manner using GenBlast[65]. Our in-house RNA-Seq pipeline[66] also generated protein-coding models. Reads from the tuatara blood sample (SRR7084910) were aligned to the unmasked genome using BWA[67]. The alignments were processed by collapsing the transcribed regions into a set of rough exons. Partially aligned reads were re-mapped using Exonerate[68] and this step identified spliced reads or introns. These introns together with the set of transcribed exons were combined to produce transcript models. The longest open reading frame in each of these models was BLASTed[69] against the set of UniProt vertebrate proteins with experimental evidence. For all ORFs with a BLAST match, the top hit was then aligned directly to the translated ORF using MUSCLE[70]. The alignment coverage and percent identity were calculated and stored, this was used later to help select well-supported transcripts and filter out fragmented ORFs.

Data from the above two pipelines were filtered to remove poorly supported models. Untranslated regions were added to the coding models using RNA-Seq data. The preliminary sets of coding models were combined, prioritizing well-supported models built from UniProt proteins and the RNA-Seq data at each locus. Redundant transcript structures were filtered out and the remaining models were collapsed into an initial set of protein coding genes.

The set of protein coding gene models was screened for pseudogenes. Cases where there was obvious evidence of repeated frame shifting, in-frame stop codons or genes that were completely covered in repeats were flagged as pseudogenes. Single exon protein coding genes were also examined for evidence of a multi-exon counterpart elsewhere in the genome as such cases implied likely retrotransposition events. These were flagged as processed pseudogenes.

Potential small non-coding RNA genes were initially identified by a BLAST of RFAM and miRbase[71] sequences against the genome. The resulting BLAST hits were then further filtered using RNAfold for miRNAs and Infernal[71] for other small non-coding gene types. The remaining models were screened for redundancy and the remainder formed the small non-coding gene set.

The Ensembl gene annotation for tuatara is released in Ensembl version 95, including orthologs, gene trees, and whole-genome alignments against other species. Also included are tracks for the RNA-Seq transcript models, the BAM alignment coverage and the complete set of splice junctions identified by our pipeline. An indexed BAM file for the blood sample alignments will be available for download. Additionally, the MAKER annotation generated by the tuatara consortium will be available in Ensembl as a supplementary gene track.

Data for tuatara can be accessed in Ensembl in a variety of ways including the genome browser, the Perl and REST APIs, the ftp site, BioMart and direct access to our MySQL databases.

3.3 Figures

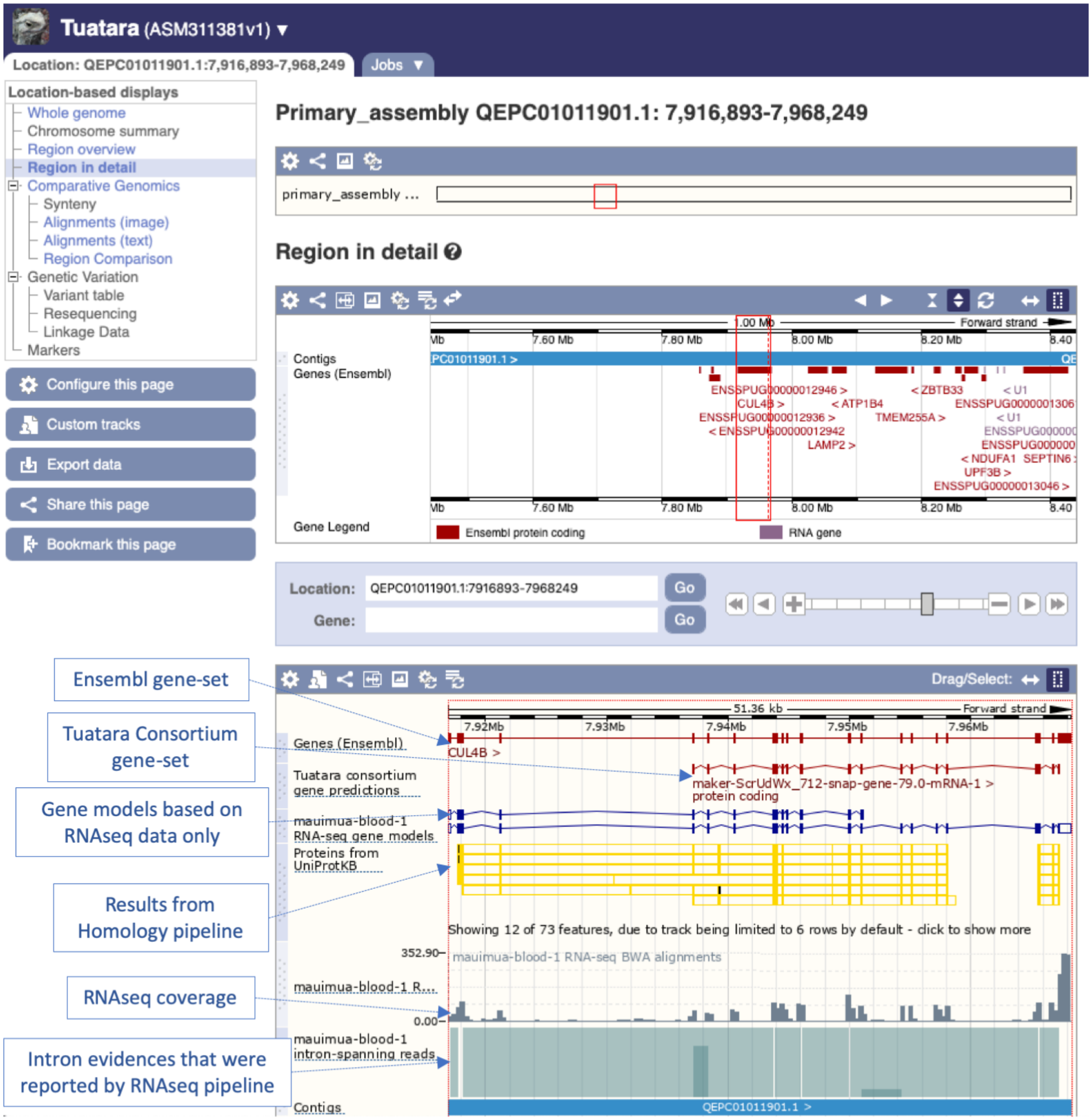

Supplementary Information Figure 3.1 Tuatara genome annotation as seen in the Ensembl browser. The Location tab allows the visualisation of genes, sequence conservation, and other annotation aligned to the genome. The lower panel, called the Region in Detail view, displays the following tracks: aligned proteins from the Homology pipeline, RNAseq models that built based on RNAseq pipeline, RNAseq coverage of the region, introns that were reported by RNAseq pipeline and the two available gene sets from Ensembl and the Tuatara Consortium.

3.4 Tables

Supplementary Information Table 3.1 Gene and transcript counts for tuatara.

**Biotype**	**Gene counts**	**Transcript counts**
Protein coding	17628	25240
Pseudogene	767	767
ncRNA	910	910
IG genes	20	20

4 *AB INITIO* REPETITIVE DNA ANNOTATION OF THE TUATARA GENOME

Lu Zeng, Terry Bertozzi, R. Daniel Kortschak, Joy M. Raison, David L. Adelson*

*Correspondence to: david.adelson@adelaide.edu.au

4.1 Introduction

In this section, we use an *ab initio* repeat identification and annotation method[48] to investigate the repeat content of the tuatara genome. In order to evaluate the performance of our *ab initio* method, we have compared the repeat libraries generated from our method and from RepeatModeler[47] (RMD). Our analysis revealed that most repeats in the tuatara genome are non-LTR LINE L2 retrotransposons. We also investigated the evolutionary relationships between tuatara L2 sequences and those from other vertebrates using both the full nucleotide sequences and the Reverse Transcriptase (RT) domains of these L2 sequences. We found two main sub-families of L2 in the tuatara, one similar to lizard L2s and the other similar to platypus L2s. The latter provide potential evidence for a horizontal transfer event of L2s between lizards and monotremes. Finally, there were a large number of repeated sequences that remain unclassified from our *ab initio* method and these are likely segmental duplications in the tuatara genome. However, some highly repeated sub-sequences in these duplications are potential novel non-autonomous transposons.

4.2 Results

4.2.1 Repeat coverage in *Sphenodon punctatus*

Using the *ab initio* method, around 64% of the reference tuatara genome was annotated as repetitive (Supplementary Information Table 4.1), and 31% of the genome comprised known repeats. This indicated that the tuatara genome was significantly enriched with repeats compared to other reptile genomes. One interesting observation was that the fraction of non-LTR retrotransposons in the tuatara genome was much higher (16.5%) than in placental mammals, with L2 being the dominant LINE element. We estimated that there are 1.4 million L2 sequences (full length and fragments) in the tuatara genome, and the longest L2 sequences we found were approximately 4 kb in length. The second most abundant repeat type was CR1 (chicken repeat 1), which comprised 3% of the tuatara genome.

In contrast to our method, RepeatModeler (RMD) identified approximately 51% of the tuatara genome as repetitive, and 36% of this was annotated based on Repbase data. L2 and CR1 were also the two dominant non-LTR retrotransposons. RMD identified 9.6% of the tuatara genome as DNA transposons, mainly from the DNA/hAT (hobo/AC/Tam3) superfamily (4.5%) and the Harbinger superfamily (4.2%). However, further analysis of the RMD consensus sequences casts significant doubt on the accuracy of these findings and this identification needs to be validated (see Supplementary Materials 4.4.9 Problems with RMD derived consensus sequences).

Supplementary Information Table 4.1: Copy number and percentage of tuatara genome covered by interspersed repeats.

	ab initio library			RMD library		
Group	Number	Total bp	Percentage	Number	Total bp	Percentage
Non-LTR						
LINE L2	1,479,425	428,138,325	10.022	1,149,043	491,620,677	11.507
LINE CR1	815,011	162,227,767	3.797	500,647	149,462,580	3.498
LINE RTE	138,126	31,151,317	0.729	184,481	97,841,699	2.290
LINE L1	239,829	30,179,939	0.706	49,905	29,081,314	0.681
SINE	329,988	32,931,264	0.771	184,701	27,664,250	0.647
Others	209,995	21,413,494	0.501	46,718	21,910,444	0.514
	3,212,374	706,042,106	16.526	2,115,495	817,580,964	19.137
ERV						
ERV1	142,313	17,564,350	0.411	20,968	9,453,167	0.221
Others	145,005	15,355,898	0.359	7,149	1,948,963	0.046
	287,318	32,920,248	0.770	28,117	11,402,130	0.267
DNA transposons						
hAT	439716	57197609	1.339	668,329	194,724,290	4.558
Harbinger	435048	80071368	1.874	463,162	180,461,900	4.224
Others	1,564,326	224,599,096	5.257	167,048	36,855,177	0.863
	2439090	361868073	8.470	1,298,539	412,041,367	9.645
LTRs						
DIRS	256,478	58,538,598	1.370	130,860	78,246,082	1.831
Gypsy	570,277	83,980,917	1.966	98,683	62,432,545	1.461
LTR others	221,043	26,438,648	0.619	259,570	115,859,366	2.713
	1,047,798	168,958,163	3.955	489,113	256,537,993	6.005
Others	599,649	67,376,623	1.578	316,948	58,30,9,055	1.364
Well-annotated	7,586,229	1,337,165,213	31.299	4,248,212	1,555,871,509	36.418
Unknown (<99.9%)	5,829,980	1,065,036,029	24.929	3,203,800	637,030,810	14.911
Total	14,015,858	2,469,577,865	57.806	7,452,012	2,192,902,319	51.329

4.2.2 Classification of L2 elements in the tuatara genome

L2 elements were found to be the most abundant repeat type in the tuatara genome using both repeat annotation methods. In the RMD repeat library, 29 L2 sequences were longer than 2 kb, with 9 of them approximately 3 kb long. In our *ab initio* library, 27 consensus sequences were longer than 2 kb with one over 3 kb long. Supplementary Information Figure 4.1 shows the sequence similarity of L2 elements (2–4 kb) from these two methods. RMD L2 sequences were found to cluster with our L2 consensus sequences, and were most similar to turtle, platypus L2s, and anole L2 based on CENSOR annotation. In addition, L2 consensus sequences from our method that were annotated as most similar to platypus L2, were also found always clustered with turtle L2 based on CENSOR annotation. However, the Repbase library classified platypus L2 as CR1. This is one example of the confusing state of repeat annotations and nomenclature. The remaining L2 consensus sequences (including the longest L2) were found to be similar to anole L2 sequences based on Repbase annotation.

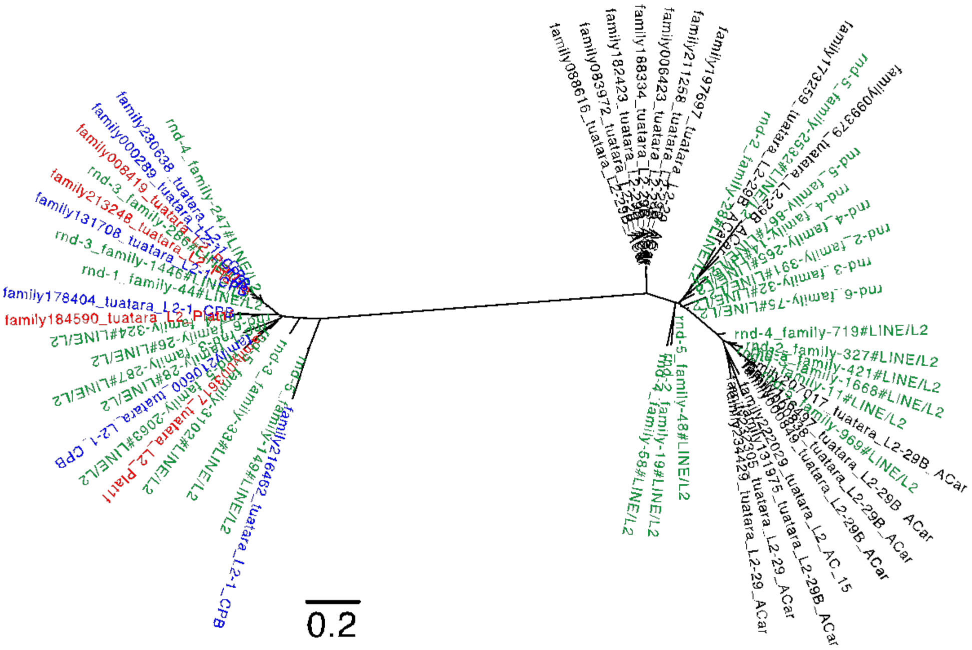

Figure 4.1: Sequence similarity analysis of RMD L2 and *ab initio* L2 consensus sequences. Shown is a Maximum likelihood dendrogram inferred using FastTree using a global alignment of tuatara L2 nucleotide sequences generated from both our *ab initio* method and RMD. Green labels identify RMD tuatara sequences, while other colours denote tuatara L2 sequences similar to turtle (blue), platypus (red), and anole (black).

4.2.3 Tuatara L2 do not cluster with chicken CR1

As mentioned above, platypus L2 sequences are categorised as belonging to the CR1 clade according to Repbase. Therefore, we used full-length chicken CR1 sequences to analyse the overall

clustering pattern of tuatara L2 sequences (Supplementary Information Figure 4.2). With the inclusion of chicken CR1 sequences, tuatara L2 sequences split into 4 groups; one group contains repeats similar to platypus L2, turtle L2 and RMD L2, the other three groups are made up of L2 sequences that are most similar to anole. The full-length chicken CR1 elements did not cluster with the L2 consensus sequences, indicating that all of the L2 sequences we have identified are not CR1 like, in spite of the Repbase annotation.

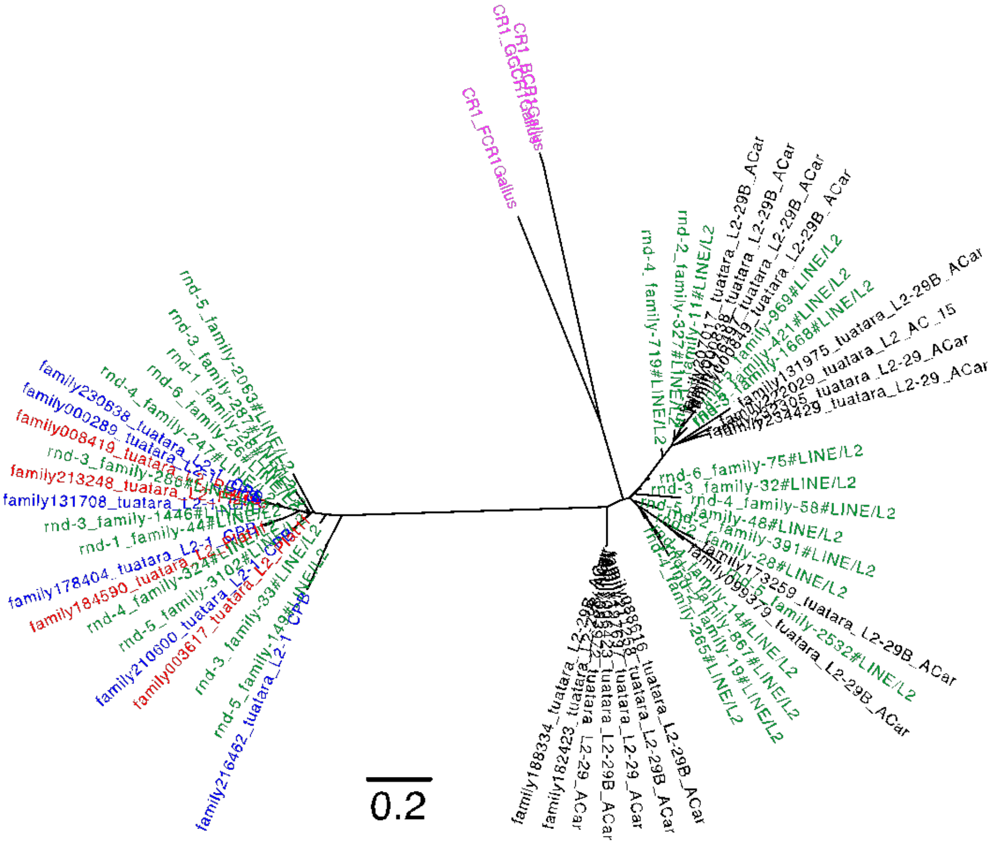

Supplementary Information Figure 4.2: Tuatara L2 and chicken CR1 do not cluster together. Maximum likelihood dendrogram inferred using FastTree based on global alignment of tuatara L2 nucleotide sequences and chicken CR1. All sequences were generated from our *ab initio* method, except green labelled sequences, which are from RepeatModeler. Dark blue labels are tuatara L2 sequences similar to turtle, red labels are tuatara L2 similar to platypus, black labels are L2 sequences similar to anole, green labels are RMD tuatara sequences, and pink labels are chicken CR1 from Repbase.

4.2.4 Phylogenetic analysis of tuatara L2 compared to other vertebrates

We then globally aligned the L2 tuatara sequences with L2 sequences from other vertebrates. The resulting maximum likelihood based phylogenetic analysis clearly showed that tuatara L2 sequences are divided into two groups (Supplementary Information Figure 4.3), one group is more

similar to bearded dragon and anole L2 (L2 from RMD and our *ab initio* method), while the other group clustered with the turtle and platypus L2 (L2 from RMD and our *ab initio* method). Platypus-like L2 consensus sequences identified from our *ab initio* method consistently clustered with platypus L2 from Repbase. The presence of two lineages of L2 (reptile vs monotreme) in tuatara is not characteristic of other reptiles and may be the result of incomplete lineage sorting of L2 in tuatara or raises the possibility of horizontal transfer of L2 between monotremes and the tuatara lineage.

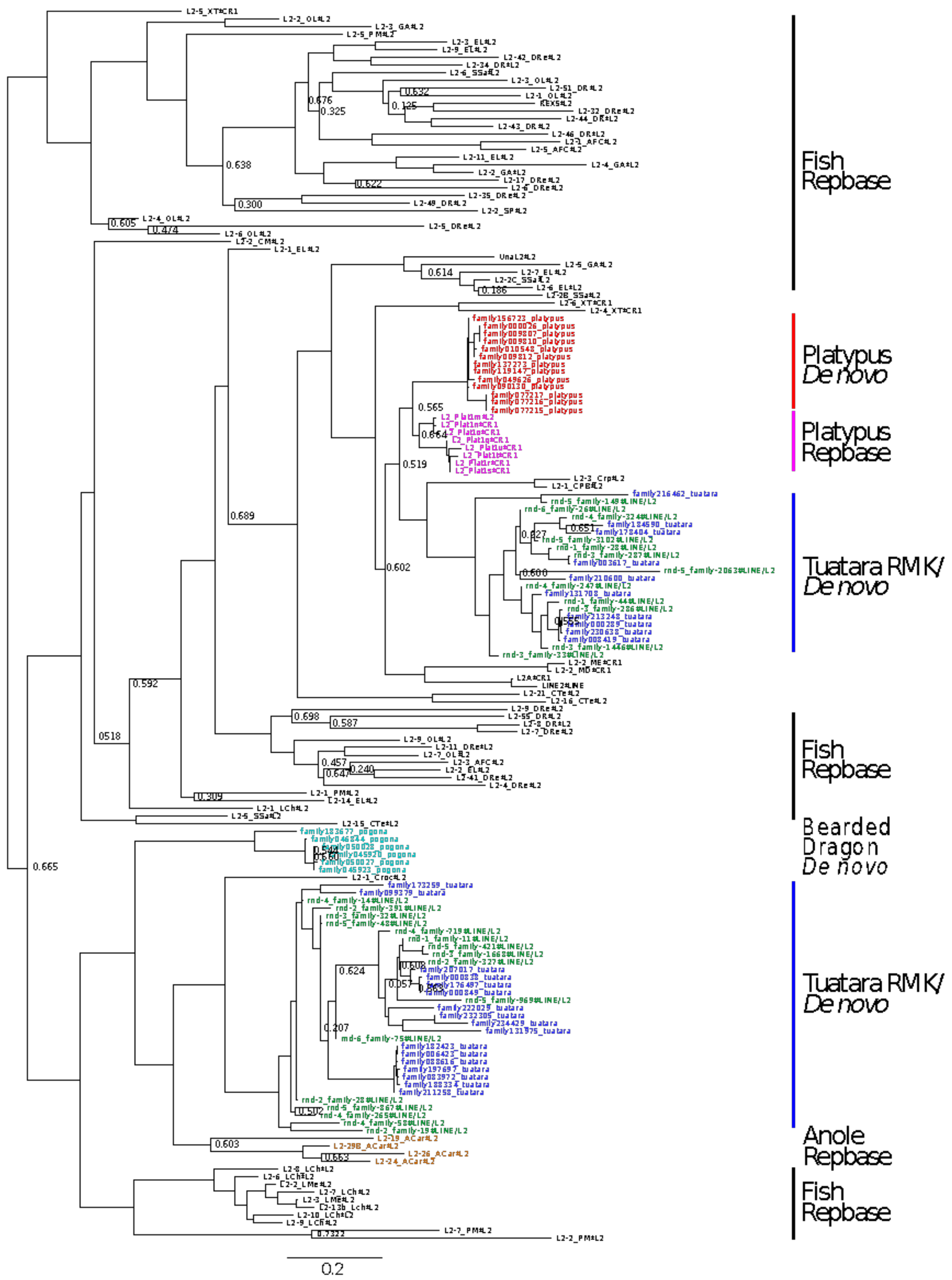

Supplementary Information Figure 4.3: Phylogenetic analysis of vertebrate L2. Maximum likelihood dendrogram inferred using FastTree based on global alignment of

L2 nucleotide sequences. L2 sequences were either extracted from full genome assemblies (tuatara, platypus and pogona) or sourced from Repbase. Sequences were aligned with MUSCLE, and visualised with FigTree. Branches are coloured to indicate the original L2 source. Dark blue labels are tuatara L2 sequences generated using our *ab initio* method, dark green labels are tuatara L2 sequences from RepeatModeler, pink labels are platypus sequences from Repbase, red labels are platypus sequences from our *ab initio* method, light green labels are pogona *ab initio* sequences and orange labels are anole sequences from Repbase.

4.2.5 Tuatara L2 may still be active

We plotted the sequence divergence of the L2 elements in order to determine if there were any that appeared to have recently been retrotransposed (Supplementary Information Figure 4.4). It was clear from this analysis that there are probably active L2 elements in the tuatara genome.

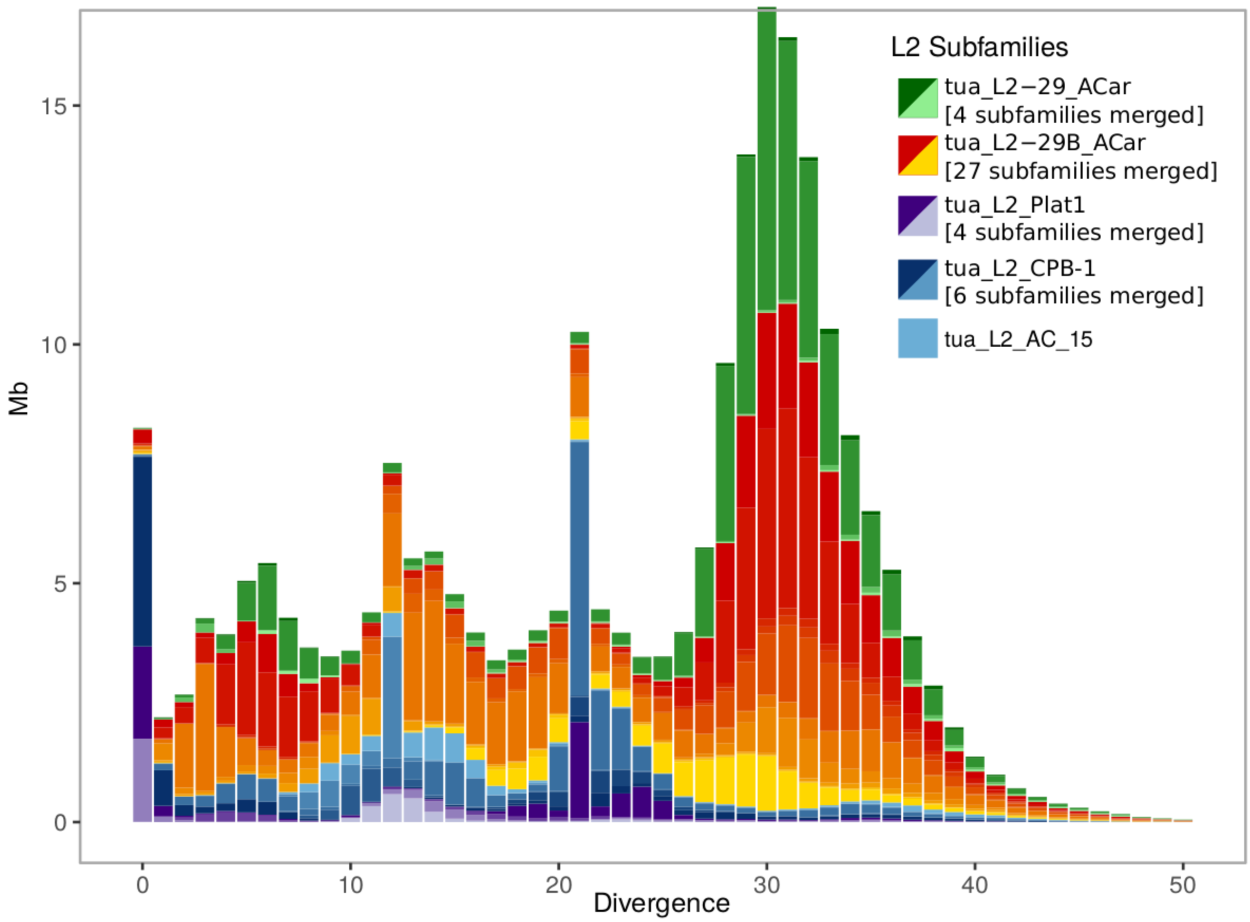

Supplementary Information Figure 4.4. Kimura substitution levels of tuatara L2 insertions. Landscape plot of L2 retrotransposons, the dominant LINEs observed in the tuatara genome, accounting for 10% of the genome. Only LINE subfamilies that occupy

more than 1,000 bp are shown. Note the spike at 0% divergence indicating recently/currently active L2 families.

Based on the above, we undertook an open reading frame (ORF) analysis of the long tuatara L2 consensus sequences (2–4 kb) and their corresponding genomic sequences to identify potentially active L2 elements. This also reduced the confounding effects of length in Supplementary Information Figures 4.1 and 4.2, which only looked at sequence similarity within the tuatara L2 sequences we identified.

With respect to our *ab initio* consensus sequences, we found that 27 of the tuatara consensus sequences were longer than 2 kb, with 1 of them longer than 3 kb. We also found that 13 (48%) of these consensus sequences appeared to contain intact open reading frame 2 (ORF2p) (longer than 500 amino acids and contained a RVT_1 reverse transcriptase motif). While in tuatara genomic sequences we found that 639 of the tuatara genome fragments were longer than 2 kb, with four of them longer than 3 kb. Significantly, we found that 530 (83%) of these genomic sequences appeared to contain intact ORF2p, see Supplementary Information Table 4.2. This strongly suggests that L2 elements may still be active in the tuatara genome.

Supplementary Information Table 4.2: Copy number and fraction of the tuatara genome covered by interspersed repeats.

	3–4 kb		2–3 kb	
	Number full length	% active L2	Number full length	% active L2
RMD L2 consensus	9	44	20	30
ab initio L2 consensus	1	100	26	46
ab initio L2 genome	4	100	635	83

We also carried out global alignments of >500aa long ORF2p sequences from tuatara and Repbase consensus sequences that had RT domains >200aa in length (see Methods). The phylogenetic analysis of RT families (Supplementary Information Figure 4.5) clearly illustrated differences between L2 groups. This figure shows that tuatara L2 RT domains differed from anole and split into two clusters, one cluster was closer to platypus, while the other cluster was closer to bearded dragon. The L2 RT domain from crocodile diverged from all other species. Although the L2 nucleotide phylogenetic tree showed that tuatara L2 shared high similarity with anole L2, when comparing sequences based on the RT domain, they were quite different, with anole RT domains clearly separated from other species. This result is consistent with either incomplete lineage sorting

of two L2 families or horizontal transfer of L2 sequences to, or from, a monotreme. Both of these families may still be active.

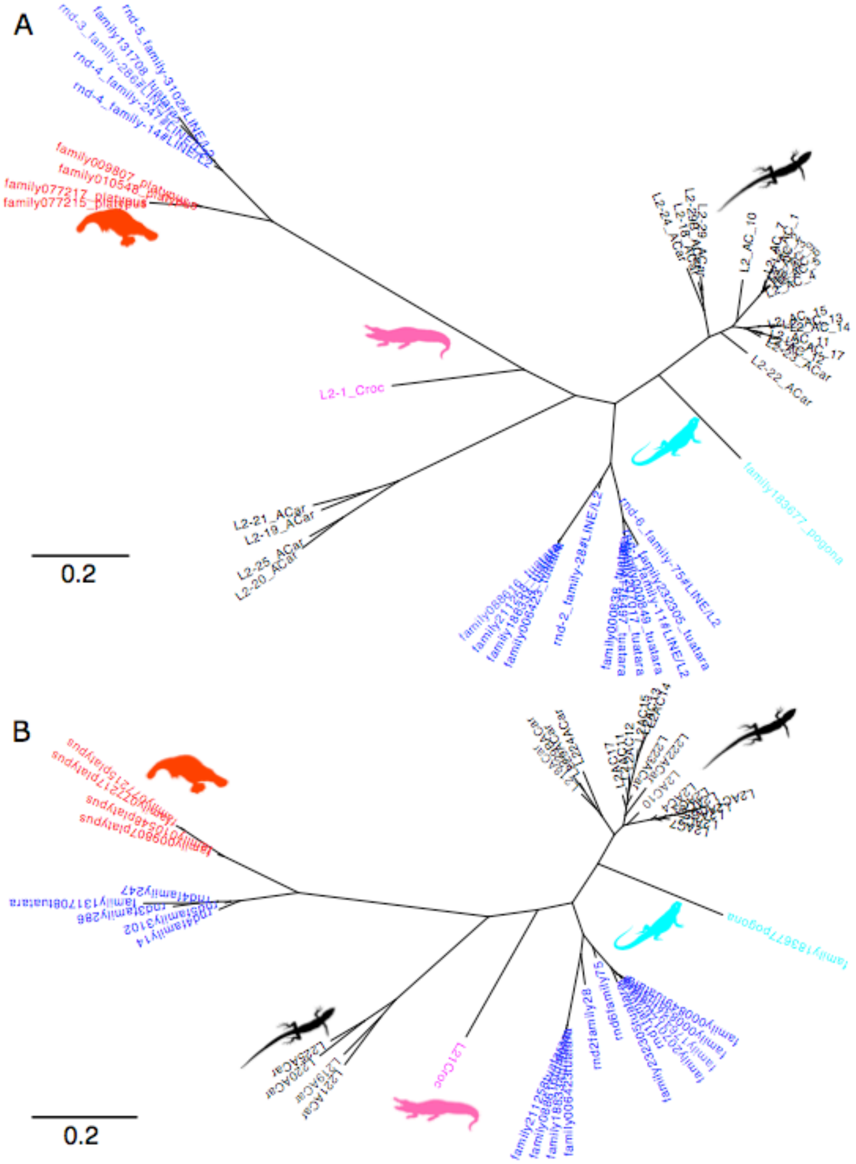

Supplementary Information Figure 4.5: Phylogenetic analysis based on the RT domain. A) Maximum likelihood dendrogram inferred using FastTree from L2 RT domain multiple alignment. Branches are coloured to indicate the original L2 source: dark blue labels are tuatara L2 RT domain generated from our *ab initio* method and RepeatModeler, red branches are platypus L2 RT domains, light blue branches are L2 RT domains from bearded dragon (pogona) and black branches were L2 RT domains from anole. B) Bayesian dendrogram inferred using MrBayes from L2 RT domain multiple alignment. Branch labelling same as panel A. Silhouettes are Designed by Creazilla https://creazilla.com/.

4.2.6 Possible horizontal transfer of L2 between monotreme and tuatara

Based on both nucleotide and RT domain phylogenies, L2 elements consistently split into two groups, one most similar to lizard L2 (33 consensus sequences), the other most similar to platypus L2 (22 consensus sequences), indicating a possible horizontal transfer event between monotreme and tuatara. We used these sequences to generate custom L2 libraries for repeat annotation (see below).

We developed a method based on reciprocal best hits for identifying horizontally transferred sequences. First, CENSOR was used to find hits that annotated as platypus-like tuatara L2 or non-platypus-like tuatara L2 in five reptile genomes (anole, alligator, crocodile, turtle and bearded dragon) and one monotreme genome (platypus) using the custom L2 libraries. Second, the CENSOR hit sequences were extracted and used as BLASTN queries to find reciprocal best hits in the custom libraries. This allowed us to determine the reliability of the original annotation of L2 hits and determine the L2 family they are most likely to belong to.

Based on the initial CENSOR output (Supplementary Information Table 4.3), platypus-like tuatara L2 elements were found to be enriched in the platypus genome, while non-platypus tuatara L2 elements were abundant in both the anole and bearded dragon genomes. Validation of these hits using reciprocal BLASTN (Supplementary Information Table 4.4) shows that although many fragments in reptiles were annotated as platypus-like tuatara L2 by CENSOR, they did not validate as reciprocal hits. This is most likely a product of different parameter settings for BLASTN used by CENSOR and the more stringent default settings used in the reciprocal BLASTN search. However, 16/22 platypus-like L2 tuatara consensus sequences were validated as being most similar to platypus L2 based on their reciprocal best hits. Similarly, 21/33 reptile-like tuatara L2 consensus sequences were validated as non-platypus-like based on the reciprocal best-hit results.

Supplementary Information Table 4.3: CENSOR output. Summary of non-redundant CENSOR hit genome intervals used as queries for reciprocal BLASTN alignment. Number of consensus sequences in custom library (in parentheses). PT L2 = Platypus-like tuatara L2; NPT L2 = Non-platypus-like tuatara L2; int = Intervals; con = Consensus.

CENSOR library	Anole		Alligator		Crocodile		Bearded dragon		Turtle		Platypus	
	int	(con)	int	(con)	int	(con)	int	(con)	int	(con)	int	(con)
PT L2 (22)	26,954	(22)	37,502	(22)	34,842	(22)	27,445	(22)	103,729	(22)	406,615	(22)
NPT L2 (33)	213,764	(33)	20,584	(33)	19,717	(33)	127,972	(33)	46,802	(33)	258,675	(33)

Supplementary Information Table 4.4: BLASTN output. Summary of the BLASTN outputs for the reciprocal alignments of the intervals from Supplementary Information Table 4.3 against platypus-like tuatara L2 and non-platypus-like tuatara L2 consensus sequences. Number of validated consensus sequences (in parentheses).

L2 Type	Anole		Alligator		Crocodile		Bearded dragon		Turtle		Platypus	
	int	(con)	int	(con)	int	(con)	int	(con)	int	(con)	int	(con)
PT L2 (22)	0	(0)	0	(0)	0	(0)	0	(0)	1	(1)	2,281	(16)
NPT L2 (33)	761	(21)	21	(6)	25	(6)	759	(22)	5	(2)	0	(0)

Furthermore, in order to estimate the relative ages of platypus-like tuatara L2 and non-platypus-like tuatara L2, super consensus were built for each of L2 types (see Section 4.4), RepeatMasker[50] was then used to calculate the divergence rate between super consensuses and their corresponding tuatara L2 elements (e.g. platypus-like tuatara L2 super consensus against platypus-like tuatara L2 elements). Supplementary Information Figure 4.6 shows that compared to non-platypus-like tuatara L2, platypus-like tuatara L2 has a lower sequence substitution level, which indicates that the platypus-like tuatara L2 sequences are of more recent origin, and may have resulted from horizontal transfer from platypus, or some intermediate vector, into the tuatara genome.

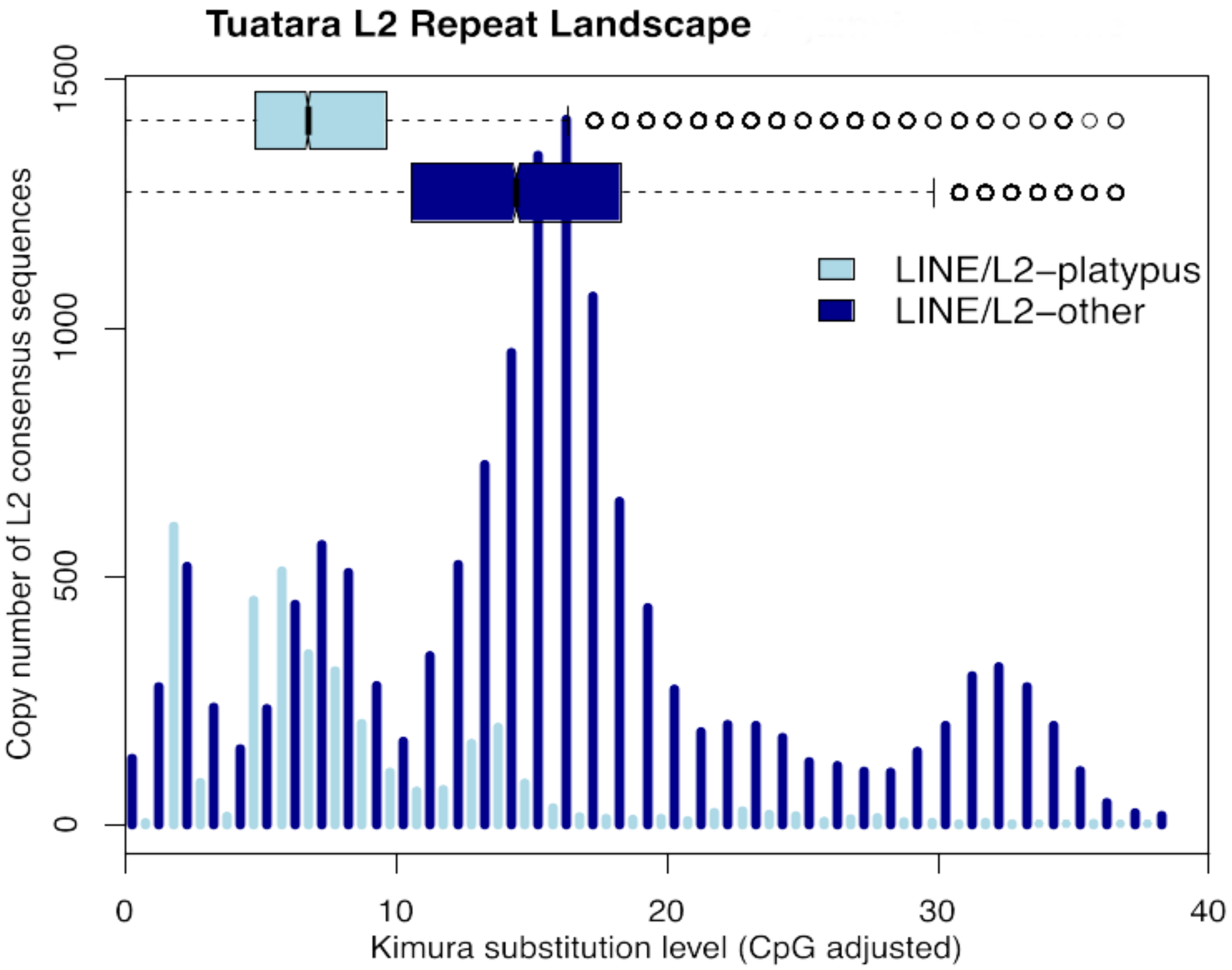

Supplementary Information Figure 4.6: Kimura substitution level of tuatara L2 elements. The divergence rate of platypus-like tuatara L2 (number, n, of copies: 3,485; min:0; max:38; percentiles: 0th (0), 25th (5), 50th (7), 75th (10), 100th (17)) and non-platypus-like tuatara L2 (number n of copies: 14,171; min:0; max: 38; percentiles: 0th (0), 25th (11), 50th (15), 75th (19), 100th (31)) was calculated using the kimura 2-Parameter divergence metric, and adjusted for 'GC' content. Dark blue bars show the sequence substitution rate of non-platypus-like tuatara L2 against non-platypus-like L2 super consensus, while light blue bars show the sequence substitution rate of platypus-like tuatara L2 against platypus-like L2 super consensus. Each pair of bars shares the same substitution level. Boxplot shows the upper and lower quartiles, the mean value of each tuatara L2 type and 0 and 100th percentile, with respect to their divergence rate.

4.2.7 Phylogenetic analysis of tuatara CR1 compared to other vertebrates

Based on both repeat annotation methods (Supplementary Information Table 4.1), CR1 elements were the second most abundant LINE in the tuatara genome, making up ~3% of the genome.

Thirty-nine of these CR1 consensus sequences were found to be longer than 2.5 kb, potentially with an intact ORF2. The longest CR1 sequence found in the tuatara genome is 4,605 bp. We globally aligned these CR1 tuatara sequences with CR1 sequences from other vertebrates (231 in total). The resulting maximum likelihood based phylogenetic analysis showed that tuatara CR1 sequences were distinct from those found in other species, including the anole lizard (Supplementary Information Figure 4.7).

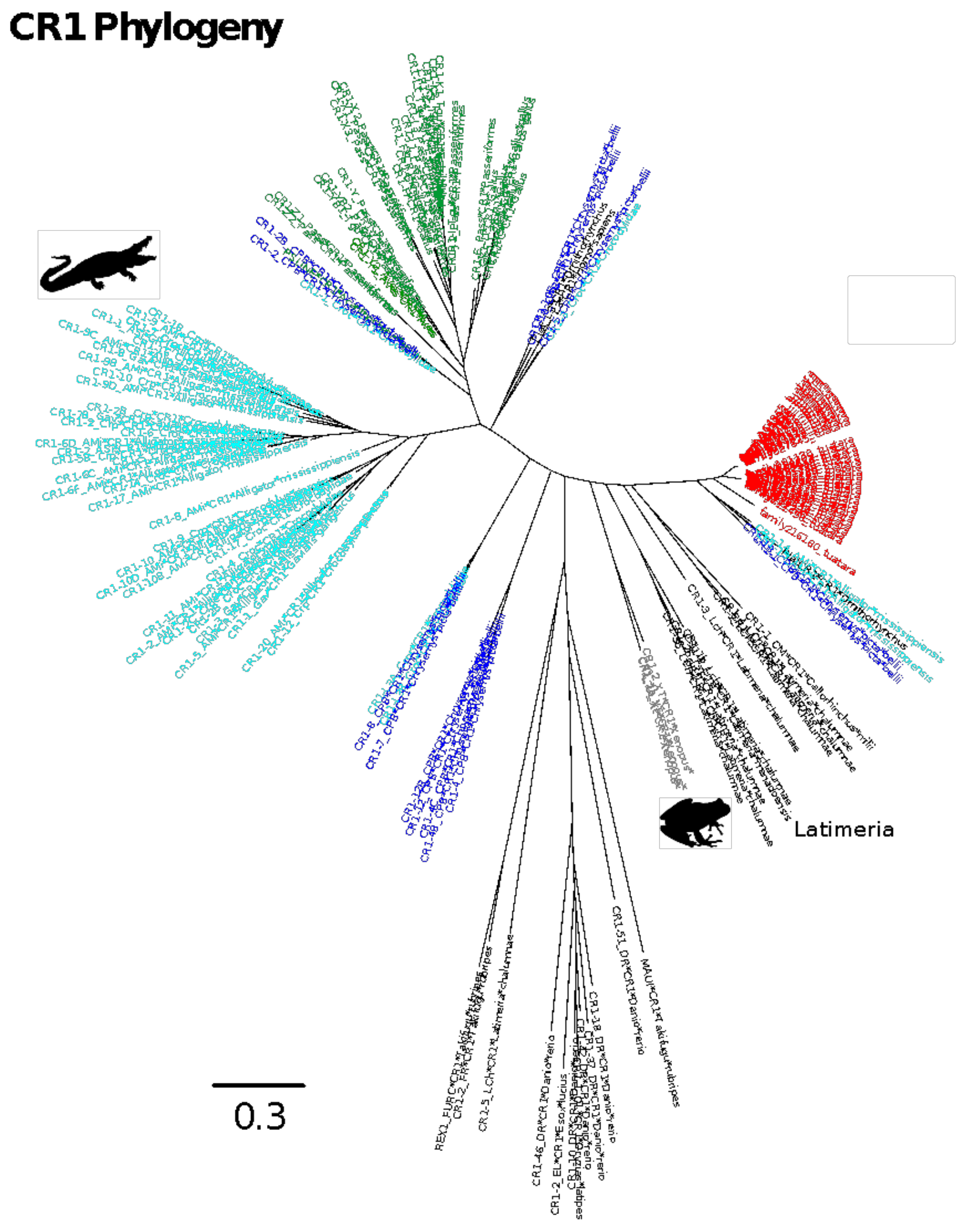

Supplementary Information Figure 4.7: Phylogenetic analysis of vertebrate CR1. Maximum likelihood dendrogram inferred using FastTree based on global alignment of CR1 nucleotide sequences. CR1 sequences were extracted from species full genome assemblies (tuatara and anole lizard) using CARP[48] or sourced from RepBase.

Sequences were aligned with MUSCLE and visualised with Archaeopteryx. Branch colours indicate the original CR1 source. Red labels are tuatara, dark blue labels are turtle, dark green labels are anole lizard, light blue labels are crocodilian sequences, pink labels are platypus sequences, black labels are bird CR1 sequences, and light grey labels are frog CR1 sequences. Silhouettes are Designed by Creazilla https://creazilla.com/.

4.2.8 Potential active CR1 elements in the tuatara genome

We then further analysed the 39 CR1 consensus sequences that were longer than 2.5 kb, 13 of which contained an intact RT domain. Tuatara CR1 were split into two groups, which may represent two classes of CR1 elements in the tuatara genome. (Supplementary Information Figure 4.8).

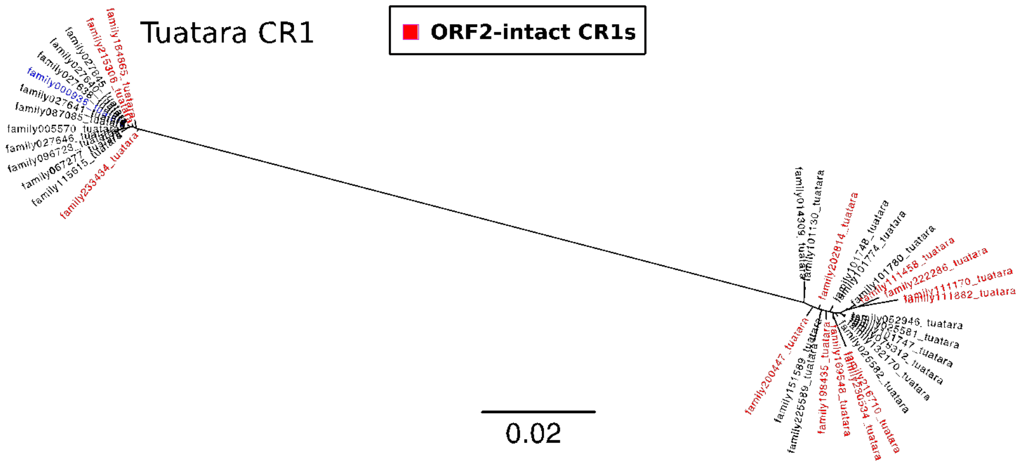

Supplementary Information Figure 4.8: Phylogenetic analysis of CR1 elements in the platypus genome. Dendrogram of full-length CR1 elements in the tuatara genome. Sequences were aligned with MUSCLE, trees inferred with FastTree and visualized with Archaeopteryx. ORF2-intact CR1s are shown with a red label at the tip of the branch, and the longest CR1 is labelled with dark blue.

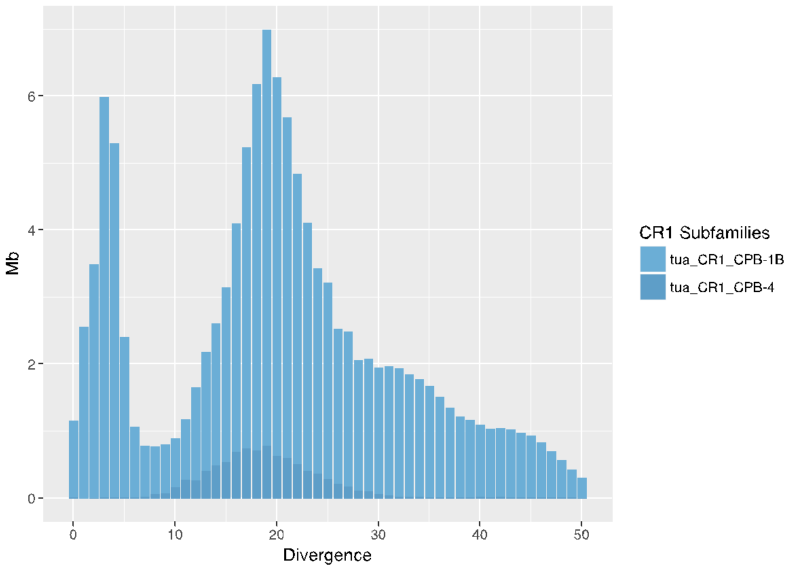

Supplementary Information Figure 4.9: The landscape of full-length CR1 in the tuatara genome. Long CR1 subfamilies with a total length greater than 1,000 bp and with distances to consensus <50% are shown.

We also calculated the divergence rate of these potentially active CR1 elements, Supplementary Information Figure 4.9 shows that the two classes of CR1 elements in the tuatara genome appear to have different CR1 activity profiles over time.

4.2.9 Divergence rate of CR1 elements in the tuatara genome

Considering all CR1 elements in the tuatara genome, we identified 14 CR1 subfamilies (Supplementary Information Figure 4.10); some of which had low divergence compared to consensus, indicating a recent wave of CR1 insertion.

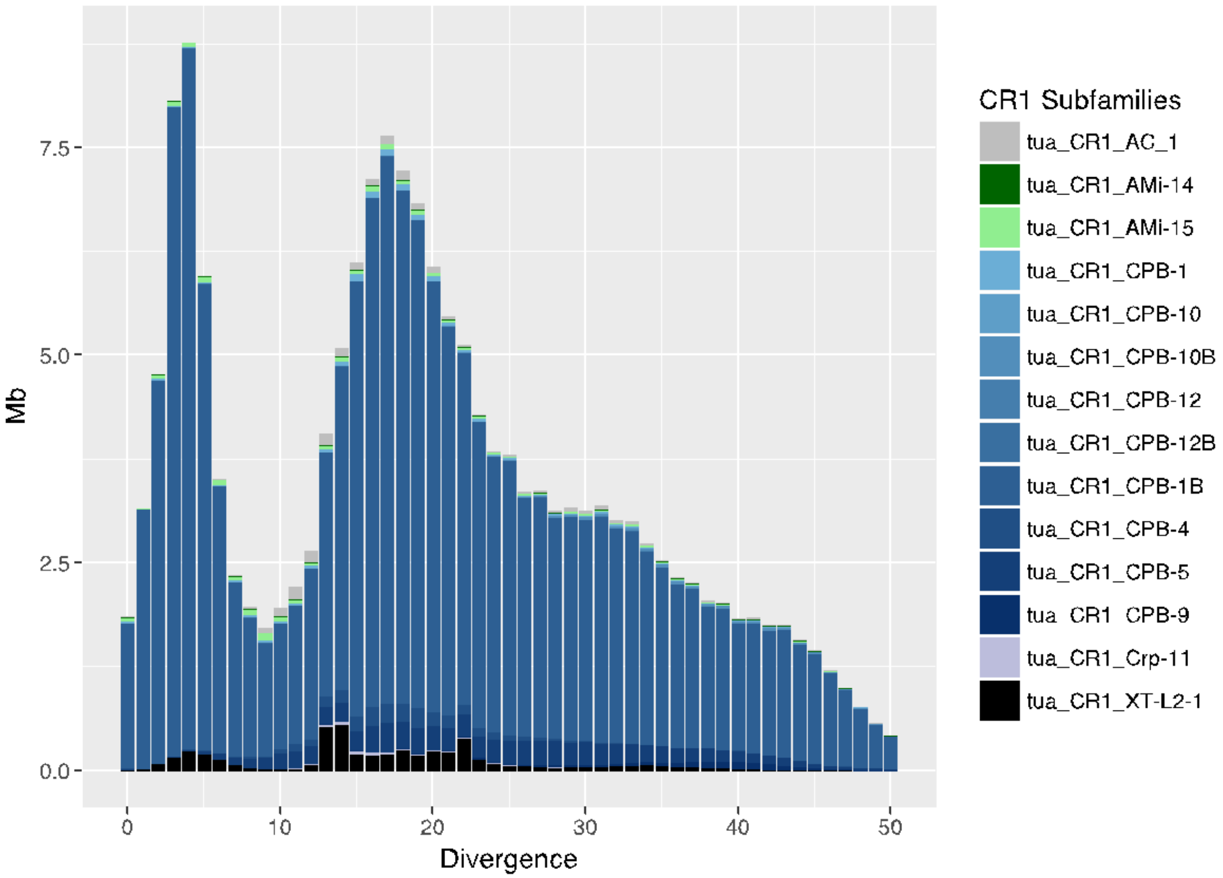

Supplementary Information Figure 4.10: CR1 retrotransposon landscape of the tuatara genome. CR1 subfamilies with a total length greater than 1,000 bp and with distances to consensus <50% are shown.

4.2.10 Unclassified (un-annotated) consensus sequences are probably segmental duplications

Since 216,591 of our *ab initio* consensus sequences were classified as unclassified repeats (unable to be annotated as transposable elements or gene families) and 33% of the tuatara genome was annotated using these unclassified repeats, we examined the characteristics of these un-annotated consensus sequences. Specifically, we looked at their length distribution and their copy number in the tuatara genome (Supplementary Information Figure 4.11). As a reference, we plotted the contig lengths of the tuatara genome. Supplementary Information Figure 4.11a shows that most of the tuatara contigs are shorter than 5 kb; the shortest contig is 800 bp and the longest 29,987,930 bp.

Supplementary Information Figure 4.11 also shows that 92% of unclassified repeat consensus sequences were shorter than 2 kb, and the longest unclassified consensus sequence was 31,536 bp. In terms of copy number, only 0.2% of the unclassified consensus sequences were present in more than 1,000 copies. Only 12 of them were present at more than 3,000 copies, they are around 1–8

kb in length. Because most of the unclassified consensus sequences are short and present at low copy number, they are most likely the product of segmental duplications.

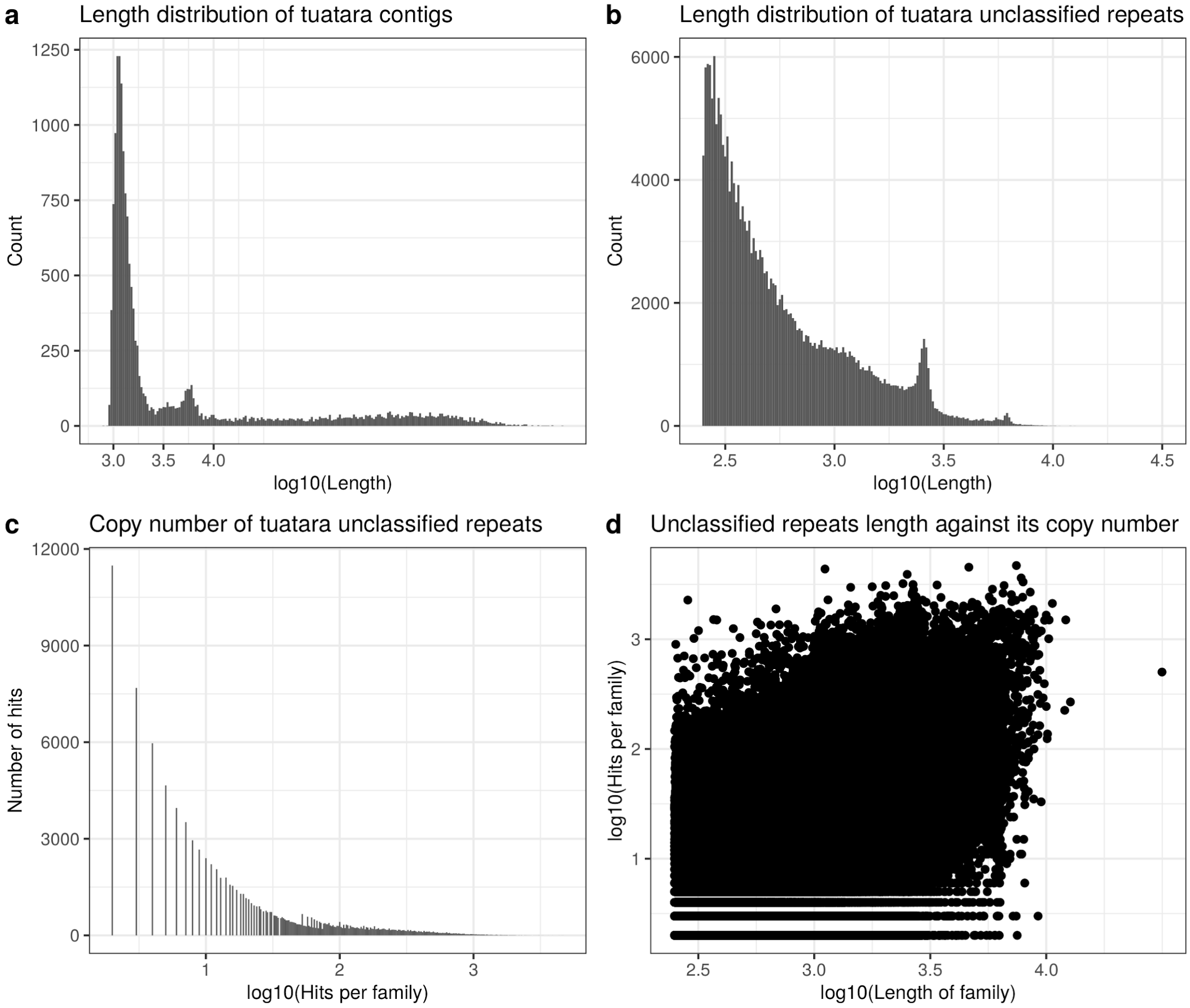

Supplementary Information Figure 4.11: Characteristics of unclassified consensus sequences in the tuatara genome. a) Length distribution of tuatara contigs, transformed length with log10; b) Length distribution of tuatara unclassified repeat consensus sequences generated from our *ab initio* method, transformed length with log10; c) Copy number distribution of unclassified repeat consensus sequences, transformed copy number with log10; d) Scatter plot of length and copy number of unclassified repeat consensus sequences.

In order to further analyse the 12 unclassified repeats with high copy number in the tuatara genome (>3,000), a coverage plot was used to visualise the copy number of genomic sequences similar to

the high copy consensus (Supplementary Information Figure 4.12). Supplementary Information Figure 4.12 clearly shows that for most of these unclassified repeat families, significant copy number enrichment was observed in a 300–1500 bp region (>2,000 copy number), while in family 206141 (7,798 bp) and family 051465 (7,944 bp), we observed multiple peak regions with copy numbers less than 700.

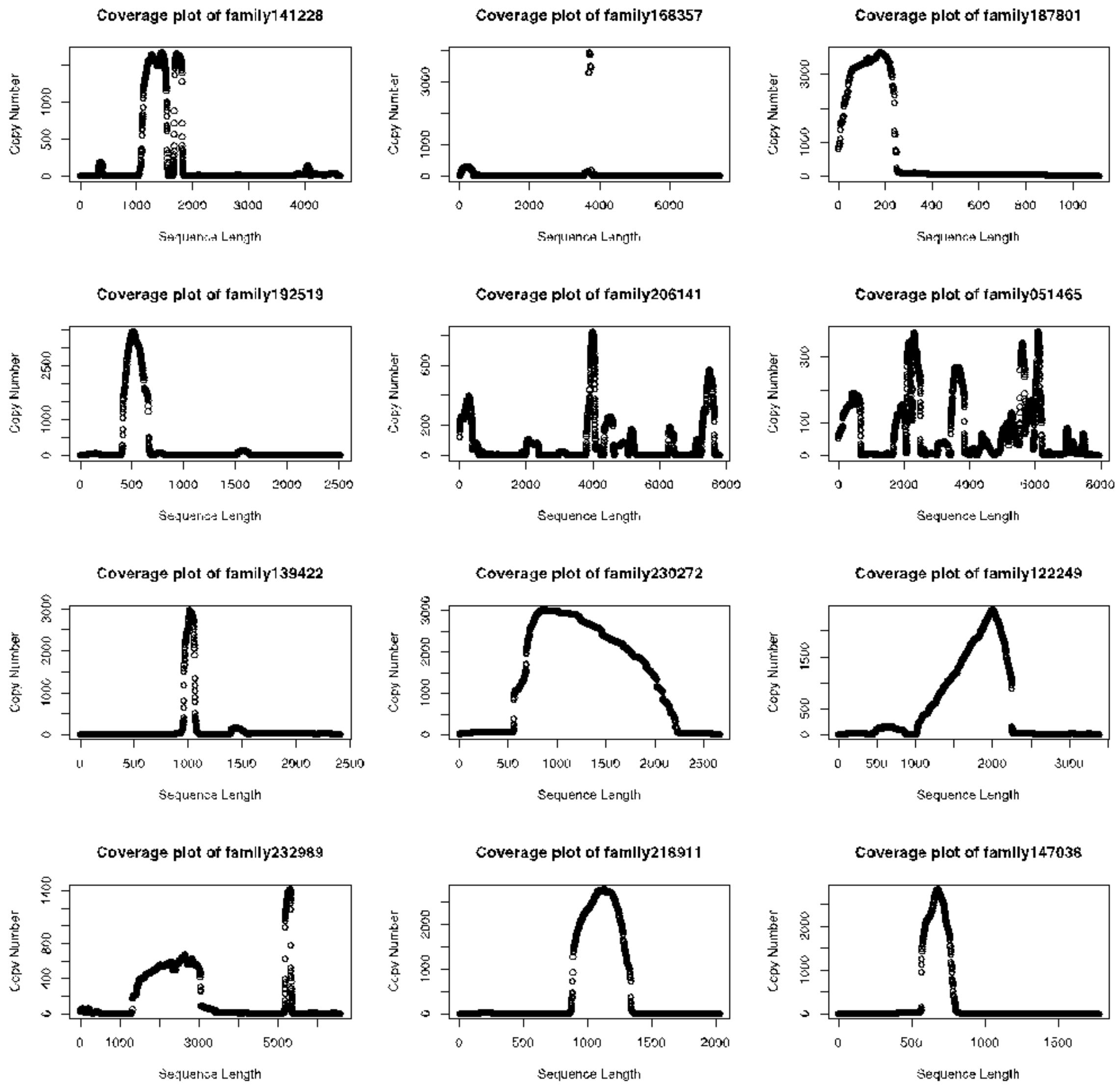

Supplementary Information Figure 4.12: Coverage plots of high copy number unclassified repeats in the tuatara genome. Overall coverage plots of 12 high copy number unclassified sequences.

Because the consensus sequences for these three families were obtained using single linkage clustering of pairwise alignments, the low baseline coverage level across the consensus indicates the approximate very low copy number of the genomic sequences used to construct the consensus.

The high copy number peaks indicate the presence of highly repetitive subsequences in the consensus.

CENSOR and BLASTN were used to further characterise the consensus sub-sequences from each peak. Supplementary Information Figure 4.13 contains outputs from both CENSOR and BLASTN that were used to annotate the peaks. In family 139422, CENSOR annotated it as a combination of different DNA transposons (Harbinger-N24 CPB, Harbinger-N33 CPB and Mariner-N5 PBa), which may indicate a potential novel DNA transposon. BLASTN annotation of the 3'end peak region was weakly similar to *Gavialis gangeticus* FAM129A non-coding regions. However, this annotation may result from a bias in the BLAST database, as there are only 2,660 tuatara entries in the NCBI nr database. In family 122249, CENSOR annotated the 5′end of the coverage peak as the 5′end of hAT-N92 DR, and the 3'end of the coverage peak as the 5'end of Gyspy116-LTR DR. However, we could not find any evidence of a promoter in these two peak regions. For the rest of the unclassified sequences, we observed what seemed to be a random combination of different repeat classes. As a final test to determine if any of the high copy sub-sequences might be SINEs, we compared all peak sub-sequences with manually curated tuatara SINEs (see Supplementary Materials 5 Repeat annotation: SINEs and DNA transposons) using BLASTN (-word_size=9, -outfmt=6), but did not detect any significant similarities.

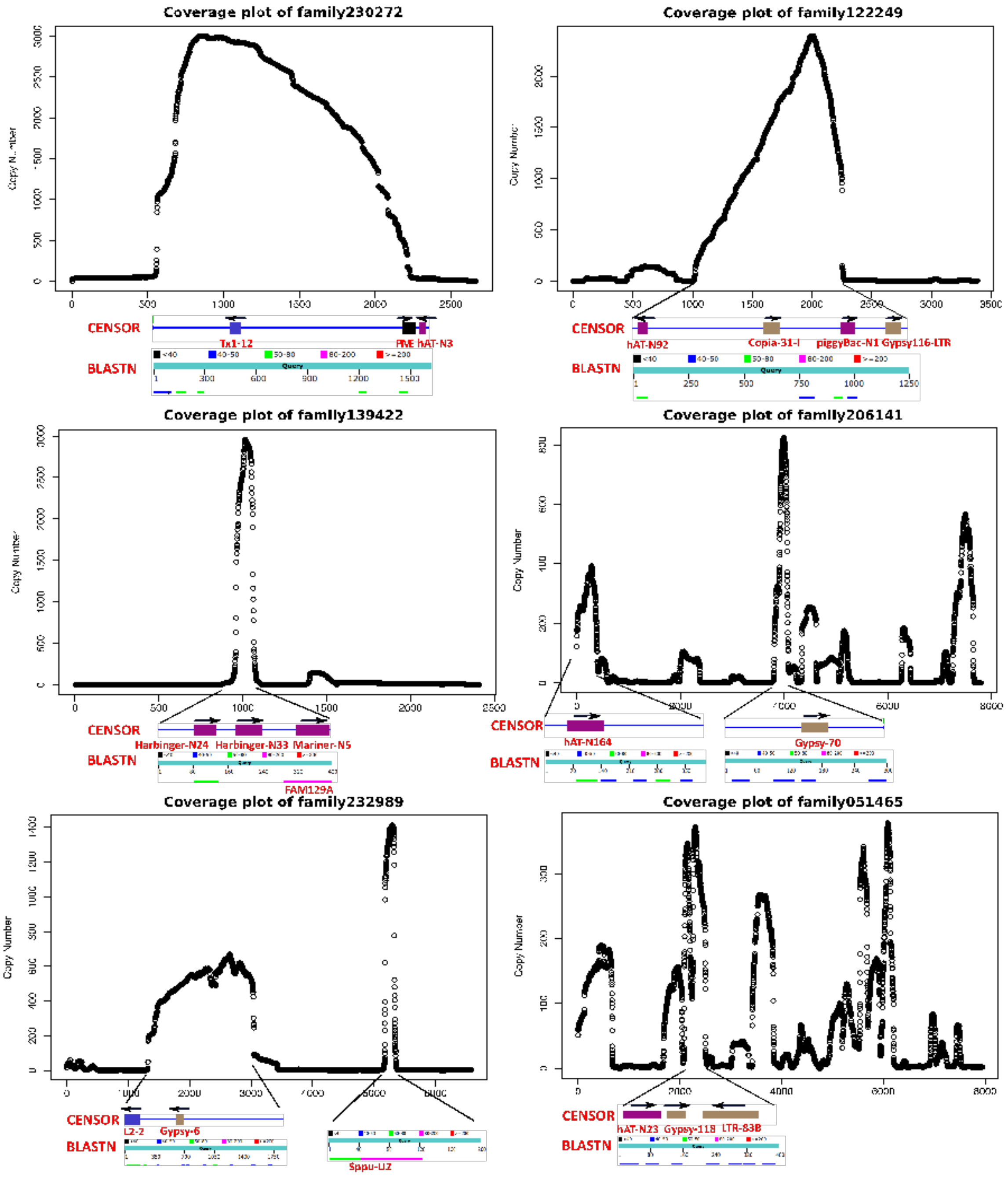

Supplementary Information Figure 4.13: Coverage plots of the 6 high copy number unclassified repeats in the tuatara genome. CENSOR and BLASTN annotation of the peak coverage region in unclassified family 230272, family 122249, family 139422, family 206141, family 232989 and family 051465. Black arrows represent the strand orientation of sequences based on CENSOR annotation.

In the case of unclassified family 141228 (4,531 bp), which has the highest copy number (5,497) (Supplementary Information Figure 4.14), a promoter region was found in the middle of the peak

with a score of 0.625 using the Promoter 2.0 (PolII promoter prediction tool). MEME (Multiple Em for Motif Elicitation) suite also detected a 40 bp motif around the promoter region (p-value: 1.30e-23). While both CENSOR and BLASTN show no significant hits against this peak.

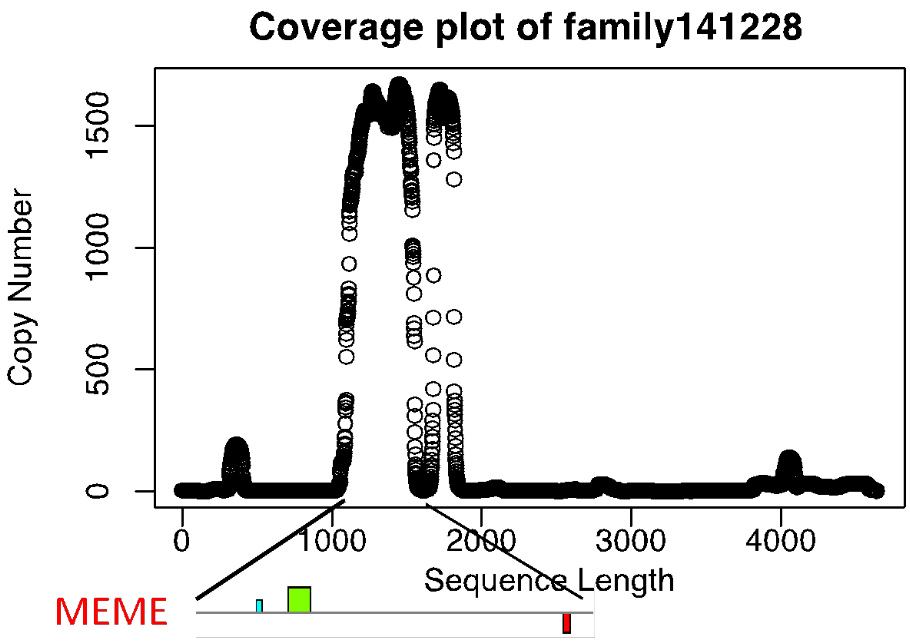

Supplementary Information Figure 4.14: Coverage plots of the highest copy number unclassified repeats in the tuatara genome. MEME annotation of the peak coverage regions in unclassified family 141228.

Draft genome assemblies from outbred individuals often contain uncollapsed haplotype blocks, so we examined the low copy number (copy number = 2) unknown repeats to see what proportion of these might be uncollapsed haplotypes instead of segmental duplications. We tested for such uncollapsed haplotypes by looking at the percent identity for alignments of the original sequence pairs identified in our initial pairwise alignments (Supplementary Information Figure 4.15).

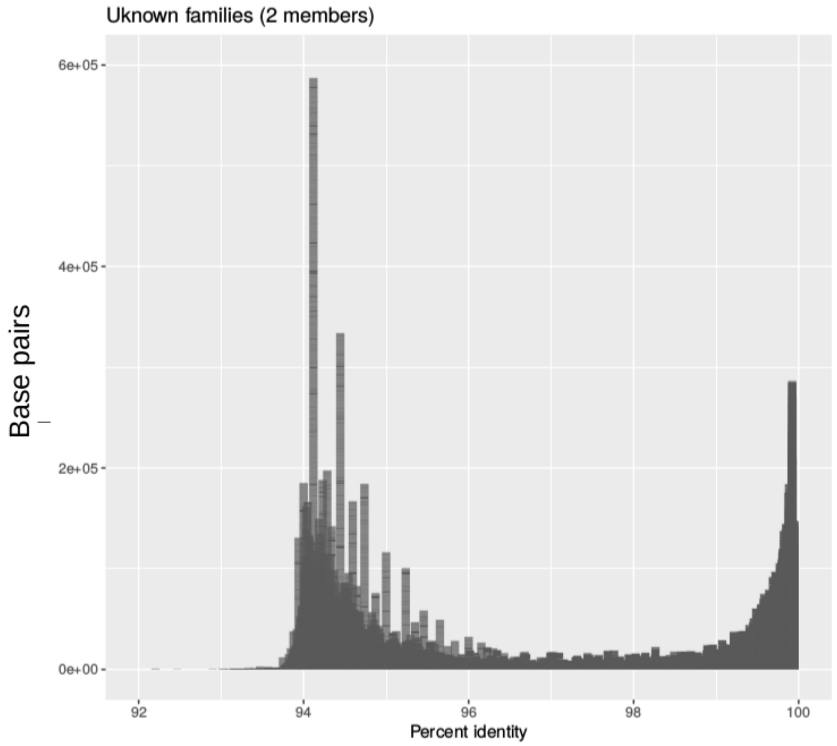

Supplementary Information Figure 4.15. Families with only two members were selected and the percent identity between the two members was calculated from the initial alignment output from Krishna.

There is a sharp peak of base pair coverage above 99.5% identity, suggesting the presence of polymorphic haplotype blocks in these repeats. Based on a typical polymorphism value of one SNP/kbp, we decided to filter out all two member families with a percent identity value of 99.9% or more. This excluded 14,104 families from our segmental duplication analysis and yielded a coverage value of 25.2% for segmental duplications (Supplementary Information Table 4.5).

Supplementary Information Table 4.5. Number of unknown families with 2 members and percent identity as a function of sequence similarity.

% Identity	Number of unknown families	Percentage of coverage from unknown families in the tuatara genome
>=99%	37,204	11.7%
>=99.5%	31,132	14.4%
>=99.9%	14,104	25.2%
>=100%	8,362	30.2%

4.3 Discussion

4.3.1 Segmental duplications in the tuatara genome

Compared to RepeatModeler, our method was able to classify more than one hundred times as many repetitive consensus sequences, with a broad length distribution, from 250 bp to 31,536 bp (Supplementary Information Table 4.5). Furthermore, our *ab initio* method allowed us to identify probable segmental duplications, as 99.8% of the unclassified repeat sequences from our method were present at fewer than 1,000 copies in the tuatara genome (Supplementary Information Figure 4.11). It is worth noting that because our method identifies similar sequences with low divergence that many of the presumptive segmental duplications we found are of relatively recent origin.

Segmental duplications (SD) are usually defined as being >1 kb long and >90%[72] identical and have proven to be relevant to disease[73] and integral to studies on genome evolution[74,75]. According to previous research, segmental duplication accounts for 2.1%[76], 4.9%[77], 3.7%[78] and 6.5%[79] respectively of the Chinese alligator, green anole lizard, chicken and zebra finch genomes. While we have not characterised segmental duplications using the commonly used parameters that identify recent duplications, we found that 6.7% of the tuatara genome is present as duplicated sequences >250 bp and >94% identical. This indicates that tuatara has a greater prevalence of SD compared to other vertebrates. Furthermore, after filtering out suspected uncollapsed haplotype blocks, the low copy number un-annotated duplications represented 25.2% of the tuatara genome based on CENSOR analysis, indicating the presence of many older SD. It is worthwhile noting that a comparable analysis of the anole lizard genome identified a total of 12% of the genome as

SD. The presence of so many SD in a vertebrate is unusual and may reflect differing drivers of genome expansion/turnover in tuatara (Supplementary Information Figure 4.11–14).

4.3.2 Significance of the LINE retrotransposons in the tuatara genome

The repeat content of reptiles and birds is lower than in mammals. The 31.3% repeat content observed in tuatara is within the bounds of previous studies where repetitive elements accounted for 23.4%[76], 27.2%[80], 10%[81], 30.4%[77], 8.5%[78] and 9.8%[79] of genome sequence in alligator, crocodile, western painted turtle, green anole lizard, chicken and zebra finch, respectively. While CR1 elements are the dominant LINEs in these genomes, L2 non-LTR LINEs are the dominant autonomous retrotransposon class in tuatara, accounting for 10% of the genome (Supplementary Information Table 4.1). L2 are regarded as ancient repeats and most individual L2 sequences tend to be non-autonomous and truncated[82]. Less than 3% of the human genome is annotated as L2 (L2 are molecular fossils in eutheria)[83], but in platypus, L2 elements are still active and they account for 19% of the genome[84]. Because our method identifies weakly divergent repetitive elements (newly inserted elements), and because most L2 elements we found in the tuatara genome could not be identified using the Repbase library, the L2 sequences we found are most likely tuatara-specific TE of recent origin and are probably still active (Supplementary Information Table 4.2).

Horizontal transfer (HT) is the non parent-to-offspring transmission of genetic material between individuals, a phenomenon primarily considered in a prokaryotic context. However, with assistance from a suitable vector (e.g. parasites, viruses), retrotransposons have the ability to jump between species as they do within a genome[85]. Relatively few studies have demonstrated HT of retrotransposons, including CR1s, RTEs and L1s[86–88], with no studies reporting HT for L2s. Our phylogenetic trees demonstrate that a particular class of tuatara L2 elements may have arisen from a recent transfer of platypus-like L2s into tuatara (Supplementary Information Figures 4.1, 4.3 and 4.4), while this same class of L2 is absent from other lepidosaurs (Supplementary Information Table 4.3 and Table 4.4).

According to the geographical distribution of fossils, monotremes are believed to have evolved in a region of Gondwanaland corresponding to Australia and Western New Guinea[89] and diverged from therian mammals about 163 to 186 million years ago[90]. In contrast, Rhynchocephalia diverged from Squamata about 220 million years ago[91] when *Sphenodon* had a very wide geographic distribution[92] before being restricted to New Zealand[93]. Because Gondwanaland broke apart around 215 to 175 million years ago, it is possible that monotremes and *Sphenodon* could have co-existed. Therefore, the platypus-like L2 elements found in tuatara are consistent with an HT event between a monotreme and sphenodon ancestor (Supplementary Information Figure 4.6).

In conclusion, while tuatara has a transposable element content similar to other reptiles it is dominated by L2 instead of CR1 LINEs and shows evidence of recent HT and expansion of platypus-like L2 elements. The CR1 elements in the tuatara genome also showed evidence of a recent expansion of CR1 similar to those found in alligator and turtle (Supplementary Information

Figures 4.7–4.10). Tuatara also has a far higher segmental duplication content compared to other vertebrate genomes. The unusual transposable element composition and segmental duplication prevalence in the tuatara genome may indicate that drivers of genome size and complexity in tuatara differ compared to mammals, birds and other reptiles.

4.4 Materials and methods

4.4.1 Reference genomes: repeat identification, annotation

4.4.1.1 *Ab initio* repeats identification and annotation

The tuatara draft genome assembly was used for repeat identification. All tuatara genome scaffolds were pairwise aligned using Krishna (https://github.com/biogo/examples/krishna)[94,95] with parameters set for 94% sequence identity (-dpid) and a minimum length (-dplen) of 250 bp. The resulting alignment intervals were then used as input for igor[94,95] to define families of repeat sequences using the default parameters. Igor output was used as input for seqer[94,95] in order to generate repeat consensus sequences for each cluster/family based on MUSCLE (v3.8.31) alignments[70]. Only family members within 95% of the length of the longest family member were aligned and to avoid consensus sequence expansion due to indels in the global alignment, a maximum of 100 randomly chosen sequences/family were included in the alignment.

Identifiable repeat consensus sequences were annotated using CENSOR[96] with the Repbase ‘Vertebrate’ library (downloaded on 1st March, 2016, includes 41,908 sequences). Further annotation of consensus sequences was based on WU-BLAST alignment against a comprehensive retroviral and retrotransposon protein database assembled from the National Centre for Biotechnology Information[97], and against swissprot to identify known protein-coding genes from large gene families inappropriately included in the repeat set. Consensus sequences identified as either simple sequence repeats (SSRs) or protein-coding sequences, but not similar to retrotransposon or endogenous retrovirus protein-coding sequences, were removed from the consensus set. A total of 3,585 consensus sets were removed, including 3,550 that were identified as protein-coding sequences, and 35 that were identified as SSRs. After annotation of these tuatara repeat consensus sequences, CENSOR was used to map these sequences back to the tuatara genome in combination with the Repbase ‘Vertebrate’ library. Supplementary Information Table 4.6 shows the comparison of repeat libraries generated using RepeatModeler[47] and our *ab initio* method[48].

Supplementary Information Table 4.6: Summary of *Sphenodon punctatus* repeat library metrics

	RepeatModeler Library	*Ab initio* Library
No. of consensus sequences	2,322	232,111
Total length (MB)	1.3	171
Well-annotated Consensus sequences	901	15,520
Unclassified Consensus sequences	1,421	216,591
Min./Max. Length (bp)	36/6,350	250/31,536

4.4.2 Analysis of LINE elements in the tuatara genome

In order to investigate the similarity of tuatara L2 and CR1 sequences generated from our *ab initi*o method and RepeatModeler (http://www.repeatmasker.org/RepeatModeler.html), we extracted long L2 and CR1 consensus sequences (2–4 kb for L2, 2.5–4 kb for CR1) from both libraries. MUSCLE was used to carry out global alignments between the two repeat sequence sets. FastTree (v2.1.8)[98] was used to infer a maximum likelihood phylogeny from the alignment output. FigTree (v1.4.2)[99] was used to visualise and annotate the tree using repeat class labels.

4.4.3 Resolving L2 classification

Many of the tuatara L2 consensus sequences were annotated as being most similar to platypus L2. This introduced an annotation problem because, according to Repbase, almost all platypus L2 consensus sequences were annotated as belonging to the CR1 clade, rather than L2 clade. In order to resolve the annotation, we needed to determine which clade platypus L2 really belonged to. Full-length consensus sequences of chicken CR1 elements were extracted from Repbase, and MUSCLE was then used to carry out global alignments between CR1 elements and the 2–4 kb long L2 consensus sequences from tuatara. Alignment output was used to construct a maximum likelihood phylogeny using FastTree. FigTree was used to visualise and annotate the tree using repeat class labels.

4.4.4 Dendrogram construction from LINE nucleotide sequence alignments

In order to determine the evolutionary position of tuatara L2 and CR1 within vertebrates, L2 and CR1 consensus sequences between 2–4 kb long from tuatara identified using our *ab initio* method and tuatara L2 sequences that ranged from 2–4 kb long identified using RMD were extracted. L2 and CR1 consensus sequences (2–4 kb) were also extracted from the Repbase 'Vertebrates' library. We globally aligned the resulting 222 L2 consensus sequences and 231 CR1 consensus sequences individually using MUSCLE. FastTree was used to infer a maximum likelihood phylogeny from

the global alignments. Archaeopteryx v0.9901 beta was used to visualise and annotate the trees using repeat class labels.

4.4.5 Phylogenetic analysis of L2 elements using RT domain sequences

All L2 and CR1 sequences were extracted from our *ab initio* consensus sequence libraries, and anole, turtle and crocodile L2 consensus sequences were extracted from the Repbase library. USEARCH[100] was then used to scan for open reading frames in L2 consensus sequences that were at least 60% of the expected length (>1.5 kb for ORF2p). After translation, ORF2p candidates were checked for similarity to known domains using HMM-HMM comparison[101] against the Pfam28.0 database[102] as of May 2015 (includes 16,230 families). ORF2p containing RT domains were extracted using the envelope coordinates from the HMMer domain hits table (–domtblout), with a minimum length of 200 amino acids. Nucleotide sequences that contained RT domains were extracted and assembled into one file (a total of 48 sequences).

Two methods were tested to describe the evolutionary dynamics of potentially active L2 elements. First, 48 RT domain nucleotide sequences within ORF2p were aligned with muscle, and then FastTree was used to infer a maximum likelihood phylogeny (-nt, -gtr). Another RT domain Phylogeny tree was inferred by using MrBayes (lse nst=6; mcmc ngen=30000 samplefreq=200 printfreq=200 diagnfreq=1000)[103] from MUSCLE alignment output. Both of the methods used a GTR model and FigTree was used to visualise and annotate the tree using repeat class labels.

4.4.6 Potential horizontal transfer of L2 elements between tuatara and platypus

CENSOR was used with a custom library of platypus-like and non-platypus-like L2 consensus sequences from tuatara to find similar sequences (both full length and fragments) in five reptile genomes (anole, crocodile, alligator and bearded dragon) and one monotreme genome (platypus). To confirm the validity of hits, each hit was extracted as a nucleotide sequence and aligned with BLASTN (default parameter) against the platypus-like tuatara L2 and non-platypus-like tuatara L2 consensus sequences. Hits smaller than 50 bp were discarded.

MUSCLE and PILER were used to build two super consensus sequences, one was generated from platypus-like tuatara L2, the other one was generated from non-platypus-like tuatara L2. RepeatMasker was used to align each super consensus against their corresponding tuatara L2 elements, in order to calculate divergence rate using Kimura 2-parameter divergence metric, adjusted for ‘GC’ content.

4.4.7 Divergence rate of CR1 elements in the tuatara genome

In order to plot the relative age profiles of CR1 insertions, tuatara CR1 consensus sequences identified using CARP (3,596 consensus sequences) were aligned and annotated against the tuatara genome by using RepeatMasker version open-4.0 (2) (-pa 32 -a -nolow -xsmall -gccalc -html -excln). Kimura distances were calculated using RepeatLandscape from the RepeatMasker

alignment outfiles (-noCpG). Kimura distances are computed based on the rates of transitions and transversions between the tuatara genomic sequences and the CR1 consensus sequence, allowing estimation of the relative ages of inserted CR1 copies.

4.4.8 Identification of novel repeat sequences from the tuatara genome

In order to explore the unclassified consensus sequences from our *ab initio* method, 216,591 unclassified sequences were extracted, and the R package ggplot2[104] was used to visualise their length distribution against copy number.

For high copy number (3,000 copies) families, a coverage plot was used to investigate the positional distribution of genomic sequence fragments with respect to these unclassified sequences. BLASTN and CENSOR were further used to characterise the consensus sub-sequences from the coverage peaks of 12 unclassified sequences found in the coverage plots. We scanned for potential promoters present in coverage peaks using an RNA PolII promoter prediction tool (Promoter 2.0)[105]. In addition, we attempted to find any evidence of a promoter in peak coverage regions using MEME (Multiple Em for Motif Elicitation) suite[106].

The criteria we used to identify novel repeats from unclassified repeat consensus sequences were as follows: the sequence must have copy number greater than 10,000; the sequence should be of a reasonable length for a repeat (200 bp–6 kb); the sequence cannot be identified as a repeat based on similarity to the Repbase library, and it should not be similar to any protein coding sequences (the sequences would then likely to belong to a gene family, or be part of a conserved domain).

4.4.9 Problems with RMD derived consensus sequences

When we looked at the repeat consensus sequences generated from the RMD method, we noticed that 14 out of 1,149 sequences had long stretches of polyN, that parts of these sequences were annotated as unknown sequences, and parts of these sequences were annotated as transposable elements. One example is sequence rnd-3 family-287#LINE/L2 (~3,645 bp), which would appear to be present at high copy number (~16,000) in the tuatara genome.

```
>rnd-3_family-287#LINE/L2 (Recon Family Size = 42, Final Multiple Alignment Size = 40)

NTGGTGAGGTTGCAGCTCGTCTGCATACNNTCCCAGCNNCCTCGGCTGTCATCGCCCGAGGACGCCGCGACCCTCCTGGGGC
TTGGCCTCCCTGCCNCCCCAAGCTCCACCGACCAGCGGGCCGAAAAGCGCGNGCCNAAAGGCGCGCGCCTTANAGGCGCNC
GCCTCACANNTACTTTTCTNCTGTACTTCCCCTCTNCTGTACTTNCCCACTAACNTGTCTNNCCCACTAACATGTCTCGCCCTNT
CTTTCCCNCCTCTCCTTCCCCTCCTCTCCTTCTCCTCTTNCCTCCATCTTTTTCCTCCCCTNCTTTCTCCTCCCAGCCTNTTCCAC
CCTCTAACCTCCCCCTCTCTGCCTCCCATACATCCTTTGCTCCCTTGGCTACTTTCTCTCGTTGCTCCCCTTCCGCTCCTGTCTC
TAGCCTTCCTCGACGCTCCCCTTCTCCCAATCTTATCCCTATTCCTCCTGTCTCTCCNGCCGACCCCGTCGCCCTCCTTCCCCT
AATCTTATCCCCATTNCTTGCCTCCCCCCTCATCAACTNCCATTCTCCTGCTCCCTCTGGAACTGTCGCTCNTCTGCCAGTAAAG
CCCCCTATANCCATGANCTTTTCCTCTCTCGCTCCCTCCANCNCCTCGCNCTTACTGAAACCTGGGTCACCCCCTCNNNNNNNN
NNNNNNNNNNNNNNNNNNNNNNNNNNNNNNNNNNNNNNNNNNNNNNNNNNNNNNNNNNNNNNNNNNNNNNNNNNNNNNNNNN
NNNNNNNNNNNNNNNNNNNNNNNNNNNNNNNNNNNNNNNNNNNNNNNNNNNNNNNNNNNNNNNNNNNNNNNNNNNNNNNNNN
NNNNNNNNNNNNNNNNNNNNNNNNNNNNNNNNNNNNNNNNNNNNNNNNNNNNNNNNNNNNNNNNNNNNNNNNNNNNNNNNNN
```

NNNNNNNNNNNNNNNNNNNNNNNNNNNNNNNNNNNNNNNNNNNNNNNNNNNNNNNNNNNNNNNNNNNNNNNNNNNNNNNNN
NNNNNNNNNNNNNNNNNNNNNNNNNNNNNNNNNNNNNNNNNNNNNNNNNNNNNNNNNNNNNNNNNNNNNNNNNNNNNNNNN
NNNNNNNNNNNNNNNNNNNNNNNNNNNNNNNNNNNNNNNNNNNNNNNNNNNNNNNNNNNNNNNNNNNNNNNNNNNNNNNNN
NNNNNNNNNNNNNNNNNNNNNNNNNNNNNNNNNNNNNNNNNNNNNNNNNNNNNNNNNNNNNNNNNNNNNNNNNNNNNNNNN
NNNNNNNNNNNNNNNNNNNNNNNNNNNNNNNNNNNNNNNNNNNNNNNNNNNNNNNNNNNNNNNNNNNNNNNNNNNNNNNNN
NNNNNNNNNNNNNNNNNNNNNNNNNNNNNNNNNNNNNNNNNNNNNNNNNNNNNNNNNNNNNNNNNNNNNNNNNNNNNNNNN
NNNNNNNNNNNNNNNNNNNNNNNNNNNNNNNNNNNNNNNNNNNNNNNNNNNNNNNNNNNNNNNNNNNNNNNNNNNNNNNNN
NNNNNNNNNNNNNNNNNNNNNNNNNNNNNNNNNNNNNNNNNNNNNNNNNNNNNNNNNNNNNNNNNNNNNNNNNNNNNNNNN
NNNNNNNNNNNNNNNNNNNNNNNNNNNNNNNNNNNNNNNNNNNNNNNNNNNNNNNNNNNNNNNNNNNNNNNNNNNNNNNNN
NNNNNNNNNNNNNNNNNNNNNNNNNNNNNNNNNNNNNNNNNNNNNNNNNNNNNNNNNNNNNNNNNNNNNNNNNNNNNNNNN
NNNNNNNNNNNNNNNNNNNNNNNNNNNNNNNNNNNNNNNNNNNNNNNNNNNNNNNNNNNNNNNNNNNNNNNNNNNNNNNTG
AGGAAATCTCCTCTCTGCTCTCCTCCTCCAACCCTTCCACTTGCNCCCTTGATCCCATTCCTTCCCGCCTCCTCACCTCCATTAC
TCCCACTGTCGCCCCTTCCTTCCTGCACATCATCAACCTCTCCCTCTCTACTGGCTCTTTCCCTTCTGCCTTCAAACATGCCACA
GTCTCTCCTATTCTCAAAAAGTCCACTCTTGACCCCTCTGACCCTTCTAGCTATCGTCCTGTCTCACTCCTTCCTTTTGCTTCCAA
GCTGCTTGAACGTGTTGTCTACTCCCGCTGCCTTGACTTCCTATCCTCCAACTCCTCCCTTGACCCCCTCCAATCTGGCTTCCG
CCCCCTTCATTCTACTGAAACTGCTCTCGCTAAGATCACCAACGATCTTCTGGTGGCTAAATCTAAACGTCTCTATTCCATTCTTG
TCCTCCTTGATCTCTCTGCTGCCTTCGATACTGTTGACCACTCACTCTTGCTGGATTCCCTTCACTCCCTTGGCTTTTGTGGCTC
TGTCCATAACTGGTTCTCCTCCTACCTCTCTGACCGCTCCTTTAATGTGTCTCTCTCCAACTCCGCCTCCTCCTCCTTCCCCCTC
TCAGTTGGTGTTCCTCAAGGCTCTGTTCTTGGCCCTCTCCTCTTCTCGATCTATACCTCATCCTTAGGCAAACTCATTGCCTCCC
ATGGCTTCCAATACCACCTCTATGCTGATGACACTCAACTCTATCTCTCTACTCCCTCTCTCTCTCCCTCTGTTCAAGCTCGTCTC
ATCAACTGTCTCTCTGACATCTCTACCTGGATGTCTCAACGCCAACTCAAACTCAACATGGCTAAAACTGAACTCCTTATCTTCC
CCCCTCATCCTTCCTCTCCCTCCTCACTGTCAATAACTATTGATGGAGTCACCATCCTGCCTGTCCCCCAAGCCCGTAGCCTAG
GCTTCATCTTTGACTCACCCCTTTCTTTTAAACCCTACATTGACTCTGTTGCTAAATCCTGTCGCTTCTCCCTCCATAACATTGCT
AAGATTCGCCCTTTTCTCTCTGTCTCGTCTGCCAAAACTCTTGTTCATGCTCTGGTCATCTCTCGCCTCGATTACTGTAACCTCCT
TCTCACTGGCCTTCCCCGGTCTCACCTTTCTCCCCTCATCTCTGTCCAAAACTCTGCTGCCAGGGTCATCCACCTCGCCNGTCG
CTTCGACCATGTGCAGCCCCTCCTCTCTTCGCTTCACTGGCTCCCCCTTCCTGAACGAATCCTGCACAAGCTCCTAGTCTTCAC
CTATAAAGCTCTTCACAACCTCGCTCCCCCCTACCTCTCTGCGCTTATCGCCCTCCGTACTCCAGCCCGTGCACTTCGCTCCTC
CTCTGTCCCCTCTCTCGCCCTTCCACGCCTTCCCTGCTCCCCTAAGCGCCTTCGTCCCTTCTCCCTCGCTGCCCCCCATGCCTG
GAACTCCCTCCCTGTCCACATCCGCCTTGCCCCCTCCCTTCGTACCTTCAAATCCCTCCTCAAAACCCACCTCTTCCGCTCTGC
TCTGTCCGACTCTTTATCCTGCTGAGCCACTATTCCTATACCTACGATCCGTTTACCCTTATCCTTGCAACATGGACCTGCCTGC
TGTCATTCTCATCGTAACCAAGCCAAGTACGTGCACCTGAGACCTCGCATCTTCATGAATGTTAGACCCCTTGTCTCTCCCTCTG
TTCCCCTTCCTNNGTCTCTCCCCCAGCTCCTGTCTCTCAGATTGTAAGCCCTAGGGGCAGGGACCGTGTCTATTTTATTACTTTG
TAAAGCGCCAGGTACATTGCTGGCGCTATATAAATGCTTGTTAATAATAATAATAA

However, when we annotated RMD consensus sequences using CENSOR and the Repbase library, we found some of these sequences had not been accurately annotated using RepeatMasker/RepeatModeler. Supplementary Information Figure 4.15 below shows an example. tua2-115#DNA/hAT-AC is annotated with two different repeat classes.

Name	From	To	Name	From	To	Class	Dir	Sim	Pos/Mm:Ts	Score
rnd-1_family-240#DNA/PIF-Harbinger	292	418	DNA9-53_OS	91	223	DNA	c	0.6850	1.7368	221
rnd-1_family-240#DNA/PIF-Harbinger	1081	1282	BEL-636_AA-I	1357	1567	LTR/BEL	c	0.7310	2.7143	414
rnd-1_family-240#DNA/PIF-Harbinger	1431	1620	Copia-103_AA-I	1990	2183	LTR/Copia	d	0.7037	2.4211	400

Masked Sequence

```
>rnd-1_family-240#DNA/PIF-Harbinger
ACATCANGGAGGGAGCAGGCACCGCCCGCGGCAGCAGGGCGGAGGCGGGAGCCCACAGCGGGGCCAGCAN
CCGGGGCNCGGAAGGCACAGGGGGNCTCACAGCCAGCTGCAGGGGGCCACGAAGGGGCCCGGGGCCGGGT
GGCCCGCCAGGTGAACGAAGTGGGGCCANTGAAGCATGCCCCGGCGGTACCCCAGCACGGACATGTGGTG
GGCGACCAGGTAGTANGCCTCGAAGGGATCCTGCACGGCGAGCCCCANCTGCACATCGGAACGGGACCAC
ACACCNGACAGXXXXXXXXXXXXXXXXXXXXXXXXXXXXXXXXXXXXXXXXXXXXXXXXXXXXXXXXXXX
XXXXXXXXXXXXXXXXXXXXXXXXXXXXXXXXXXXXXXXXXXXXXXXXXXXXXXXXXXXXXXXXXXXXTG
AACGCAGCCAAAGTAGGACACGGCCAGGGCAGGGCCCCCCTCCGCCAGACAGGCCGTGTAGGCGGCCAGG
CTGCACACAGACAGGGCAGACACGGTCTCCGCCGCGGACCACGCCACATGCGAGCACCTCATCTCCGGGA
TGCGCCCATCGGGAAAGGGANNCAGGCCCAGCGTCTCCGCATGCAGCCGGCAATCGCGGGCGCTCGCCTC
ATAGCCAAAGTAGCGNAGNCCGGAGCTCAGGCTCGCATAGGCACCCGACAGGCTGTCCGGGCAGCTCAGG
AGCAATTGCACGGCCGGGGAATGCCACAGGTCACACAGGGCCATGGTCTCGCGGAAGGTCCAGCTATGGG
GGGCCCCATGGGGGCCGGGAGCCCNGNGGAAGCGCAGCCAGCCATGGTCCAGCAAGCGANGGAGACTAAC
NGCAAGGACAGGGGGGCCCCACAATCCCTGGAGGGACGGGAGGGGANGCAGGGGGGCCCCAAGGCCAGGC
GATAAGGCCACAGGCCGCAACATNGCACGGCAAACGAGCGAGGCGTGGGGTGCGGACCCAGCACAGCTGC
NGGAACNGAGCGCACAGACAGGACAGCCTACACACTAATCAGGGCGGCGAGGACAACAGGCAGCCAACAC
NGAGAGGTGGAGGTGATGACAGCAGGAATXXXXXXXXXXXXXXXXXXXXXXXXXXXXXXXXXXXXXXXXX
XXXXXXXXXXXXXXXXXXXXXXXXXXXXXXXXXXXXXXXXXXXXXXXXXXXXXXXXXXXXXXXXXXXXXX
XXXXXXXXXXXXXXXXXXXXXXXXXXXXXXXXXXXXXXXXXXXXXXXXXXXXXXXXXXXXXXXXXXXXXX
XXXXXXXXXXXXXXXXXXXXXXXACACGNGCCCCAGCCTATAACGGGTGCGGCCGCACCCCACCCGGGCA
CCGNTCACCATGGAGCTGGGNCGGTTGGGCGCCTGCGGGATGCACCTAGCCGTCTGCGCGGAGTGGTACC
GCTCGCACACGGACCTCCTGCGCATCCAGAXXXXXXXXXXXXXXXXXXXXXXXXXXXXXXXXXXXXXXXX
XXXXXXXXXXXXXXXXXXXXXXXXXXXXXXXXXXXXXXXXXXXXXXXXXXXXXXXXXXXXXXXXXXXXXX
XXXXXXXXXXXXXXXXXXXXXXXXXXXXXXXXXXXXXXXXXXXXXXXXXXXXXXXXXXXXXXXXXXXXXX
XXXXXXXXXXGCTCCAGTGGCGGAGGGAGACCATGGGGCGCGCTGCCCTGCTCGCTATGCCCATTGNCCG
GCGGGTATGGGTCACGGAGCGGGCACAGGGGTGGTGGGCCAGGGCGCTGGCTCAGTGGGACGAGGCCGCC
TGGAGGGCGCACTTTCGGATGGACAGGCGCACCCTNCTGCGACTCGCGGAGCTGTTAACGCCCTGGCTGG
AGCGGCAGCACACCCCCATGCGGACCCCCATNCCCATGGTCCAGCGGGTGGCC
```

Supplementary Information Figure 4.16: Example of bad annotation of consensus sequence by RMD (tua2-115#DNA/hAT-AC).

5 REPEAT ANNOTATION: SINES AND DNA TRANSPOSONS

Valentina Peona, Claire R. Peart, Vera M. Warmuth, Alexander Suh*

*Correspondence to: alexander.suh@ebc.uu.se

5.1 Methods

Raw consensus sequences of SINE retrotransposons and DNA transposons were initially predicted *de novo* by RepeatModeler (version 1.0.8[47]). First, the names of all raw consensus sequences were shortened to ease downstream analyses (e.g., "rnd-1_family-1#Unknown" was renamed into "tua1-1#Unknown"). Subsequently, consensus sequences classified as SINE retrotransposons (or with sequence similarity to SINEs or small RNAs; see *Supplementary Materials 7 Tuatara genome: non-coding RNA annotation and analysis* for further details on the latter) and DNA transposons were subject to manual curation using standard procedures[107,108]. Briefly, each consensus sequence was queried against the entire tuatara draft assembly using BLASTn (version 2.2.28+[69]), and up to 20 of the best hits were extracted with 1 kb flanks and automatically aligned to each consensus using MAFFT (version 6[109]). From these alignments, majority-rule consensus sequences were generated, carefully inspected by eye, and considered 'complete' if their 5'- and 3'-termini were flanked by a unique, single-copy sequence in the alignments. The information on the termini was further used to manually estimate the size of target site duplications and to reclassify consensus sequences into TE families. This yielded manually curated consensus sequences as a proxy for each novel subfamily of 64 DNA transposons (Supplementary Information Table 5.1) and 18 SINE retrotransposons (Supplementary Information Table 5.2). These were grouped into families of consensus sequences (subfamilies) with sequence similarities to each other. Following the nomenclature of similar efforts in birds[110], subfamilies with sequence similarity to vertebrate repeats from Repbase[111] in CENSOR (http://www.girinst.org/censor/index.php) were named according to the name of the best hit + suffix "_tua", with the exception of MIR SINEs which were named according to their phylogeny (see below). Subfamilies with only a partial hit in CENSOR were named with the suffix "-L_tua" ("L" indicating "like"), and subfamilies with no hit in CENSOR (and newly defined tuatara MIR families, see below) were defined as novel repeat families indicated by the name prefix "tua" before the TE superfamily name (e.g., tuaMIR-1a and tuaDNA1). Subsequently, a custom library of these classified tuatara SINE and DNA subfamilies, combined with all repeat subfamilies from birds, crocodilians, turtles, and squamates available in Repbase, was used to annotate SINEs and DNA transposons in the tuatara genome assembly via RepeatMasker (version 4.0.7[112]). Final downstream analyses comprised the generation of TE landscapes (Supplementary Information Figures 5.1–4) and a phylogenetic tree of MIR SINEs (Supplementary Information Figure 5.5). Both analyses followed procedures described in detail elsewhere[113]. Briefly, using the

RepeatMasker ".align" output file, the Kimura 2-parameter distance between each repeat copy and its consensus sequence was calculated using the calcDivergenceFromAlign.pl script from the RepeatMasker program package under exclusion of hypermutable CpG sites. TE landscape plots were then generated using the total amount of annotated sequence per TE consensus in 1%-bins of divergence (https://github.com/ValentinaBoP/TuataraTELandscapes/). Furthermore, tuatara MIR SINEs were aligned with all MIR elements available in Repbase using MAFFT (E-INS-i, version 7). After manual re-alignment, maximum likelihood sequence analysis was performed in RAxML (8.0.0[114], GTRCAT model, 1,000 bootstrap replicates) on the CIPRES Science Gateway[115]. The resulting phylogenetic tree topology was used to define tuatara MIR families (Supplementary Information Figure 5.3). All SINE and DNA transposon consensus sequences are available in Data 5.1.

5.2 Results and discussion: DNA transposons

Our manual curation of DNA elements yielded 64 novel subfamilies that are present in the tuatara genome (Supplementary Information Table 5.1). These include 24 families (groups of subfamilies with sequence similarity to each other) which have no sequence similarity to previously known transposable elements in Repbase[111] and were thus named tuaDNA* to imply that they are tuatara-specific families. Although most of these novel families have target site duplications with 8 bp as typical for the hAT superfamily[116], most elements were non-autonomous, i.e., lacking the transposase open reading frame needed for further classification. We thus conservatively classified these families as DNA elements of an unknown superfamily (Supplementary Information Table 5.1). RepeatMasker analysis of the tuatara genome assembly detected copies of a total of 492 DNA transposon subfamilies and the relative age distributions of the most common ones are shown as a DNA transposon landscape (Supplementary Information Figure 5.1, 5.2). At least 30 of these DNA transposon subfamilies were recently active, as each of them has copies totalling over 0.01 Mb with distances to consensus $\leq$ 5% (Supplementary Information Figure 5.1a). Notably, these belong to a diverse range of DNA transposon superfamilies, namely Charlie, Harbinger, hAT, Mariner, piggyBac, and Polinton/Maverick (Supplementary Information Figure 5.1a). There even seems to be potentially ongoing DNA transposon activity, as suggested by Harbinger, hAT, piggyBac, and tuaDNA families with dozens or hundreds of copies with 0% distance to consensus (Supplementary Information Figure 5.1b). The tuatara genome is thus not only characterized by a very high diversity of ancient and lineage-specific DNA transposon families (Supplementary Information Figure 5.2), but also a high diversity of recently active families, which is unique among amniotes.

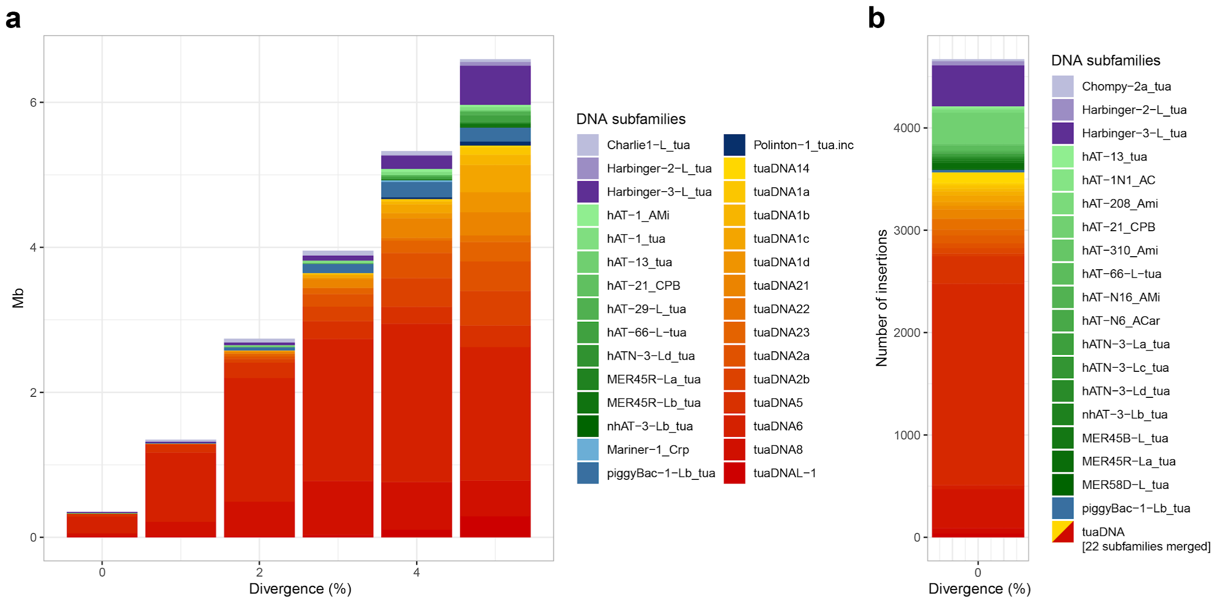

Supplementary Information Figure 5.1: Recent DNA transposon landscape of the tuatara genome. (**a**) Only DNA subfamilies that occupy more than 0.01 Mb with distances to consensus ≤ 5% are shown. (**b**) Subfamily composition of potentially active DNA transposons, i.e., copies/fragments with 0% divergence to consensus. Only the 41 DNA subfamilies with at least 10 copies in this divergence bin are shown.

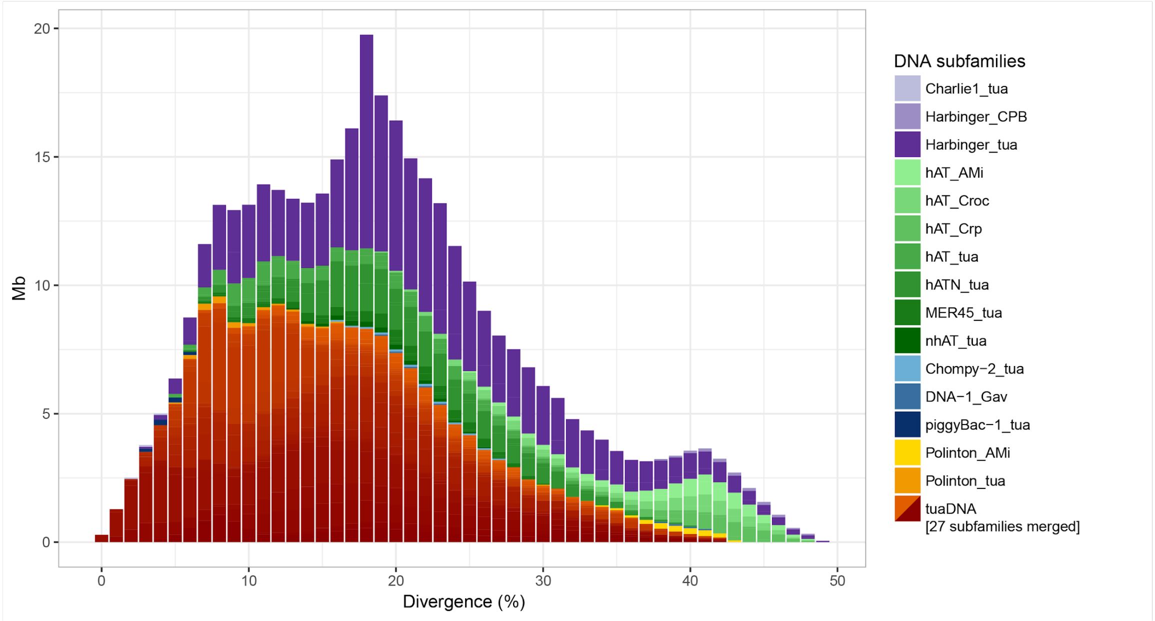

Supplementary Information Figure 5.2: Landscape plot of all DNA transposons. Including 24 novel tuatara DNA transposon families (27 subfamilies). Only DNA subfamilies that occupy more than 0.1 Mb are shown.

5.3 Results and discussion: SINE retrotransposons

Our manual curation of SINE elements yielded 18 novel subfamilies that are present in the tuatara genome (Supplementary Information Table 5.2). Of these, 15 have L2-like tails and are therefore recognized and mobilized by the enzymatic machinery of L2 LINEs[117], and two of these have CR1-like tails and are thus mobilized by CR1 LINEs[118,119]. RepeatMasker analysis of the tuatara genome assembly detected copies of a total of 60 SINE subfamilies and the relative age distributions of the most common ones are shown as a SINE retrotransposon landscape (Supplementary Information Figure 5.3, 5.4). At least 17 of these SINE subfamilies were recently active, as each of them has a total of over 1,000 bp of copies with distances to consensus ≤ 5% (Supplementary Information Figure 5.3a). While ancient amniote CORE-SINE subfamilies (i.e., LFSINE[120], AmnSINE[121,122], MIR[123,124]) are present in tuatara, likely since the amniote ancestor, most SINEs are of more recent origin. Half of the SINE-derived base pairs below with ≤ 5% distance to consensus belong to a family of CR1-mobilized SINEs (tuaCR1-SINE1) and the other half falls into three families of tuatara-specific MIR elements (thus belonging to CORE-SINEs); namely tuaMIR-a, tuaMIR-b, and tuaMIR-c (Supplementary Information Figure 5.3, 5.4). These three MIR families consist of multiple closely related subfamilies, suggesting that the tuatara lineage underwent continuous activity and diversification of MIR elements (Supplementary Information Figure 5.5). There even seems to be potentially ongoing MIR activity, as implied by the presence of dozens or hundreds of tuaMIR-b and tuaMIR-c copies with 0% distance to consensus (Supplementary Information Figure 5.3b). Notably, the MIR subfamily diversity of tuatara (Supplementary Information Figure 5.4, 5.5) surpasses that of all previously analyzed genomes, even that of marsupial and monotreme mammals where millions of copies are derived from relatively few MIR subfamilies[125,126].

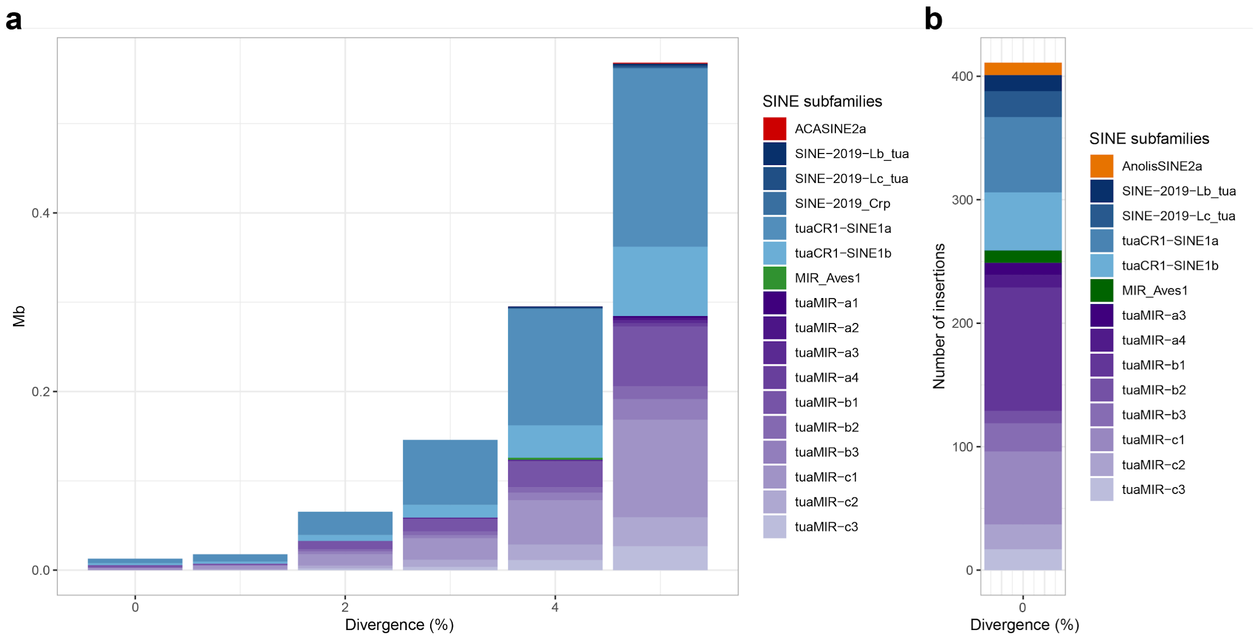

Supplementary Information Figure 5.3: Recent SINE retrotransposon landscape of the tuatara genome. (**a**) Only SINE subfamilies that occupy more than 1,000 bp with distances to consensus ≤ 5% are shown. (**b**) Subfamily composition of potentially active SINE retrotransposons, i.e., copies/fragments with 0% divergence to consensus. Only the 14 SINE subfamilies with at least 10 copies in this divergence bin are shown.

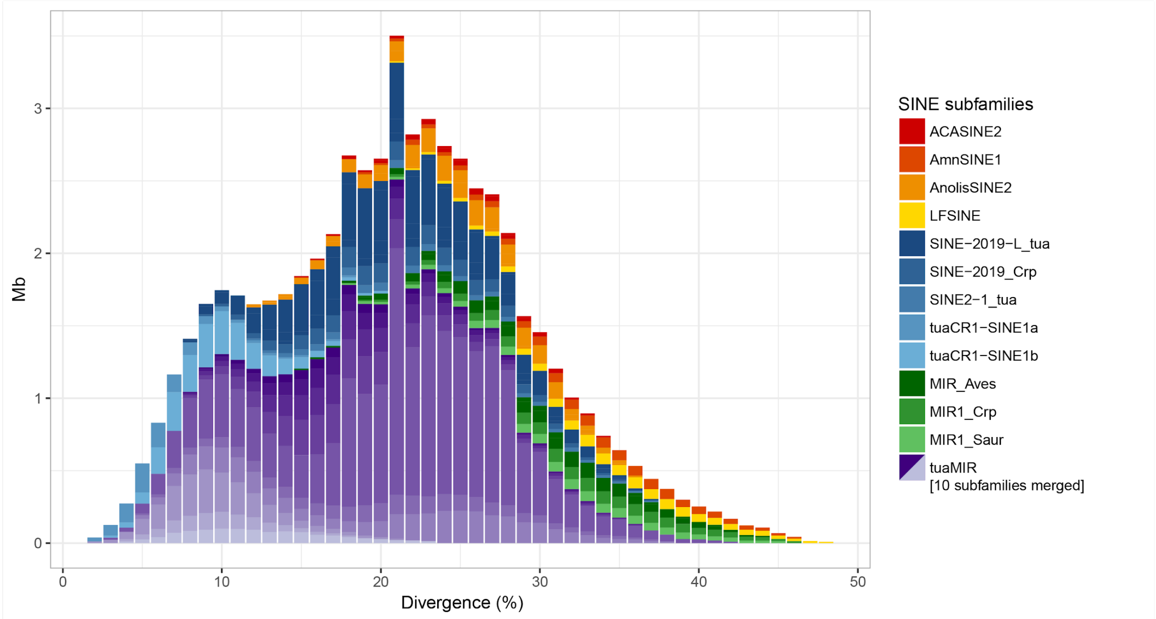

Supplementary Information Figure 5.4: Landscape plot of all SINE retrotransposons. Only SINE subfamilies that occupy more than 0.01 Mb are shown.

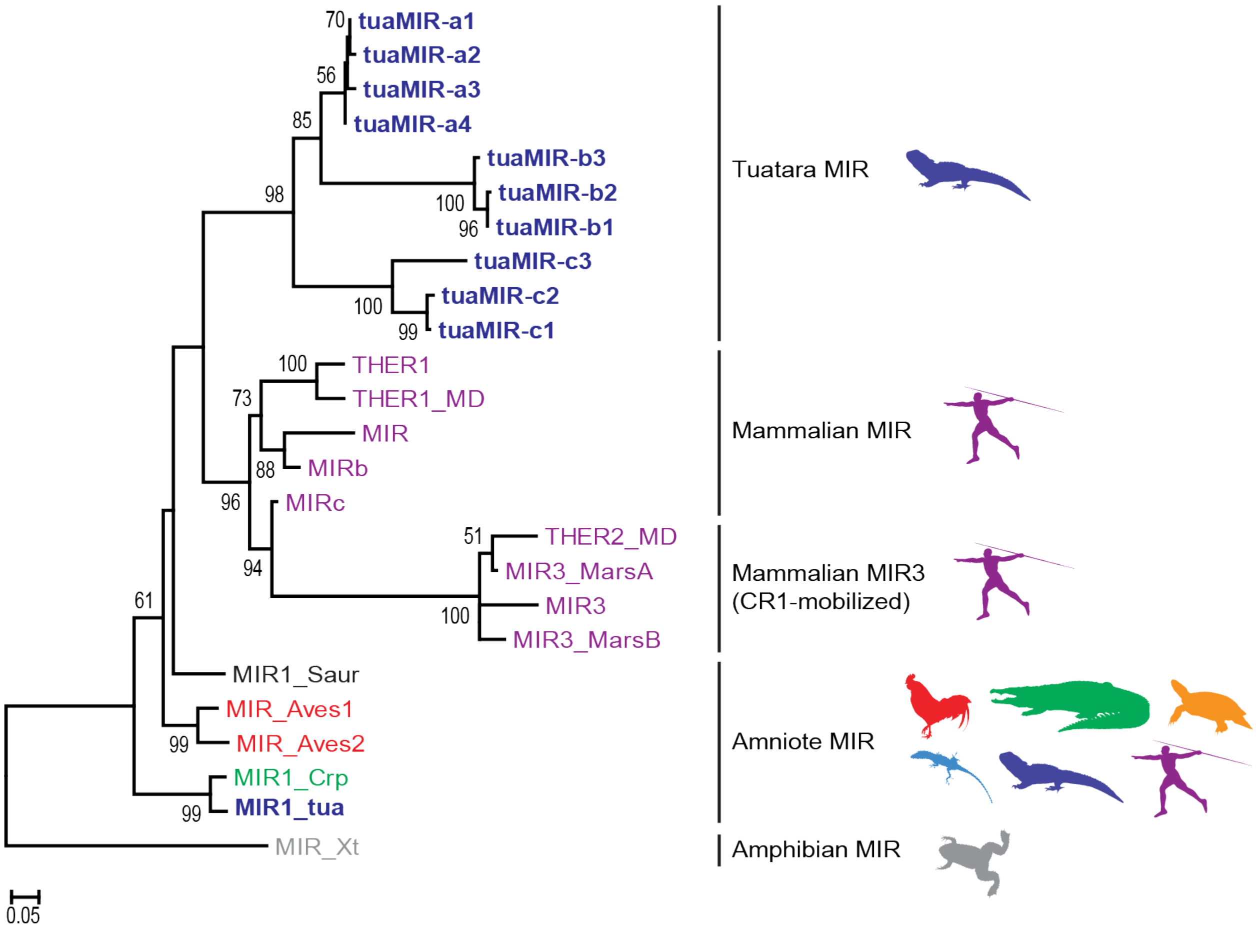

Supplementary Information Figure 5.5: Diversification of MIR SINEs in the tuatara lineage. Maximum likelihood phylogram with consensus sequences colour-coded according to the genome of first description. MIR1_Saur was first described in the American alligator genome and was probably active in the common ancestor of Sauropsida[108]. The presence of low copy numbers of MIR_Aves1 and MIR_Aves2 in mammalian genomes (Alexander Suh, unpublished data) suggests that all these early-branching MIR lineages resulted from ancient sauropsid or amniote activity. Note that all MIR families have L2-like tails with the exception of mammalian MIR3 which exhibits CR1-like tails, therefore parasitizing a different superfamily of LINEs.

Supplementary Information Table 5.1: Characteristics of novel tuatara DNA transposons.

Class	Subclass	Superfamily	Family	Subfamily	Similarity to Repbase repeats	Comment	Consensus status	Consensus length	TSD	TSD motif	TIR
DNA transposon	DNA	Harbinger	Chompy-2	Chompy-2a_tua	Chompy-2_Croc (79%; Crocodylia)	Non-autonomous	Complete	79	3		7
DNA transposon	DNA	Harbinger	Chompy-2	Chompy-2b-tua	Chompy-2_Croc (81%; Crocodylia)	Non-autonomous	Complete	70	3		17
DNA transposon	DNA	Harbinger	Chompy-4	Chompy-4-L_tua	Partially Chompy-4_Croc (86%; Crocodylia)	Non-autonomous	Complete	90	3		43
DNA transposon	DNA	Harbinger	Harbinger-2	Harbinger-2-L_tua	Partially Harbinger-3_AMi (65%; Alligator mississippiensis) + Harbinger-2_AMi (69%; Alligator mississippiensis)		Complete	6627	3		20
DNA transposon	DNA	Harbinger	Harbinger-3	Harbinger-3-L_tua	Partially Mariner-N13_HSal (83%; Harpegnathus saltator) + LTR-12_AMi (67%; Alligator mississippiensis) + Harbinger-3_AMi (64%; Alligator mississippiensis) + hAT-N11_AMi (69%; Alligator mississippiensis) + L1MA9_5 (73%; Mammalia)	Harbinger with lots of other TE fragments nested within, potentially non-autonomous	Complete	6639	3		20
DNA transposon	DNA	hAT	hAT-1	hAT-1_tua	hAT-1_AMi (96%; Alligator mississippiensis)		Complete	3504	8		9
DNA transposon	DNA	hAT	hAT-13	hAT-13_tua	hAT-13_Ami (89%; Alligator mississippiensis)	Non-autonomous	Complete	339	8		12
DNA transposon	DNA	hAT	hAT-39	hAT-39_tua	hAT-39_LCh (84%; Latimeria chalumnae) except for middle part		Complete	1171	8		22
DNA transposon	DNA	hAT	hAT-4	hAT-4_tua	hAT-4_Crp (85%; Crocodylus porosus)	Non-autonomous	Complete	401	8		14

DNA transposon	DNA	hAT	hAT-N18	hAT-N18_tua	hAT-N18_Crp (81%; Crocodylus porosus) + hAT-N18_SSa (75%; Salmo salar)	Non-autonomous	Complet e	177	8		16
DNA transposon	DNA	hAT	Charlie1	Charlie1-L_tua	Partially CHARLIE1 (84%; Mammalia)	Contains CR1 fragment (72% similarity to CR1-C; Gallus gallus)	Complet e	1308	8	NNNTAN NN	2
DNA transposon	DNA	hAT	hAT-29	hAT-29-L_tua	Partially hAT-29_CPB (78%; Chrysemys picta)	Non-autonomous	Complet e	418	8		5
DNA transposon	DNA	hAT	hAT-66	hAT-66-L-tua	Partially hAT-66_HM (83%; Hydra magnipapillata)	Non-autonomous	Complet e	920	8		15
DNA transposon	DNA	hAT	hAT-7	hAT-7-L-tua	Partially hAT-7_CPB (76%; Chrysemys picta) + hAT-5_CPB (74%; Chrysemys picta)	Non-autonomous	Complet e	307	8	NNNTAN NN	14
DNA transposon	DNA	hAT	hAT-N1	hAT-N1-L_tua	Partially hAT-N1_XT (82%; Xenopus tropicalis)	Non-autonomous	Complet e	470	8	NNNTAN NN	12
DNA transposon	DNA	hAT	hAT-N11	hAT-N11-L_tua	Partially hAT-N11_AMi (81%; Alligator mississippiensi s) + hAT-N4_Gav (77%; Gavialis gangeticus)	Non-autonomous	Complet e	592	8		10
DNA transposon	DNA	hAT	hAT-N18	hAT-N18-La_tua	Partially hAT-N18_Crp (79%; Crocodylus porosus) + hAT-N27_Ssa (84%; Salmo salar)	Non-autonomous	Complet e	175	8	NNNTAN NN	16
DNA transposon	DNA	hAT	hAT-N18	hAT-N18-Lb_tua	Partially hAT-N18_Crp (87%; Crocodylus porosus)	Non-autonomous	Complet e	177	8	NNNTAN NN	16
DNA transposon	DNA	hAT	hAT-1598	hAT-1598-L_tua	Partially hAT-N22_Ami (79%; Alligator mississippiensi s) + hAT-1598_Gav (80%; Gavialis gangeticus)	Non-autonomous	Complet e	507	8		11
DNA transposon	DNA	hAT	hATN-3	hATN-3-La_tua	Partially hATN-3_SM (79%; Schmidtea mediterranea)	Non-autonomous	Complet e	320	8	NNNTAN NN	16

DNA transposon	DNA	hAT	hATN-3	hATN-3-Lb_tua	Partially hATN-3_SM (80%; Schmidtea mediterranea)	Non-autonomous	Complete	321	8	NNNTANNN	16
DNA transposon	DNA	hAT	hATN-3	hATN-3-Lc_tua	Partially hATN-3_SM (82%; Schmidtea mediterranea)	Non-autonomous	Complete	314	8	NNNTANNN	16
DNA transposon	DNA	hAT	hATN-3	hATN-3-Ld_tua	Partially hATN-3_SM (83%; Schmidtea mediterranea)	Non-autonomous	Complete	320	8	NNNTANNN	16
DNA transposon	DNA	hAT	nhAT-3	nhAT-3-La_tua	Partially nhAT-3_EF (81%; Eptesicus fuscus)	Non-autonomous	Complete	386	8	NNNTANNN	14
DNA transposon	DNA	hAT	nhAT-3	nhAT-3-Lb_tua	Partially nhAT-3_EF (86%; Eptesicus fuscus)	Non-autonomous	Complete	102	8	NNNTANNN	3
DNA transposon	DNA	Maverick	Polinton-1	Polinton-1_tua.inc	Polinton-1_CPB (73%; Chrysemys picta)		Incomplete 5' and 3' ends	6211	?		?
DNA transposon	DNA	piggyBac	piggyBac-1	piggyBac-1-La_tua	Partially piggyBac-1_Ami (68%; Alligator mississippiensis)	Non-autonomous	Complete	370	4	TTAA	4
DNA transposon	DNA	piggyBac	piggyBac-1	piggyBac-1-Lb_tua	Partially piggyBac-1_AMi (69%; Alligator mississippiensis)		Complete	1196	4	TTAA	11
DNA transposon	DNA	Zator	Zator-2	Zator-2_tua	Zator-2_HM (73%; Hydra magnipapillata)	Non-autonomous	Complete	363	3		26
DNA transposon	DNA	Mariner	tuaMar	tuaMar1	None	Non-autonomous	Complete	192	2	TA	22
DNA transposon	DNA		tuaDNA1	tuaDNA1a	None		Complete	1737	8		3
DNA transposon	DNA		tuaDNA1	tuaDNA1b	None		Complete	1741	8		3
DNA transposon	DNA		tuaDNA1	tuaDNA1c	Partially hAT-N13_Crp (70%; Crocodylus porosus)		Complete	1756	8		12

DNA transposon	DNA		tuaDNA1	tuaDNA1d	None		Complet e	1741	8		12
DNA transposon	DNA		tuaDNA2	tuaDNA2a	None		Complet e	1056	8	NTAAAT AG	11
DNA transposon	DNA		tuaDNA2	tuaDNA2b	None		Complet e	1034	8		12
DNA transposon	DNA		tuaDNA3	tuaDNA3a	Partially Chompy-1_Croc (91%; Crocodylia)	Not a Harbinger due to different TSDs	Complet e	1365	8		11
DNA transposon	DNA		tuaDNA3	tuaDNA3b	Partially Chompy-3_Croc (87%; Crocodylia)	Not a Harbinger due to different TSDs; contains LTR fragment (75% similarity to Gypsy-50_GA-LTR; Gasterosteus aculeatus)	Complet e	1519	8		13
DNA transposon	DNA		tuaDNA4	tuaDNA4	Partially Chompy-4_Crp (78%; Crocodylus porosus)	Non-autonomous, not clear if Harbinger or not due to unknown TSDs	Complet e	725	?		9
DNA transposon	DNA		tuaDNA5	tuaDNA5	Partially Harbinger-1B_Crp (83%; Crocodylus porosus)	Not a Harbinger due to different TSDs	Complet e	1532	8		4
DNA transposon	DNA		tuaDNA6	tuaDNA6	None	Contains Mariner fragment (81% similarity to Mariner-31_HM; Hydra magnipapillata)	Complet e	1176	8		15
DNA transposon	DNA		tuaDNA7	tuaDNA7	None	Non-autonomous	Complet e	563	3		13
DNA transposon	DNA		tuaDNA8	tuaDNA8	None	Non-autonomous	Complet e	336	8		15
DNA transposon	DNA		tuaDNA9	tuaDNA9	None	Non-autonomous	Complet e	382	8		11
DNA transposon	DNA		tuaDNA10	tuaDNA10	None	Non-autonomous	Complet e	303	?		12
DNA transposon	DNA		tuaDNA11	tuaDNA11	None	Non-autonomous	Complet e	654	8		10

DNA transposon	DNA		tuaDNA12	tuaDNA12	None	Non-autonomous	Complete	827	8		3
DNA transposon	DNA		tuaDNA13	tuaDNA13	None	Non-autonomous	Complete	860	8		10
DNA transposon	DNA		tuaDNA14	tuaDNA14	None	Non-autonomous	Complete	738	8		18
DNA transposon	DNA		tuaDNA15	tuaDNA15	None	Non-autonomous	Complete	368	8		15
DNA transposon	DNA		tuaDNA16	tuaDNA16	None	Non-autonomous	Complete	499	?		19
DNA transposon	DNA		tuaDNA17	tuaDNA17	None	Non-autonomous	Complete	468	8		7
DNA transposon	DNA		tuaDNA18	tuaDNA18	None	Non-autonomous	Complete	689	8		16
DNA transposon	DNA		tuaDNA19	tuaDNA19	None	Non-autonomous, contains Mariner fragment (75% similarity to MARINERNA8_MD; Monodelphis domestica)	Complete	264	8		8
DNA transposon	DNA		tuaDNA20	tuaDNA20	None	Non-autonomous, contains SINE head (78% similarity to MIR1_Crp; Crocodylus porosus)	Complete	423	8		15
DNA transposon	DNA		tuaDNA21	tuaDNA21	None		Complete	1382	8		14
DNA transposon	DNA		tuaDNA22	tuaDNA22	None		Complete	1185	8		4
DNA transposon	DNA		tuaDNA23	tuaDNA23	None		Complete	1031	8		9
DNA transposon	DNA?		tuaDNAL	tuaDNAL-1	None	Contains Mariner fragment (67% similarity to Mariner-7N1_CPB; Chrysemys picta)	Complete	2641	?		3
DNA transposon	DNA		MER45	MER45B-L_tua	Partially MER45B (80%; Homo sapiens)	Non-autonomous	Complete	889	8		15

DNA transposon	DNA		MER45	MER45R-La_tua	Partially MER45R (81%; Homo sapiens)		Complet e	1655	?		14
DNA transposon	DNA		MER45	MER45R-Lb_tua	Partially MER45R (83%; Homo sapiens)	Non-autonomous	Complet e	242	8		14
DNA transposon	DNA		MER45	MER45R-Lc_tua	Partially MER45R (85%; Homo sapiens)	Non-autonomous	Complet e	251	8		14
DNA transposon	DNA		MER58	MER58D-L_tua	Partially MER58D (84%; Mammalia)	Non-autonomous	Complet e	668	8		16
Retrotransposon	LTR	ERVK?	tuaLTR1	tuaLTR1	None	Solo-LTR	Complet e	511	5		3
Retrotransposon	LTR	Gypsy	LTR-58	LTR-58-L_tua	Partially LTR-58_Gav (71%; Gavialis gangeticus)	Solo-LTR	Complet e	1008	5		6

Supplementary Information Table 5.2: Characteristics of novel tuatara SINE retrotransposons.

Class	Subclass	Superfamily	Family	Subfamily	Similarity to Repbase repeats	Comment	Consensus status	Consensus length	TSD	TSD motif	TIR
Retrotransposon	SINE	MIR	MIR1	MIR1_tua	MIR1_Crp (96%; Crocodylus porosus)	Very short element, core region only	Complete	55	var		N/A
Retrotransposon	SINE	MIR	tuaMIR	tuaMIR-a1	THER1 (80%; Mammalia)		Complete	235	var		N/A
Retrotransposon	SINE	MIR	tuaMIR	tuaMIR-a2	THER1 (80%; Mammalia)		Complete	238	var		N/A
Retrotransposon	SINE	MIR	tuaMIR	tuaMIR-a3	THER1 (79%; Mammalia)		Complete	245	var		N/A
Retrotransposon	SINE	MIR	tuaMIR	tuaMIR-a4	THER1 (79%; Mammalia)		Complete	244	var		N/A
Retrotransposon	SINE	MIR	tuaMIR	tuaMIR-b1	Partially SINE2-1_XT (78%; Xenopus tropicalis)	Different SINE head (82% similarity to SINE2-2_ACar; Anolis carolinensis)	Complete	295	var		N/A
Retrotransposon	SINE	MIR	tuaMIR	tuaMIR-b2	Partially SINE2-1_XT (78%; Xenopus tropicalis)	Different SINE head (79% similarity to MIR3_MarsB; Marsupialia)	Complete	274	var		N/A
Retrotransposon	SINE	MIR	tuaMIR	tuaMIR-b3	Partially SINE2-1_XT (75%; Xenopus tropicalis)	Different SINE head (79% similarity to MIR1_AMi; Alligator mississippiensis)	Complete	296	var		N/A
Retrotransposon	SINE	MIR	tuaMIR	tuaMIR-c1	MIR_Aves1 (76%; Aves)	Potentially different SINE head	Complete	249	var		N/A
Retrotransposon	SINE	MIR	tuaMIR	tuaMIR-c2	MIR_Aves1 (75%; Aves)	Potentially different SINE head	Complete	241	var		N/A

Retrotransposon	SINE	MIR	tuaMIR	tuaMIR-c3	Partially MIR (93%; Mammalia)	Different SINE head (79% similarity to SINE2-2_ACar; Anolis carolinensis)	Complete	269	var		N/A
Retrotransposon	SINE	tRNA	SINE2-1	SINE2-1_tua	SINE2-1_Croc (79%; Crocodylia)		Complete	266	var		N/A
Retrotransposon	SINE	tRNA	tuaCR1-SINE	tuaCR1-SINE1a	Partially Squam2 (81%; Squamata) CR1-15_Ami (81%; Alligator mississippiensis)	tRNA-Ala head, CR1 tail	Complete	284	var		N/A
Retrotransposon	SINE	tRNA	tuaCR1-SINE	tuaCR1-SINE1b	Squam2 (83%; Squamata)	tRNA-Ala head, CR1 tail	Complete	270	var		N/A
Retrotransposon	SINE?	L2	SINE-2019	SINE-2019-La_tua	Partially SINE-2019_Crp (69%)	Different SINE head	Complete	457	var		N/A
Retrotransposon	SINE?	L2	SINE-2019	SINE-2019-Lb_tua	Partially SINE-2019_Crp (69%; Crocodylus porosus)	L2 tail (82% similarity to L2-2_Croc; Crocodylia)	Complete	885	var		N/A
Retrotransposon	SINE?	L2	SINE-2019	SINE-2019-Lc_tua	Partially SINE-2019_Crp (70%)	Different SINE head	Complete	738	var		N/A
Retrotransposon	SINE?	L2	SINE-2019	SINE-2019-Ld_tua	Partially SINE-2019_Crp (76%; Crocodylus porosus)	Different SINE head	Complete	315	var		N/A

6 DIVERSITY OF LTR RETROELEMENTS IN THE TUATARA GENOME

Jose Horacio Grau*

*Correspondence to: jh.grau.jipoulou@gmail.com

6.1 Introduction

Transposable elements are generally classified into Class I elements (called retrotransposons or retroelements), which use an RNA intermediate for transposition in a copy paste fashion; and Class II elements, which replicate without an RNA intermediate, either by a cut-and-paste mechanism (DNA transposons), by rolling circle DNA replication (helitrons), or by so far unknown mechanisms (politrons/mavericks). Among the Class I elements two major subclasses are recognized: (1) retroelements (REs) with long terminal repeats (LTRs) and (2) elements without LTRs (non-LTR REs)[127,128]. LTR REs, can be classified into four major families, namely Bel/Pao, Ty1/Copia, Ty3/Gypsy, and retroviruses[129,130]. A common LTR retrotransposon typically encodes two polyproteins, termed GAG and POL. The capsid protein (GAG) usually contains matrix, capsid, and nucleocapsid domains; POL consists of aspartic proteinase (AP), reverse transcriptase (RT), ribonuclease (RN), and integrase (INT) domains, the latter three (RT, RN, INT) are responsible for retrotranscribing cDNA from RNA intermediates and inserting it into the host genome. Endogenous retroviruses constitute a specific class of LTR REs that sometimes additionally contain an open reading frame (ORF) for an envelope protein (ENV), which enables ERVs to move from one cell to another. In contrast, all other LTR REs either lack or contain a remnant of an ENV gene and can only reinsert into their own host genome[131–133]. There are, however, ERVs that secondarily lost their ENV gene and thus their infectious ability. Such ERVs are retrotransposing instead of infecting other cells like typical retroviruses[134].

As a precondition for understanding the role of LTR retroelements in shaping animal genomes the diversity of these elements has to be systematized[135–137]. Several computer programs have been developed to automatically detect LTR REs[138]. Some of these computing methods have made it possible to detect and identify previously unknown elements[139] and there is an increasing body of work that suggests many genomes still host remnants of inactive retrotransposons corresponding to ancient retrotransposition events. Here we extend these analyses to explore LTR RE diversity in the tuatara genome.

6.2 Results

We identified approximately 7,500 full-length long terminal repeat (LTR) retroelements including endogenous retroviruses (ERVs), which we classified into at least 12 groups using phylogenetic reconstructions of their retrotranscriptase domains (Supplementary Information Figure 6.1). We

identified nearly 450 ERVs from five major retroviral clades and some 5,500 Ty3/Gypsy elements representing five major clades. Notably, a Ty1/Copia element (Mtanga-like) is abundant in the tuatara genome with more than 1,140 full-length elements. The general spectrum of tuatara LTR retroelements is comparable to that of other sauropsids, but lacks Bel/Pao LTR retroelements which are present in anole[140]. An unexpected result is the presence of at least 37 complete spumaretroviruses in the tuatara genome (Supplementary Information Figure 6.1, 6.2).

`

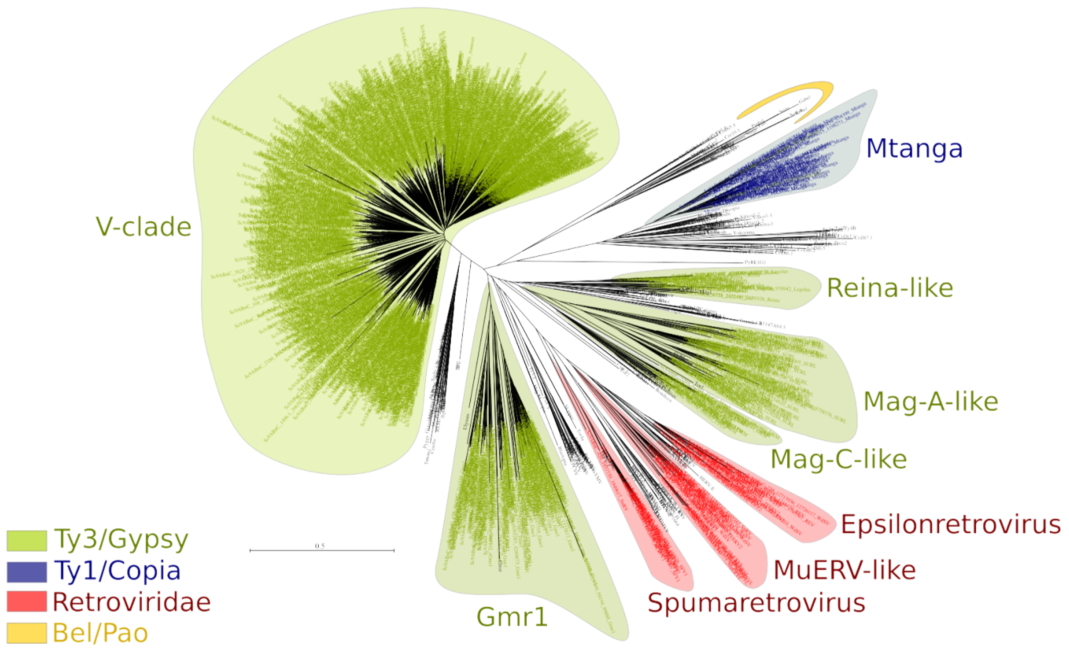

Supplementary Information Figure 6.1 The tuatara genome contains ~7500 full-length long terminal repeat (LTR) retroelements including nearly 450 endogenous retroviruses (ERVs). The general spectrum of tuatara LTR retroelements is comparable to that of other sauropsids, but lacks Bel/Pao LTR retroelements which are present in anole, and has an abundance of Ty3/Gypsy retroelements.

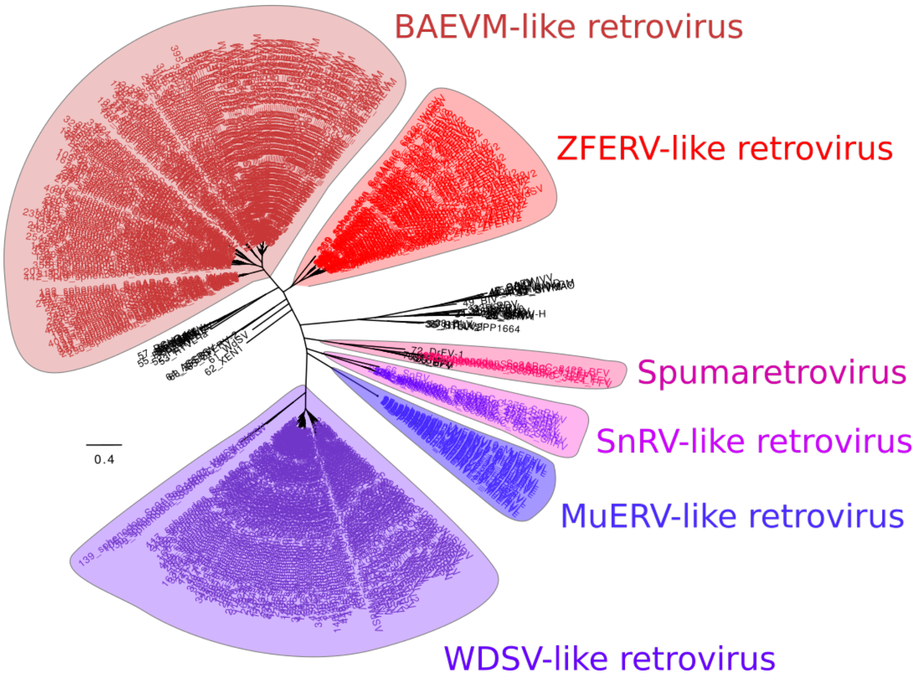

Supplementary Information Figure 6.2 Maximum likelihood phylogram of endogenous retroviruses found in the tuatara genome. Colour-coded according to the retrovirus family detailed in the phylogram. No Betaretrovirus ERVs were found in the tuatara genome.

6.3 Discussion and conclusions

The general spectrum of LTR-retroelements with the genome of the tuatara is comparable to that of other Sauropsids, in that it contains abundant LTR-retroelements typically expanded in both Lepidosaurs and Archeosauronmorphs. An unexpected result is the presence of an old spumaretrovirus clade within the tuatara genome. Spumaretroviruses are considered among the most ancient ERVs[141,142] and were previously found only in coelacanth[143], sloth[144], aye-aye[145] and Cape golden mole[146]. Thus, the occurrence of several full-length endogenous spumaretroviruses in tuatara, some encoding a complete envelope gene, is the first in a sauropsid genome.

6.4 Methods

LTR RE searches used a reference collection of retroelement domains and alignments obtained from the publicly available Gypsy Database 2.0 (GyDB)[137]. For the detection of LTR REs the retro-transcriptase (RT) domain was used because it is the best conserved through evolutionary time[147]. In order to obtain a custom representation of the LTR RE diversity the program suffixerator, which is part of GenomeTools[148], was applied with default parameters and created an

enhanced suffix file which was later scanned with LTR harvest[49] with default parameters to predict putative LTR REs through the detection of their LTRs. To leave out the false LTR RE predictions made by LTR harvest, we then searched each LTR harvest predicted sequence against a database of RT domains of GyDB using blastx. Matches with an e-value of 1e-30 and alignments for only the best match were reported. Since blastx has the ability to produce full-length amino acid alignments from nucleotide sequences with little penalty for frameshifts, a bioperl script was used to parse out putative protein alignments of the RT domain from blastx report. Because this approach yielded thousands of RT alignments for a single genome, we clustered the LTR RE dataset using the program CD-HIT[44,149] with an identity threshold of 80%, and discarded sequences shorter than 120 aa to reduce the high number of similar and identical copies of each retroelement.

The non-redundant dataset was fused with the complete RT domains of GyDB and aligned and used to infer a series of maximum-likelihood (ML) and neighbour-joiningNJ) trees in order to accurately place the retroelements in a phylogenetic context. All alignments were conducted with the program Mafft[150] using local alignment and a Blosum 30 aa substitution matrix as parameters. Final alignment files were prepared by removing columns with more than 70% of gaps. ML trees were inferred with RaxML 8.2[151] using the PROTCATRTREV protein model of evolution, NJ trees were inferred with NINJA 1.2.2[152]. Both ML and NJ trees essentially yielded the same results.

7 TUATARA GENOME: NON-CODING RNA ANNOTATION AND ANALYSIS

Paul P. Gardner* and James M. Paterson

*Correspondence to: paul.gardner@otago.ac.nz

7.1 Introduction

Non-coding RNAs (ncRNAs) perform vital roles in translation (e.g. ribosomal RNAs, transfer RNAs), messenger RNA maturation (e.g. spliceosomal RNAs) and gene regulation (e.g. microRNAs)[153,154]. Yet the annotation of ncRNAs is complicated by the fact that they tend to conserve complex secondary structures more than primary sequence. To address this problem we use covariance models (CMs) that account for both covarying base-paired sites and sequence conservation[155,156]. These have proven to have a major accuracy advantage over alternative methods for ncRNA gene annotation[157].

7.2 Methods

To annotate ncRNAs in the tuatara genome (version 30Sep2015_rUdWx) we used CMs from the RNA families (Rfam) database (version 13.0)[55] and tRNAscan-SE (version 1.3.1)[57,158]. Overlapping annotations resulting from deep homology were resolved using clan annotations from Rfam[159] as previously described[160]. In brief, in cases of overlapping tRNA and tRNAscan-SE annotations, the more specific tRNAscan-SE results were used, if families from the same clan overlap, the family with the highest is used in a winner-takes-all approach.

The Anolis genome (version AnoCar2.0) was annotated using the same methodology. Comparisons between ncRNA copy numbers encoded in the Anolis and tuatara genomes give an indication of expansions and contractions of different families since these reptiles last shared a common ancestor (~250 million years ago).

High genomic copy-number families were assessed for likelihood of incorporation into repeat-generating machinery (e.g. retrotransposons)[117]. See *Supplementary Materials 5 Repeat annotation: SINES and DNA transposons* for further details.

7.3 Figures

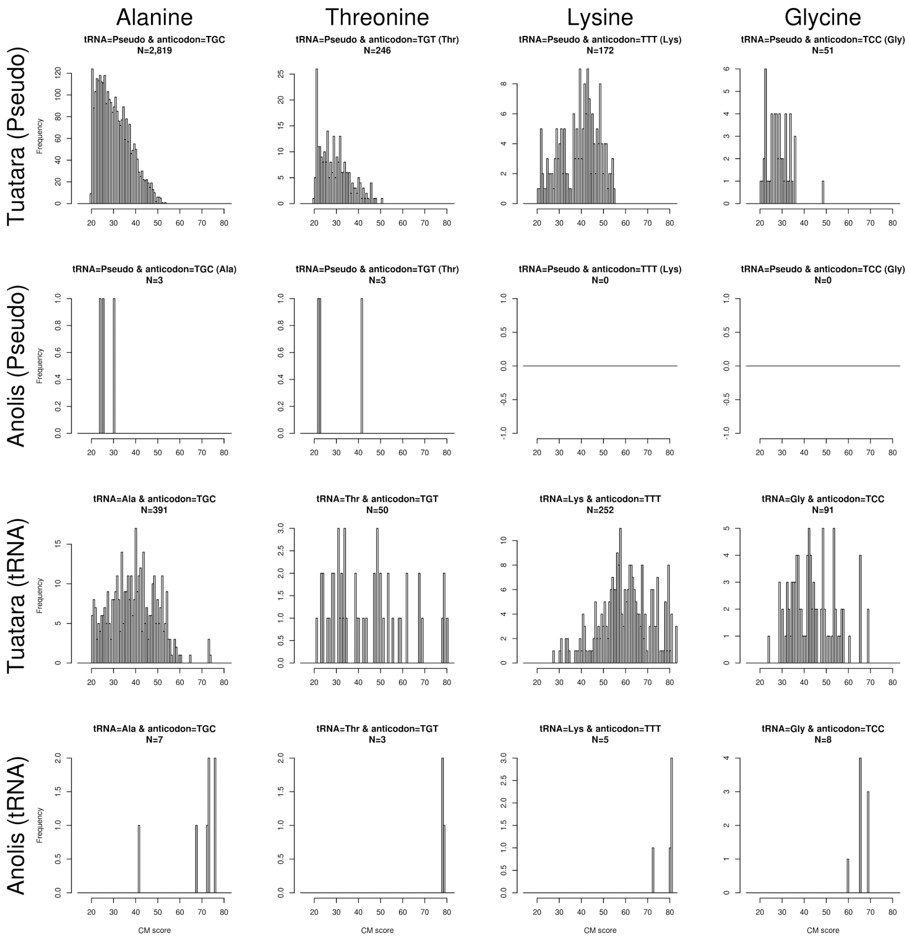

Supplementary Information Figure 7.1: Major expansions of alanine, threonine, lysine and glycine tRNA isotypes. The bitscore distributions for functional and pseudogenised genomic copies are shown for both tuatara and Anolis paralogues. Bitscores give an indication of similarity to an idealised functional tRNAs.

7.4 Tables

Supplementary Information Table 7.1: Genomic copy numbers of RNA families and broad RNA types for annotated tuatara and Anolis genomes. Only the high copy-number (N>10) ncRNA families have been included.

ncRNA type	ncRNA family	Anolis genomic copy-number	Tuatara genomic copy-number
**Cis-regulatory elements**		110	196
	Histone 3` UTR stem-loop	67	144
**ncRNA genes**		362	66
	7SK RNA	307	18
**lncRNAs**		14	14
**miRNAs**		290	319
	mir-598	65	18
**rRNAs**		1640	381
	5S rRNA	1596	85
	LSU rRNA	29	229
	SSU rRNA	15	67
**C/D box snoRNAs**		108	140
**H/ACA box snoRNAs**		73	90
**scaRNA**		13	16
**Spliceosomal RNAs**		264	162
	U1	38	22
	U2	128	23
	U6	67	50
**tRNA**		375	3341

Supplementary Information Table 7.2: Genomic copy numbers of selected tRNA isotypes (functional and pseudogenised copies, as predicted by tRNAscan-SE). Only high copy-number (∑N>100) isotypes have been reported.

		**Anolis**		**Tuatara**	
**Isotype**	**Anticodon**	**#tRNAs**	**#Pseudogenes**	**#tRNAs**	**#Pseudogenes**
Thr	AGT	4	5	90	23
Met	CAT	13	0	90	7
Lys	CTT	7	2	148	117
Ser	GCT	6	15	66	42
Ser	GGA	1	207	1	7
His	GTG	5	20	75	28
Val	TAC	2	1	18	112
SeC/SeC(e)	TCA	7	0	22	0
Gly	TCC	8	0	91	51
Ala	TGC	7	3	391	2819
Pro	TGG	4	6	61	142
Thr	TGT	3	3	50	246
Glu	TTC	9	2	84	57
Lys	TTT	5	0	252	172

8 THE nCpG DISTRIBUTION OF THE TUATARA GENOME AND ANALYSIS OF PATTERNS AND EVOLUTION OF CpG CONTENT IN VERTEBRATES

Valeria Velásquez Zapata, Zhiqiang Wu, Nicole Valenzuela*

*Correspondence to: nvalenzu@iastate.edu

8.1 Introduction

DNA methylation is an epigenetic modification common in animals where methyl groups are added to cytosine nucleotides[161], thus altering gene expression by preventing transcription factor binding or favouring repressor binding without changing the DNA sequence[162–164]. Because DNA methylation participates in development, disease, and mediates responses to environmental inputs[165–168], increasing efforts are devoted to understanding its function and evolution. Vertebrate DNA methylation targets CpG dinucleotides across genic and intergenic regions[169], such that the distribution of CpG within genomes can impact DNA methylation and its regulatory consequences[170]. The normalized CpG content (or nCpG) is used as an *in silico* proxy of DNA methylation. This nCpG measures the ratio of the observed to expected abundance of CpG dinucleotides in a particular DNA sequence given the frequency of cytosines (C) and guanines (G) present in the genome [CpG(O/E) = CpG observed/expected][171]. 5-methylcytosine tends to deaminate spontaneously, thus mutating to thymine and reducing the nCpG content overtime, rendering nCpG an index of historic methylation[172–175].

The genomic distribution of nCpG content has been studied in several vertebrates, including human and other primates, opossum, platypus, chicken, turtle, lizard, frog, fish, and in tunicates (a chordate)[170,176,177]. These comparative studies of nCpG revealed commonalities and differences across vertebrate lineages, particularly between painted turtles and other chordates[177]. However, because painted turtle is the only vertebrate with temperature-dependent sex determination (TSD) whose nCpG has been studied thus far while all others have genotypic sex determination (GSD), it is difficult to discern if the differences observed in turtle are due to its sex-determining mechanism or to lineage specific effects. Here we analyze the nCpG distribution of the newly sequenced tuatara genome (a TSD reptile) and other TSD and GSD reptiles (some not previously studied) plus chicken and human (Supplementary Information Figure 8.1), to illuminate the evolution of vertebrate nCpG content.

8.2 Methods

8.2.1 Data source

The annotated genomes of the tuatara (this study) and several TSD and GSD reptiles, chicken and human (both GSD) were obtained from public databases (Supplementary Information Table 8.1). Genomic regions were identified from the available annotation files. Two databases were used: the Ensembl database (data source for the *Homo sapiens* and *Pelodiscus sinensis* genomes) and the University of California Santa Cruz (UCSC) database (data source for the *Gallus gallus*, *Alligator mississippiensis*, *Anolis carolinensis*, and *Chrysemys picta bellii* genomes). For the UCSC genomes, gtf files were obtained in the table browser of the UCSC database, downloading format for genes and gene predictions, using the genescan annotation.

8.2.2 Data processing

We followed the protocols described in Radhakrishnan et al 2017[177] with some modifications. Briefly, CDS sequences were extracted to generate fasta files for gene, exons, and intron regions using Bedtools[178]. Promoter and intergenic sequences were then extracted from the upstream regions of these genes at various scales (100 bp, 300 bp, 500 bp, 1000 bp and 3000 bp) using the size chromosome data and gene direction information as a reference. Sequences with undefined nucleotides were eliminated prior to further analysis, which reduced the final dataset for some genomic regions in some taxa more substantially, particularly in reptiles other than tuatara (Supplementary Information Figure 8.2).

The nCpG content was calculated for each genic and intergenic region as

$$nCpG = \frac{(\frac{cg}{l})}{(\frac{c}{l}) \times (\frac{g}{l})}$$

where l = sequence length, c = number of cytosines, g = number of guanines, and cg = number of cytosines bordered by a guanine linked by a phosphate group (CpG)[171].

nCpG distributions for each genomic region were plotted using R software version 3.3.1[179]. The likelihood of a mixture model with one (G=1) and two (G=2) components was assessed using the R package Mclust[180] and the better fit model was chosen by a likelihood ratio test.

8.3 Results and discussion

We present the first examination of nCpG distribution in tuatara, the only representative of the reptilian order Rhynchocephalia, and shed new light on the commonalities and diversity of nCpG distribution across vertebrates, a trait that is linked to DNA methylation and may consequently

impact gene expression. First, our results show that the nCpG content of tuatara and other vertebrates is substantially below 1 in all genomic regions (Supplementary Information Figure 8.1), consistent with the notion that vertebrate genomes have experienced considerable historic methylation, leading to nCpG depletion from what is expected from C and G content alone[181]. The only exception was detected in chicken intergenic regions.

Second, our results revealed that the tuatara exhibited bimodal nCpG distribution in all genomic regions examined (gene promoters, exons, introns, and intergenic sequences), similar to recent reports in painted turtles, the only other TSD reptile previously studied[177], a pattern also shared by alligator (TSD) (Supplementary Information Figure 8.1).

Bimodal nCpG distributions at promoters characterize all vertebrates examined previously and in this study (human, non-human primates, opossum, platypus, chicken, alligator, *Chrysemys* turtle, *Pelodiscus* turtle, *Anolis* lizard, *Xenopus* frog, *Danio* fish) with the exception of platypus and *Ciona* tunicates (a representative of a chordate lineage basal to vertebrates)[170,176,177], suggesting that this bimodal pattern in promoters may have arisen with the emergence of vertebrates but was lost in monotremes. It should be noted that the previous assessment of unimodality in platypus promoters was qualitative[170] and it is likely that explicit statistical tests such as mixed models [[177] and this study] would have uncovered a better fit for a bimodal model, albeit much more subtle than the bimodality previously reported in birds and other mammals. Additionally, the promoter bimodality is generally stronger than bimodality in other genomic regions (Supplementary Information Figure 8.1), likely reflecting distinct functions of high- and low-nCpG promoters as reported for their contrasting role in regulating gene expression[176]. Whether promoter nCpG bimodality itself is driven by natural selection remains an open question.

Second, the bimodal nCpG pattern seen in introns of TSD reptiles (tuatara, painted turtle and alligator) would appear at first glance to mark a divide between TSD taxa and the GSD vertebrates previously reported to display a unimodal nCpG distribution in introns[170,176,177]. Furthermore, a bimodal intronic nCpG distribution was also reported in tunicates, which would suggest that such bimodal pattern may be ancestral to vertebrates and may have been lost in GSD vertebrate lineages, at least in those examined to date. However, here we revisited the genomes of several of these previously studied GSD vertebrates (human, chicken, anole) and investigated a GSD turtle for the first time (*Pelodiscus*). We tested explicitly for the existence of bimodality in all genomic regions examined using mixed models. Our results challenge the notion of intronic unimodality in nCpG distribution among vertebrates in general, supporting instead a bimodal pattern that appears to be ancestral and conserved across all vertebrates irrespective of their sex-determining mechanism. This finding agrees with the recent detection of bimodal DNA methylation in gene bodies in human, chicken, Danio fish, and Ciona tunicates[182].

Third, the observed bimodality is characterized by an overabundance (higher density) of sequences in the low-nCpG-mode distribution compared to the high- nCpG-mode distribution in all cases (Supplementary Information Figure 8.1), suggesting that if unchecked, the deamination of methyl-

cytosines continually impoverishes the vertebrate genome from its high-nCpG sequences. This nCpG attrition is particularly pronounced in exons, introns and intergenic regions of tuatara and human, where the bimodality is ostensibly subtler than in most other vertebrates (Supplementary Information Figure 8.1). The high levels of empirical methylation measured in tuatara compared to other vertebrates (affecting 81% of the remaining CpG sites) indicate that the nCpG depletion process may be still ongoing, perhaps related to the high abundance and recent activity of repetitive sequences detected in the tuatara genome. Thus, we hypothesize that perhaps the nCpG depletion in exons, introns and intergenic regions of the tuatara genome derives from adaptive methylation targeting repetitive elements for silencing over evolutionary time.

Taken together, our results from the comparative analysis of the tuatara genome and that of several other amniotes indicate that nCpG content has been shaped by distinct forces in promoter and non-promoter regions of the vertebrate genome, potentially reflecting adaptive responses to natural selection.

8.4 Figures

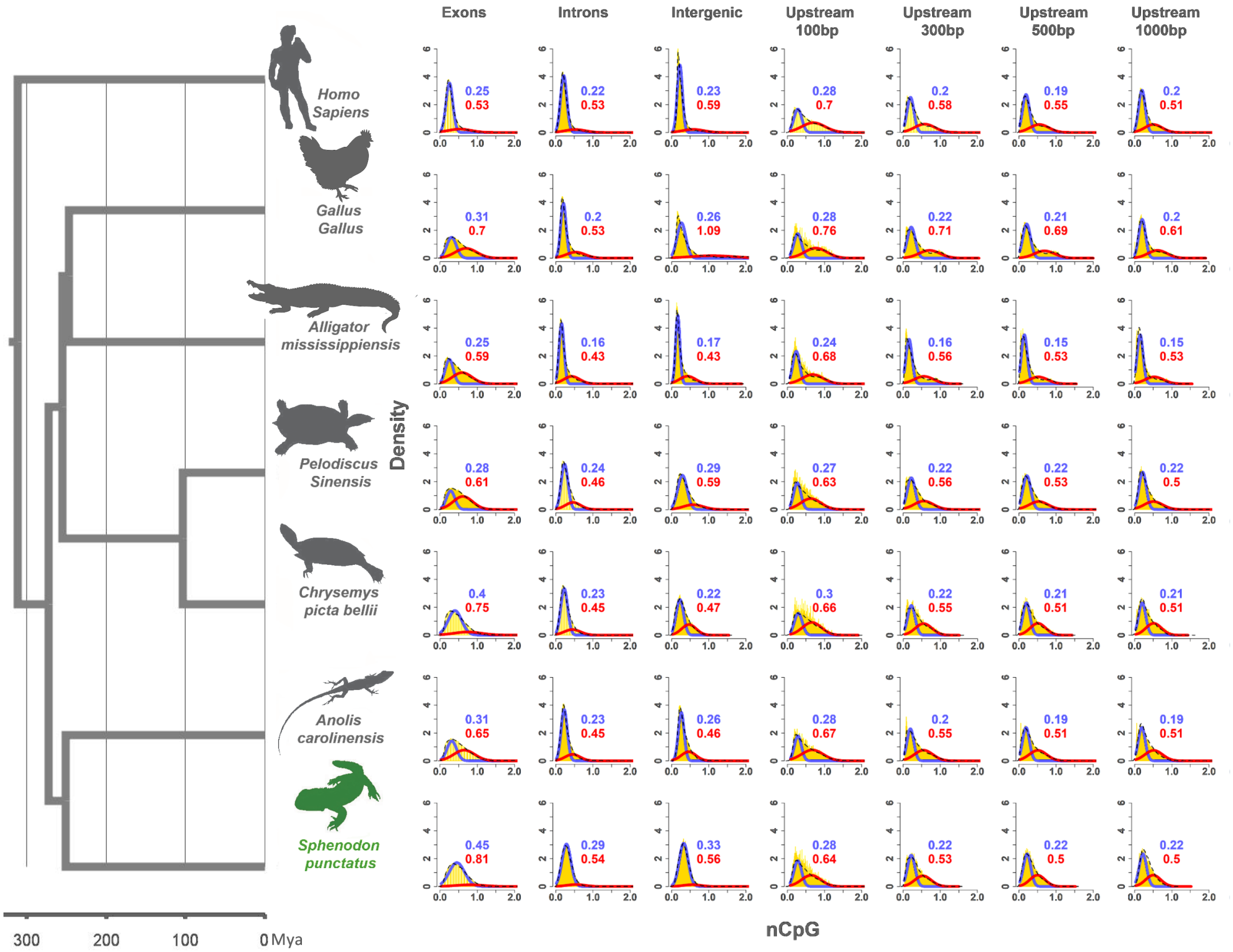

Supplementary Information Figure 8.1: Normalized CpG distributions (nCpG) for tuatara and other vertebrates. Tuatara show a statistically-significant bimodal nCpG distribution in all genomic regions examined (gene promoters, exons, introns, and intergenic sequences). Explicit testing using mixed models uncovered global nCpG bimodality across all taxa, indicating that bimodality is ancestral and highly conserved across vertebrates irrespective of sex-determining mechanism. Normalized CpG distributions (nCpG) in the annotated genomes of tuatara and other vertebrates by genomic region. nCpG content was calculated as (cg/l)/[(c/l)(g/l)] where l = sequence length, c = number of cytosines, g = number of guanines, and cg = number of cytosines bordered by a guanine linked by a phosphate group. The likelihood of a mixture model with one (G=1) and two (G=2) components was assessed and the better fit model was chosen by a likelihood ratio test using the R package Mclust. Sample sizes (N), test statistics, and p-values of the mixture models are presented in Supplementary Information Table 8.2. Attributions: Silhouettes are hand drawn from the original photos of Aaron Eckley, but for Anolis (Donald Hobern, CC-BY-2.0), crocodile and hen (Designed by Creazilla https://creazilla.com/).

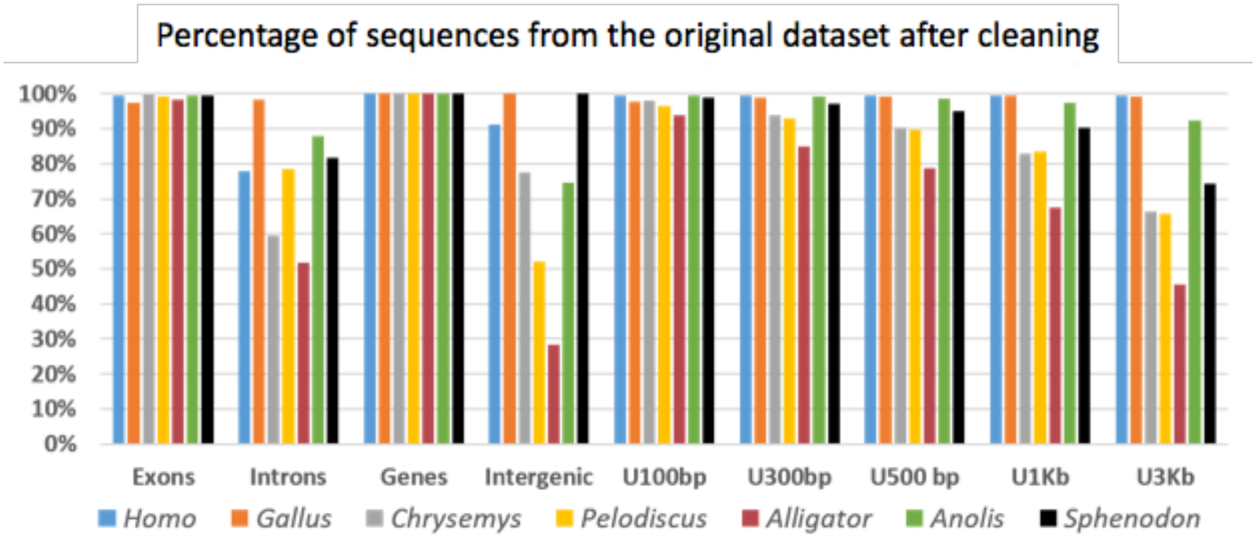

Supplementary Information Figure 8.2: Effect of removal of sequences with undefined nucleotides (Ns) from the database used in this study.

8.5 Tables

Supplementary Information Table 8.1: Vertebrate genomes examined in this study. SDM = Sex-determining mechanism. TSD = Temperature-dependent sex determination. GSD = Genotypic sex determination.

Group	Species	SDM	Source	Genome_ID
Tuatara	*Sphenodon punctatus*	TSD	UCSC	This study
Lizard	*Anolis carolinensis*	GSD	UCSC	AnoCar2.0 (GCA_000090745.1)
Turtle	*Chrysemys picta bellii*	TSD	UCSC	ChrPic1 (GCA_000241765.1)
Turtle	*Pelodiscus sinensis*	GSD	Ensembl	PelSin_1.0 (GCA_000230535.1)
Crocodilian	*Alligator mississippiensis*	TSD	UCSC	AllMis (GCA_000281125.1)
Bird	*Gallus gallus*	GSD	UCSC	Galgal5 (GCF_000002315.4)
Mammal	*Homo sapiens*	GSD	Ensembl	GRCh38.p7 (GCA_000001405.22)

Supplementary Information Table 8.2. Likelihood ratio test (LRT) of a mixture model with one (G = 1) and two (G = 2) components. N = number of regions in each category within the vertebrate genomes examined; df = degrees of freedom.

Taxa	Genomic Region	N	df	LRT Statistic	p-value
Homo sapiens	Exons	54128	5	15113	<0.00001
	Introns	202982	5	63156	<0.00001
	Intergenic	30613	5	14721	<0.00001
	Upstream 100bp	37111	5	5194	<0.00001
	Upstream 300bp	51310	5	11335	<0.00001
	Upstream 500bp	55325	5	12284	<0.00001
	Upstream 1000bp	37301	5	7856	<0.00001
Gallus gallus	Exons	185061	5	9958	<0.00001
	Introns	154536	5	54337	<0.00001
	Intergenic	51538	5	30653	<0.00001
	Upstream 100bp	21107	5	3525	<0.00001
	Upstream 300bp	26846	5	6276	<0.00001
	Upstream 500bp	28262	5	7241	<0.00001
	Upstream 1000bp	21480	5	5596	<0.00001
Alligator mississippiensis	Exons	299789	5	23305	<0.00001
	Introns	126321	5	39153	<0.00001
	Intergenic	16353	5	5885	<0.00001
	Upstream 100bp	25335	5	5730	<0.00001
	Upstream 300bp	32480	5	10254	<0.00001
	Upstream 500bp	32879	5	11002	<0.00001
	Upstream 1000bp	32879	5	10902	<0.00001
Pelodiscus siniensis	Exons	165615	5	7938	<0.00001
	Introns	111804	5	16734	<0.00001
	Intergenic	17650	5	1737	<0.00001
	Upstream 100bp	5617	5	512	<0.00001
	Upstream 300bp	6807	5	828	<0.00001
	Upstream 500bp	6950	5	851	<0.00001
	Upstream 1000bp	5212	5	385	<0.00001
Chrysemys picta bellii	Exons	263318	5	8142	<0.00001
	Introns	125437	5	18065	<0.00001
	Intergenic	93715	5	12382	<0.00001
	Upstream 100bp	16837	5	2321	<0.00001
	Upstream 300bp	40790	5	6323	<0.00001
	Upstream 500bp	40997	5	6515	<0.00001
	Upstream 1000bp	28512	5	4588	<0.00001
Anolis carolinensis	Exons	179391	5	6246	<0.00001
	Introns	129776	5	22287	<0.00001
	Intergenic	25203	5	3941	<0.00001
	Upstream 100bp	18342	5	2557	<0.00001
	Upstream 300bp	24638	5	4103	<0.00001
	Upstream 500bp	26199	5	4012	<0.00001
	Upstream 1000bp	25301	5	2892	<0.00001
Sphenodon punctatus	Exons	119669	5	4105	<0.00001
	Introns	99562	5	7411	<0.00001
	Intergenic	8608	5	1374	<0.00001
	Upstream 100bp	12556	5	1547	<0.00001
	Upstream 300bp	16487	5	2028	<0.00001
	Upstream 500bp	17188	5	1784	<0.00001
	Upstream 1000bp	12558	5	750	<0.00001

9 EVOLUTION OF GENOMIC ORGANIZATION OF THE MHC

Yuanyuan Cheng*, Hilary Miller

*Correspondence to: yuanyuan.cheng@uq.edu.au

Genes of the major histocompatibility complex (MHC) play an important role in disease resistance and kin recognition and are among the most polymorphic in the vertebrate genome. Prior work[183] located the tuatara core MHC region to chromosome 13q, although a number of class I and class II genes were mapped to several other autosomes. Here, annotation of tuatara MHC regions and comparisons of gene organization to seven other representative species (Supplementary Information Figure 9.1) identified 56 genes spanning 13 scaffolds, including six class I genes, six class II genes, 19 class III genes, 18 framework genes, and seven extended class II genes. Four class I and four class II genes which are not linked to other MHC related genes were excluded from the comparative analysis, as they are likely located outside the core MHC region[183]. As shown in Supplementary Information Figure 9.1, the genomic organization of tuatara MHC is most similar to that of the anole, which we interpret as being typical of the Lepidosauria. Unlike the chicken MHC, which was proposed to represent the minimal essential MHC gene cluster[184], MHC of the tuatara, anole, alligator, and turtle all show a high gene content and complexity that is more similar to the MHC regions of amphibians and mammals. While the majority of genes annotated in the tuatara MHC are well conserved as one-to-one orthologs, extensive genomic rearrangements were observed among these distant lineages (Supplementary Information Figure 9.1). The most noteworthy rearrangement lies in the relative locations of genes in the class I and class III regions (as defined by their locations in the human MHC), with these two regions inverted between the mammalian and frog MHC, and the genes interspersed in the tuatara and anole genomes. This finding raises questions over the traditional categorization of these genes as class I or class III region genes, as evolutionarily there is no explicable difference between the two sets of genes in terms of gene clustering, organization, or function.

Prior work identified strong population differentiation at tuatara MHC class I genes, with population bottlenecks and isolation considered a larger influence in shaping this diversity than selection[185]. Further work using the expanded MHC gene set identified here might identify further population-level features important for the conservation of this species.

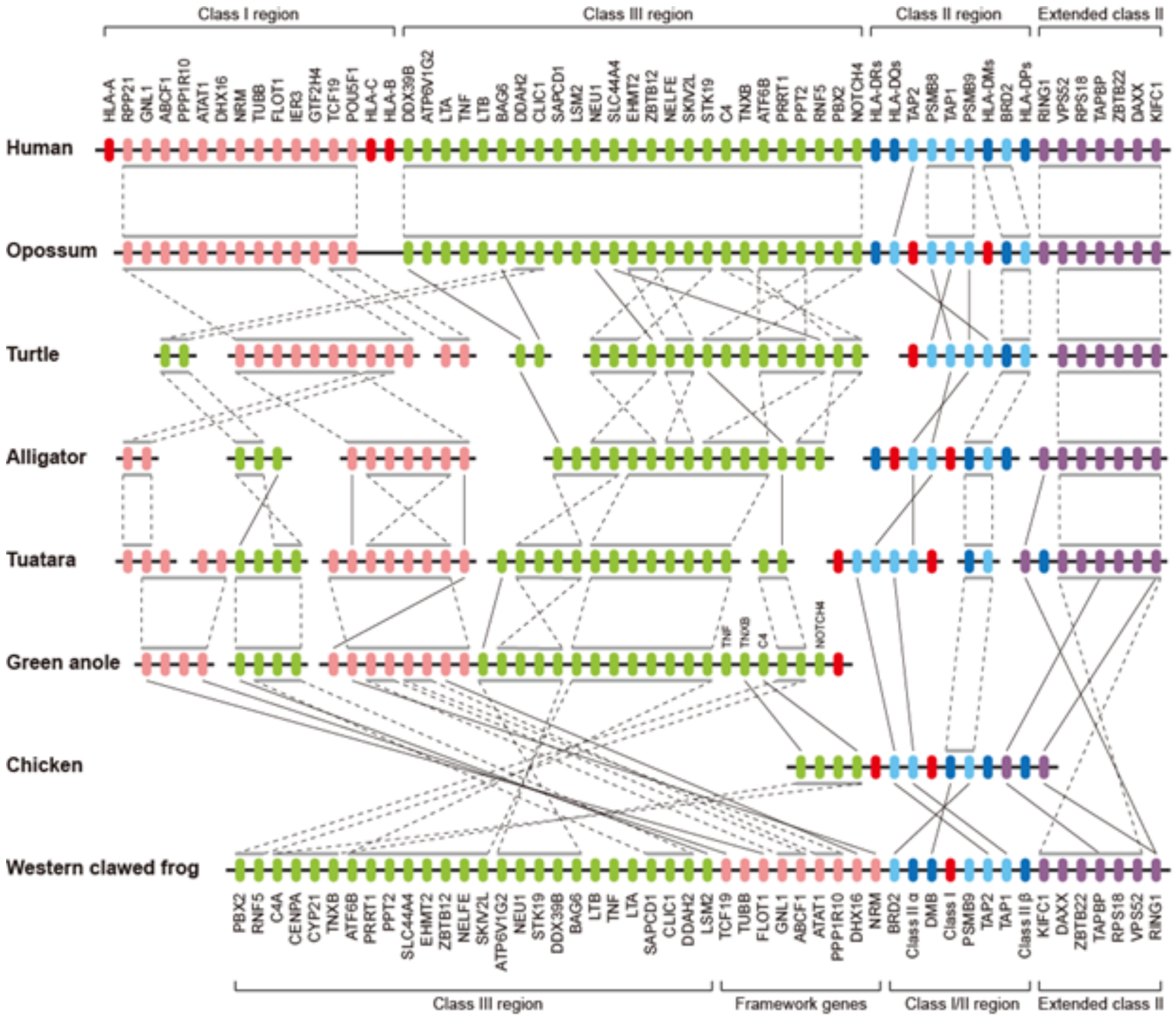

Supplementary Information Figure 9.1 Comparative analysis of the MHC core region. Species examined include human (assembly version GRCh38.p11), gray short-tailed opossum *Monodelphis domestica* (MonDom5), western painted turtle *Chrysemys picta bellii* (Chrysemys_picta_bellii-3.0.3), Chinese alligator *Alligator sinensis* (ASM45574v1), green anole *Anolis carolinensis* (AnoCar2.0), chicken *Gallus gallus* (Gallus_gallus-5.0), and western clawed frog *Xenopus tropicalis* (Xenopus_tropicalis_v9.1). Only genes that were annotated in the tuatara genome were included in the analysis. Orthologs between species are connected by a solid line; the grey bars above/below genes indicate syntenic blocks and are linked by dashed lines between species. Anolis class I/II and extended class II regions are not shown due to the high degree of genome assembly fragmentation in these regions. Colour legend: red – class I genes, pink – class I region framework genes, green – class III genes, dark blue – class II genes, light blue – class II region framework genes, purple – extended class II genes.

10 MOLECULAR EVOLUTION OF TUATARA VISUAL SYSTEM

Ryan K Schott*

*Correspondence to: schottr@si.edu

10.1 Introduction

The tuatara is primarily a nocturnal predator that is able to capture prey under extremely dim light conditions[186]. Vision appears to be the primary sensory system for prey capture: auditory and olfactory cues alone were insufficient for prey capture, but under even very dim light (down to 0.00615 lux), in the absence of other cues, tuatara were able to capture prey[186,187]. Despite this extreme nocturnal visual adaptation, the tuatara eye has an interesting mix of diurnal and nocturnal features including a fovea typical of diurnal species and a slit pupil found in many nocturnal species, as well as a distinct complement of photoreceptors[188].

Previous morphological analyses of the tuatara retina revealed four types of photoreceptors: large single rods, small single rods, double rods, and small single cones[188,189]. The small single cone did not have an oil droplet and was restricted to the periphery of the retina. Unusually, the rods were found to have colourless oil droplets, which are normally found in cones. Most vertebrates also only have a single type of rod, however, some geckos and snakes have multiple rod types, which are evolutionarily derived from cones through the process of photoreceptor transmutation[188,190,191]. Based on detailed ultrastructural analysis using electron microscopy, Meyer-Rochow and Ahnelt[192] found that the 'rods' of tuatara were also derived from cones. Unfortunately, the ultrastructure of the small single cone reported by Walls[188] was not analyzed. Furthermore, there have been no molecular studies of the tuatara visual system.

The tuatara genome provides a unique opportunity to uncover the molecular basis of these interesting morphological adaptations. Utilizing this resource, we analyzed the loss of visual genes in tuatara, evaluated visual pigment and photoreceptor complement, and tested for shifts in selective pressure in visual genes that may reflect the distinct visual adaptations in this enigmatic species.

10.2 Methods

To explore the molecular evolution of the tuatara visual system a set of 119 visual genes were targeted for analysis[193,194]. These were extracted from the *Sphenodon punctatus* genome assembly and CDS annotation using BLAST searches (blastn, discontiguous megablast, tblastn) using *Anolis* and *Chrysemys* sequence queries. Gene identifications were confirmed with reciprocal BLAST

and phylogenetic analysis, as necessary. Additionally, sequences were manually curated to ensure open reading frames.

Visual gene loss in tuatara was compared to that in other amniotes following the analysis of Perry et al.[194], which was updated to account for recent genome publications (most notably an updated platypus genome assembly[195]). Genes were initially searched for in the NCBI nucleotide database. If a sequence could not be found, BLAST was used as described above to search genome assemblies directly. Gene loss was inferred if a sequence could not be found in any of the available genomes for a particular lineage. Partial sequences were counted as present if they had an open reading frame.

Phototransduction genes were targeted for additional analyses to determine how selection has shaped the molecular evolution of the tuatara visual system. The phototransduction gene datasets of Schott et al.[196,197] were used with the addition of the tuatara sequences. Additionally, new datasets were built for SWS2 and SLC24A1 since these genes were not analyzed previously because they were lost in squamates (but not tuatara) following the methodology outlined in Schott et al.[196,197] .

To estimate the strength and form of selection acting on these genes we utilized the codon-based likelihood model implemented in PAML[198]. Specifically, we used the random sites models to estimate alignment-wide selection and Clade Model C (CmC [199]) to compare selective pressure in tuatara with other reptiles. Since geckos and snakes have previously been shown to be under different selective pressures, we evaluated whether tuatara had similar selective pressures as these groups by including tuatara in partitions with geckos, snakes, and both geckos and snakes, as outlined in Supplementary Information Figure 10.1. All analyses were run with varying starting values to avoid potential local optima. To determine significance, model pairs were compared using a likelihood ratio test (LRT) with a $\chi 2$ distribution, while non-nested models were evaluated using Akaike Information Criterion (AIC).

10.3 Results and discussion

10.3.1 Visual gene loss in tuatara and amniotes

Loss of visual genes in nocturnal animals is expected due to relaxed selective pressures, most often on genes involved in bright light vision. Indeed, previous analyses showed high levels of gene loss in groups that are hypothesized to have undergone strong nocturnal bottlenecks (mammals, snakes, geckos), while primarily nocturnal predators (such as crocodilians) showed moderate gene loss[194]. Surprisingly, we found that visual gene loss in tuatara was remarkably low compared to other major amniote lineages (Supplementary Information Figure 10.2, Table 10.1; Supplementary Data https://doi.org/10.5281/zenodo.2597599). Tuatara specifically lost only a single gene, TMT3, a nonvisual opsin, that was lost independently in mammals, snakes, and archosaurs (birds and crocodilians). Additionally, two genes (GNGT1 and PDE6A) were also absent in tuatara due to

their loss in the reptilian ancestor. The total loss of only three visual genes is the lowest among amniote lineages tied with diurnal squamates (e.g., anguids and iguanids).

10.3.2 Visual pigments and photoreceptors of tuatara

Morphological analyses of tuatara photoreceptors suggest that the three types of 'rods' (large, single, double) are actually evolutionarily derived from cones through photoreceptor transmutation[188,189,192]. However, this could not previously be confirmed from a molecular perspective. We found that tuatara possess the full complement of visual opsins (and therefore visual pigments) that were present in the vertebrate ancestor (RH1, RH2, LWS, SWS1, SWS2), as well as both the rod and cone phototransduction genes present in other reptiles. These genes are evolutionary conserved (see below) suggesting functioning rod and cone phototransduction pathways. This provides strong molecular support for photoreceptor transmutation in tuatara.

The multiple types of rod-like cone photoreceptors, which contain oil droplets, likely express cone visual pigments. If tuatara follows the typical vertebrate pattern this would place the long-wavelength sensitive (LWS) visual pigment in the large single 'rods' and double 'rods' and the other cone visual pigments (RH2, SWS2, SWS1) in subtypes of the small single 'rod' (Supplementary Information Figure 10.3). The small single (dwarf) cone is interesting as it lacks an oil droplet and is restricted to the periphery of the retina, features typical of rods. Most diurnal lizards lack morphological rods, but still express rod visual pigments and other rod phototransduction proteins[196], presumably in transmuted cone photoreceptors. If cone-like rods are the ancestral condition for squamates, this raises the possibility that the dwarf cone in tuatara is also a rod-like cone, homologous with those in squamates, and therefore would be the rod opsin (RH1)-bearing photoreceptor (Supplementary Information Figure 10.3). It is also possible that RH1 is instead expressed in one of the droplet-bearing rod-like cones. If this is the case then the dwarf cone likely instead contains the UV-sensitive SWS1 pigment, which is often the rarest photoreceptor type in other vertebrates. RH1 expression in a droplet-bearing cell would be unusual, but may be the case for the diurnal iguanid *Polychrus marmoratus*[200]. Unfortunately, the opsins underlying the photoreceptor types in squamates have not been confirmed, which makes inferring such patterns in tuatara difficult. Despite this, these results point to interesting patterns of evolution in the photoreceptors of tuatara and other lepidosaurs that warrant further study.

The five main classes of visual pigment (encoded by the visual opsins) each absorb light at a characteristic wavelength. Differences in the absorption between classes are due to differences in protein sequence at spectral tuning sites[201]. We analyzed the opsin sequences of tuatara at known spectral tuning sites to roughly estimate the wavelengths of maximal absorbance (λ_{max}) of the visual pigments. Overall, we found little variation at known sites, although it should be noted that spectral tuning is not well understood in non-avian reptiles and there are likely to be other sites that affect spectral tuning in tuatara that have not been accounted for here. In rod opsin (RH1) we found N83 in tuatara. This substitution causes a small blue-shift in many vertebrate RH1s and has also been implicated as a dim-light adaptation in several lineages that inhabit light-limited

environments[202–204]. Thus, tuatara RH1 may be further dim-light adapted and likely has a slightly blue-shifted λ_{max} around 490–495 nm. In the LWS opsin, we found that tuatara has A269, which results in a ~15 nm blue-shift in mammals[201], likely resulting in a tuatara LWS λ_{max} around 535–540 nm. The tuatara RH2, SWS2, and SWS1 pigments do not show any differences at known spectral tuning sites and these pigments thus likely had λ_{max}s around 500, 440, and 360 nm, respectively.

10.3.2.1 Selective pressures of tuatara phototransduction genes

Tuatara has a highly sensitive visual system and is able to capture prey under extremely low light conditions (down to 0.00615 lux[186]). This is achieved using rod-like photoreceptors that are evolutionarily derived, through photoreceptor transmutation, from cones. This process is expected to impose distinct selective pressures on the visual system. Previous work has shown that the phototransduction genes of caenophidian snakes and geckos underwent a long-term shift in selective pressure associated with this process of photoreceptor transmutation[196]. We tested for a similar shift in tuatara phototransduction genes.

Overall, tuatara phototransduction genes do not show a significant long-term shift in selective pressures. In only five of the 33 genes was tuatara included in the best-fitting partition (Supplementary Information Table 10.2; Supplementary Data DOI: 10.5281/zenodo.2597599). This included two rod genes (*CNGB1*, *PDE6B*) and three cone genes (*CNGA3*, *GNGT2*, and *PDE6H*). Interestingly there does seem to be a focus in both the rod and cone genes on the cyclic nucleotide gated channels (CNGs) and phosphodiesterase (PDE), although it is not clear if this could point to a functional adaptation towards dim-light vision. The low number of phototransduction genes under distinct selective pressures in tuatara is in contrast to caenophidians being in the best fitting partition for 25 of the 29 genes and geckos for 20 of the 25 genes present in these lineages. These results suggest that photoreceptor transmutation did not impose similar selective pressures in tuatara as in snakes and geckos.

10.3.2.2 Evolution of the tuatara visual system

The results presented here provide a novel molecular perspective on the evolution of the tuatara visual system that corroborates the morphological data that tuatara possess a unique mixture of diurnal and nocturnal adaptations. Despite substantial dim-light adaptation and rod-like cone photoreceptors, the tuatara visual system is highly conserved with the tuatara lineage having one of the lowest rates of gene loss of any amniote and highly conserved phototransduction genes. The maintenance of five visual opsins suggests that tuatara has a robust colour vision system that, due to the presence of rod-like cones, may function in dim-light conditions, similar to nocturnal geckos, which also have rod-like cones[205]. Geckos only have 3 visual pigments, however, implying that tuatara nocturnal colour vision could have superior colour discrimination. Both geckos and snakes showed concerted, long-term shifts in selective pressure on phototransduction genes associated with photoreceptor transmutation. Although tuatara also has strong evidence for photoreceptor transmutation we did not recover a similar selective pattern in this lineage. This suggests a distinct

path to nocturnal adaptation in tuatara that is perhaps constrained by its unusual life history: juvenile tuatara are often diurnal and arboreal to avoid predation by cannibalistic adults, while adults hunt primarily at night, but also bask during the day[206]. This dichotomous selective pressure likely contributed strongly to the unique visual system of the tuatara.

10.4 Figures

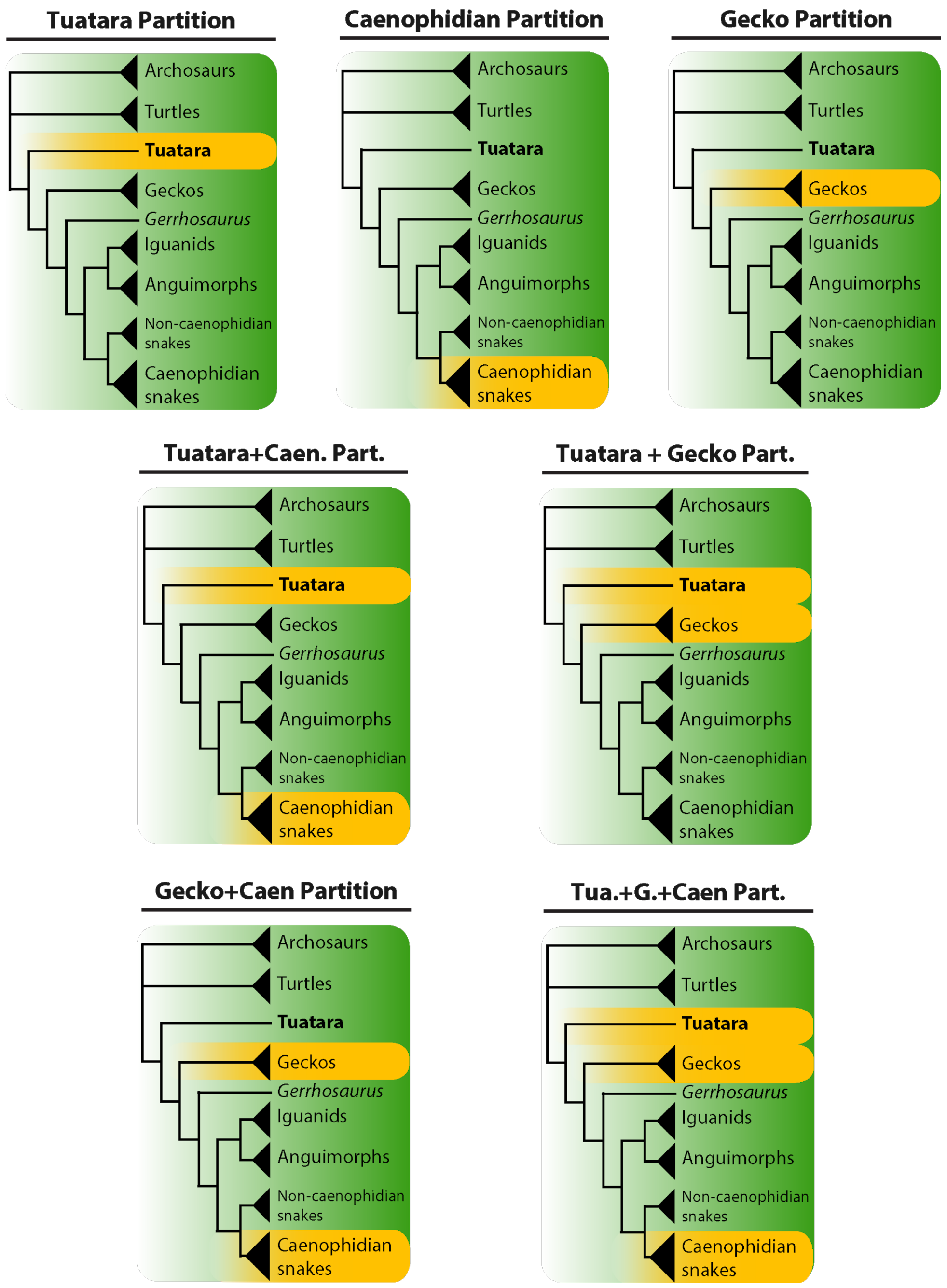

Supplementary Information Figure 10.1. Clade partitioning schemes used to test for long-term shifts in selective pressures in tuatara phototransduction genes. The clade/lineage highlighted in yellow was placed into a separate partition from the rest of the tree allowing independent estimates of d_N/d_S in the divergent site class for the two partitions.

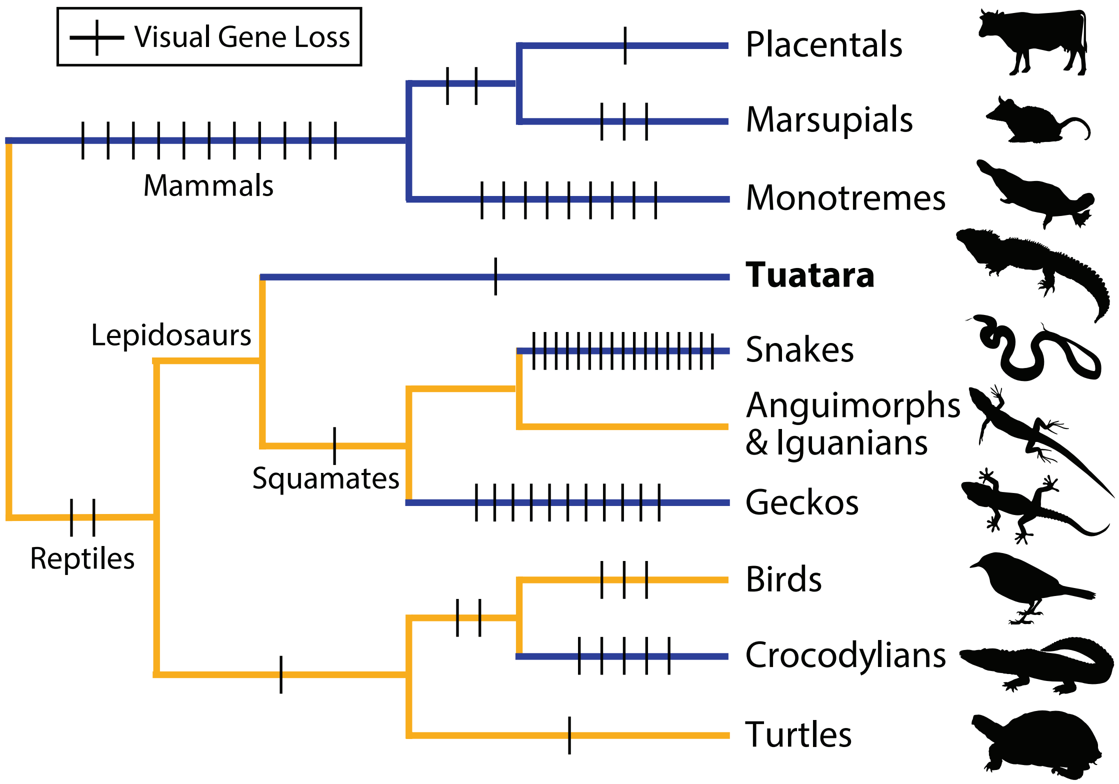

Supplementary Information 10.2. Phylogenetic tree of amniotes depicting inferred visual gene losses. Lineages are coloured based on a rough approximation of their ancestral activity pattern (blue, nocturnal; yellow, diurnal). Note that the tuatara lineage has experienced some of the lowest rates of gene loss despite a nocturnal ancestry, which in other lineages is associated with increased gene loss. See Supplementary Information Table 10.1 for a summary of genes lost per branch. The complete table of visual genes analyzed and their presence or absence from the various groups is available here at DOI: 10.5281/zenodo.2597599. Silhouettes "Designed by Creazilla" www.creazilla.com

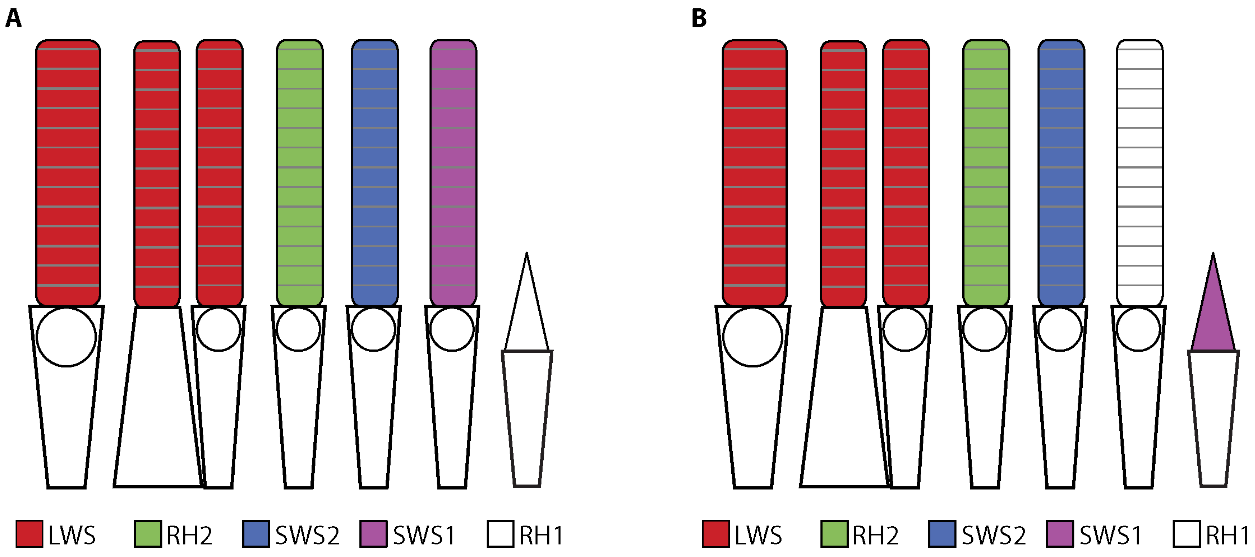

Supplementary Information 10.3. Schematic of photoreceptors types of tuatara showing two potential distributions of visual pigments. Tuatara has three morphological types of rod-like cones and one dwarf cone [189], and has four cone opsins (LWS, RH2, SWS2, and SWS1) and one rod opsin (RH1). The dwarf cone lacks an oil droplet and is absent from the fovea and central retina making it a strong candidate for the rod opsin (RH1)-bearing photoreceptor, perhaps homologous with the rod opsin-bearing cones of diurnal lizards (A). Alternatively RH1 may be present in a subtype of the rod-like cone, making the dwarf cone most likely to contain a UV-sensitive SWS1, which is often the rarest photoreceptor type.

10.5 Tables

Supplementary Information Table 10.1. Summary of genes inferred to be specifically lost along each branch of the phylogenetic tree of amniotes depicted in Supplementary Information 10.2. The complete table of visual genes analyzed and their presence or absence from the various groups is available at DOI: 10.5281/zenodo.2597599.

Branch	Number of Specific Inferred Losses (Total Losses)	Specific Genes Inferred to be Lost
**Reptiles**	**2 (2)**	***GNGT1, PDE6A***
**Archelosaur**	**1 (3)**	***Parietopsin***
**Turtles**	**1 (4)**	***GUCY2F***
**Archosaurs**	**2 (5)**	***GNB2, TMT3***
**Crocodilians**	**5 (10)**	***NEUR3, Parapinopsin, RH2, SWS1, TMT2***
**Birds**	**3 (8)**	***ARBB2, NRL, PPP2R1A,***
**Lepidosaurs**	**0 (2)**	**-**
**Tuatara**	**1 (3)**	***TMT3***
**Squamates**	**1 (3)**	***SLC24A1***
**Anguimorphs & Iguanians**	**0 (3)**	**-**

**Snakes**	**17 (20)**	***CRYBA1, CRYBA4, CRYBB1, CRYBB3, CRYGN, GRK1, NEUR2, NEUR3, OPN4m, Parapinopsin, Parietopsin, Pinopsin, RH2, SWS2, TMT1, TMT2, TMT3***
**Geckos**	**11 (14)**	***CNGA1, CRYGN, NEUR2, NEUR3, OPN4m, Parapinopsin, Parietopsin, PDE6B, PDE6G, RH1, SWS2,***
**Mammals**	**11 (11)**	***NEUR2, NEUR3, NEUR5, OPN4x, Parapinopsin, Parietopsin, Pinopsin, RH2, TMT1, TMT3, VAOP***
**Monotremes**	**9 (20)**	***CNGA3, CRYBA4, CRYBB1, GNGT2, GUCY2F, OPN3, RGS9BP, RP1L1, SWS1***
**Therians**	**2 (13)**	***NEUR4, SWS2***
**Marsupials**	**3 (16)**	***GUCA1B, GUCY2F, RGR***
**Placentals**	**1 (14)**	***TMT2***

Supplementary Information Table 10.2. Summary of analyses comparing selective pressures in tuatara with those in reptiles, geckos and caenophidian snakes. The best fitting partition (Supplementary Information 10.1) is shown for each gene (when significant vs the null model, M2a_rel). Full results tables are presented in the Supplementary Data (DOI: 10.5281/zenodo.2597599). Abbreviations: **C**, caenophidian partition; **G**, gecko partition; **T**, tuatara partition; **GC**, gecko plus caenophidian partitions; **TC**, tuatara plus caenophidian partition; **TG**, tuatara plus gecko partition; **TGC**, tuatara plus gecko plus caenophidian partition.

**Type**	**Gene**	**Best-fitting Partition**
Rod	*CNGA1*	-
Rod	*CNGB1*	**TGC**
Rod	*GNAT1*	GC
Rod	*GNB1*	-
Rod	*GRK1*	G
Rod	*PDE6B*	**TC**
Rod	*PDE6G*	C
Rod	*RH1*	C
Rod	*SAG*	GC
Rod	*SLC24A1*	-
Cone	*ARR3*	GC
Cone	*CNGA3*	**TGC**
Cone	*CNGB3*	GC
Cone	*GNAT2*	GC
Cone	*GNB3*	GC
Cone	*GNGT2*	**TGC**
Cone	*GRK7*	GC
Cone	*GUCA1C*	G

Cone	*LWS*	C
Cone	*PDE6C*	GC
Cone	*PDE6H*	**TG**
Cone	*RH2*	G
Cone	*SLC24A2*	GC
Cone	*SWS1*	C
Cone	*SWS2*	-
Both	*GNB5*	GC
Both	*GUCA1A*	C
Both	*GUCA1B*	C
Both	*GUCY2D*	GC
Both	*GUCY2F*	C
Both	*RCVRN*	C
Both	*RGS9*	GC
Both	*RGS9BP*	GC

11 ODORANT RECEPTORS

Melissa D. Jordan and Richard D. Newcomb

Correspondence to: Melissa.Jordan@plantandfood.co.nz; Richard.Newcomb@plantandfood.co.nz

11.1 Methods

Odorant receptor sequences from the green anole (*Anolis carolinensis*) and Burmese python were used as query sequences for tBLASTn[69] searches of the tuatara genome using Geneious 10.0.3 (https://www.geneious.com). Iterative searches were performed using newly identified tuatara ORs to ensure a comprehensive set of genes was attained. Genes were classified in the following way: intact genes consisted of an uninterrupted open reading frame >300 amino acids with an appropriate start and stop codon, ORs were considered pseudogenes if they contained interrupting stop codons or frameshifts. Translated amino acid sequences for phylogenetic analysis were obtained from manually annotated published data for *Gallus gallus* and *Taeniopygia guttata*[207] or from automatic annotation via NCBI for *Anolis carolinesis*, *Gekko japonicas*, *Notechis scutatus*, *Ophiophagus hannah*, *Pogona vitticeps*, *Protobothrops mucrosquamatus*, *Pseudonaja textilis*, *Python bivittatus* and *Thamnophis sirtalis*. Only sequences greater than 280 amino acids in length were used in the analysis. Multiple sequence alignment was performed using FFT-NS-1 parameters in MAFFT (v7.338)[150]. The alignment was trimmed such that any columns containing more than 75% gaps were removed, which resulted in an alignment consisting of 322 characters. Phylogenetic relationships were estimated using the Neighbor-Joining method with Poisson substitution model and 1000 bootstrap replicates within MEGA 7.0.21[208] and the tree drawn and edited using Figtree v1.4.4 (https://tree.bio.ed.ac.uk/software/figtree).

11.2 Results

Vertebrate ORs are G protein-coupled receptors that contain seven transmembrane domains[209]. They are expressed in the membrane of olfactory neurons within the olfactory epithelium where odorants are detected by extracellular regions of the receptor. Binding of an odorant to its corresponding receptor initiates a signal transduction pathway which ultimately leads to transmission of the action potential to the olfactory bulb[210]. Four hundred and seventy two genes encoding predicted odorant receptors (ORs) were identified from the genome of tuatara (10.5281/zenodo.2592798). Of these, 62 genes were identified as pseudogenes, containing one to multiple stop codons or frameshifts within their sequence. The percentage of intact genes was therefore 72%, which is perhaps a slight underestimate as some of the 60 partial sequences are likely to represent intact genes.

Vertebrate ORs have a complex classification but can generally be split into two types, Type I and Type II. Type I can be subdivided into further groupings (alpha-zeta)[211,212] and also encompasses the mammalian OR classification groupings of Class II (gamma) and Class I (alpha and beta)[213]. Class I ORs are thought to be more specialised towards detecting water soluble odorants and correspondingly all fish ORs fall into Class I. Class II ORs recognise airborne odorants.

Phylogenetic analyses were undertaken to compare ORs from tuatara with those from other terrestrial Sauropsids, including snakes (Burmese python, brown snake, garter snake, king cobra, pit viper and tiger snake), lizards (bearded dragon, green anole and Japanese gecko) and birds (chicken and zebra finch). Tuatara ORs are predominantly members of the Class II gamma group, with a small number of Class I ORs corresponding to the alpha group (Supplementary Information 11.1). Within the gamma group tuatara have an extensively radiated clade containing 65 ORs that is sister to a much larger equivalent set of radiated ORs in the birds (gamma-c group[207]). The presence of tuatara ORs across the tree indicates that the origin of the major OR lineages predates the split of birds, lizards and tuatara and likely evolved rapidly via duplication and divergence from a small number of Class II ORs in fish (one in zebrafish) and then amphibians[211,212]. The tuatara genome demonstrates that this rapid expansion of ORs that recognise airborne odorants occurred across all the major lineages within the Sauropsida.

Supplementary Information Figure 11.1 The evolutionary history of terrestrial Sauropsid odorant receptors inferred using the neighbor-joining method. The unrooted tree contains 3213 amino acid sequences. Branches are coloured according to the following categories: Green – tuatara, Blue – birds (*Gallus gallus*, *Taeniopygia guttata*), Red – snakes (*Notechis scutatus*, *Ophiophagus Hannah, Protobothrops mucrosquamatus*, *Pseudonaja textilis*, *Python bivittatus* and *Thamnophis sirtalis*), Orange – lizards (*Anolis carolinesis, Pogona vitticeps*) and Purple – gecko (*Gekko japonicas*). Bootstrap support values above 75% (1000 replicates) are indicated for major branch splits relating to the different OR groups and branches leading to the species-specific OR expansions in birds (group γ-c) and tuatara (*).

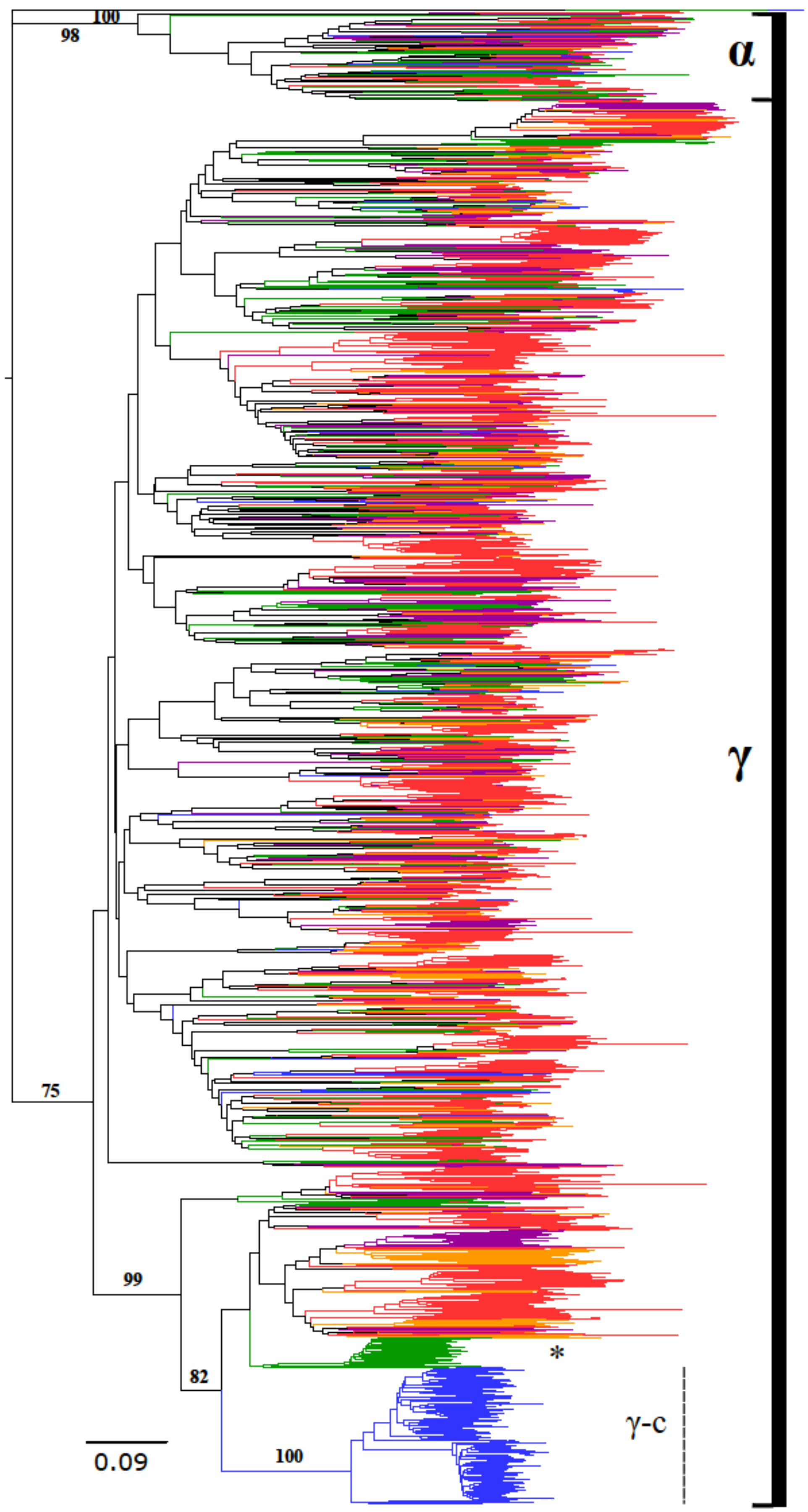

100
98
α
γ
75
99
82
*
γ-c
100
0.09

12 TRANSIENT RECEPTOR POTENTIAL (TRP) ION CHANNELS GENES IN SPHENODON PUNCTATUS

José Ignacio Arroyo

Correspondence to: jiarroyo@uc.cl

12.1 Background

Tuatara is a behavioural thermoregulator. The Transient Receptor Potential (TRP) ion channels genes play an important role in thermoregulation as some of these genes participate in thermosensation and cardiovascular physiology. To search for the genomic adaptations related to thermoregulation in tuatara we performed a comparative genomic analysis of TRP genes in tuatara and six other species of amniotes. Duplicated thermo- and non-thermosensitive TRP genes were identified in tuatara that are differentially retained in this species, including members of most of the TRP subfamilies. A signature of positive selection in heat-sensitive TRP genes was observed when comparing orthologous genes as well as when comparing recently duplicated paralogous genes within Sphenodon.

Gene duplication is a fundamental mechanism of genomic innovation. After duplication paralogous genes can diverge under the action of positive selection and be differentially retained among descending lineages[214]. Both these processes, gene divergence and differential retention, can be potentially related to changes in the traits of species including their reproduction and survival. One of the fundamental traits of species is their ability to maintain their body temperature within certain ranges, a task that they can perform physiologically (endotherms) or behaviourally as is the case of ectotherms[215].

Tuatara (*Sphenodon punctatus*) are behavioural thermoregulators[216–218], meaning that in order to regulate their body temperature they move through their environment to find optimal temperature habitats. Temperature is a crucial environmental variable that affects physiological processes, life history traits and fitness[219]. In behavioural thermoregulation, thermosensation of the environment is a critical step. From a molecular standpoint, thermosensation is controlled by Transient Receptor Potential (TRP) ion channels[220]. TRPs are cold/heat-gated ion channels that have a long length, all of them including at least six putative transmembrane domains. For instance, human TRP genes are diverse in length and range between 11.4 and about 911 kb, with the number of exons varying from 11 to 39[221]. The TRP family includes seven well-recognized subfamilies: TRPA1, TRPV, TRPML, TRPM, TRPC, TRPP (also called PKD) and TRPN[222,223]. Most of these genes are dispersed in vertebrate genomes despite some of them being tandemly arranged, likely due to being derived from small-scale duplication events. For instance, in the human genome TRPM3 and -6 are in chromosome 9, TRPV1–3 are in tandem in chromosome 17, TRPV5–6 are in chromosome 7 and TRPML2–3 are in chromosome 1, whereas all the remaining members are dispersed in

different chromosomes in the genome[221]. The repertoire of these genes varies among species. This variation has been relatively well studied in the model species in Bilateria. For instance, Drosophila possess 17 TRP genes and humans have 27[221]. Within chordates there is lower variation but it remains not well studied and mostly limited to model species. Among TRPs, TRPA1, TRPM and TRPV are thermosensitive (thermoTRPs)[224–226], which detect from cold (i.e. TRPV1) to hot (i.e. TRPM8) stimuli[221]. TRP genes participate in thermoregulation, which is critical for reproduction and survival in ectotherms such as reptiles, thus it seems probable that these genes could have been a target of natural selection whether on specific sites or on the divergence of duplicated genes. For instance, it has been shown that the inhibition of cold- and heat-sensitive TRP channels in the saltwater crocodile produces alterations in thermoregulation[227].

12.2 Methods

Here, we performed a comparative genomic analysis of TRPs in tuatara and six representative species of amniotes in order to characterize the evolutionary history of this gene family in this species, including the variation in the repertoire of genes due to duplication and subsequent differential retention and the rates of molecular evolution on orthologous and paralogous genes (divergence). To accomplish this goal, protein sequences for the seven TRP subfamilies were obtained from the Ensembl database for four representative amniote species covering the main lineages: human (*Homo sapiens*), chicken (*Gallus gallus*), lizard (*Anolis carolinensis*) and turtle (*Pelodiscus sinensis*). To annotate TRP genes from draft genomes of viper (*Vipera berus*), alligator (*Alligator mississippiensis*) and tuatara (*Sphenodon punctatus*) we used as a reference human TRP protein sequences that were aligned against draft genomes using tblastn[69]. We only included sequence matches with a cover and identity >30%. New identified genes were named in agreement to their phylogenetic relationships. Alignment was performed using MAFFT (strategy L-INS-i[150]). Phylogenetic relationships were estimated using the approximately maximum likelihood approach of FastTree2[98] as implemented in the CIPRES portal[115]. The tree was rooted with VDAC which are also voltage-gated ion channels (VGIC), which yields a topology consistent with previous studies[222]. All data are detailed in TreeBASE submission ID 23409.

12.3 Results and discussion

We found that the repertoire of TRP genes varied among the species surveyed between 28–37 genes (Supplementary Information Table 12.1). Tuatara has the largest repertoire of TRP genes among the species surveyed. We identified 37 TRP-like sequences in the tuatara genome draft, but because TRPs are long genes, most identified genes corresponded to partial sequences (coverage of recovered alignments with human orthologs varied from 40–100%). Nonetheless for five genes (TRPV1-3, TRPM1-2) we recovered complete or (five cases of) almost complete genes (>90% of coverage to human orthologs. Most genes were recovered in separate scaffolds, except for some

cases where it was possible to recover tandemly arranged TRP sequences (Supplementary Information Table 12.1). These genes included partial sequences of TRPN-like genes (ScrUdWx_1447), TRPML2–3 (ScrUdWx_1600), TRPV1–3 (contig ScrUdWx_1875), TRPN and TRPC (ScrUdWx_55) and a pair of PKD1L3s (ScrUdWx_67_1, ScrUdWx_67_2) (Supplementary Information Table 12.1).

The phylogenetic reconstruction recovered well-supported monophylies for each of the seven subfamilies (Supplementary Information 12.1; most of them >0.8) and well supported monophylies of each of the respective paralogs within each subfamily (Supplementary Information 12.1; supports not shown). The TRPP and TRPML clades were recovered in one clade and the -V, -C, -N, -A and -M in a separate clade (Supplementary Information Figure 12.1). Relationships among subfamilies were consistent with previous studies[221].

A detailed examination of the topologies of the TRP subfamilies revealed repeated gene duplications followed by extensive loss and differential retention of genes within amniotes (Supplementary Information Figure 12.2). These duplicates include new (not previously reported) genes in tuatara (and other amniotes), including TRPV1/2/4, TRPC3/5/7, TRPN-2, TRPN-4, TRPN-5, PKD1-2, PKDREJ, PKDL13-2 and TRPML3 (asterisks in Supplementary Information Figure 12.1, 12.2). Some duplicates appear to be old as they were recovered as sisters of other subfamilies as is the case of TRPV1/2/4, TRPC3/5/7, while other duplications seem more recent originating in the common ancestor of reptiles or being specific to a single species. This analysis shows that the evolutionary history of these genes has been more dynamic than previously shown for some model species.

We explored variation in ω (dN/dS), the ratio of the rate of non-synonymous substitutions (dN) to the rate of synonymous substitutions (dS), in a maximum likelihood framework using the codeml program from PAML v4.4[198]. In brief, $\omega \approx 1$ indicates neutrality, $\omega < 1$ indicates negative or purifying selection and $\omega > 1$ indicates positive selection. Branch-site models were implemented to explore changes in ω across codons in a specific branch of the tree[228]. It only included sequences with a coverage >90%. In this case the branch of the tuatara was labelled as the foreground branch and non-tuatara branches were labelled as background. The Bayes Empirical Bayes (BEB) method was used to identify sites under positive selection[229,230]. The modified model A[231,232] with a ω free to vary was compared with a model with a ω fixed to 1, representing neutrality. The model estimates the proportion of four types of site classes (p_0, p_1, p_{2a} and p_{2b}) and their respective omega values (ω_0, ω_1, ω_{2a} and ω_{2b}). Site class 0 includes codons that are conserved throughout the tree, with $0<\omega 0<1$ estimated. Site class 1 includes codons that are evolving neutrally throughout the tree with $\omega 1=1$. Site classes 2a and 2b include codons that are conserved or neutral on the background branches, but become under positive selection on the foreground branches with $\omega_2>1$.

A branch-site analysis of molecular evolutionary rates among thermosensitive genes (testing only the five sequences with a coverage >90%; TRPV1–3, TRPM1–2) found significant positive

selection in the heat-sensitive genes TRPM2 ($2\Delta lnL=3.18$, p-value=0.07, $p_2=0.014$, $\omega_2=4.66$) and TRPV2 ($2\Delta lnL=12.04$, p-value=0.0005, $p_2=0.071$, $\omega_2=9.29$).

In addition, to explore selection in genes where it was not possible to obtain complete coverage we used a pairwise comparative approach to estimate dN/dS rates as implemented in the program yn00[230]. In this approach we compared *Sphenodon* genes with TRP genes of *Anolis*, any other species that shared differentially retained genes with *Sphenodon* and also compared duplicated paralogous genes within *Sphenodon*. Among the 21 pairwise comparisons, all of them have an $\omega<1$ and in three cases there were moderately accelerated rates of ω (>0.5), particularly among comparisons of partial TRPN sequences (Supplementary Information Figure 12.3).

In general these results show a high rate of gene differential retention and positive selection in genes for which a function in heat sensation is well established[226,233]. Given the role of these genes, it seems probable that these genomic changes in TRP genes are associated with the evolution of thermoregulation in this species. Duplications in non-thermosensitive TRP genes that participate in cardiovascular physiology also appear to play a role in thermoregulation (see[220,222]). While the function of these genes have not been specifically studied in tuatara, function has been studied in other species. For instance PKD1 is an integral membrane protein involved in cell–cell/matrix interactions[234], PKD1L3 participates in taste reception[235], TRPN has roles in proprioreception and hearing[236,237].

12.4 Figures

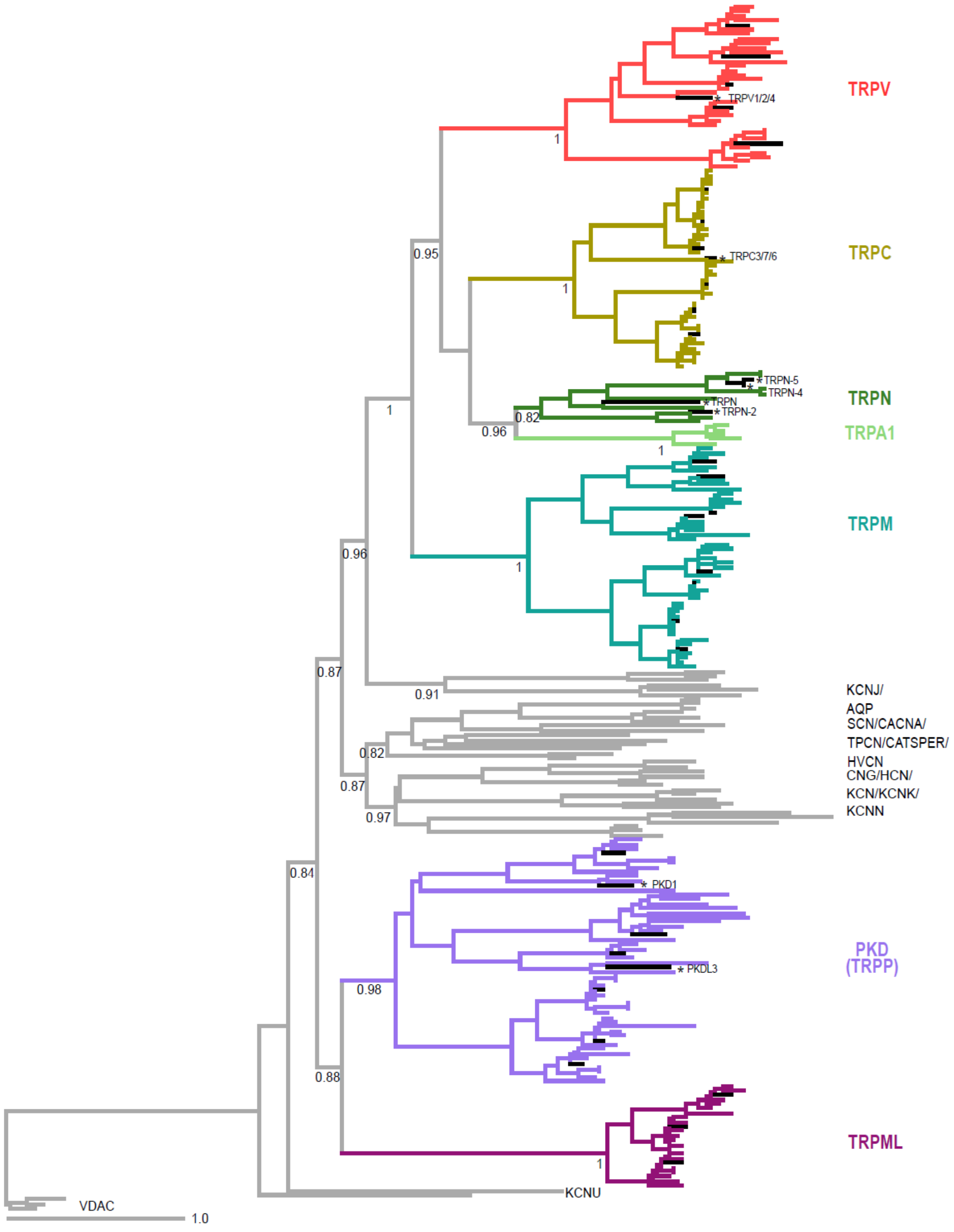
TRPV
TRPV1/2/4
1
TRPC
TRPC3/7/6
0.95
1
TRPN-5
TRPN-4
TRPN
TRPN-2
TRPN
0.82
0.96
TRPA1
1
1
TRPM
0.96
1
0.87
0.91
KCNJ/
AQP
SCN/CACNA/
TPCN/CATSPER/
HVCN
CNG/HCN/
KCN/KCNK/
KCNN
0.82
0.87
0.97
0.84
PKD1
PKD
(TRPP)
PKDL3
0.98
0.88
TRPML
1
KCNU
VDAC
1.0

Supplementary Information Figure 12.1. Phylogenetic relationships among TRP ion channels genes in amniotes. Blackened terminal branches denote tuatara branches. Node supports correspond to local bootstrap support values derived from 1,000 pseudoreplicates, as implemented in FastTree2 (only relevant nodes are showed). Asterisks indicate TRPs differentially retained in tuatara. The differential retention of genes is characterized in a phylogenetic tree because after their evolutionary origin they are lost in most species but are retained only in a few, as is the case of the new genes characterized in tuatara. These new genes (not previously reported) include: (from top to bottom) TRPV1/2/4, TRPC3/5/7, TRPN-2, TRPN-4, TRPN-5, PKD1-2 and PKDL3-2. These new genes were named according to their phylogenetic affiliations. For instance, the gene TRPV1/2/4 retained only in tuatara and turtle is sister to the genes TRPV1, TRPV2 and TRPV4. Other voltage-gated ion channels and voltage-gated ion channels (e.g. CATSPER, AQP) were also included in the phylogenetic reconstruction. The tree was rooted with the voltage-gated non-TRP human VDAC genes.

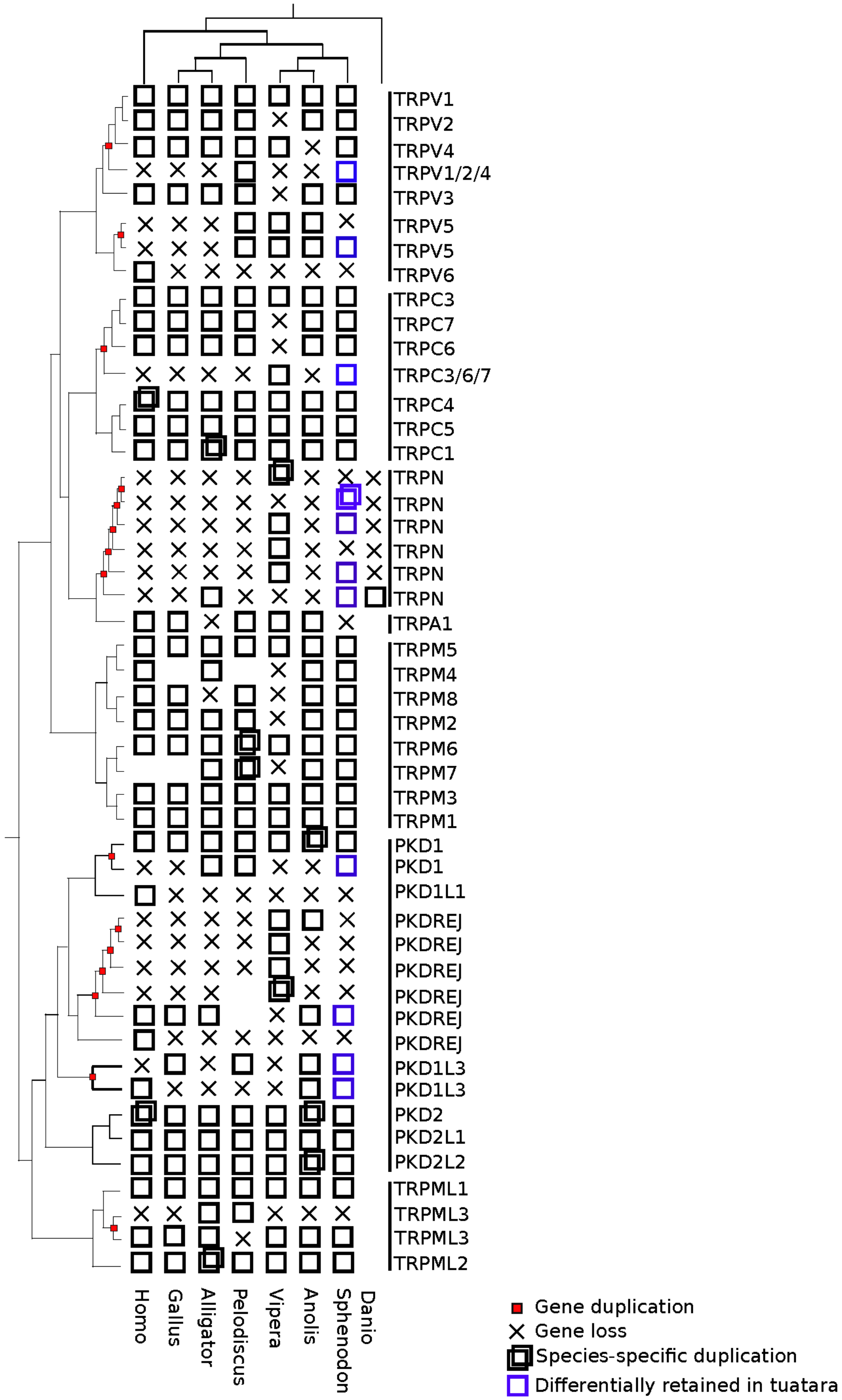
TRPV1
TRPV2
TRPV4
TRPV1/2/4
TRPV3
TRPV5
TRPV5
TRPV6
TRPC3
TRPC7
TRPC6
TRPC3/6/7
TRPC4
TRPC5
TRPC1
TRPN
TRPN
TRPN
TRPN
TRPN
TRPN
TRPA1
TRPM5
TRPM4
TRPM8
TRPM2
TRPM6
TRPM7
TRPM3
TRPM1
PKD1
PKD1
PKD1L1
PKDREJ
PKDREJ
PKDREJ
PKDREJ
PKDREJ
PKDREJ
PKD1L3
PKD1L3
PKD2
PKD2L1
PKD2L2
TRPML1
TRPML3
TRPML3
TRPML2
Homo
Gallus
Alligator
Pelodiscus
Vipera
Anolis
Sphenodon
Danio
Gene duplication
Gene loss
Species-specific duplication
Differentially retained in tuatara

Supplementary Information Figure 12.2. Repertoire of TRP genes among *Sphenodon punctatus* and other six vertebrate species. The repertoire of TRP genes identified in tuatara (*Sphenodon punctatus)* and other six vertebrate species: lizard (*Anolis carolinensis*), viper (*Vipera berus*), turtle (*Pelodiscus sinensis*), alligator (*Alligator mississippiensis*), chicken (*Gallus gallus*), and human (*Homo sapiens*). Small red squares on nodes indicate gene duplications, duplicated boxes indicate species-specific duplications, and blue boxes indicate differential gene retention in tuatara. Crosses indicate genes lost after duplication and empty spaces genes most likely lost, but not yet confirmed.

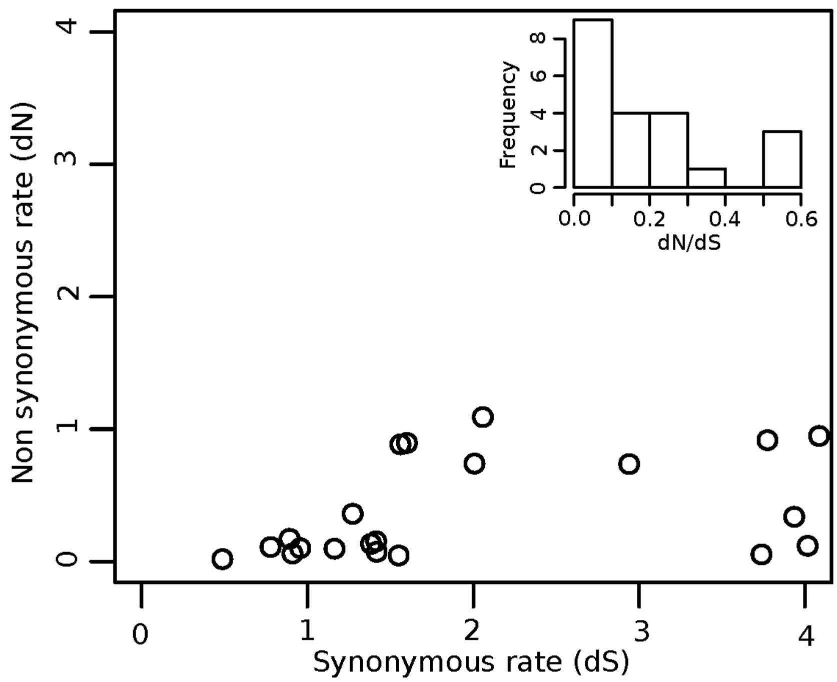

Supplementary Information Figure 12.3. Pairwise comparisons of dN-dS rates of TRP genes between *Sphenodon* and *Anolis* and between *Sphenodon* duplicates. (Right upper corner) distribution of Omega values.

12.5 Tables

Supplementary Information Table 12.1. Genomic composition of TRP genes identified in *Sphenodon punctatus* and other six species of vertebrates.

Species	ML	P(PKD)	M	A	N	C	V
Anolis	3	8	8	1	NF	6	6
Vipera	4	10	3	1	6	3	4
Sphenodon	3	8	8	NF	5	7	6
Alligator	5	6	8	NF	1	9	4
Gallus	3	6	7	1	NF	6	5
Pelodiscus	3	7	9	1	NF	7	8
Homo	3	7	7	1	NF	6	6
Contig (ScrUdWx)*							
1447					p-p		
1600	2-3						
1875							1-2-3
550					p-3		
67_1, 67_2		1L3-1L3-2					

NF: Not Found. *Here we show the TRP genes of Sphenodon that were found in tandem in different contigs. For instance, in contig ScrUdWx_1600 there were found in tandem genes TRPML2-TRPML3.

13 SELENOPROTEINS

Didac Santesmasses, Marco Mariotti and Roderic Guigó*

*Correspondence to: roderic.guigo@crg.cat

13.1 The tuatara selenoproteome

Selenoproteins incorporate selenium in the form of selenocysteine (Sec), the 21st amino acid. The human genome encodes 25 selenoproteins whose roles include antioxidation, redox regulation, thyroid hormone synthesis, calcium signal transduction and others[238]. Sec is inserted in response to a UGA codon, normally a stop codon, within selenoprotein mRNAs. UGA recoding is directed by an RNA secondary structure termed SECIS located in the 3'UTR of eukaryotic selenoprotein mRNAs, and requires a dedicated machinery[238]. We identified 26 selenoprotein genes in the tuatara genome (Supplementary Information Table 13.1), along with the machinery genes for their synthesis (Supplementary Information Table 13.2), using Selenoprofiles[239]. The number of selenoproteins, and the protein families they belong to, broadly fit with our expectations according to other vertebrate genomes previously analyzed[240]. One unexpected finding was the presence of two selenoproteins from the SELENOW (previously SelW) family. SELENOW is a short protein with unknown function that has a thioredoxin domain (Rdx family, PF10262). Two SELENOW selenoproteins, SELENOW1 and SELENOW2, were predicted to be present in the last common ancestor of vertebrates, though most genomes have only one of the two genes[240]. SELENOW2 was predicted to have been lost in the amniote stem (sauropsids and mammals). The presence of SELENOW2 in tuatara, and in other Lepidosauria genomes analyzed here, suggests that the loss of SELENOW2 must have occurred later in evolution, after the split of sauria and mammals. We could identify the SECIS element only in the SELENOW1 gene, but we detected the two genes in the transcriptome assembly, as evidence for their expression.

13.2 Multiple tRNASec gene copies

The selenocysteine specific tRNA ($tRNA^{Sec}$) plays a central role in both biosynthesis and incorporation of Sec[238]. We identified four tRNA-Sec genes in the tuatara genome using Secmarker[241]. Other Lepidosauria genomes analyzed here had, instead, only a single $tRNA^{Sec}$. Therefore, the three extra copies, which were found in the same scaffold within a ~5 kb region, may be specific to tuatara. There is generally a single functional copy of $tRNA^{Sec}$ in genomes[238], and when additional copies are present they show characteristics of non-functional pseudogenes[241]. In tuatara, all extra copies conserved the same predicted secondary structure, but possessed a compensatory mutation in the acceptor stem and other mutations that were not expected to disrupt the pairing potential. Additionally, the upstream promoter elements necessary for $tRNA^{Sec}$ transcription[242] were present in all four $tRNA^{Sec}$ (Supplementary Information Figure 13.1). These

external promoter elements are not transcribed[243], hence none of the extra tRNA^{Sec} copies appeared by retrotransposition. These observations suggest all four genes might be functional.

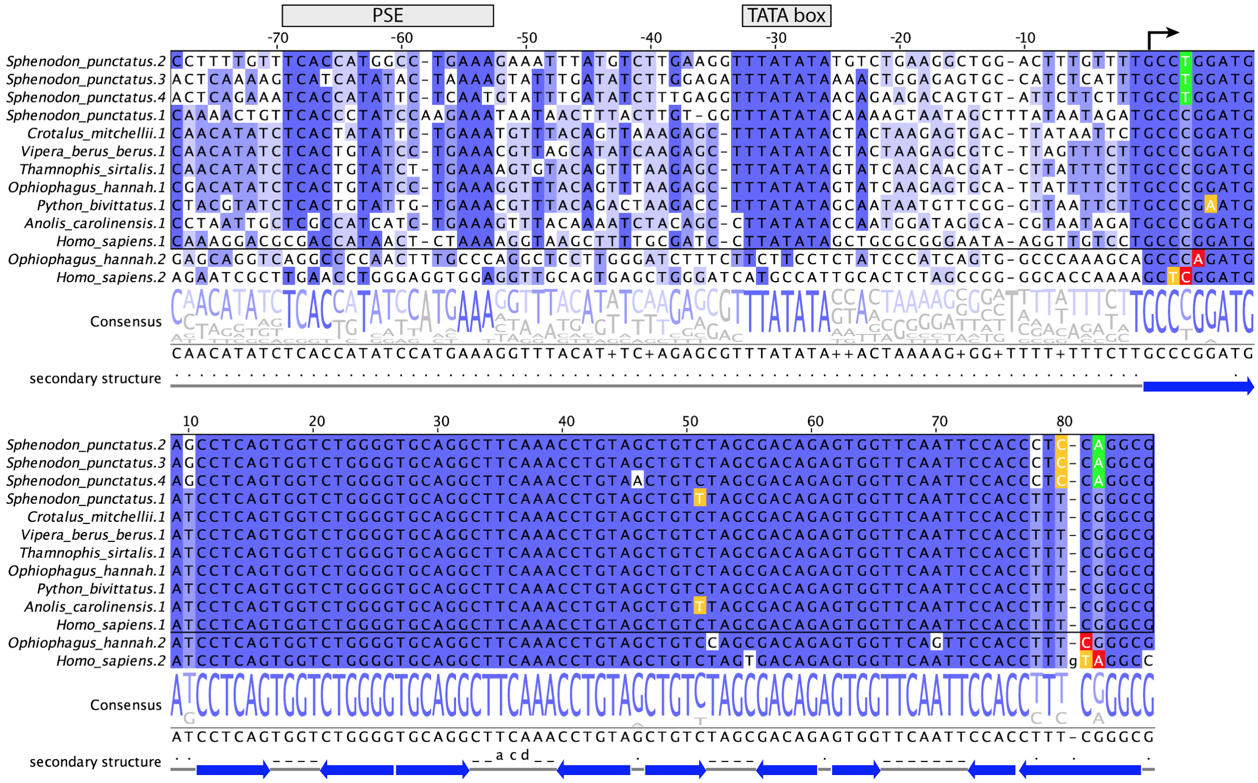

Supplementary Information Figure 13.1. Multiple sequence alignment of tRNA^{Sec} from *Lepidosauria* and human. The top four sequences, numbered from 1 to 4, were identified in the tuatara genome. The prediction number 1 is the direct ortholog of the tRNA^{Sec} gene in other species, while the extra copies 2–4 are specific to this genome. The upstream promoter elements TATA box and the proximal sequence element PSE[242] are conserved in all tRNA^{Sec}, with the exception of the two bottom sequences. These correspond to the known pseudogene in human (Homo_sapiens.2)[244], and an additional prediction in the king cobra genome (Ophiophagus_hannah.2), which is also probably a pseudogene. Mutations in the tRNA are coloured according to the secondary structure (represented by arrows at the bottom); green corresponds to compensatory changes (mutations in both residues in a pair, maintaining the pairing potential), orange corresponds to single-sided variation (mutation in one of two paired residues, with pairing maintained), and red corresponds to disrupting changes (mutations that break pairings). The blue coloured scale corresponds to sequence conservation.

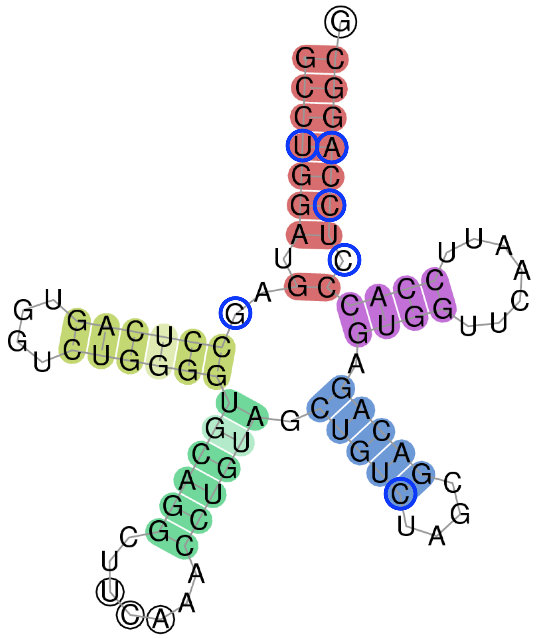

Supplementary Information Figure 13.2. Predicted secondary structure of one of the extra copies of tRNA-Sec in tuatara. All three extra copies had the same predicted structure. The sequence corresponds to Sphenodon_punctatus.2 in Supplementary Information Figure 13.1. Circled in blue are the different residues compared to the ancestor gene, Sphenodon_punctatus.1 in Supplementary Information Figure 13.1.

Supplementary Information Table 13.1. The 26 Selenoprotein genes in the tuatara genome. The protein family, the scaffold of the genome assembly, and the corresponding transcript identifiers are indicated, when available. Transcripts marked with a star contain the in-frame UGA-Sec codon. Otherwise their sequence might be incomplete.

Family	Genome	Transcriptome
DI	scaffold_2907.3419	
DI	scaffold_7697.7712	c171299_g1_i1
DI	scaffold_8323.8272	
GPX	scaffold_3968.4331	

GPX	scaffold_452.452	c196142_g1_i1*
GPX	scaffold_629.629	c60729_g1_i1*
GPX	scaffold_6905.6973	c145789_g1_i1,c28440_g1_i1*
MSRB1	scaffold_26505.25541	c154665_g2_i1*
SELENOF	scaffold_129.129	c149446_g1_i1*
SELENOH	scaffold_3111.3595	c195195_g1_i1
SELENOI	scaffold_270.270	c153700_g1_i2*,c153700_g1_i1*,c153700_g1_i3*
SELENOK	scaffold_100.100	c166617_g1_i1
SELENOM	scaffold_10583.10360	c139842_g1_i1*
SELENON	scaffold_89.89	c165428_g1_i1,c129907_g1_i3,c52835_g1_i1,c129907_g1_i1,c129907_g1_i2
SELENOO	scaffold_53.888	c147028_g1_i1*
SELENOP	scaffold_4793.5110	c128991_g1_i1*
SELENOS	scaffold_239.1054	c110652_g1_i2,c110652_g1_i1
SELENOT	scaffold_19238.18510	
SELENOT	scaffold_3522.3949	c140842_g1_i1*
SELENOU	scaffold_1074.1784	c129718_g1_i2,c129718_g1_i3*,c129718_g1_i1*
SELENOW	scaffold_4241.4607	c153750_g4_i2*,c153750_g4_i1*
SELENOW	scaffold_4981.5251	c48917_g1_i2*,c48917_g1_i1*
SPS2	scaffold_8800.8726	c2589_g1_i2*,c144616_g1_i2,c144616_g1_i1,c2589_g1_i1*
TR	scaffold_121.954	c130940_g1_i2,c118043_g1_i2*,c118043_g1_i1,c130940_g1_i1
TR	scaffold_2078.2679	c143772_g2_i1*
TR	scaffold_7984.7975	c148842_g1_i1*,c148842_g1_i2

Supplementary Information Table 13.2. Proteins from the selenoprotein synthesis machinery. The corresponding scaffold and transcript identifiers are indicated.

Family	Genome	Transcriptome
eEFsec	scaffold_434.434	c155634_g2_i1
pstk	scaffold_814.1566	c144447_g1_i1,c126277_g1_i1,c126277_g1_i2,c126277_g1_i3
SBP2	scaffold_2348.2913	c154056_g1_i1,c154056_g1_i2
secp43	scaffold_1648.2293	c150861_g1_i2,c150861_g1_i3,c150861_g1_i1
SecS	scaffold_3344.3788	c144130_g1_i1,c144130_g1_i2

14 THE TUATARA GENOME AND A COMPARISON OF DNA SUBSTITUTION RATES ACROSS AMNIOTES

Marc Tollis* and David Winter

*Correspondence to: marc.tollis@nau.edu

14.1 Introduction

The tuatara (*Sphenodon punctatus*) is the sole representative of an ancient reptilian group that has existed virtually unchanged since the Mesozoic Era. As a member of the lepidosaurian order Rhyncocephalia - the sister taxon to all living squamates - the tuatara has a conserved tetrapod phenotype, and its fossil record shows that the modern species does not differ significantly from more ancient relatives. In addition, the tuatara is a long-lived species with a relatively late onset of sexual maturity (12–22 years) and reported lifespans up to 120 years. The combination of extreme morphological conservation and long generation times in the tuatara suggests strong conservation at the genomic level, as DNA substitution rates, generation times and phenotypic divergence are often correlated in reptiles[245]. Meanwhile, the rate of DNA substitution along the tuatara branch with respect to other amniotes is a controversial subject. For instance, one study used ancient DNA specimens and a demographic model to estimate that the tuatara has the fastest recorded substitution rate among studied amniotes[246]. These results suggested a decoupling of phenotypic divergence and the rate of DNA substitution. However, these conclusions remain under considerable debate[247,248]. Clearly, more data in a larger comparative context will shed light on whether or not the tuatara embodies a paradox of molecular evolution. Here, we leverage the tuatara genome assembly, as well as those of 26 additional tetrapods, in a comparative genomic approach to estimate substitution rates across amniotes. We aim to test the hypothesis that slow life history and phenotypic divergence in tuatara are underpinned by a similarly slow divergence at the genomic level.

14.2 Whole genome alignments

We constructed 26 pairwise whole genome alignments using the green anole lizard genome (*Anolis carolinensis*, anoCar2.0) as a reference. The selection of query species was meant to represent an even phylogenetic sampling of the major groups of amniotes (lepidosaurs, birds, crocodilians, turtles and mammals), including *Sphenodon*, with *Xenopus tropicalis* as an outgroup (Supplementary Information Table 14.1). Each query species' genome assembly was aligned to the reference using LASTZ v1.02[249] to create the initial alignments, followed by "chaining" to construct gapless blocks in the alignment and "netting" to rank and select the highest scoring chains, utilizing the HOXD55 substitution score matrix[250]. Final pairwise alignments were

combined into a multiple alignment using MULTIZ v11.2[251], using the following guide tree: (Xenopus, ((Ornithorhynchus, (Monodelphis, ((Loxodonta, Dasypus), ((Homo, Mus), (Canis, Bos))))), ((((Struthio, ((Gallus, Anas), (Melopsittacus, (Taenopygia, Geospiza)))), (Alligator, (Crocodylus, Gavialis))), (Chrysemys, Pelodiscus)), (Sphenodon, (((Anolis, Pogona), ((Python, Boa), (Crotalus, Ophiophagus))), Gekko))))) which is uncontroversial based on the literature[108,252–254]. The multiple genome alignment was filtered to contain blocks with data for 24 out of 27 species.

14.3 Data extraction

To extract genetic features from the whole genome alignment, we downloaded the coding sequence annotations for the reference species *A. carolinensis* (anoCar2.0, Ensembl v86, last accessed August 2016). We then extracted sequence information for fourfold degenerate sites based upon the codon positions in the multiple genome alignment using msa_view in PHylogenetic Analysis with Space/Time Models (PHAST[255]).

14.4 Phylogenetic analysis

We reconstructed the phylogeny of the 27 tetrapods included in our analysis using the fourfold degenerate site data as a single data partition in RAxML v8.2.3[151]. We generated 20 maximum likelihood (ML) trees under the GTRCAT substitution model and conducted 500 bootstrap replicates to assess statistical support of the best ML tree. Given that this phylogeny was constructed using fourfold degenerate site data, which should evolve at close to the neutral rate, we used the topology of the best ML tree to obtain a nonconserved model of evolution for the substitution rate analysis using phyloFit in PHAST, by fitting the tree model to the data using a time-reversible substitution model (REV).

14.5 Substitution rate estimation

Using the topology and branch lengths obtained from the best ML phylogeny, we estimated absolute rates of molecular evolution in terms of substitution per site per million years and estimated the divergence times of amniotes via the semiparametric penalized likelihood (PL) method[256] with the program r8s v1.8[257]. We ran two separate PL analyses based on different node constraint criteria. The first set of criteria utilized fossil constraints: the minimum age of Diapsida (i.e., the time to most recent common ancestor or TMRCA of chicken and Anolis) was set at 255.9 My, the minimum age of Archosauria was set at 247.1 My, the minimum and maximum ages for Mammalia were set at 164.9–201.5 My, minimum and maximum ages for Neognathae (i.e., the TMRCA of chicken and zebra finch) were set at 66–86.8 My, and the minimum and maximum ages for Serpentes (i.e., the TMRCA of python and cobra) were set at 98.32–113 My[258,259]. The second set of criteria utilized the median estimated ages obtained from www.timetree.org[260]: the TMRCA of sauropsids was fixed at 283 My, the TMRCA of lepidosaurs was fixed at 252 My, the

TMRCA of Archosauria was fixed at 237 My, the TMRCA for Mammalia was fixed at 177 My, the TMRCA for Eutheria was fixed at 105 My. Across both analyses, we fixed the root of the tree at 352 My, the TMRCA of amniotes at 312 My, and the TMRCA of crocodilians at 80 My[108]. The PL method estimates a different substitution rate on each branch and relies on a "roughness" penalty when these rates differ greatly, quantified by a smoothing parameter for which larger values indicate a clock-like model[256]. For each set of node constraint criteria, we used cross-validation to optimize the smoothing parameter, allowing values to range on a log10 scale starting from 100 with the exponent increasing 0.3 for a total of ten steps, and reran the analysis with the optimal value. We also used the gradient check implemented in r8s v1.8 to ensure the signs of any active constraints were correct (i.e. negative if a minimum constraint was used) and estimated divergence times for all unconstrained nodes.

14.6 Results

The filtered whole genome alignment was 790,566,929 bases in length, with 17.2% gaps and an average of 25.7 species represented in each alignment block, and we extracted 818,968 fourfold degenerate sites. The ML phylogeny included a topology consistent with expectations: most notably that Sphenodon was the sister taxon to all included squamates, with 100% bootstrap support for all branches (Supplementary Information Figure 14.1). Node constraints utilizing both criteria passed all gradient checks, and the TMRCA for Sphenodon and squamates (i.e. Lepidosauria) was highly similar across the two methods (Supplementary Information Figures 14.2 and 14.3) at 251.2 and 252 My, respectively. The optimal value for the smoothing parameter was one, indicating a significant deviation from the molecular clock. The analyses using different constraint criteria differed slightly in their absolute estimated substitution rates, but not in relative rates. Across analyses, the tuatara resulted in the slowest estimated substitution rate among lepidosaurs (Supplementary Information Figure 14.4).

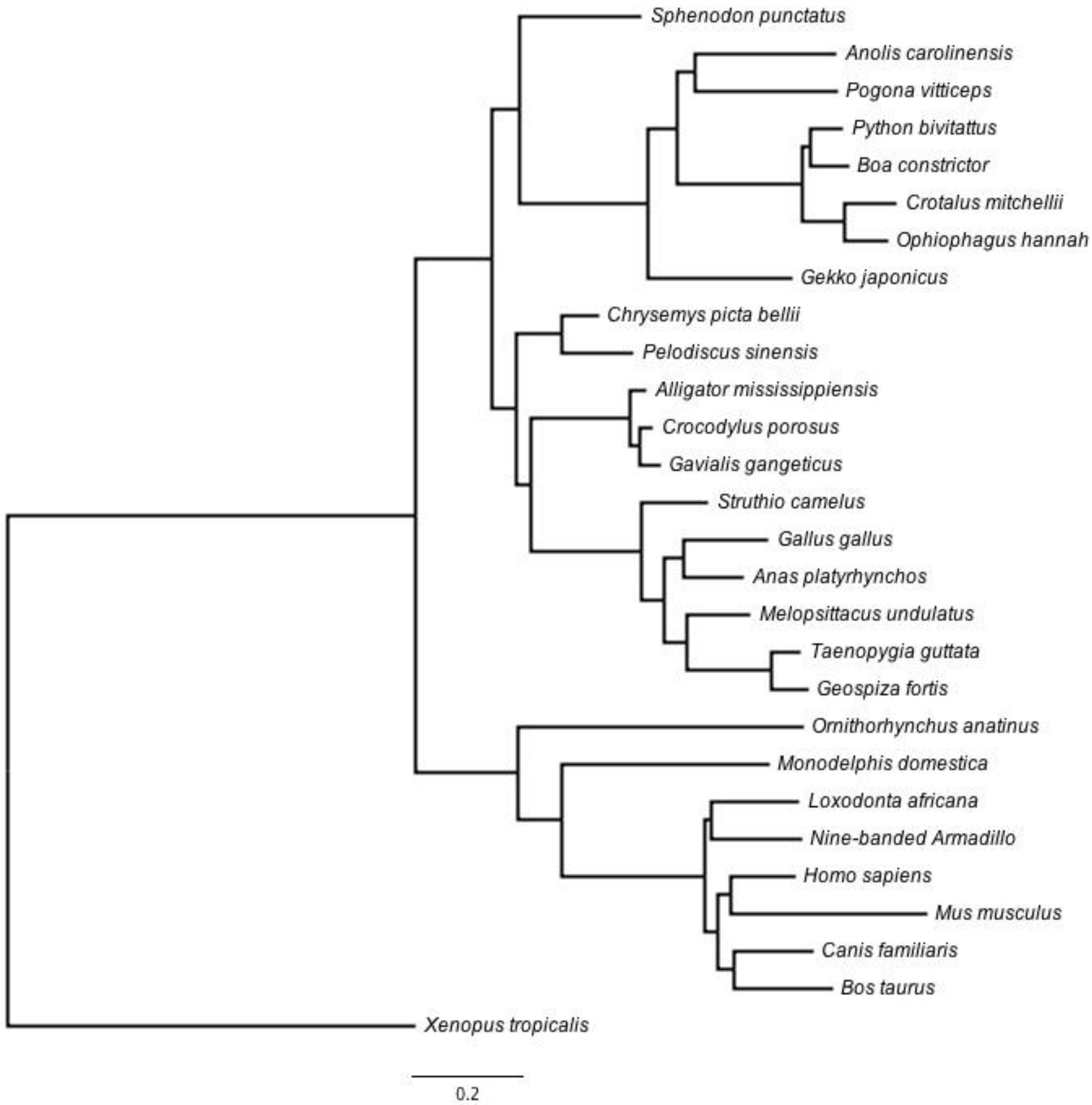

Supplementary Information Figure 14.1. Maximum likelihood phylogenetic reconstruction of 27 tetrapods using 818,968 fourfold degenerate sites. We obtained full support for all branches using 500 bootstrap replicates.

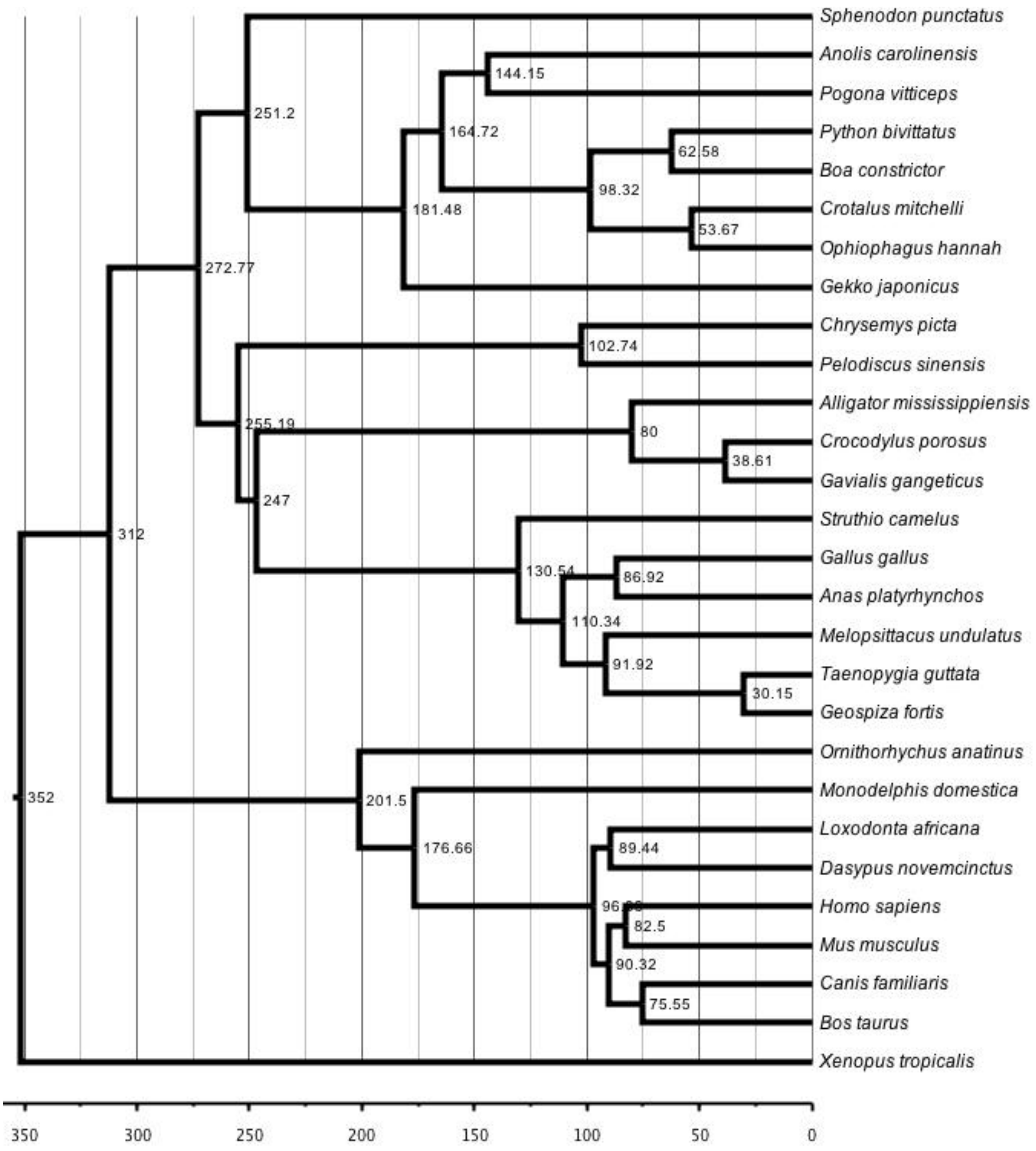

Supplementary Information Figure 14.2. Chronogram of amniote evolution obtained from penalized likelihood estimation of the phylogeny derived from fourfold degenerate sites and fossil calibrations. Node labels indicating ages and x-axis are given in millions of years.

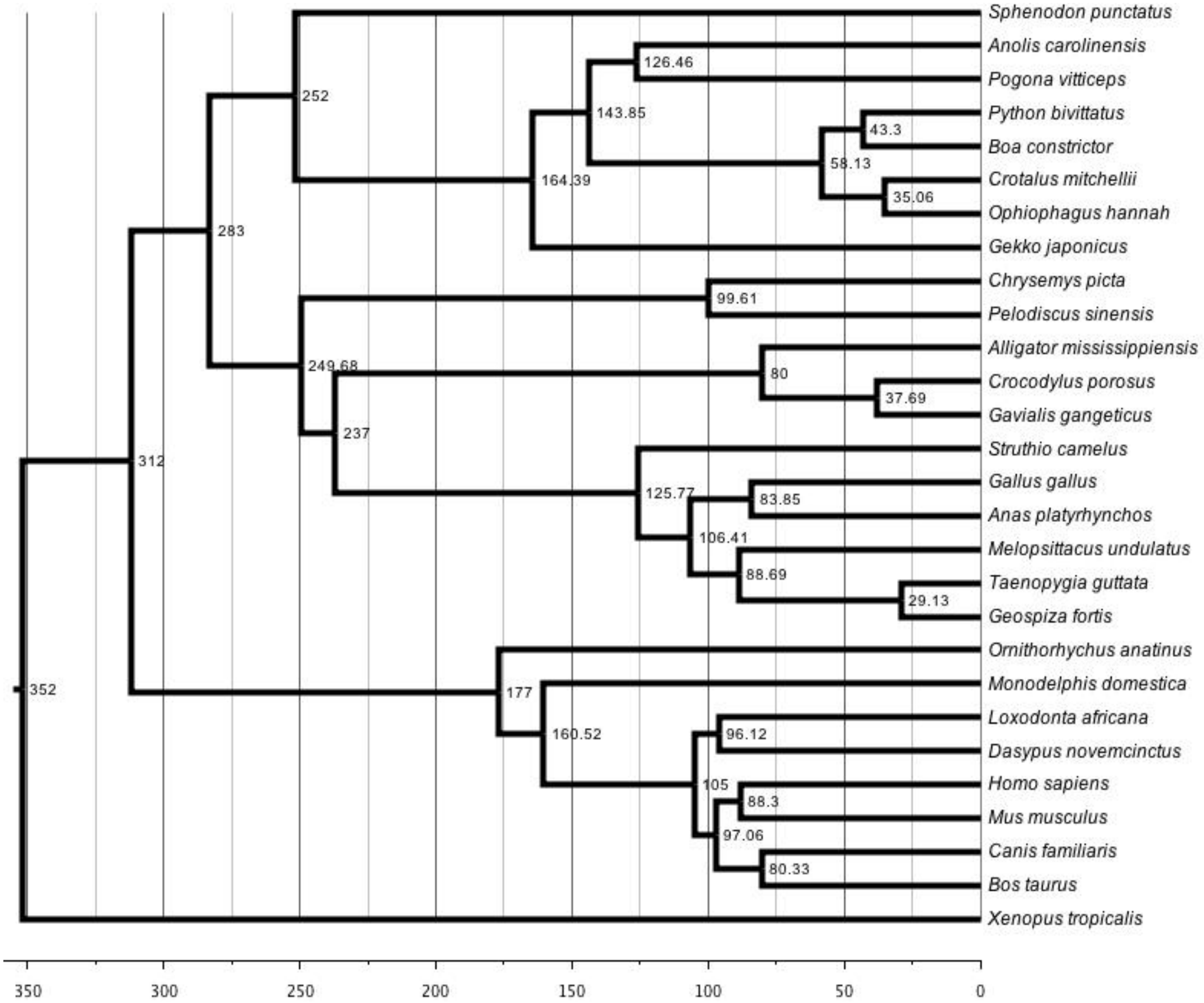

Supplementary Information Figure 14.3. Chronogram of amniote evolution obtained from penalized likelihood estimation of the phylogeny derived from fourfold degenerate sites and timetree calibrations. Node labels indicating ages and x-axis are given in millions of years.

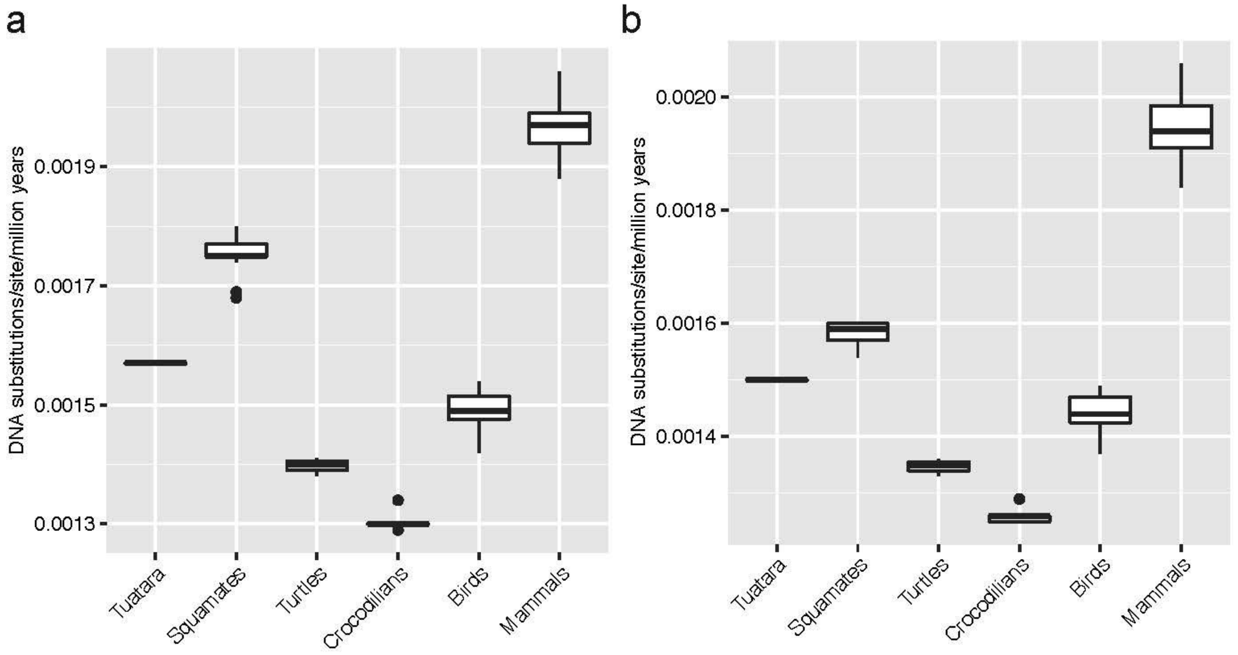

Supplementary Information Figure 14.4. Estimated DNA substitution rates of amniote clades based on fourfold degenerate sites. a, Boxplots showing distribution of estimated substitution rates by clade using semiparametric penalized likelihood in r8s with fossil constraints from fossil constraints from Benton et al. [258] and Head [259] for tuatara (n=1, 0.00159), squamates (n=13, min=0.00171, max=0.00183, median=0.00178, 25th percentile=0.00178, 75th percentile=0.00183), turtles (n=3, min=0.00138, max=0.00141, median=0.00140, 25th percentile=0.00139, 75th percentile=0.00140), crocodilians (n=5, min=0.00128, max=0.0133, median=0.00129, 25th percentile=0.00129, 75th percentile=0.00129), birds (n=11, min=0.0014, max=0.00152, median=0.00147, 25th percentile=0.00146, 75th percentile=0.0015), and mammals (n=15, min=0.00188, max=0.00208, median=0.00197, 25th percentile=0.00194, 75th percentile=0.00201). b, Boxplots showing distribution of estimated substitution rates with median TMRCA estimates from timetree.org for tuatara (n=1, 0.00157), squamates (n=13, min=0.00168, max=0.00180, median=0.00175, 25th percentile=0.00178, 75th percentile=0.00177), turtles (n=3, min=0.00138, max=0.00141, median=0.00140, 25th percentile=0.00139, 75th percentile=0.00140), crocodilians (n=5, min=0.00129, max=0.0134, median=0.00130, 25th percentile=0.00130, 75th percentile=0.00130), birds (n=11, min=0.00142, max=0.00154, median=0.00149, 25th percentile=0.00147, 75th percentile=0.00152), and mammals (n=15, min=0.00188, max=0.00206, median=0.00197, 25th percentile=0.00194, 75th percentile=0.00199).

Supplementary Information Table 14.1. Major clades, species, genome assemblies and sources used for the whole genome alignments in this study. *reference sequence; UCSC, University of California Santa Cruz Genome Browser; NCBI, National Center for Biotechnology Information

Clade	Common Name	Scientific Name	Genome Assembly Version	Repository
Lepidosaurs	Tuatara	*Sphenodon punctatus*	tuatara_30Sep2015_rUdWx	Current
	Green anole	*Anolis carolinensis*	AnoCar2.0*	UCSC
	Burmese python	*Python molurus bivittatus*	pitBiv5.0.2	NCBI
	Speckled rattlesnake	*Crotalus mitchelli*	CroMitch1.0	NCBI
	King cobra	*Ophiophagus hanna*	OphHan1.0	NCBI
	Schlegel's Japanese gecko	*Gekko japonicus*	Gekko_japonicus_V1.1	NCBI
	Boa constrictor	*Boa constrictor*	snake_7C_scaffolds	DOI: 10.1186/2047-217X-2-10
Birds	Common ostrich	*Struthio camelus*	STRCAM	GigaDB
	Chicken	*Gallus gallus*	galGal3	UCSC
	Duck	*Anas platyrhyncos*	anaPla1	UCSC
	Zebra finch	*Taenopygia gutatta*	taeGut3	UCSC
	Medium ground finch	*Geospiza fortis*	geoFor1	UCSC

	Budgerigar	*Melopsittacus*	melUnd1	UCSC
Crocodilians	Saltwater crocodile	*Crocodylus porosus*	croc_sub2	www.crocgenomes.org
	Indian gharial	*Gavialis gangeticus*	ggan.v0.2	www.crocgenomes.org
	American alligator	*Alligator mississippiensis*	allMis1	UCSC
Turtles	Chinese softshell turtle	*Pelodiscus sinensis*	PelSin_1.0	NCBI
	Western painted turtle	*Chrysemys picta bellii*	Chrysemys_picta_bellii-3.0.3	NCBI
Mammals	Gray short-tailed opossum	*Monodelphis domestica*	monDom5	UCSC
	Platypus	*Ornithorhynchus anatinus*	ornAna1	UCSC
	House mouse	*Mus musculus*	mm10	UCSC
	Domestic dog	*Canis lupus familiaris*	canFam3	UCSC
	Cow	*Bos taurus*	bosTau8	UCSC
	African savannah elephant	*Loxodonta africana*	loxAfr3	UCSC
	Nine-banded armadillo	*Dasypus novemcinctus*	dasNov3	UCSC
	Human	*Homo sapiens*	hg19	UCSC
Amphibian	Western clawed frog	*Xenopus tropicalis*	xenTro3	UCSC

15 PHYLOGENETIC ANALYSIS OF VERTEBRATE SINGLE-COPY ORTHOLOGS

Marc Tollis* and Stefan Prost

*Correspondence to: marc.tollis@nau.edu

We analyzed 245 single copy orthologs present in 26 vertebrates, including tuatara, which were aligned by the codon-based PRANK algorithm[261] and concatenated and partitioned with FASconCAT-G v1.02[262]. Using RAxML v8.2.3[151], we generated 20 maximum likelihood trees from the 245 partitions under the GTRGAMMA substitution model, and selected the tree with the highest likelihood (Supplementary Information Figure 15.1). We assessed branch support on the best tree using 1,000 bootstrap replicates.

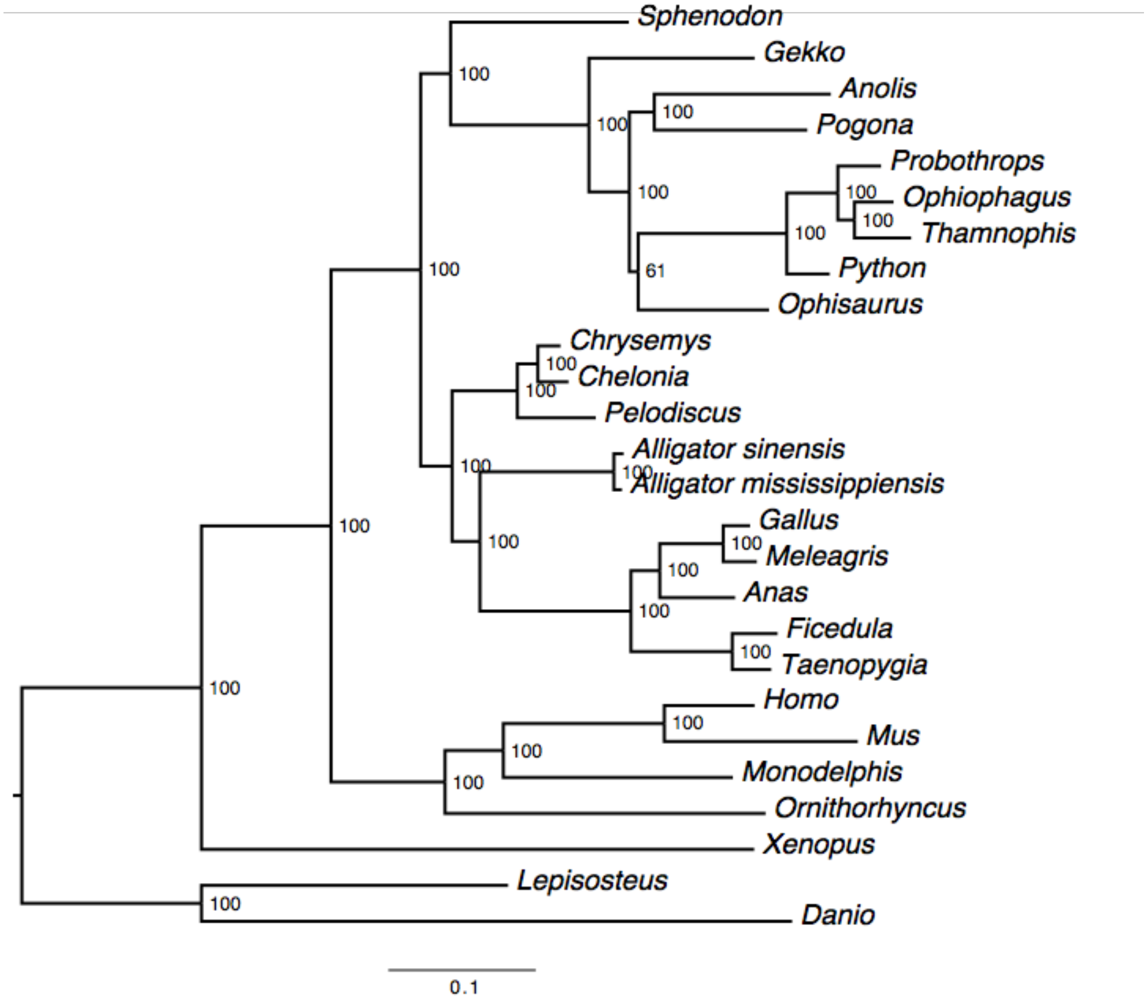

Supplementary Information Figure 15.1. Maximum likelihood phylogeny estimated from a concatenated supermatrix of 245 single copy ortholog partitions. Branch lengths are in terms of substitutions per site. Branch support from 1,000 bootstrap replicates is shown.

We also generated gene trees based on the 245 one-to-one orthologs found between all species. To do so, each aligned gene sequence was first analyzed phylogenetically using RaxML[151]; using the GTRGAMMA model. Subsequently, we binned all gene trees to reconstruct a species tree (Supplementary Information Figure 15.2) using Astral[263], assessing branch support using local posterior probabilities[264]. Astral applies binning of gene trees with similar topologies, based on an incompatibility graph between gene trees. Subsequently, it chooses the most likely species tree under the multi-species coalescent model.

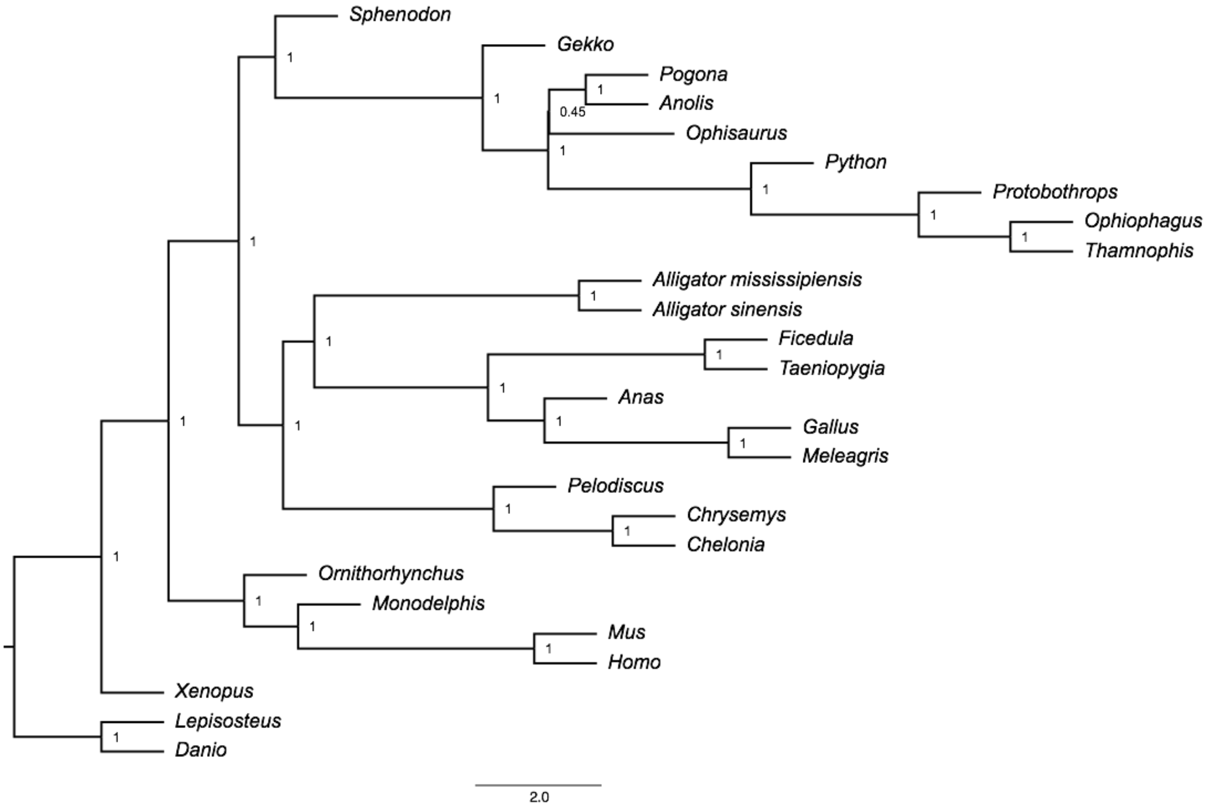

Supplementary Information Figure 15.2. Species tree reconstruction using 245 single copy orthologs. Branch lengths are given in terms of coalescent units. Local posterior probabilities are given for each branch.

Finally, we obtained the amino acid sequences of 128 one-to-one single copy orthologous proteins from the 26 vertebrates, which were aligned using MAFFT v7.450[150] and concatenated into a partitioned supermatrix with AMAS (https://github.com/marekborowiec/AMAS)[265]. The concatenated alignment was 89,346 bp in length containing 27,263 parsimony-informative sites.

We carried out maximum likelihood tree estimation in IQ-TREE v1.6.12[266] with model testing for each partition[267] and assessed branch support using 10,000 ultrafast bootstraps[268] and SH-aLRT replicates[269], see Supplementary Information Figure 15.3.

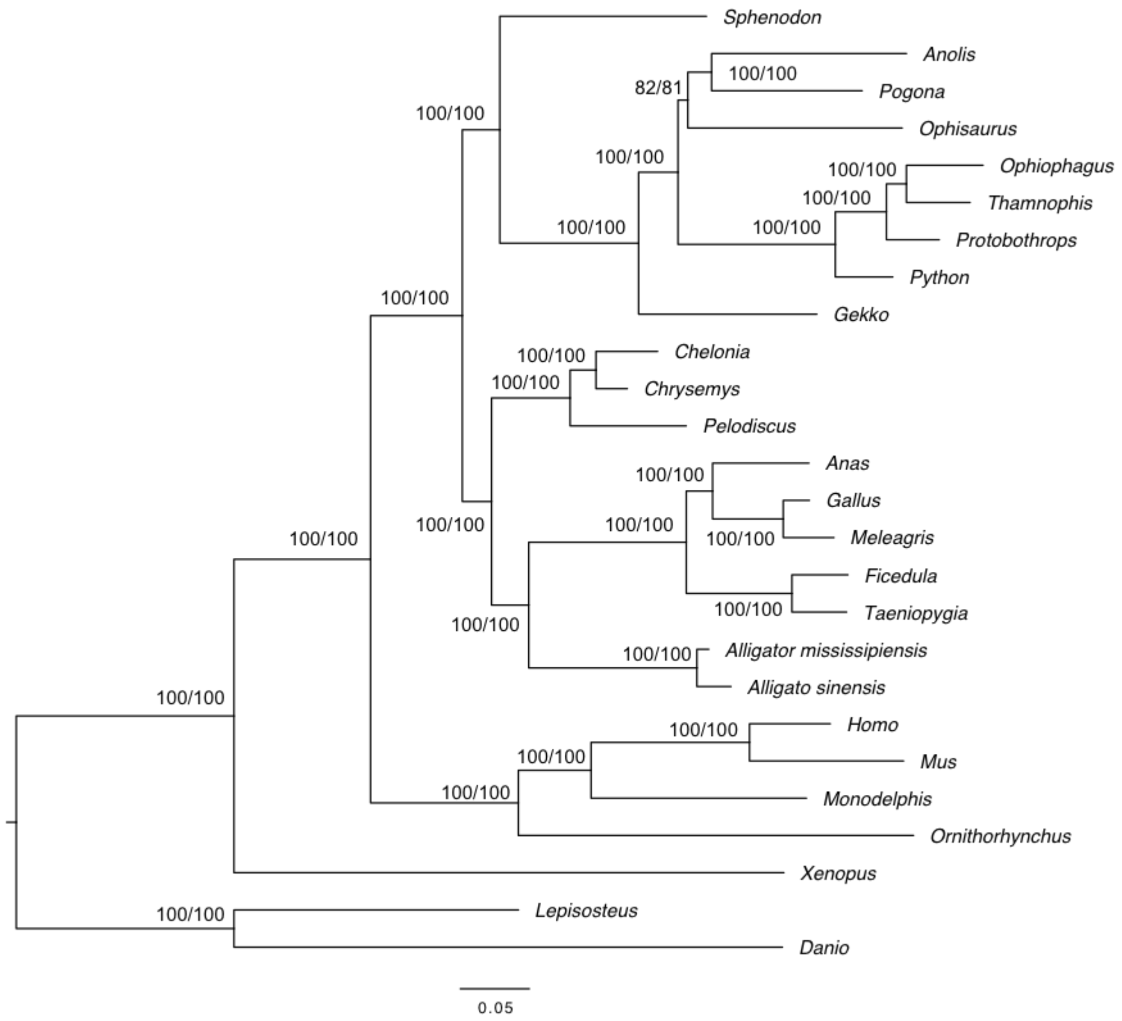

Supplementary Information Figure 15.3 Maximum likelihood phylogeny reconstructed from 128 one-to-one orthologous proteins from 26 vertebrates. Branch lengths are shown in substitutions per site. Support values are shown for 10,000 ultrafast bootstrap (before slash) and SH-aLRT (after slash) replicates.

16 ANALYSIS OF DIVERGENCE TIMES AND A TEST FOR PUNCTUATED EVOLUTION

Chris L. Organ

Correspondence to: organ@montana.edu

16.1 Divergence times

We inferred time-calibrated phylogenies with BEAST v2.4.8[270] using the CIPRES Science Gateway[115]. We randomly sampled 50 of the 245 one-to-one ortholog alignments generated for the gene tree reconstruction (Supplementary Materials 15). This alignment had 166,307 sites, which were partitioned using a GRT model with a four-category gamma distribution of rate variation. The proportion of invariant sites was set to 0.1 and the nucleotide state frequencies was set to empirical. We used a calibrated Yule prior for the tree with a gamma prior (alpha=0.001, beta=1,000) on the birth rate. We also implemented a relaxed log normal clock – the prior for the mean and standard deviation of the branch rates were drawn from gamma distributions (alpha=0.001, beta=1,000; and alpha=0.5, beta=1). Given the general uncertainty surrounding the ancestry of fossil lineages, we chose to be conservative and used normal priors on node calibrations drawn from established resources[258,259] as follows: Archosauria (m=254, s=2.0), Diapsida (m=276, s=7.0), Mammalia (m=183.2, s=7.0), Neornithes (m=76.5, s=4.0), Root/Vertebrata (m=444.5, s=8.0), Serpentes (m=105.5, s=3.0), and Tetrapoda (m=344, s=3.0). The MCMC chain ran for 20,000,000 generations with a sampling frequency of 1,000. The analysis was repeated using a new random gene set four times, which, along with the ESS parameter reported in BEAST, was used to assess convergence.

16.2 Punctuated evolution

Evolution is a temporally heterogeneous process. The rate of evolution is expected to vary through time and across taxonomic groups, especially if change is associated with speciation events, a process known as punctuated evolution[271]. The process of punctuated genome evolution is expected to produce a pattern of correlation between the amount of genome evolution and the net number of speciation events along a lineage. We used Bayesian phylogenetic generalized least squares to regress the total phylogenetic path length (of four-fold degenerate sites) on the net number of speciation events (nodes in a phylogenetic tree)[272]. We find strong evidence for punctuated evolution at these sites (pMCMC ≈ 0; 100% of the posterior probability for the line's slope was greater than 0, the null value). Thirty-nine percent (95% credible interval = 0.15 to 0.62) of branch length variation is attributable to punctuated evolution. Furthermore, punctuated evolution accounts for 33.5% (r^2; 95% credible interval = 0.34 to 0.38) of deviation from the molecular clock at four-fold degenerate sites (Supplementary Information Figure 16.1).

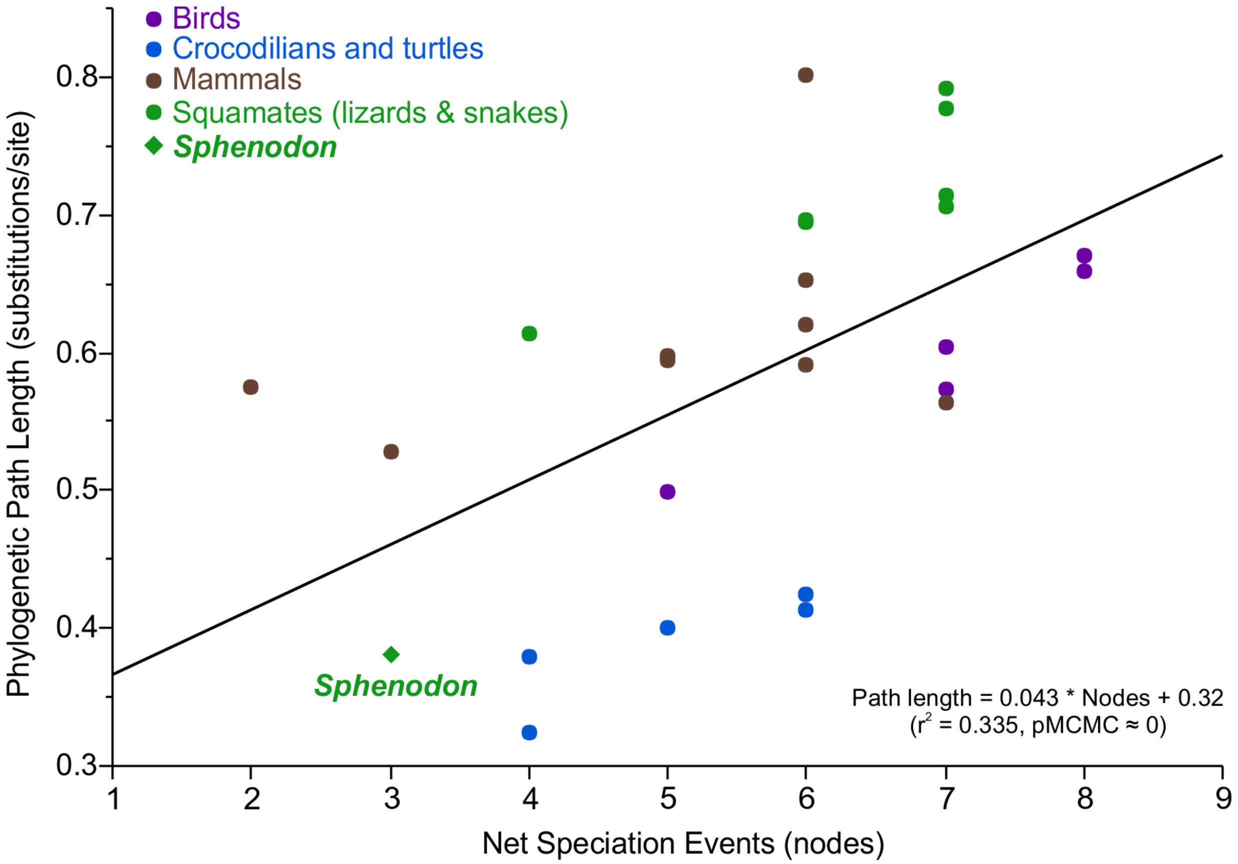

Supplementary Information Figure 16.1. Punctuated genome evolution. Note that the *Sphenodon* genome has undergone less evolution than expected given its history of net speciation.

As a "living fossil", we might expect Sphenodon to be an outlier in this analysis, with rates below the phylogenetic average. To test this, we removed Sphenodon and inferred another punctuated regression model (as described above) to predict its path length using its phylogenetic position and its net number of speciation events[273]. We predict a path length of 0.44 substitutions/site (95% credible prediction interval = 0.29 to 0.58) for *Sphenodon*, which does not differ substantially from its inferred length of 0.38 substitutions/site. The rate of *Sphenodon* evolution is 0.0008 substitutions/site/millions of years. The only more slowly evolving genomes are those of basal Archelosauria (turtles and crocodilians), which have an average rate of 0.00056 substitutions/site/millions of years. Previous analyses of the rate at which genomes evolve have not accounted for punctuated evolution. An analysis similar to previous studies (of path lengths but not including nodes) supports *Sphenodon* as having undergone less evolution than other species (posterior predictive median = 0.52, 95% credible prediction interval = 0.36 to 0.68, pMCMC = 0.045). Together, these models support the hypothesis that the slowly evolving genome of *Sphenodon* is attributable to its 240-plus million-year history without substantial diversification.

These results could be explained by underestimated branch lengths in regions of a phylogeny that have few species (either because of taxon sampling or extinction, or both). Multiple substitutions won't be detected along these paths[274], but this artifact can be detected[274,275]. We tested for this artifact (http://www.evolution.reading.ac.uk/pe/index.html) but found no evidence for its presence.

17 PATTERNS OF SELECTION ON TUATARA ORTHOLOGS

Shawn M. Rupp, Victoria G. Twort, Thomas R. Buckley*, Melissa A. Wilson*,

*Correspondence to: buckleyt@landcareresearch.co.nz and mwilsons@asu.edu

We performed two analyses on ortholog sets from squamate genomes. These analyses were focused on investigating patterns of selection among squamate genomes and comparing patterns of molecular evolution at sex determining genes relative to other orthologs.

17.1 Methods

17.1.1 Positive selection on the tuatara lineage

The first analysis was targeted at detecting positive selection along the branch leading to tuatara. One-to-one orthologs were extracted from the ortholog file and those absent in the tuatara were excluded. The resulting ortholog sets were realigned using a codon-based method. First, nucleotides were translated to amino acids using Translatorx[276], then aligned using MAFFT[150,277], using the auto option to select the most appropriate alignment strategy. The corresponding codon alignment was constructed from the amino acid alignment using PAL2NAL[278]. Alignments were then screened using a set of custom scripts and TrimAl[279] to check the frame, replace stop codons and remove gapped regions. Only alignments with 12 or more of the 17 species were included. Gene trees were reconstructed in Garli under the GTR+I model.

Alignments were then analysed in CodeML from the PAML[198] package using the tuatara branch as the foreground branch. Gapped sites were included in these analyses. The branch model was employed. The resulting p-values were corrected with FDR of 5% with the R package Qvalue[280]. Gene ontology (GO) and KEGG enrichment tests analyses were carried out with Kobas[281]. A Fisher's exact test was used to assess significantly overrepresented GO and KEGG categories in the test set of tuatara orthologs. The Anolis, mouse and human annotations were used as references with the tuatara orthologs used as the test data set. Transcripts were identified using Blastx against the nr database.

17.1.2 Patterns of molecular evolution at sex determining genes

The second analysis compared patterns of molecular evolution at sex determining genes relative to other orthologs. We used multiz[249] to create a multiple alignment of six of the species previously aligned to the green anole (anoCar2). These species included the tuatara (sphPun), gecko (gekJap1), bearded dragon (pogVit1), Burmese python (pytBiv1), speckled rattlesnake (croMit1), and king cobra (ophHan1). This resulted in an alignment of seven species (including the anole) comparing the tuatara and six squamate species.

To obtain aligned coding sequences, we submitted the multiple alignment to Galaxy's Stitch Gene Blocks Tool[282] using the BED12 genome annotation for the green anole (anoCar2) imported from the UCSC Table Browser[283]. We submitted the resulting CDS fasta alignment to AlignmentProcessor0.12 (https://github.com/WilsonSayresLab/AlignmentProcessor) on Arizona State University's Ocotillo cluster to perform quality control and run CodeML[198] on each gene alignment. CodeML[198] was run twice for each gene: once for the null model in which dN/dS was assumed to be zero, and again for the alternative model in which the tuatara was specified as the forward branch and dN/dS was allowed to vary for each branch. Next, we subset the CodeML[198] output to include only 1 to 1 orthologs using the database generated previously for this project.

We compiled CodeML[198] output for each model and conducted a Likelihood Ratio Test in R3.3.1[179] using the log likelihood values generated by CodeML[198]. We then compared overall dN, dS, dN/dS, and tree lengths between sex-determining genes and non-sex-determining genes for gene alignments with a minimum of three species which contained the tuatara. We calculated mean and median values in R3.3.1[179] and determined 95% confidence interval using R boot package[284] using 1,000 bootstrap replicates. We calculated p values both using Wilcoxon Rank Sum Tests and permutation analyses with 10,000 replicates using in lab scripts.

Lastly, we calculated the branch lengths of the tuatara branch and the species with the longest branch length (other than the tuatara) and merged this data with the likelihood ratio test output to compare the tuatara's branch length to the overall tree length excluding the tuatara. We examined significant genes to see whether the tuatara showed a pattern of faster evolution when compared to the rest of the phylogeny. Genes were considered "faster" than the rest of the tree if the tuatara had a greater branch length than the squamate species with the longest branch length and were considered "slower" if the tuatara's branch length was less than the longest squamate branch. We calculated the percentage of significantly faster, significantly slower, and not significantly different genes for sex-determining genes, non-sex-determining genes, and all genes.

17.2 Results

17.2.1 Positive selection on the tuatara lineage

Extended analyses through an independent pipeline yielded a total of 4284 ortholog sets which were analysed in CodeML. Of these 659 orthologs had corrected p-values <0.05 from the likelihood ratio test of the equal ω across branches versus a model where the tuatara (foreground) branch had a different ω value to shared ω across all other (background) branches. None of the tests with significant p-values have a foreground ω>1. Supplementary Data 1. https://zenodo.org/record/2583274

Using the *Anolis*, human and mouse annotations a total of 14,435 and 11 KEGG pathways were significantly overrepresented, respectively. With human and mouse annotations a total of 1,910 and 73 GO categories were overrepresented. The KEGG pathways from the comparison to *Anolis*

include a number of RNA and mRNA regulation and metabolic pathways as well as general metabolism. Supplementary Data 2. https://zenodo.org/record/2583274

17.2.2 Patterns of molecular evolution at sex determining genes

We find that most genes exhibit a pattern of molecular evolution that suggests the tuatara branch evolves at a different rate than the rest of the tree (Supplementary Information Table 17.1). When comparing genes involved in the sex-determination pathways (Supplementary Information Table 17.2), we find that both the entire length of the tree is shorter for these genes (Supplementary Information Table 17.3) and that dN and dN/dS values are lower for genes involved in sex determination than typical genes in the genome (Supplementary Information Table 17.4; Supplementary Information Figure 17.1), although this pattern is not significant (Fishers exact test, p=0.48).

Supplementary Information Table 17.1. Tuatara–squamate gene tree evolution compared between all genes and sex determining genes. A log-likelihood test comparing the likelihood of a model where the rate of evolution was the same on all branches compared to the likelihood of a model where there was a different rate of evolution on the tuatara branch was conducted for all genes present in both the tuatara and anole (the reference for the alignment) and were present in a minimum of three species. The log likelihood was calculated for each gene for both the null and alternative model in CodeML[198] and a likelihood ratio test conducted in R3.3.1[179] using alpha of 0.05 and 1 degree of freedom.

		Genes which Are Significantly Different from the Null Model	Total Number of Genes
**All Genes**	**Percentage**	75.91%	9,586
	**Number of Genes**	7,277	
**Sex Determining Genes**	**Percentage**	84.0%	25
	**Number of Genes**	21	
**Non-Sex-Determining Genes**	**Percentage**	75.89%	9,561
	**Number of Genes**	7,256	

Supplementary Information Table 17.2. Sex determining gene substitution rates. We computed substitution rates, d_N, d_S, and d_N/d_S, using PAML[198] for genes previously identified as playing a primary role in sex determination. For eight sex determining genes we did not identify homologous sequence in the tuatara in the multiple species alignment (highlighted in gray), likely as a consequence of alignment failure due to high sequence divergence. Prior identification of *dmrt1* in our genome data[285] and its earlier localisation in the tuatara genome by gene mapping[286] supports this view.

TranscriptID	GeneSymbol	dN	dS	dNdS	Tree Length	Tuatara branch length
ENSACAT00000009605	AR (DHTR)	0.0900	2.0497	0.0439	1.9847	0.0571163
ENSACAT00000008263	ARX	0.0749	1.4746	0.0508	1.3473	0.0348331
ENSACAT00000000495	CIRBP	0.0461	2.4627	0.0187	2.1876	0.0636031
ENSACAT00000004647	CSDC2	0.0933	1.4834	0.0629	1.5226	0.106651
ENSACAT00000003256	CSDE1	0.0464	0.8424	0.0551	0.8258	0.0575427
ENSACAT00000015874	DHCR7	0.3298	1.6549	0.1993	2.1308	0.134717
ENSACAT00000010360	EMX2	0.1142	1.8239	0.0626	1.7465	0.0348258
ENSACAT00000006243	ESR1	0.1894	1.4322	0.1322	1.6464	0.113636
ENSACAT00000001292	FEM1B	0.0809	2.3919	0.0338	2.2297	0.0435242
ENSACAT00000015028	JAKMIP1	0.3526	2.9505	0.1195	3.2637	0.056045
ENSACAT00000014908	JAKMIP2	0.0494	1.5791	0.0313	1.4699	0.0361767
ENSACAT00000008030	JAKMIP3	0.0968	1.2884	0.0751	1.3204	0.095763
ENSACAT00000002517	LHX1/LIM1	0.1131	2.3564	0.0480	2.2445	0.0276626
ENSACAT00000014613	LHX9	0.0500	0.9320	0.0536	0.8877	0.0480989
ENSACAT00000030595	M33/TUSC3	0.0155	1.4471	0.0107	1.2941	0.0125199
ENSACAT00000014130	PAX2	0.0271	0.8004	0.0339	0.7732	0.0621367
ENSACAT00000030504	PDGFB	0.3974	1.2046	0.3299	1.9014	0.280557
ENSACAT00000017280	RSPO1	0.2540	1.0624	0.2391	1.4557	0.192456
ENSACAT00000016485	SF1	0.0415	2.0755	0.0200	1.8859	0.00712568
ENSACAT00000008756	SOX9	0.1188	1.8735	0.0634	1.8038	0.129618
ENSACAT00000018021	STAT3	0.0357	2.1405	0.0167	1.9370	0.0282673
ENSACAT00000005401	UBA1	0.1646	1.9622	0.0839	2.0987	0.11493
ENSACAT00000012577	WNT4	0.4560	2.9148	0.1564	3.2907	0.0161123
ENSACAT00000002516	WT1	0.0986	1.2456	0.0792	1.3415	0.110817
ENSACAT00000009898	ZFPM2/FOG2	0.2839	1.3392	0.2120	1.7684	0.227515
ENSACAT00000015597	AMH	0.5398	1.9654	0.2747	2.8312	NA
ENSACAT00000002340	DMRT1	0.0895	0.7924	0.1129	0.8935	NA
ENSACAT00000001907	GATA4	0.1677	1.5525	0.1080	1.6969	NA
ENSACAT00000007239	MSL2	0.0344	0.5761	0.0597	0.5901	NA
ENSACAT00000010492	SDC1	0.7469	1.5435	0.4839	2.9324	NA
ENSACAT00000006298	STAT1	0.1039	0.8107	0.1282	0.9141	NA
ENSACAT00000025425	STAT2	0.3812	1.1877	0.3210	1.8272	NA
ENSACAT00000009792	STAT6	0.2880	1.1722	0.2457	1.6266	NA

Supplementary Information Table 17.3. Tuatara–squamate tree lengths for sex determining genes. Here we compare tree lengths from the alternative models. All genes examined were present in both the tuatara and anole and were present in a minimum of three species. 95% confidence intervals were calculated with 1000 bootstrap replicates. P values were calculated using a Wilcoxon rank sum test.

		Sex Determining Genes	All Other Genes	P Wilcoxon Test
dN	mean	0.1448 (0.1295, 0.1571)	0.2495 (0.2426, 0.2864)	0.0014
	median	0.0968 (0.0909, 0.1029)	0.2051 (0.1487, 0.1993)	
dS	mean	1.712 (1.733, 1.868)	1.876 (1.857, 2.273)	0.3976
	median	1.579 (1.455, 1.712)	1.735 (1.662, 1.803)	
dN/dS	mean	0.0893 (0.0799, 0.0978)	0.2670 (0.000, 1.391)	0.0024
	median	1.774 (1.695, 1.831)	0.1175 (0.1019, 0.1304)	
Tree Length	mean	1.774 (1.738, 1.870)	2.147 (1.886, 2.279)	0.0298
	median	1.768 (1.665, 1.779)	1.986 (1.801, 2.000)	
Number of Genes		25	9561	

Supplementary Information Table 17.4. Log-likelihood of tuatara branch length comparisons across gene trees. All genes examined were present in the tuatara, anole, and at least one additional species and had log likelihood values and tree lengths for both the null and alternative models. Genes were identified as significant or not using a two-sided likelihood ratio test comparison between a null (equal rate) and alternative (unequal rate) model of the substitution rate on the tuatara branch length.

		Tuatara Branch is Significantly Shorter	Tuatara Branch is Significantly Longer	Tuatara Branch is not Significantly Different	Total Number of Genes
Sex Determining Genes	Percentage	44.0%	40.0%	16.0%	25
	Number of Genes	11	10	4	
Non-Sex Determining Genes	Percentage	36.44%	39.34%	24.11%	9,561
	Number of Genes	3,484	3,761	2,305	
All Genes	Percentage	49.83%	39.34%	24.09%	9,586
	Number of Genes	3,495	3,771	2,309	

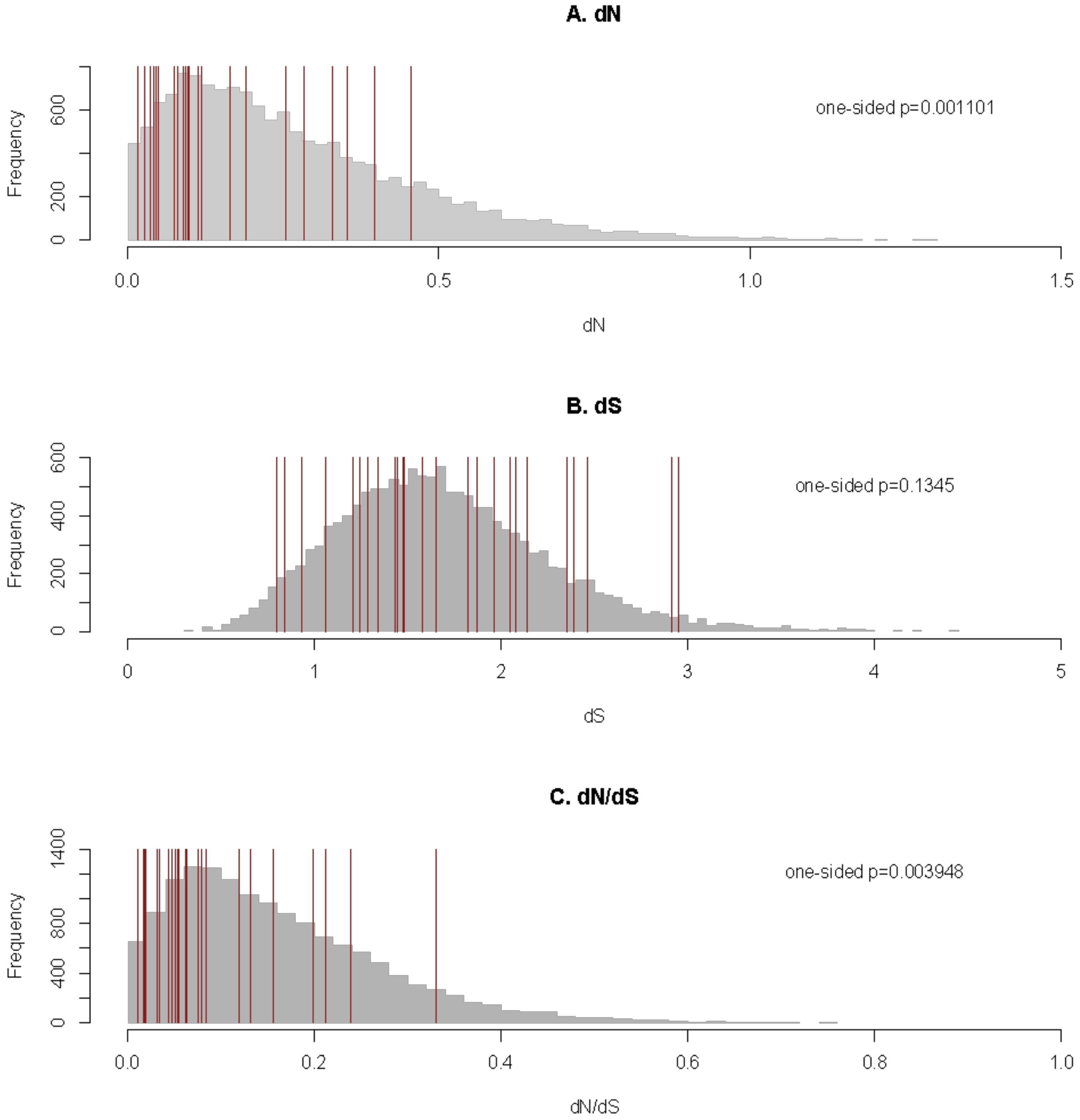

Supplementary Information Figure 17.1. Distribution of dN, dS, and dN/dS values for sex-determining genes versus all genes in the seven-way species alignment. The seven-species multiple species alignment includes tuatara (sphPun), gecko (gekJap1), bearded dragon (pogVit1), Burmese python (pytBiv1), speckled rattlesnake (croMit1), and king cobra (ophHan1) aligned to green anole (anoCar2). We also show the p-value from a one-sided Wilcoxon Rank Sum testing the hypothesis that the value of dN, dS, or dN/dS for the sex-determining genes (marked in red) is less than the rest of the genome (in gray). A total of 9,586 genes were used. 9,561 genes were defined as non-sex-determining and 25 were defined as sex-determining (as in Supplementary Information Table 17.4).

17.3 Discussion

Our analyses show significant deviation between patterns of molecular evolution at the codon level between tuatara and other reptiles and birds. Approximately 15% of the orthologs we tested had significantly different ω values on the tuatara branch relative to other birds and reptiles tested. Although none of these orthologs had ω values >1, suggestive of strong positive selection, the results do indicate that shifts in patterns of selection are affecting many genes and functional categories of genes across the tuatara genome.

Further, our analysis of sex-determining genes is consistent with an overall pattern of extreme conservation of genes involved in sex determination. Specifically, even with a biased subset of genes that align, and are conserved across species, including tuatara, sex-determining genes are especially conserved in terms of overall substitution rate, as well as in dN/dS. This suggests that they may be more likely to also be conserved in terms of function.

18 RECONSTRUCTION OF THE DEMOGRAPHIC HISTORY OF THE TUATARA

Stefan Prost*, Jose Grau* and Neil Gemmell

*Correspondence to: stefanprost.research@protonmail.com and jh.grau.jipoulou@gmail.com

18.1 Methods

Demographic history was inferred from the diploid sequence of our tuatara genome using a pairwise sequential Markovian coalescent (PSMC) method[287]. To do so we generated a diploid genome by mapping the raw read data back to the tuatara de novo assembly (QEPC00000000.1) using BWA mem[67] and called the diploid consensus sequence using Samtools mpileup[288]. We then conducted a range of preliminary analyses and found that PSMC plots were not sensitive to the values chosen for the maximum number of iterations (N), the number of free atomic time intervals (p), the maximum time to the most recent common ancestor (t), and the initial value of ρ. Based on these investigations, our final PSMC analyses used values of $N = 25$, $t = 5$, $\rho = 1$ and $p = 4 + 25 \times 2 + 4 + 6$ (data not shown). We determined the variance in estimates of Ne using 100 bootstrap replicates.

Plots of demographic history were scaled using a generation time of 30 years, slightly shorter than the 32–44 years previously used[289], together with estimates of mutation rates of 1.4 and $2\text{x}10^{-8}$ based on the divergence time between Anolis and tuatara and substitutions per site per generation obtained from our analysis of 4-fold degenerate sites (Supplementary Materials section 14).

18.2 Results

The reconstructed demographic histories did not vary substantially between the different values of p, t and ρ parameters. Using the 'optimal' settings we recovered a fluctuating population trend of repeated population increases and decreases. More specifically, we found an increase in effective population size (N_e) dating back to about 10 Mya ago, a drastic decrease in N_e about 1–3 Mya, and a subsequent rapid increase in N_e between 500 ka and 1 Mya.

18.3 Discussion

Our analyses show a significant fluctuation in effective population size, with increases detectable around 10 Mya, a decrease about 1–3 Mya, and a further rapid increase occurring between 500 kya and 1 Mya. These events correlate well with the known geological history of New Zealand (Zealandia), which experienced a pronounced marine transgression during the Oligocene (ca. 30–21 Mya)[290]. At its peak, ~23 Mya, Zealandia likely consisted of a series of small island masses, each separated by significant marine water bodies. Endemic terrestrial biota incapable of

dispersing across these major marine barriers, like the tuatara, would have been geographically isolated during this period, with populations only reconnecting as the landmasses subsequently enlarged and land bridges formed. The increase in effective population size at around 10 Mya may be reflective of the larger landmass and thus habitat available for tuatara during that period. The subsequent reduction in effective population size 1–3 Mya is consistent with a period of climatic cooling, which may have limited tuatara population size, while the rapid increase in effective population size 500 ka to 1 Mya seems strongly concordant with the formation of land bridges that connected the two main island land masses 1.5–2 Mya [291].

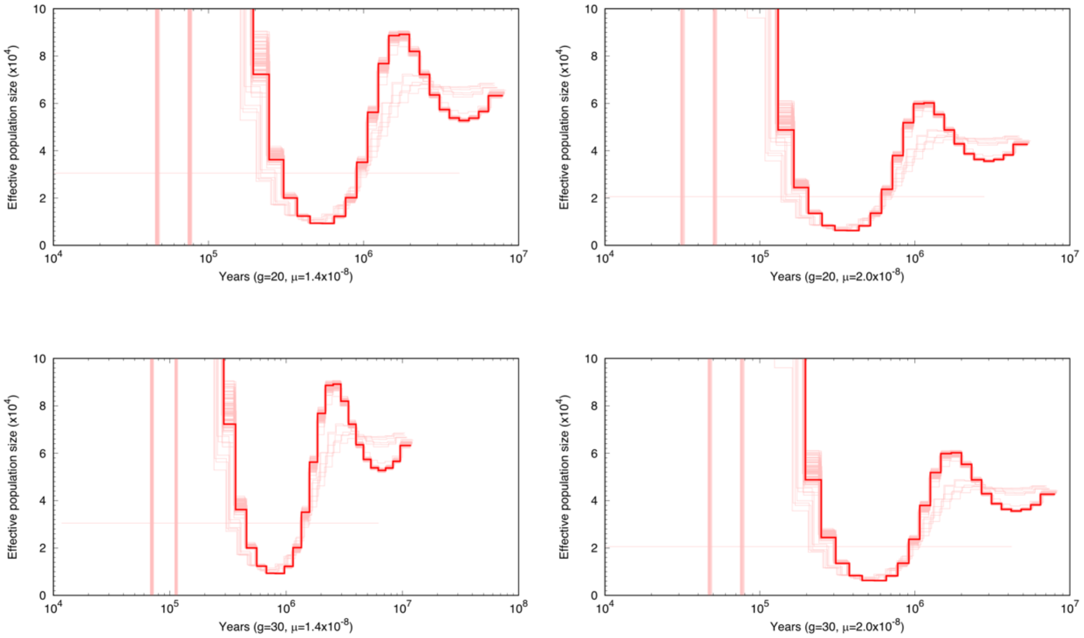

Supplementary Information Figure 18.1. PSMC plots of the demographic history of tuatara. Top left to bottom right generation and mutation rate vary. Top, generation time (g) is 20 years, Bottom, generation time (g) is 30 years. Left, substitutions per site per generation (μ) 1.4x10^{-}8, Right, substitutions per site per generation (μ) 2.0x10^{-}8.

19 POPULATION GENOMICS ANALYSES

David Winter*, Nicolas Dussex, Helen Taylor, Tracey Van Stijn, Shannon Clarke, Neil Gemmell*

*Correspondence to: d.winter@massey.ac.nz and neil.gemmell@otago.ac.nz

19.1 Methods

19.1.1 Sample collection and library preparation

We obtained 30 blood samples previously collected for animals from Little Barrier Island (Hauturu), Stephens Island (Takapourewa) and North Brother Island (Nga Whatu Kai Ponu) (Supplementary Information Table 19.1). Samples were collected under Victoria University of Wellington Animal Ethics approvals 2006R12; 2009R12; 2012R33; 22347 and held and used under permit 32037-RES issued by the New Zealand Department of Conservation. We extracted DNA using NucleoSpin Blood QuickPure columns (Macherey-Nagel) and evaluated DNA quality and concentration using both a Nanodrop 2000c spectrophotometer (Thermo Fisher Scientific) and a Fluorometric Quantitation Qubit system (Thermo Fisher Scientific). We undertook genotyping-by-sequencing (GBS)[292] on 28 samples where DNA quality and quantity were sufficient, using the restriction enzymes *Ape*KI and *Msp*I. GBS libraries were prepared as described previously[293], but we further purified the amplified library utilising a Pippin Prep (SAGE Science, Beverly, Massachusetts, United States) to select the DNA sequencing library in the size range of 150–500 bp. We performed single-end sequencing (1x100 bp) on an Illumina HiSeq2500 utilising v4 chemistry at AgResearch, Invermay, New Zealand, which yielded approximately 25Gb of raw sequence data per lane.

19.1.2 Read mapping and genotyping

Sequencing reads were de-multiplexed using GBSX[294] before being mapped to the tuatara genome with bwa mem[295]. Only reads with a mapping quality score > 30 that did not produce split or secondary alignments were used for downstream analysis. Because the results of GBS analysis can be sensitive to the particular analysis methods used, we took two separate approaches to calling single nucleotide variants (SNVs) from our data. We first used the reference mapping pipeline from Stacks 1.4.4[296], a software package designed for reduced-representation sequencing data. We also used the gatk haplotypecaller[297,298], a general-purpose variant caller that uses a more sophisticated genotyping model than stacks, to call SNVs. We filtered the gatk data to remove loci that were multiallelic, those with evidence for strand bias (odds ratio > 4) and those for which there was a significant difference in the quality scores of reads containing reference and alternative

alleles (z-score > 5). For both datasets we minimized the effects of allelic dropout by retaining only those loci that could be genotyped in at least four individuals in all three populations.

19.1.3 Functional annotation of SNVs

We used snpEFF[299] along with the functional annotation produced in this work to generate information from each SNV in the gatk dataset. We used pyVCF[300] to isolate SNVs falling in protein coding genes or close to putative mRNA splice sites. This subset of the data was then used to calculate alternative allele frequency spectra for both synonymous and non-synonymous variants in protein coding genes. We also examined the allele frequency of those SNVs that introduce an in-frame stop codon.

19.1.4 Population genomic analysis

We used vcftools[301] and the "population" program included in stacks to generate summary statistics for both the gatk and STACKS SNV dataset. Population differentiation was measured using Weir and Cockerham's estimator of F_{ST}[302], having first thinned each dataset to contain at most one SNV per 10kb of the reference genome. We calculated F_{ST} for the complete dataset and, additionally, for each pairwise combination of population-samples.

Our GBS experiment produced relatively low coverage alignments. For this reason, we incorporated the uncertainty associated with calling genotypes from low-coverage data[303] in our population genomic analysis by using genotype likelihood methods as implemented in angsd[304] and related software. We estimated a covariance matrix from genotype likelihoods using pcangsd[305], then used the R programming language[179] to perform a principal component analysis from this matrix. Admixture proportions for each individual were estimated using NGSadmix[304] with the value of K (the number of hypothetical populations from which individuals could inherit their genome) set between 2 and 4. Nucleotide diversity was calculated for each population using angsd.

19.1.5 Differentiation with respect to sex

We tested our SNV data for genetic differentiation with respect to the sex using the "population" program in STACKS. F_{ST} was calculated for each SNV while treating male and female tuatara as separate populations. The p-values obtained from the STACKS pipeline were transformed to q-values (i.e. controlled for multiple comparisons using a false-discovery rate approach) using the R function "p.adjust".

19.2 Results

19.2.1 Both SNV datasets support strong population structure

We generated a total of 22 gigabases of sequencing from our 28 individuals (Supplementary Information Table 19.1). The gatk haplotypecaller produced considerably more SNVs from this data than the stacks pipeline (Supplementary Information Table 19.2). This result arises from the higher depth-threshold required to call a site in stacks, which is also reflected in a much greater depth of coverage for called SNVs in the stacks dataset. The stacks dataset also has a much higher among-site variance in sequencing depth and more evidence for reference bias when compared to the gatk data. Both of these results suggest the stacks data may contain artifactual variant calls[306], possibly because the coverage thresholds used by this software can be expected to enrich the called-variants for multicopy and paralogous sequences when applied to low coverage datasets. For this reason we report only analyses from the gatk data in the main paper. Nevertheless, the evidence for strong population structure in modern tuatara populations is supported by both datasets ($F_{ST} = 0.42$ in the stacks data, 0.45 in the gatk data).

Pairwise F_{ST} estimates support the distinctiveness of the North Brother Island population, as reported in the main paper. The smallest pairwise F_{ST} was recorded between the Stephens and Little Barrier populations ($F_{ST} = 0.29$), while comparisons with the North Brother Island produced higher estimates ($F_{ST} = 0.51$ when combined with Little Barrier and 0.43 for Stephens).

While we acknowledge that the population genomic comparisons reported here use samples that are in many instances several decades old, our analyses likely closely reflect contemporary population structure for tuatara, even in the face of three decades of management. This is because this species is both long-lived and slow to reproduce[206], with many of the animals sampled decades ago known to still be alive and reproductive. Further, while translocation has been used to establish new populations, there have been no translocations among existing populations of tuatara in the past thirty years.

19.2.2 No loci are differentiated by sex

After correcting for multiple comparison no SNV was significantly differentiated with regard to sex (Supplementary Information Figure 19.1), the lowest q-value obtained for any locus was 0.41.

19.2.3 No evidence for excess genetic load in any population

The results presented in the body of this work demonstrate that modern tuatara populations have relatively low genetic diversity and have been isolated to small islands for thousands of years. This history of isolation may have implications for the management of remaining tuatara populations, as small isolated populations are prone to accumulating deleterious mutations. The population on North Brother Island / Nga Whatu Kai Ponu is of particular concern in this regard, given its low genetic diversity and the fact it is currently managed as a separate conservation unit. The generally

low coverage of our GBS data prevents us from estimating inbreeding coefficients, even using genotype likelihood approaches. We can, however, directly test for an excess of potentially damaging alleles in any population.

Non-synonymous mutations in protein coding genes are predominantly deleterious, so we started by considering the occurrence and frequency of synonymous and non-synonymous variants in coding regions across all populations. In total, 1101 (1.2%) of our called SNVs fall into a coding sequence. There are slightly fewer non-synonymous variants (518) than synonymous ones (572). Given these classes of variant arise in an approximately 3:1 ratio, this result suggests natural selection is at least partially preventing the accumulation of protein-altering mutations. When we consider the frequency of variants of each class in each population (Supplementary Information Figure 19.2), we find this result holds for alleles that are fixed within a population and for segregating variants.

SNVs that truncate a protein by introducing a premature stop codon in a protein coding gene are particularly likely to be deleterious. These SNVs are rare in our data, with only five such variants detected. Little Barrier / Hauturu is the only population to have more than two such alleles, and all four truncating alleles that occur in this population occur at a low frequency (Supplementary Information Figure 19.3)**.**

Although our GBS data only allows us to consider a small number of potentially damaging variants, we find no evidence for an excess of such variants in any population. The population from Nga Whatu Kai Ponu / North Brother Island has less genetic diversity than other populations we consider, but we find no evidence that it has accumulated protein-altering mutations at a greater rate than either of the more diverse populations.

Supplementary Information Table 19.1. Tuatara samples used for population genetic analysis via genotype-by sequencing and epigenetics. Samples in **BOLD** were not used for genotype-by-sequencing, while those samples in *Italics* were not used for post-bisulfite adapter tagging (PBAT) because of library failures.

Sample Name	ID	FT Number	Population	Sex	Status	Date Collected
SI IM	486	CD0516	Stephens Island	Male	adult	1/27/1984
SI 2M	487	CD0517	Stephens Island	Male	adult	1/28/1984
SI 3F	488	CD0518	Stephens Island	Female	adult	1/28/1984
SI 4F	490	CD0520	Stephens Island	Female	adult	1/28/1984
SI 5M	491	CD0521	Stephens Island	Male	adult	1/28/1984

SI 6F	492	CD0522	Stephens Island	Female	adult	1/28/1984
SI 7F	493	CD0523	Stephens Island	Female	adult	1/28/1984
SI 8M	494	CD0524	Stephens Island	Male	adult	1/29/1984
SI 9F	495	CD0525	Stephens Island	Female	adult	1/29/1984
SI 10M	496	CD0526	Stephens Island	Male	adult	1/29/1983
LBI 1M	2133	FT2925	Little Barrier Island	Male	adult	2/1/1991
LBI 2F	2134	FT2926	Little Barrier Island	Female	adult	2/1/1991
LBI 3M	2135	FT2927	Little Barrier Island	Male	adult	2/1/1991
LBI 4F	2136	FT2928	Little Barrier Island	Female	adult	2/1/1991
LBI 5M	2188	FT2980	Little Barrier Island	Male	adult	3/23/1991
LBI 6F	2189	FT2981	Little Barrier Island	Female	adult	3/23/1991
LBI 7M	2190	FT2982	Little Barrier Island	Male	adult	3/23/1991
LBI 8F	2191	FT2983	Little Barrier Island	Female	adult	3/23/1991
LBI 9F	2200	FT2992	Little Barrier Island	Female	adult	3/23/1991
LBI 10M	2201	FT2993	Little Barrier Island	Male	adult	3/23/1991
NBRO 1M	1025	FT0198	North Brother Island	Male	adult	1/21/1988
NBRO 2M	1026	FT0199	North Brother Island	Male	adult	8/21/1988
NBRO 3F	1033	FT0206	North Brother Island	Female	adult	3/21/1989
NBRO 4M	1034	FT0207	North Brother Island	Male	adult	10/21/1989
NBRO 5M	1035	FT0208	North Brother Island	Male	adult	5/21/1990
NBRO 6M	1036	FT0209	North Brother Island	Male	adult	12/21/1990
NBRO 7F	1039	FT0212	North Brother Island	Female	juvenile	7/21/1991
NBRO 8F	1042	FT0215	North Brother Island	Female	adult	2/21/1992
NBRO 9F	1051	FT0224	North Brother Island	Female	adult	9/21/1992
NBRO 10F	1053	FT0226	North Brother Island	Female	adult	4/21/1993

Supplementary Information Table 19.2 Summary statistics from SNV calling. "N" is the number of variable sites. "Ts:Tv" is the ratio of transitions to transversions in the dataset, "Depth" provides the mean total depth SNVs in each dataset, with variance across sites given in parentheses. "Reference freq." is the mean frequency of reads containing the reference allele at each site. "F_{ST}" is the mean value of Weir and Cockerham's estimator of this measure across all sites.

Dataset	N	Ts:Tv	Depth	Reference freq.	F_{ST}
STACKS	19379	2.82	83.7 (1771)	0.524	0.42
gatk	89256	2.79	47.6 (279)	0.502	0.45

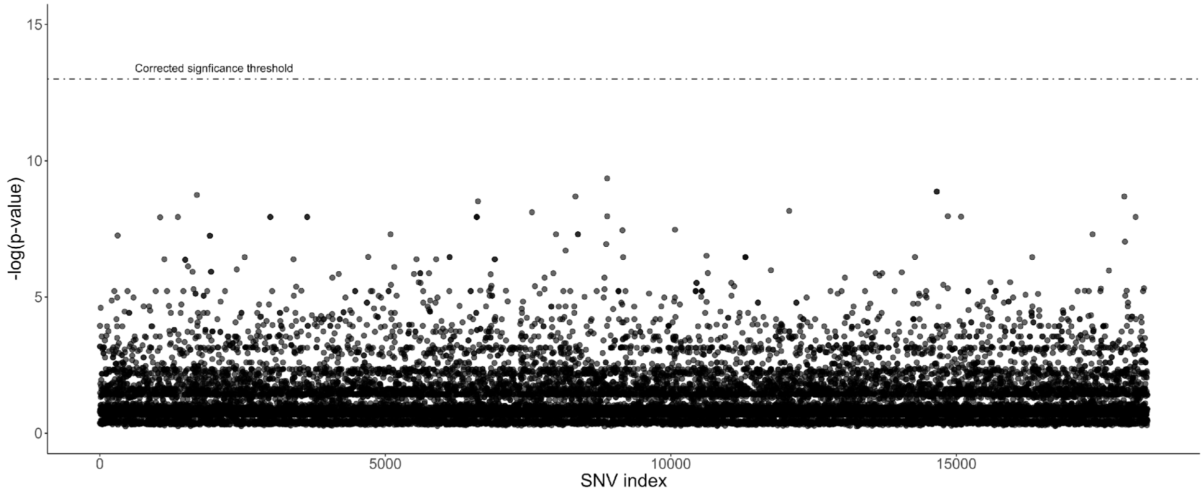

Supplementary Information Figure 19.1 No SNV is significantly differentiated with respect to sex. Each point represents a p-value from a test of population differentiation for a single SNV. In this case, the sex of individual tuatara was used in place of a population (n = 28, 13 males and 15 females, P-values calculated using Fishers Exact Test and corrected for multiple testing using the Bonferonni method). The dashed line represents the threshold for statistical significance after accounting for multiple testing. Note the y-axis is log-transformed.

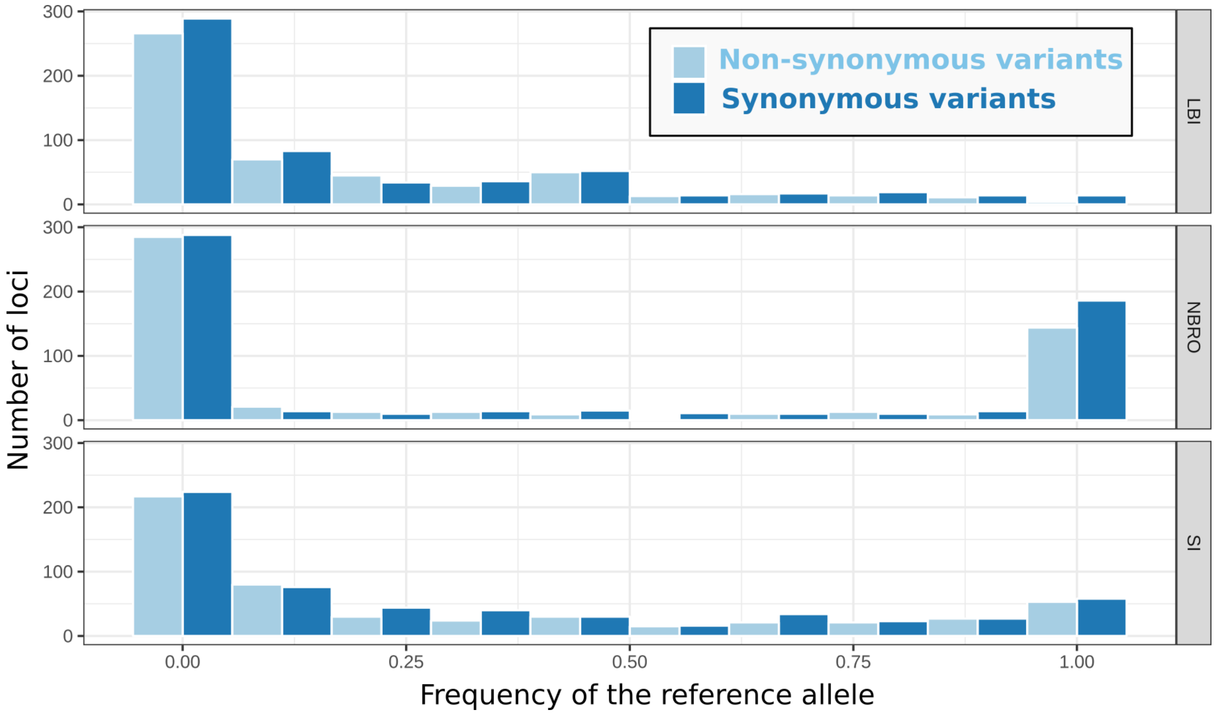

Supplementary Information Figure 19.2 No evidence for an excess of non-synonymous variants in any population. Each sub-plot displays the allele frequency spectrum of SNVs in protein coding regions of the tuatara genome from one population. LBI = Little Barrier Island, NBRO = North Brother, SI = Stephens Is. In each case the spectrum is plotted separately for synonymous (dark blue) and non-synonymous variants (light blue). Note that Hauturu/Little Barrier Island population is from Northern New Zealand, and thus likely more closely related to the reference individual, leading to more sites fixed for the reference allele and fewer fixed for an alternative allele.

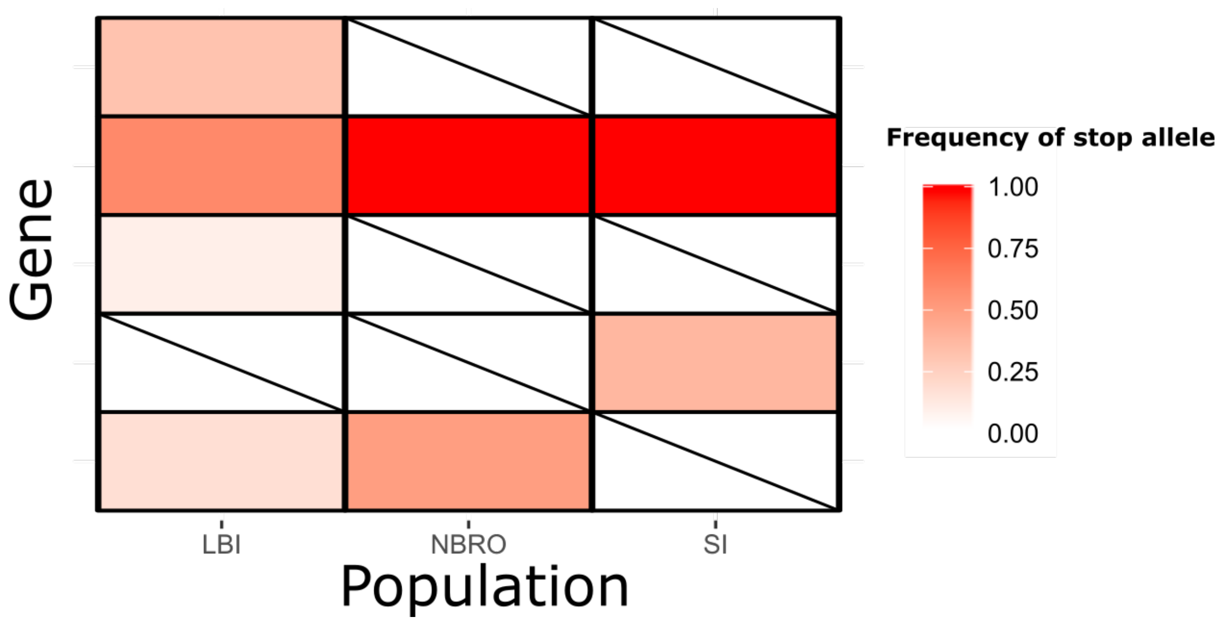

Supplementary Information Figure 19.3 Frequency of alleles that introduce a premature stop codon. Each row represents a distinct gene and each column a population. Cells are shaded to reflect frequency of the stop-codon allele in each population, cells with a diagonal line have an allele frequency of zero.

20 A CULTURAL DIMENSION

The tuatara is a taonga, or special treasure, for many Māori — notably Ngātiwai and Ngāti Koata who are the kaitiaki (guardians) of tuatara and are both claimants in the Waitangi Tribunal Wai 262 claim, also known as the flora and fauna claim. Our engagement of iwi with the tuatara project is consistent with the principles developed through that claim process. The tuatara genome project was undertaken in partnership with Ngātiwai iwi; our common goal is to increase knowledge and understanding of tuatara to aid the long-term conservation of this species, which while a taonga was also used in the recent past by Ngātiwai and other iwi as a form of personal cooling during harvesting of mutton birds (manu oi). Ngātiwai were involved in all decision-making regarding the use of the genome data by potential project partners; for each new project proposed introductions were made, and we subsequently discussed the benefits that might accrue from this work and how these could be shared with the iwi. The need to engage with, and protect the rights of, indigenous communities in such a transparent way has seldom been considered in the genomic projects published to date but is now a mandated consideration under the Convention for Biological Diversity's Nagoya Protocol on Access to Genetic Resources and the Fair and Equitable Sharing of Benefits Arising from their Utilization. Our partnership, which required significant time and investment from both parties since its initiation in 2011, is a step towards an inclusive model of genomic science that we hope others will adopt and improve upon. While each partnership is unique, we provide a template agreement that we hope will be useful to others.

20.1 GENOME RESEARCH AGREEMENT TEMPLATE

Between XXXXX and ZZZZZ

1.0 Preamble

1.1 XXXXX and ZZZZZ ("the Researcher"), are undertaking a collaborative research project to sequence the genome of _______, that will increase our knowledge and understanding of _______ ("the Project"). The expected outcome of this collaboration is the publication and release of the ______ genome sequence to the international scientific community.

1.2 _______ are located on lands or waters to which XXXXX holds traditional title or has cultural significance to XXXXX.

1.3 ______ are species of special significance to XXXXX and must be respected by all people granted access to them. They must be protected at all times for the present and future i generations.

1.4 It is acknowledged that ______________are providing funding or services in kind for the Project.

2.0 Purpose of Agreement

2.1 This Agreement sets out the terms and conditions for the Researcher participating in the Project. This Agreement will clearly identify the obligations of each of the parties towards meeting the Project outcomes.

3.0 Obligations of the Parties

3.1 XXXXXX will ensure that the following obligations are met:

a) Appoint individuals to oversee the coordination and management of the project. Their responsibilities will include interacting with, and instructing the research team of decisions of XXXXXX concerning the study.

b) Ensure that appropriate Communication Protocols will be followed.

c) Participate in and support the project (workshop discussions, feedback on the project and the information gathered)

d) Approve unpublished reports, media releases, peer-reviewed publications, presentations, photographs, and all results and information related to the research for distribution to XXXXXX and the general public

e) Provide guidance and advice and support to the Researcher on all cultural matters where assistance is required.

f) Approve the sending of DNA, tissue samples and data to collaborating overseas laboratories for genome sequencing, assembly and analysis. Such approval will not be unreasonably withheld.

3.2 The Researcher will ensure that they comply with the following conditions:

a) Act in good faith and work professionally while carrying out their required work with XXXXX.

b) Ensure that confidentiality is maintained when they conduct their research;

c) Ensure that XXXXX participants are provided with all necessary advice and guidance when interpreting scientific results obtained as part of the project.

d) Discuss all aspects of their proposed work with XXXXX and seek approval before any work is undertaken on the islands, or organic or non-organic material is removed from the islands.

e) Where proposed work has been amended, then the Researcher must notify XXXXX of the change and seek approval before work is undertaken.

f) The Researcher will inform XXXXX when reports are sent to funders, and will provide XXXXX with the full scientific research information contained within these reports.

g) The Researcher will consult with XXXXX prior to the sending of DNA, tissue samples and data to collaborating overseas laboratories for genome sequencing, assembly and analysis.

h) Where other parties may wish to contribute to the project or obtain access to the data prior to its release into the public domain, such arrangements will be developed in consultation with XXXXX and further research agreements may be negotiated where appropriate.

i) Any information relating to XXXXX, and the cultural practices to which that knowledge relates, obtained by the Researcher remains the intellectual property of XXXXX.

j) The Researcher will not engage with or use XXXXX information or resources without seeking written consent to do so.

k) It is agreed by the Researcher that the aim of this research is to sequence the genome of _______ and not commercialisation resulting from this genome sequencing. However, should the researchers believe there may be potential for further research leading to commercialisation they must first consult with XXXXX for approval and for discussion on a benefit sharing agreement.

4.0 Access to Information / Release of Scientific Results

4.1 The first point of contact for any results and interpretations from the study will be the individuals appointed by XXXXX to oversee the coordination and management of the project

4.2 XXXXX will be responsible for reviewing and providing prior written consultation, which may be given by email by the project coordinator appointed by XXXXX, for the release of research results and information, traditional knowledge, meeting minutes, general dialogue, and photographs in peer-reviewed publications, popular articles, interviews, presentations, and any other form of media.

4.3 XXXXX members will provide feedback regarding the release of all research results and information, traditional knowledge, meeting minutes, general dialogue, and photographs in peer-reviewed publications, popular articles, interviews and presentations to the appointed project coordinator. The project coordinator appointed by XXXXX will notify the Researcher of XXXXX feedback within two weeks.

4.4 The Researcher must be willing to report the results of the research at meetings, annual Hui or any other gathering as directed by XXXXX.

5.0 Media

5.1 No statements are to be made to the media, Department of Conservation or any other agency without the Parties consulting with each other over this first.

5.2 Any information contained in reports, media releases and articles that has previously been approved for release and is therefore already in the public domain, is no longer subject to clause 5.1.

6.0 Confidentiality

6.1 Information disclosed by one party to the other concerning the Project and the arrangements between the Parties that are detailed in this Agreement are confidential.

6.2 Neither Party will disclose any confidential information to any third party, other than funders and approved collaborators without the express

agreement of the other Party. This agreement will not be unreasonably withheld.

6.3 The Parties agree that confidential information received under this Agreement will not be used for any purpose other than carrying out the Project and any future subcontract or research agreements relating to the Project.

6.4 This confidentiality provision will survive the expiration or termination of this Agreement and shall be legally enforceable by one Party against the other, until that confidential information enters the public domain.

7.0 Breach of terms and conditions of this Agreement

7.1 Where any terms within this agreement have been breached, then the following process will be followed:

a) XXXXX will be notified of the nature of the breach;

b) The Researcher or nominee will be asked to attend a meeting with XXXXX or its representatives to discuss the incident;

c) XXXXX will determine the severity of the breach in consultation with ________ and then discuss the course of action to be taken;

d) The Researcher will then be notified of the outcome.

8.0 Dispute Resolution

8.1 Any dispute concerning the subject matter of this Agreement will be settled by full and frank discussion and negotiation between Parties. These discussions will take place kanohi ki te kanohi (face to face) and be conducted in a manner that is non-threatening and nurtures resolving of issues.

8.2 Should the dispute not be resolved satisfactorily by these means, the Parties agree that they will engage in mediation conducted in accordance with the terms and conditions of the LEADR New Zealand Inc Standard Mediation Agreement.

Signed for and on behalf of XXXXX:

Signature: Date:

Name: Position:

In the presence of:

Name of witness:

Signature:

Signed for and on behalf of ZZZZZ:

Signature: Date:

Name: Position:

In the presence of:

Name of witness:

Signature:

Read and Understood:

Signature: Date:

Name: Position:

21 REFERENCES

36. Gnerre, S. *et al.* High-quality draft assemblies of mammalian genomes from massively parallel sequence data. *Proc. Natl. Acad. Sci. U. S. A.* **108**, 1513–1518 (2011).
37. Luo, R. *et al.* SOAPdenovo2: An empirically improved memory-efficient short-read de novo assembler. *Gigascience* **1**, 18 (2012).
38. Xue, W. *et al.* L_RNA_scaffolder: Scaffolding genomes with transcripts. *BMC Genomics* **14**, 604 (2013).
39. Putnam, N. H. *et al.* Chromosome-scale shotgun assembly using an in vitro method for long-range linkage. *Genome Res.* **26**, 342–350 (2016).
40. Todd, E. V., Black, M. A. & Gemmell, N. J. The power and promise of RNA-seq in ecology and evolution. *Mol. Ecol.* **25**, 1224–1241 (2016).
41. Miller, H. C., Biggs, P. J., Voelckel, C. & Nelson, N. J. De novo sequence assembly and characterisation of a partial transcriptome for an evolutionarily distinct reptile, the tuatara (*Sphenodon punctatus*). *BMC Genomics* **13**, 439 (2012).
42. Grabherr, M. G. *et al.* Full-length transcriptome assembly from RNA-Seq data without a reference genome. *Nat. Biotechnol.* **29**, 644–652 (2011).
43. Haas, B. J. *et al.* De novo transcript sequence reconstruction from RNA-seq using the Trinity platform for reference generation and analysis. *Nat. Protoc.* **8**, 1494–1512 (2013).
44. Li, W. & Godzik, A. Cd-hit: A fast program for clustering and comparing large sets of protein or nucleotide sequences. *Bioinformatics* **22**, 1658–1659 (2006).
45. Peat, J. R., Ortega-Recalde, O., Kardailsky, O. & Hore, T. A. The elephant shark methylome reveals conservation of epigenetic regulation across jawed vertebrates. *F1000Res.* **6**, 526 (2017).

46. Krueger, F. & Andrews, S. R. Bismark: A flexible aligner and methylation caller for Bisulfite-Seq applications. *Bioinformatics* **27**, 1571–1572 (2011).

47. Smit, A. & Hubley, R. *RepeatModeler Open-1.0. 2008–2015*. (2015).

48. Zeng, L., Kortschak, R. D., Raison, J. M., Bertozzi, T. & Adelson, D. L. Superior ab initio identification, annotation and characterisation of TEs and segmental duplications from genome assemblies. *PLOS One* **13**, e0193588 (2018).

49. Ellinghaus, D., Kurtz, S. & Willhoeft, U. LTRharvest, an efficient and flexible software for de novo detection of LTR retrotransposons. *BMC Bioinformatics* **9**, 18 (2008).

50. Smit, A. F. A., Hubley, R. & Green, P. *RepeatMasker Open-4.0. 2013–2015*. (2015).

51. Holt, C. & Yandell, M. MAKER2: An annotation pipeline and genome-database management tool for second-generation genome projects. *BMC Bioinformatics* **12**, 491 (2011).

52. Korf, I. Gene finding in novel genomes. *BMC Bioinformatics* **5**, 59 (2004).

53. Stanke, M. & Waack, S. Gene prediction with a hidden Markov model and a new intron submodel. *Bioinformatics* **19** Suppl 2, ii215–25 (2003).

54. Simão, F. A., Waterhouse, R. M., Ioannidis, P., Kriventseva, E. V. & Zdobnov, E. M. BUSCO: Assessing genome assembly and annotation completeness with single-copy orthologs. *Bioinformatics* **31**, 3210–3212 (2015).

55. Kalvari, I. *et al.* Rfam 13.0: shifting to a genome-centric resource for non-coding RNA families. *Nucleic Acids Res* **46**, 335–342 (2018).

56. Nawrocki, E. P. & Eddy, S. R. Infernal 1.1: 100-fold faster RNA homology searches. *Bioinformatics* **29**, 2933–2935 (2013).

57. Lowe, T. M. & Eddy, S. R. tRNAscan-SE: A program for improved detection of transfer

RNA genes in genomic sequence. *Nucleic Acids Res.* **25**, 955–964 (1997).

58. Herrero, J. *et al.* Ensembl comparative genomics resources. *Database* **2016**, bav096 (2016).
59. Federhen, S. The NCBI Taxonomy database. *Nucleic Acids Res.* **40**, D136–43 (2012).
60. Severin, J. *et al.* eHive: An artificial intelligence workflow system for genomic analysis. *BMC Bioinformatics* **11**, 240 (2010).
61. Kristensen, D. M., Wolf, Y. I., Mushegian, A. R. & Koonin, E. V. Computational methods for gene orthology inference. *Brief. Bioinform.* **12**, 379–391 (2011).
62. Aken, B. L. *et al.* The Ensembl gene annotation system. *Database* 2016, baw093 (2016).
63. The UniProt Consortium. UniProt: The universal protein knowledgebase. *Nucleic Acids Res.* **46**, 2699 (2018).
64. Morgulis, A., Gertz, E. M., Schäffer, A. A. & Agarwala, R. A fast and symmetric DUST implementation to mask low-complexity DNA sequences. *J. Comput. Biol.* **13**, 1028–1040 (2006).
65. She, R. *et al.* genBlastG: Using BLAST searches to build homologous gene models. *Bioinformatics* **27**, 2141–2143 (2011).
66. Collins, J. E., White, S., Searle, S. M. J. & Stemple, D. L. Incorporating RNA-seq data into the zebrafish Ensembl genebuild. *Genome Res.* (2012) doi:10.1101/gr.137901.112.
67. Li, H. & Durbin, R. Fast and accurate short read alignment with Burrows–Wheeler transform. *Bioinformatics* **25**, 1754–1760 (2009).
68. Slater, G. S. C. & Birney, E. Automated generation of heuristics for biological sequence comparison. *BMC Bioinformatics* **6**, 31 (2005).
69. Altschul, S. F., Gish, W., Miller, W., Myers, E. W. & Lipman, D. J. Basic local alignment search tool. *J. Mol. Biol.* **215**, 403–410 (1990).

70. Edgar, R. C. MUSCLE: Multiple sequence alignment with high accuracy and high throughput. *Nucleic Acids Res.* **32**, 1792–1797 (2004).

71. Nawrocki, E. P. *et al.* Rfam 12.0: Updates to the RNA families database. *Nucleic Acids Res.* **43**, D130–7 (2015).

72. Bailey, J. A., Yavor, A. M., Massa, H. F., Trask, B. J. & Eichler, E. E. Segmental duplications: Organization and impact within the current human genome project assembly. *Genome Res.* **11**, 1005–1017 (2001).

73. Conrad, B. & Antonarakis, S. E. Gene duplication: A drive for phenotypic diversity and cause of human disease. *Annu. Rev. Genomics Hum. Genet.* **8**, 17–35 (2007).

74. Sharp, A. J. *et al.* Segmental duplications and copy-number variation in the human genome. *Am. J. Hum. Genet.* **77**, 78–88 (2005).

75. Teichmann, S. A. & Babu, M. M. Gene regulatory network growth by duplication. *Nat. Genet.* **36**, 492–496 (2004).

76. Wan, Q. H. *et al.* Genome analysis and signature discovery for diving and sensory properties of the endangered Chinese alligator. *Cell Res.* **23**, 1091–1105 (2013).

77. Alföldi, J. *et al.* The genome of the green anole lizard and a comparative analysis with birds and mammals. *Nature* **477**, 587–591 (2011).

78. Hillier, L. W. *et al.* Sequence and comparative analysis of the chicken genome provide unique perspectives on vertebrate evolution. *Nature* **432**, 695–716 (2004).

79. Warren, W. C. *et al.* The genome of a songbird. *Nature* **464**, 757–762 (2010).

80. St John, J. A. *et al.* Sequencing three crocodilian genomes to illuminate the evolution of archosaurs and amniotes. *Genome Biol.* **13**, 415 (2012).

81. Shaffer, H. B. *et al.* The western painted turtle genome, a model for the evolution of

extreme physiological adaptations in a slowly evolving lineage. *Genome Biol.* **14**, R28 (2013).

82. Lovsin, N., Gubensek, F. & Kordi, D. Evolutionary dynamics in a novel L2 clade of non-LTR retrotransposons in Deuterostomia. *Mol. Biol. Evol.* **18**, 2213–2224 (2001).
83. Lander, E. S. *et al.* Initial sequencing and analysis of the human genome. *Nature* **409**, 860–921 (2001).
84. Warren, W. C. *et al.* Genome analysis of the platypus reveals unique signatures of evolution. *Nature* **453**, 175–183 (2008).
85. Ivancevic, A. M., Walsh, A. M., Kortschak, R. D. & Adelson, D. L. Jumping the fine LINE between species: Horizontal transfer of transposable elements in animals catalyses genome evolution. *Bioessays* **35**, 1071–1082 (2013).
86. Walsh, A. M., Kortschak, R. D., Gardner, M. G., Bertozzi, T. & Adelson, D. L. Widespread horizontal transfer of retrotransposons. *Proc. Natl. Acad. Sci. U. S. A.* **110**, 1012–1016 (2013).
87. Zupunski, V., Gubensek, F. & Kordis, D. Evolutionary dynamics and evolutionary history in the RTE clade of non-LTR retrotransposons. *Mol. Biol. Evol.* **18**, 1849–1863 (2001).
88. Sormacheva, I. *et al.* Vertical evolution and horizontal transfer of CR1 non-LTR retrotransposons and Tc1/mariner DNA transposons in Lepidoptera species. *Mol. Biol. Evol.* **29**, 3685–3702 (2012).
89. Helgen, K. M., Miguez, R. P., James, L. K. & Lauren, E. H. Twentieth century occurrence of the long-beaked echidna *Zaglossus bruijnii* in the Kimberley region of Australia. *Zookeys* 103–132 (2012).
90. Messer, M., Weiss, A. S., Shaw, D. C. & Westerman, M. Evolution of the monotremes:

Phylogenetic relationship to marsupials and eutherians, and estimation of divergence dates based on α-lactalbumin amino acid sequences. *J. Mamm. Evol.* **5**, 95–105 (1998).

91. McKenna, M. C. The vertebrates updated: Vertebrate paleontology and evolution. *Science* **239**, 512–513 (1988).

92. Jones, M. E., Tennyson, A. J., Worthy, J. P., Evans, S. E. & Worthy, T. H. A sphenodontine (Rhynchocephalia) from the Miocene of New Zealand and palaeobiogeography of the tuatara (*Sphenodon*). *Proc. Biol. Sci.* **276**, 1385–1390 (2009).

93. Spellerberg, I. F. & Sawyer, J. W. D. *An introduction to applied biogeography*. (Cambridge University Press, 1999).

94. Edgar, R. C. & Myers, E. W. PILER: Identification and classification of genomic repeats. *Bioinformatics* **21** Suppl 1, i152–8 (2005).

95. Kortschak, R. D. & Adelson, D. L. bíogo: A simple high-performance bioinformatics toolkit for the Go language. *bioRxiv* 005033 (2014).

96. Kohany, O., Gentles, A. J., Hankus, L. & Jurka, J. Annotation, submission and screening of repetitive elements in Repbase: RepbaseSubmitter and Censor. *BMC Bioinformatics* **7**, 474 (2006).

97. Wheeler, D. L. *et al.* Database resources of the National Center for Biotechnology Information. *Nucleic Acids Res.* **35**, D5–12 (2007).

98. Price, M. N., Dehal, P. S. & Arkin, A. P. FastTree: Computing large minimum evolution trees with profiles instead of a distance matrix. *Mol. Biol. Evol.* **26**, 1641–1650 (2009).

99. Rambaut, A. *FigTree v1. 4*. (2012).

100. Edgar, R. C. Search and clustering orders of magnitude faster than BLAST. *Bioinformatics* **26**, 2460–2461 (2010).

101. Finn, R. D., Clements, J. & Eddy, S. R. HMMER web server: Interactive sequence similarity searching. *Nucleic Acids Res.* **39**, W29–37 (2011).

102. Punta, M. *et al.* The Pfam protein families database. *Nucleic Acids Res.* **40**, D290–301 (2012).

103. Ronquist, F. & Huelsenbeck, J. P. MrBayes 3: Bayesian phylogenetic inference under mixed models. *Bioinformatics* **19**, 1572–1574 (2003).

104. Wickham, H. *ggplot2: elegant graphics for data analysis*. (Springer, 2016).

105. Knudsen, S. Promoter2.0: For the recognition of PolII promoter sequences. *Bioinformatics* **15**, 356–361 (1999).

106. Bailey, T. L. *et al.* MEME SUITE: Tools for motif discovery and searching. *Nucleic Acids Res.* **37**, W202–8 (2009).

107. Dasmahapatra, K. K. *et al.* Butterfly genome reveals promiscuous exchange of mimicry adaptations among species. *Nature* **487**, 94–98 (2012).

108. Green, R. E. *et al.* Three crocodilian genomes reveal ancestral patterns of evolution among archosaurs. *Science* **346**, 1335 (2014).

109. Katoh, K. & Toh, H. Recent developments in the MAFFT multiple sequence alignment program. *Brief. Bioinform.* **9**, 286–298 (2008).

110. Suh, A., Smeds, L. & Ellegren, H. Abundant recent activity of retrovirus-like retrotransposons within and among flycatcher species implies a rich source of structural variation in songbird genomes. *Mol. Ecol.* **27**, 99–111 (2018).

111. Jurka, J. *et al.* Repbase Update, a database of eukaryotic repetitive elements. *Cytogenet. Genome Res.* **110**, 462–467 (2005).

112. Smit, A., Hubley, R. & Green, P. RepeatMasker Open-3.3.0. http://www.repeatmasker.org.

(1996–2010).

113. Suh, A. *et al.* Multiple lineages of ancient CR1 retroposons shaped the early genome evolution of amniotes. *Genome Biol. Evol.* **7**, 205–217 (2015).

114. Stamatakis, A., Hoover, P. & Rougemont, J. A rapid bootstrap algorithm for the RAxML web servers. *Syst. Biol.* **75**, 758–771 (2008).

115. Miller, M. A., Pfeiffer, W. & Schwartz, T. Creating the CIPRES Science Gateway for inference of large phylogenetic trees. *Proceedings of the Gateway Computing Environments Workshop (GCE)* 1–8 (2010).

116. Feschotte, C. & Pritham, E. J. DNA transposons and the evolution of eukaryotic genomes. *Annu. Rev. Genet.* **41**, 331–368 (2007).

117. Ohshima, K. & Okada, N. SINEs and LINEs: Symbionts of eukaryotic genomes with a common tail. *Cytogenet. Genome Res.* **110**, 475–490 (2005).

118. Suh, A. The specific requirements for CR1 retrotransposition explain the scarcity of retrogenes in birds. *J. Mol. Evol.* **81**, 18–20 (2015).

119. Ohshima, K., Hamada, M., Terai, Y. & Okada, N. The 3’ ends of tRNA-derived short interspersed repetitive elements are derived from the 3' ends of long interspersed repetitive elements. *Mol. Cell. Biol.* **16**, 3756–3764 (1996).

120. Bejerano, G. *et al.* A distal enhancer and an ultraconserved exon are derived from a novel retroposon. *Nature* **441**, 87–90 (2006).

121. Hirakawa, M., Nishihara, H., Kanehisa, M. & Okada, N. Characterization and evolutionary landscape of AmnSINE1 in Amniota genomes. *Gene* **441**, 100–110 (2009).

122. Nishihara, H., Smit, A. F. A. & Okada, N. Functional noncoding sequences derived from SINEs in the mammalian genome. *Genome Res.* **16**, 864–874 (2006).

123. Jurka, J., Zietkiewicz, E. & Labuda, D. Ubiquitous mammalian-wide interspersed repeats (MIRs) are molecular fossils from the mesozoic era. *Nucleic Acids Res.* **23**, 170–175 (1995).

124. Smit, A. F. A. & Riggs, A. D. MIRs are classic, tRNA-derived SINEs that amplified before the mammalian radiation. *Nucleic Acids Res.* **23**, 98–102 (1995).

125. Warren, W. C. *et al.* Genome analysis of the platypus reveals unique signatures of evolution. *Nature* **453**, 175–183 (2008).

126. Mikkelsen, T. S. *et al.* Genome of the marsupial *Monodelphis domestica* reveals innovation in non-coding sequences. *Nature* **447**, 167–177 (2007).

127. Deininger, P. L. & Batzer, M. A. Mammalian retroelements. *Genome Res.* **12**, 1455–1465 (2002).

128. Rebollo, R., Romanish, M. T. & Mager, D. L. Transposable elements: an abundant and natural source of regulatory sequences for host genes. *Annu. Rev. Genet.* **46**, 21–42 (2012).

129. Wicker, T. *et al.* A unified classification system for eukaryotic transposable elements. *Nat. Rev. Genet.* **8**, 973–982 (2007).

130. Kapitonov, V. V. & Jurka, J. A universal classification of eukaryotic transposable elements implemented in Repbase. *Nat. Rev. Genet.* **9**, 411–412 (2008).

131. Eickbush, T. H. & Jamburuthugoda, V. K. The diversity of retrotransposons and the properties of their reverse transcriptases. *Virus Res.* **134**, 221–234 (2008).

132. Llorens, C., Munoz-Pomer, A., Bernad, L., Botella, H. & Moya, A. Network dynamics of eukaryotic LTR retroelements beyond phylogenetic trees. *Biol. Direct* **4**, 41 (2009).

133. Kazazian, H. H., Jr. Mobile elements: Drivers of genome evolution. *Science* **303**, 1626–1632 (2004).

134. Magiorkinis, G., Gifford, R. J., Katzourakis, A., De Ranter, J. & Belshaw, R. Env-less endogenous retroviruses are genomic superspreaders. *Proc. Natl. Acad. Sci. U. S. A.* **109**, 7385–7390 (2012).

135. Havecker, E. R., Gao, X. & Voytas, D. F. The diversity of LTR retrotransposons. *Genome Biol.* **5**, 225 (2004).

136. de la Chaux, N. & Wagner, A. BEL/Pao retrotransposons in metazoan genomes. *BMC Evol. Biol.* **11**, 154 (2011).

137. Llorens, C. *et al.* The Gypsy Database (GyDB) of mobile genetic elements: Release 2.0. *Nucleic Acids Res.* **39**, D70–4 (2011).

138. Lerat, E. Identifying repeats and transposable elements in sequenced genomes: How to find your way through the dense forest of programs. *Heredity* **104**, 520–533 (2010).

139. Rho, M. *et al.* LTR retroelements in the genome of *Daphnia pulex*. *BMC Genomics* **11**, 425 (2010).

140. Tollis, M. & Boissinot, S. The transposable element profile of the anolis genome: How a lizard can provide insights into the evolution of vertebrate genome size and structure. *Mob. Genet. Elements* **1**, 107–111 (2011).

141. Aiewsakun, P. & Katzourakis, A. Marine origin of retroviruses in the early Palaeozoic Era. *Nat. Commun.* **8**, 13954 (2017).

142. Hayward, A., Cornwallis, C. K. & Jern, P. Pan-vertebrate comparative genomics unmasks retrovirus macroevolution. *Proc. Natl. Acad. Sci. U. S. A.* **112**, 464–469 (2015).

143. Han, G.-Z. & Worobey, M. An endogenous foamy-like viral element in the coelacanth genome. *PLOS Pathog.* **8**, e1002790 (2012).

144. Katzourakis, A., Gifford, R. J., Tristem, M., Gilbert, M. T. P. & Pybus, O. G.

Macroevolution of complex retroviruses. *Science* **325**, 1512 (2009).

145. Han, G.-Z. & Worobey, M. An endogenous foamy virus in the aye-aye (*Daubentonia madagascariensis*). *J. Virol.* **86**, 7696–7698 (2012).

146. Lee, A., Nolan, A., Watson, J. & Tristem, M. Identification of an ancient endogenous retrovirus, predating the divergence of the placental mammals. *Philos. Trans. R. Soc. Lond. B Biol. Sci.* **368**, 20120503 (2013).

147. McClure, M. A., Johnson, M. S., Feng, D. F. & Doolittle, R. F. Sequence comparisons of retroviral proteins: Relative rates of change and general phylogeny. *Proc. Natl. Acad. Sci. U. S. A.* **85**, 2469–2473 (1988).

148. Gremme, G., Steinbiss, S. & Kurtz, S. GenomeTools: A comprehensive software library for efficient processing of structured genome annotations. *IEEE/ACM Trans. Comput. Biol. Bioinform.* **10**, 645–656 (2013).

149. Fu, L., Niu, B., Zhu, Z., Wu, S. & Li, W. CD-HIT: Accelerated for clustering the next-generation sequencing data. *Bioinformatics* vol. 28 3150–3152 (2012).

150. Katoh, K. & Standley, D. M. MAFFT multiple sequence alignment software version 7: improvements in performance and usability. *Mol. Biol. Evol.* **30**, 772–780 (2013).

151. Stamatakis, A. RAxML version 8: A tool for phylogenetic analysis and post-analysis of large phylogenies. *Bioinformatics* **30**, 1312–1313 (2014).

152. Wheeler, T. J. Large-scale neighbor-joining with NINJA. in *Algorithms in Bioinformatics. WABI 2009* (ed. Salzberg S. L., W. T.) vol. 5724 375–389 (Springer Berlin Heidelberg, 2009).

153. Eddy, S. R. Non-coding RNA genes and the modern RNA world. *Nat. Rev. Genet.* **2**, 919–929 (2001).

154. Hoeppner, M. P., Denisenko, E., Gardner, P. P., Schmeier, S. & Poole, A. M. An evaluation of function of multicopy non-coding RNAs in mammals using ENCODE/FANTOM data and comparative genomics. *Mol. Biol. Evol.* (2018) doi:10.1093/molbev/msy046.

155. Eddy, S. R. & Durbin, R. RNA sequence analysis using covariance models. *Nucleic Acids Res.* **22**, 2079–2088 (1994).

156. Sakakibara, Y. *et al.* Stochastic context-free grammars for tRNA modeling. *Nucleic Acids Res.* **22**, 5112–5120 (1994).

157. Freyhult, E. K., Bollback, J. P. & Gardner, P. P. Exploring genomic dark matter: A critical assessment of the performance of homology search methods on noncoding RNA. *Genome Res.* **17**, 117–125 (2007).

158. Lowe, T. M. & Chan, P. P. tRNAscan-SE On-line: Integrating search and context for analysis of transfer RNA genes. *Nucleic Acids Res.* **44**, W54–7 (2016).

159. Gardner, P. P. *et al.* Rfam: Wikipedia, clans and the ‘decimal’ release. *Nucleic Acids Res.* **39**, D141–D145 (2011).

160. Gardner, P. P. *et al.* Conservation and losses of non-coding RNAs in avian genomes. *PLOS One* **10**, e0121797 (2015).

161. Jabbari, K. & Bernardi, G. Cytosine methylation and CpG, TpG (CpA) and TpA frequencies. *Gene* **333**, 143–149 (2004).

162. Watt, F. & Molloy, P. L. Cytosine methylation prevents binding to DNA of a hela-cell transcription factor required for optimal expression of the adenovirus major late promoter. *Genes Dev.* **2**, 1136–1143 (1988).

163. Boyes, J. & Bird, A. DNA methylation inhibits transcription indirectly via a methyl-CpG binding-protein. *Cell* **64**, 1123–1134 (1991).

164. Hendrich, B. & Bird, A. Identification and characterization of a family of mammalian methyl-CpG binding proteins. *Mol. Cell. Biol.* **18**, 6538–6547 (1998).

165. Varriale, A. DNA methylation, epigenetics, and evolution in vertebrates: Facts and challenges. *Int. J. Evol. Biol.* **2014**, 475981–475981 (2014).

166. Bergman, Y. & Cedar, H. DNA methylation dynamics in health and disease. *Nat. Struct. Mol. Biol.* **20**, 274–281 (2013).

167. Cantone, I. & Fisher, A. G. Epigenetic programming and reprogramming during development. *Nat. Struct. Mol. Biol.* **20**, 282–289 (2013).

168. Head, J. A. Patterns of DNA methylation in animals: An ecotoxicological perspective. *Integr. Comp. Biol.* **54**, 77–86 (2014).

169. Zemach, A., McDaniel, I. E., Silva, P. & Zilberman, D. Genome-wide evolutionary analysis of eukaryotic DNA methylation. *Science* **328**, 916–919 (2010).

170. Yang, H., Li, D. & Cheng, C. Relating gene expression evolution with CpG content changes. *BMC Genomics* **15**, (2014).

171. Elango, N., Hunt, B. G., Goodisman, M. A. D. & Yi, S. V. DNA methylation is widespread and associated with differential gene expression in castes of the honeybee, *Apis mellifera*. *Proc. Natl. Acad. Sci. U. S. A.* **106**, 11206–11211 (2009).

172. Coulondre, C., Miller, J. H., Farabaugh, P. J. & Gilbert, W. Molecular basis of base substitution hotspots in *Escherichia coli*. *Nature* **274**, 775–780 (1978).

173. Shen, J. C., Rideout, W. M. & Jones, P. A. The rate of hydrolytic deamination of 5-methylcytosine in double-stranded DNA. *Nucleic Acids Res.* **22**, 972–976 (1994).

174. Jabbari, K., Caccio, S., de Barros, J. P. P., Desgres, J. & Bernardi, G. Evolutionary changes in CpG and methylation levels in the genome of vertebrates. *Gene* **205**, 109–118 (1997).

175. Bird, A. P. DNA methylation and the frequency of CpG in animal DNA. *Nucleic Acids Res.* **8**, 1499–1504 (1980).

176. Elango, N. & Yi, S. V. DNA methylation and structural and functional bimodality of vertebrate promoters. *Mol. Biol. Evol.* **25**, 1602–1608 (2008).

177. Radhakrishnan, S., Literman, R., Mizoguchi, B. A. & Valenzuela, N. MeDIPseq and nCpG analyses illuminate sexually dimorphic methylation of gonadal development genes with high historic methylation in turtle hatchlings with temperature-dependent sex determination. *Epigenetics Chromatin* **10**, 1–16 (2017).

178. Quinlan, A. R. & Hall, I. M. BEDTools: A flexible suite of utilities for comparing genomic features. *Bioinformatics* **26**, 841–842 (2010).

179. R Core Team. *R: A language and environment for statistical computing. R Foundation for Statistical Computing, Vienna, Austria. ISBN 3-900051-07-0, URL http://www.R-project.org*. (2016).

180. Fraley, C. & Raftery, A. *MCLUST version 3 for R: Normal mixture modeling and model-based clustering*. http://www.stat.washington.edu/mclust/ (2006).

181. Radhakrishnan, S., Literman, R., Mizoguchi, B. A. & Valenzuela, N. MeDIPseq and nCpG analyses illuminate sexually dimorphic methylation of gonadal development genes with high historic methylation in turtle hatchlings with temperature-dependent sex determination. *Epigenetics Chromatin* **10**, 1–16 (2017).

182. Keller, T. E., Han, P. & Yi, S. V. Evolutionary transition of promoter and gene body DNA methylation across invertebrate–vertebrate boundary. *Mol. Biol. Evol.* **33**, 1019–1028 (2016).

183. Miller, H. C. *et al.* Major histocompatibility complex genes map to two chromosomes in an

evolutionarily ancient reptile, the tuatara *Sphenodon punctatus*. *G3* **5**, 1439–1451 (2015).

184. Kaufman, J. *et al.* The chicken B locus is a minimal essential major histocompatibility complex. *Nature* **401**, 923–925 (1999).

185. Miller, H. C., Allendorf, F. & Daugherty, C. H. Genetic diversity and differentiation at MHC genes in island populations of tuatara (*Sphenodon* spp.). *Mol. Ecol.* **19**, 3894–3908 (2010).

186. Meyer-Rochow, V. & Teh, K. L. Visual predation by tuatara (*Sphenodon punctatus*) on the beach beetle (*Chaerodes trachyscelides*) as a selective force in the production of distinct colour morphs. *Tuatara* **31**, (1991).

187. Meyer-Rochow, V. Behaviour of young tuatara (*Sphenodon punctatus*) in total darkness. *Tuatara* **30**, (1988).

188. Walls, G. L. *The vertebrate eye and its adaptive radiation*. (Cranbrook Institute of Science, 1942).

189. Underwood, G. The Eye. in *Biology of the Reptilia* (ed. Gans, C.) 1–97 (Academic Press, 1970).

190. Röll, B. Gecko vision—visual cells, evolution, and ecological constraints. *J. Neurocytol.* **29**, 471–484 (2000).

191. Schott, R. K. *et al.* Evolutionary transformation of rod photoreceptors in the all-cone retina of a diurnal garter snake. *Proc. Natl. Acad. Sci. U. S. A.* **113**, 356–361 (2016).

192. Meyer-Rochow, V. B., Wohlfahrt, S. & Ahnelt, P. K. Photoreceptor cell types in the retina of the tuatara (*Sphenodon punctatus*) have cone characteristics. *Micron* **36**, 423–428 (2005).

193. Schott, R. K. *et al.* Targeted capture of complete coding regions across divergent species. *Genome Biol. Evol.* **9**, 398–414 (2017).

194. Perry, B. W. *et al.* Molecular adaptations for sensing and securing prey and insight into amniote genome diversity from the garter snake genome. *Genome Biol. Evol.* **10**, 2110–2129 (2018).

195. Martin, H. C. *et al.* Insights into platypus population structure and history from whole-genome sequencing. *Mol. Biol. Evol.* **35**, 1238–1252 (2018).

196. Schott, R. K., Van Nynatten, A., Card, D. C., Castoe, T. A. & S. W. Chang, B. Shifts in selective pressures on snake phototransduction genes associated with photoreceptor transmutation and dim-light ancestry. *Mol. Biol. Evol.* **35**, 1376–1389 (2018).

197. Schott, R. K., Bhattacharyya, N. & Chang, B. S. Evolutionary signatures of photoreceptor transmutation in geckos reveal potential adaptation and convergence with snakes. *Evolution* (2019) doi:10.1111/evo.13810.

198. Yang, Z. PAML 4: Phylogenetic analysis by maximum likelihood. *Mol. Biol. Evol.* **24**, 1586–1591 (2007).

199. Bielawski, J. P. & Yang, Z. A maximum likelihood method for detecting functional divergence at individual codon sites, with application to gene family evolution. *J. Mol. Evol.* **59**, 121–132 (2004).

200. Loew, E. R., Fleishman, L. J., Foster, R. G. & Provencio, I. Visual pigments and oil droplets in diurnal lizards: A comparative study of Caribbean anoles. *J. Exp. Biol.* **205**, 927–938 (2002).

201. Yokoyama, S. Evolution of dim-light and color vision pigments. *Annu. Rev. Genomics Hum. Genet.* **9**, 259–282 (2008).

202. Hauser, F. E. *et al.* Accelerated evolution and functional divergence of the dim light visual pigment accompanies cichlid colonization of Central America. *Mol. Biol. Evol.* **34**, 2650–

2664 (2017).

203. Dungan, S. Z., Kosyakov, A. & Chang, B. S. Spectral tuning of killer whale (*Orcinus orca*) rhodopsin: Evidence for positive selection and functional adaptation in a cetacean visual pigment. *Mol. Biol. Evol.* **33**, 323–336 (2016).

204. Sugawara, T., Imai, H., Nikaido, M., Imamoto, Y. & Okada, N. Vertebrate rhodopsin adaptation to dim light via rapid meta-II intermediate formation. *Mol. Biol. Evol.* **27**, 506–519 (2010).

205. Roth, L. S. & Kelber, A. Nocturnal colour vision in geckos. *Proc. Biol. Sci.* **271 Suppl 6**, S485–7 (2004).

206. Cree, A. *Tuatara: Biology and Conservation of a Venerable Survivor*. (Canterbury University Press, 2014).

207. Steiger, S. S., Fidler, A. E. & Kempenaers, B. Evidence for increased olfactory receptor gene repertoire size in two nocturnal bird species with well-developed olfactory ability. *BMC Evol. Biol.* **9**, 117 (2009).

208. Kumar, S., Stecher, G. & Tamura, K. MEGA7: Molecular Evolutionary Genetics Analysis version 7.0 for bigger datasets. *Mol. Biol. Evol.* **33**, 1870–1874 (2016).

209. Buck, L. & Axel, R. A novel multigene family may encode odorant receptors: A molecular basis for odor recognition. *Cell* **65**, 175–187 (1991).

210. Kaupp, U. B. Olfactory signalling in vertebrates and insects: Differences and commonalities. *Nat. Rev. Neurosci.* **11**, 188–200 (2010).

211. Niimura, Y. & Nei, M. Evolutionary dynamics of olfactory receptor genes in fishes and tetrapods. *Proc. Natl. Acad. Sci. U. S. A.* **102**, 6039–6044 (2005).

212. Hayden, S. & Teeling, E. C. The molecular biology of vertebrate olfaction. *Anat. Rec.* **297**,

2216–2226 (2014).

213. Glusman, G. *et al.* The olfactory receptor gene superfamily: Data mining, classification, and nomenclature. *Mamm. Genome* **11**, 1016–1023 (2000).

214. Qian, W. & Zhang, J. Genomic evidence for adaptation by gene duplication. *Genome Res.* **24**, 1356–1362 (2014).

215. Flouris, A. D. Functional architecture of behavioural thermoregulation. *Eur. J. Appl. Physiol.* **111**, 1–8 (2011).

216. Besson, A. A. & Cree, A. A cold-adapted reptile becomes a more effective thermoregulator in a thermally challenging environment. *Oecologia* **163**, 571–581 (2010).

217. Corkery, I., Bell, B. D. & Nelson, N. J. Behavioral thermoregulation of the tuatara, *Sphenodon punctatus*, under hydric and digestive constraints. *Herpetol. Conserv. Biol.* **9**, 29–37 (2014).

218. Saint Girons, H. Thermoregulation in reptiles with special reference to the tuatara and its ecophysiology. *Tuatara* **24**, 59–80 (1980).

219. Brown, J. H., Gillooly, J. F., Allen, A. P. & Savage, V. M. Toward a metabolic theory of ecology. *Ecology* (2004).

220. Seebacher, F. & Franklin, C. E. Physiological mechanisms of thermoregulation in reptiles: A review. *J. Comp. Physiol. B* **175**, 533–541 (2005).

221. Nilius, B. & Owsianik, G. The transient receptor potential family of ion channels. *Genome Biol.* **12**, 218 (2011).

222. Yu, F. H. & Catterall, W. A. The VGL-chanome: A protein superfamily specialized for electrical signaling and ionic homeostasis. *Sci. Signal.* **2004**, re15 (2004).

223. Clapham, D. E., Julius, D., Montell, C. & Schultz, G. International Union of Pharmacology.

XLIX. Nomenclature and structure-function relationships of transient receptor potential channels. *Pharmacol. Rev.* **57**, 427–450 (2005).

224. Voets, T. *et al.* The principle of temperature-dependent gating in cold- and heat-sensitive TRP channels. *Nature* **430**, 748–754 (2004).

225. Wang, H. & Siemens, J. TRP ion channels in thermosensation, thermoregulation and metabolism. *Temperature (Austin)* **2**, 178–187 (2015).

226. Tan, C.-H. & McNaughton, P. A. The TRPM2 ion channel is required for sensitivity to warmth. *Nature* **536**, 460–463 (2016).

227. Seebacher, F. & Murray, S. A. Transient receptor potential ion channels control thermoregulatory behaviour in reptiles. *PLOS One* **2**, e281 (2007).

228. Yang, Z. & dos Reis, M. Statistical properties of the branch-site test of positive selection. *Mol. Biol. Evol.* **28**, 1217–1228 (2011).

229. Nielsen, R. & Yang, Z. Likelihood models for detecting positively selected amino acid sites and applications to the HIV-1 envelope gene. *Genetics* **148**, 929–936 (1998).

230. Yang, Z., Nielsen, R., Goldman, N. & Pedersen, A. M. Codon-substitution models for heterogeneous selection pressure at amino acid sites. *Genetics* **155**, 431–449 (2000).

231. Yang, Z., Wong, W. S. W. & Nielsen, R. Bayes empirical bayes inference of amino acid sites under positive selection. *Mol. Biol. Evol.* **22**, 1107–1118 (2005).

232. Zhang, J., Nielsen, R. & Yang, Z. Evaluation of an improved branch-site likelihood method for detecting positive selection at the molecular level. *Mol. Biol. Evol.* **22**, 2472–2479 (2005).

233. Caterina, M. J., Rosen, T. A., Tominaga, M., Brake, A. J. & Julius, D. A capsaicin-receptor homologue with a high threshold for noxious heat. *Nature* **398**, 436–441 (1999).

234. Hughes, J. *et al.* The polycystic kidney disease 1 (PKD1) gene encodes a novel protein with multiple cell recognition domains. *Nat. Genet.* **10**, 151–160 (1995).

235. Ishimaru, Y. *et al.* Transient receptor potential family members PKD1L3 and PKD2L1 form a candidate sour taste receptor. *Proc. Natl. Acad. Sci. U. S. A.* **103**, 12569–12574 (2006).

236. Sidi, S., Friedrich, R. W. & Nicolson, T. NompC TRP channel required for vertebrate sensory hair cell mechanotransduction. *Science* **301**, 96–99 (2003).

237. Shin, J.-B. *et al.* Xenopus TRPN1 (NOMPC) localizes to microtubule-based cilia in epithelial cells, including inner-ear hair cells. *Proc. Natl. Acad. Sci. U. S. A.* **102**, 12572–12577 (2005).

238. Labunskyy, V. M., Hatfield, D. L. & Gladyshev, V. N. Selenoproteins: Molecular pathways and physiological roles. *Physiol. Rev.* **94**, 739–777 (2014).

239. Mariotti, M. & Guigó, R. Selenoprofiles: Profile-based scanning of eukaryotic genome sequences for selenoprotein genes. *Bioinformatics* **26**, 2656–2663 (2010).

240. Mariotti, M. *et al.* Composition and evolution of the vertebrate and mammalian selenoproteomes. *PLOS One* **7**, e33066 (2012).

241. Santesmasses, D., Mariotti, M. & Guigó, R. Computational identification of the selenocysteine tRNA (tRNASec) in genomes. *PLOS Comput. Biol.* **13**, e1005383 (2017).

242. Park, J. M. *et al.* Upstream promoter elements are sufficient for selenocysteine $tRNA^{[Ser]Sec}$ gene transcription and to determine the transcription start point. *Gene* **162**, 13–19 (1995).

243. Carlson, B. A. *et al.* Selenocysteine $tRNA^{[Ser]Sec}$: From nonsense suppressor tRNA to the quintessential constituent in selenoprotein biosynthesis. in *Selenium* (eds. D., H., U., S., P., T. & V., G.) 3–12 (Springer International Publishing, 2016).

244. O'Neill, V. A., Eden, F. C., Pratt, K. & Hatfield, D. L. A human opal suppressor tRNA gene

and pseudogene. *J. Biol. Chem.* **260**, 2501–2508 (1985).

245. Bromham, L. Molecular clocks in reptiles: Life history influences rate of molecular evolution. *Mol. Biol. Evol.* **19**, 302–309 (2002).

246. Hay, J. M., Subramanian, S., Millar, C. D., Mohandesan, E. & Lambert, D. M. Rapid molecular evolution in a living fossil. *Trends Genet.* **24**, 106–109 (2008).

247. Miller, H. C., Moore, J. A., Allendorf, F. W. & Daugherty, C. H. The evolutionary rate of tuatara revisited. *Trends in genetics: TIG* vol. 25 13–15 (2009).

248. Subramanian, S., Hay, J. M., Mohandesan, E., Millar, C. D. & Lambert, D. M. Molecular and morphological evolution in tuatara are decoupled. *Trends Genet.* **25**, 16–18 (2009).

249. Harris, R. S. Improved pairwise alignment of genomic DNA. (Pennsylvania State UniversityUniversity Park, PA, United States, 2007).

250. Kent, W. J., Baertsch, R., Hinrichs, A., Miller, W. & Haussler, D. Evolution's cauldron: Duplication, deletion, and rearrangement in the mouse and human genomes. *Proc. Natl. Acad. Sci. U.S.A.* **100**, 11484–11489 (2003).

251. Blanchette, M. *et al.* Aligning multiple genomic sequences with the threaded blockset aligner. *Genome Res.* **14**, 708–715 (2004).

252. Meredith, R. W. *et al.* Impacts of the Cretaceous terrestrial revolution and KPg extinction on mammal diversification. *Science* **334**, 521–524 (2011).

253. Jarvis, E. D. *et al.* Whole-genome analyses resolve early branches in the tree of life of modern birds. *Science* **346**, 1320–1331 (2014).

254. Pyron, R. A., Burbrink, F. T. & Wiens, J. J. A phylogeny and revised classification of Squamata, including 4161 species of lizards and snakes. *BMC Evol. Biol.* **13**, 93 (2013).

255. Hubisz, M. J., Pollard, K. S. & Siepel, A. PHAST and RPHAST: Phylogenetic analysis with

space/time models. *Brief. Bioinform.* **12**, 41–51 (2011).

256. Sanderson, M. J. Estimating absolute rates of molecular evolution and divergence times: A penalized likelihood approach. *Mol. Biol. Evol.* **19**, 101–109 (2002).

257. Sanderson, M. J. r8s: Inferring absolute rates of molecular evolution and divergence times in the absence of a molecular clock. *Bioinformatics* **19**, 301–302 (2003).

258. Benton, M. J. *et al.* Constraints on the timescale of animal evolutionary history. *Palaeontol. Electronica* (2015) doi:10.26879/424.

259. Head, J. J. Fossil calibration dates for molecular phylogenetic analysis of snakes 1: Serpentes, Alethinophidia, Boidae, Pythonidae. *Palaeontol. Electronica* (2015) doi:10.26879/487.

260. Hedges, S. B., Marin, J., Suleski, M., Paymer, M. & Kumar, S. Tree of life reveals clock-like speciation and diversification. *Mol. Biol. Evol.* **32**, 835–845 (2015).

261. Löytynoja, A. & Goldman, N. An algorithm for progressive multiple alignment of sequences with insertions. *Proc. Natl. Acad. Sci. U. S. A.* **102**, 10557–10562 (2005).

262. Kück, P. & Longo, G. C. FASconCAT-G: Extensive functions for multiple sequence alignment preparations concerning phylogenetic studies. *Front. Zool.* **11**, 81 (2014).

263. Mirarab, S. *et al.* ASTRAL: Genome-scale coalescent-based species tree estimation. *Bioinformatics* **30**, i541–8 (2014).

264. Sayyari, E. & Mirarab, S. Fast coalescent-based computation of local branch support from quartet frequencies. *Mol. Biol. Evol.* **33**, 1654–1668 (2016).

265. Borowiec, M. L. AMAS: a fast tool for alignment manipulation and computing of summary statistics. *PeerJ* **4**, e1660 (2016).

266. Nguyen, L.-T., Schmidt, H. A., von Haeseler, A. & Minh, B. Q. IQ-TREE: a fast and

effective stochastic algorithm for estimating maximum-likelihood phylogenies. *Mol. Biol. Evol.* **32**, 268–274 (2015).

267. Kalyaanamoorthy, S., Minh, B. Q., Wong, T. K. F., von Haeseler, A. & Jermiin, L. S. ModelFinder: fast model selection for accurate phylogenetic estimates. *Nat. Methods* **14**, 587–589 (2017).

268. Hoang, D. T., Chernomor, O., von Haeseler, A., Minh, B. Q. & Vinh, L. S. UFBoot2: Improving the Ultrafast Bootstrap Approximation. *Mol. Biol. Evol.* **35**, 518–522 (2018).

269. Guindon, S. *et al.* New algorithms and methods to estimate maximum-likelihood phylogenies: assessing the performance of PhyML 3.0. *Syst. Biol.* **59**, 307–321 (2010).

270. Suchard, M. A. & Rambaut, A. Many-core algorithms for statistical phylogenetics. *Bioinformatics* **25**, 1370–1376 (2009).

271. Gould, S. J. & Eldredge, N. Punctuated equilibria: The tempo and mode of evolution reconsidered. *Paleobiology* **3**, 115–151 (1977).

272. Pagel, M., Venditti, C. & Meade, A. Large punctuational contribution of speciation to evolutionary divergence at the molecular level. *Science* **314**, 119–121 (2006).

273. Organ, C. L., Shedlock, A. M., Meade, A., Pagel, M. & Edwards, S. V. Origin of avian genome size and structure in non-avian dinosaurs. *Nature* **446**, 180–184 (2007).

274. Venditti, C., Meade, A. & Pagel, M. Detecting the node-density artifact in phylogeny reconstruction. *Syst. Biol.* **55**, 637–643 (2006).

275. Webster, A. J., Payne, R. J. H. & Pagel, M. Molecular phylogenies link rates of evolution and speciation. *Science* **301**, 478 (2003).

276. Abascal, F., Zardoya, R. & Telford, M. J. TranslatorX: Multiple alignment of nucleotide sequences guided by amino acid translations. *Nucleic Acids Res.* **38**, W7–13 (2010).

277. Katoh, K., Kuma, K.-I., Toh, H. & Miyata, T. MAFFT version 5: Improvement in accuracy of multiple sequence alignment. *Nucleic Acids Res.* **33**, 511–518 (2005).

278. Suyama, M., Torrents, D. & Bork, P. PAL2NAL: Robust conversion of protein sequence alignments into the corresponding codon alignments. *Nucleic Acids Res.* **34**, W609–12 (2006).

279. Capella-Gutiérrez, S., Silla-Martínez, J. M. & Gabaldón, T. trimAl: A tool for automated alignment trimming in large-scale phylogenetic analyses. *Bioinformatics* **25**, 1972–1973 (2009).

280. Storey, J. D. A direct approach to false discovery rates. *J. R. Stat. Soc. Series B Stat. Methodol.* **64**, 479–498 (2002).

281. Xie, C. *et al.* KOBAS 2.0: A web server for annotation and identification of enriched pathways and diseases. *Nucleic Acids Res.* **39**, W316–22 (2011).

282. Blankenberg, D., Taylor, J., Nekrutenko, A. & Galaxy Team. Making whole genome multiple alignments usable for biologists. *Bioinformatics* **27**, 2426–2428 (2011).

283. Karolchik, D. *et al.* The UCSC Table Browser data retrieval tool. *Nucleic Acids Res.* **32**, D493–6 (2004).

284. Canty, A. & Ripley, B. boot: Bootstrap R (S-Plus) functions. R package version 1.3-11. (2014).

285. Janes, D. E. *et al.* Molecular evolution of Dmrt1 accompanies change of sex-determining mechanisms in reptilia. *Biol. Lett.* **10**, 20140809 (2014).

286. O'Meally, D., Miller, H., Patel, H. R., Graves, J. A. M. & Ezaz, T. The first cytogenetic map of the tuatara, *Sphenodon punctatus*. *Cytogenet. Genome Res.* **127**, 213–223 (2009).

287. Li, H. & Durbin, R. Inference of human population history from individual whole-genome

sequences. *Nature* **475**, 493–496 (2011).

288. Li, H. *et al.* The Sequence Alignment/Map format and SAMtools. *Bioinformatics* **25**, 2078–2079 (2009).

289. Mitchell, N. J., Allendorf, F. W., Keall, S. N., Daugherty, C. H. & Nelson, N. J. Demographic effects of temperature-dependent sex determination: Will tuatara survive global warming? *Glob. Chang. Biol.* **16**, 60–72 (2010).

290. Cooper, A. & Cooper, R. A. The Oligocene bottleneck and New Zealand biota: Genetic record of a past environmental crisis. *Proc. Biol. Sci.* **261**, 293–302 (1995).

291. Bunce, M. *et al.* The evolutionary history of the extinct ratite moa and New Zealand Neogene paleogeography. *Proc. Natl. Acad. Sci. U. S. A.* **106**, 20646–20651 (2009).

292. Elshire, R. J. *et al.* A robust, simple genotyping-by-sequencing (GBS) approach for high diversity species. *PLOS One* **6**, e19379 (2011).

293. Dodds, K. G. *et al.* Construction of relatedness matrices using genotyping-by-sequencing data. *BMC Genomics* **16**, 1047 (2015).

294. Herten, K., Hestand, M. S., Vermeesch, J. R. & Van Houdt, J. K. J. GBSX: A toolkit for experimental design and demultiplexing genotyping by sequencing experiments. *BMC Bioinformatics* **16**, 73 (2015).

295. Li, H. Aligning sequence reads, clone sequences and assembly contigs with BWA-MEM. *arXiv [q-bio.GN]* (2013).

296. Catchen, J. M., Amores, A., Hohenlohe, P., Cresko, W. & Postlethwait, J. H. Stacks: Building and genotyping loci de novo from short-read sequences. *G3* **1**, 171–182 (2011).

297. Van der Auwera, G. A. *et al.* From FastQ data to high confidence variant calls: The Genome Analysis Toolkit best practices pipeline. *Curr. Protoc. Bioinformatics* **43**, 11.10.1–

33 (2013).

298. Poplin, R. *et al.* Scaling accurate genetic variant discovery to tens of thousands of samples. *bioRxiv* 201178 (2018) doi:10.1101/201178.

299. Cingolani, P. *et al.* A program for annotating and predicting the effects of single nucleotide polymorphisms, SnpEff: SNPs in the genome of *Drosophila melanogaster* strain w1118; iso-2; iso-3. *Fly* **6**, 80–92 (2012).

300. Casbon, J. PyVCF: A variant call format reader for Python. (2017).

301. Danecek, P. *et al.* The variant call format and VCFtools. *Bioinformatics* **27**, 2156–2158 (2011).

302. Weir, B. S. & Cockerham, C. C. Estimating F-statistics for the analysis of population structure. *Evolution* **38**, 1358–1370 (1984).

303. Han, E., Sinsheimer, J. S. & Novembre, J. Characterizing bias in population genetic inferences from low-coverage sequencing data. *Mol. Biol. Evol.* **31**, 723–735 (2014).

304. Korneliussen, T. S., Albrechtsen, A. & Nielsen, R. ANGSD: Analysis of Next Generation Sequencing data. *BMC Bioinformatics* **15**, 356 (2014).

305. Meisner, J. & Albrechtsen, A. Inferring population structure and admixture proportions in low-depth NGS data. *Genetics* **210**, 719–731 (2018).

306. Li, H. Toward better understanding of artifacts in variant calling from high-coverage samples. *Bioinformatics* **30**, 2843–2851 (2014).
