# Supplementary material for: The tuatara genome reveals ancient features of amniote evolution
Source: Nature. 2020 Aug 5;584(7821):403–9. doi: 10.1038/s41586-020-2561-9 (PMC7116210; doi:10.1038/s41586-020-2561-9)
Supplement: Supplementary file 2 — Reporting Summary [file 41586_2020_2561_MOESM2_ESM.pdf]

nature research

Corresponding author(s): Neil J. Gemmell et al.

Last updated by author(s): 4/5/2020

Reporting Summary

Nature Research wishes to improve the reproducibility of the work that we publish. This form provides structure for consistency and transparency in reporting. For further information on Nature Research policies, see Authors & Referees and the Editorial Policy Checklist.

Statistics

For all statistical analyses, confirm that the following items are present in the figure legend, table legend, main text, or Methods section.

n/a	Confirmed	
☐	☒	The exact sample size (*n*) for each experimental group/condition, given as a discrete number and unit of measurement
☐	☒	A statement on whether measurements were taken from distinct samples or whether the same sample was measured repeatedly
☐	☒	The statistical test(s) used AND whether they are one- or two-sided<br>*Only common tests should be described solely by name; describe more complex techniques in the Methods section.*
☐	☒	A description of all covariates tested
☐	☒	A description of any assumptions or corrections, such as tests of normality and adjustment for multiple comparisons
☐	☒	A full description of the statistical parameters including central tendency (e.g. means) or other basic estimates (e.g. regression coefficient) AND variation (e.g. standard deviation) or associated estimates of uncertainty (e.g. confidence intervals)
☐	☒	For null hypothesis testing, the test statistic (e.g. *F*, *t*, *r*) with confidence intervals, effect sizes, degrees of freedom and *P* value noted<br>*Give P values as exact values whenever suitable.*
☐	☒	For Bayesian analysis, information on the choice of priors and Markov chain Monte Carlo settings
☐	☒	For hierarchical and complex designs, identification of the appropriate level for tests and full reporting of outcomes
☒	☐	Estimates of effect sizes (e.g. Cohen's *d*, Pearson's *r*), indicating how they were calculated

Our web collection on statistics for biologists contains articles on many of the points above.

Software and code

Policy information about availability of computer code

Data collection

Standard genome assembly and bioinformatic tools were employed and are detailed in full in the methods and supplementary materials for the paper. Genome assembly was undertaken using AllPaths-LG (version 49856, http://software.broadinstitute.org/allpaths-lg/blog/?page_id=12) and Dovetail Genomics HiRise scaffolding software. Transcriptomes were assembled using Trinity v2.2.0. Bisuplhite sequencing data were trimmed using TrimGalore v0.4.0 and reads mapped using Bismark v0.14.311 to identify metylated sites. Repeat annotation was undertaken using CARP, RepeatModeler and LTRharvest. Gene annotation used RepeatMasker (4.0.3) and MAKER2. Genotype-by-sequencing was undertaken using FastQC v0.10.1 (http://www.bioinformatics.babraham.ac.uk/projects/fastqc/) followed by a QC analysis pipeline: “Deconvolute and quality control” https://github.com/AgResearch/DECONVQC] and subsequent demultiplexing using GBSX, read mapping using BWA MEM, and SNV calling using STACKS and GATK.

Data analysis

Standard bioinformatic tools were employed for our analyses. These and are detailed in the methods and supplementary materials for the paper. Where custom code was utilized this is also specified and either available from GitHub or directly from the authors of the relevant section of our manuscript. All attributions to each component of our work are clearly signalled.

For completeness the full list is provided here also:

Repeat and gene annotation
RepeatMasker (v4.0.5), http://www.repeatmasker.org/
MAKER2 (v2.31.8), http://www.yandell-lab.org/software/maker.html
BLAST (v2.2.30+), https://blast.ncbi.nlm.nih.gov/Blast.cgi
SNAP (v2.4.7), http://snap.cs.berkeley.edu
Augustus (v3.3), http://augustus.gobics.de
BUSCO (v3.0), https://busco.ezlab.org

Orthology calling

Ensembl GeneTree pipeline: https://github.com/Ensembl/ensembl and https://github.com/Ensembl/ensembl-compara branch "release/87"
Ensembl Hive workflow management system https://github.com/Ensembl/ensembl-hive branch "version/2.3"
Plotting script: https://github.com/Ensembl/ensembl-compara/blob/release/89/scripts/homology/plotGocData.r
hcluster 0.5.0 https://sourceforge.net/projects/treesoft
TCoffee 9.03.r1318 http://www.tcoffee.org/
Mafft 7.221 https://mafft.cbrc.jp/alignment/software/mafft-7.221-with-extensions-src.tgz
Treebest https://github.com/Ensembl/treebest
QuickTree 1.1 https://github.com/khowe/quicktree
NCBI Blast 2.2.30+ ftp://ftp.ncbi.nlm.nih.gov/blast/executables/blast+/2.2.30/
HMMER 2.3.2 http://eddylab.org/software/hmmer/2.3.2/hmmer-2.3.2.tar.gz
PantherScore 1.0.3 https://github.com/Ensembl/pantherScore
PAML 4.3 http://abacus.gene.ucl.ac.uk/software/SoftOld/paml4.3.tar.gz
Ktreedist 1.0.0 http://molevol.cmima.csic.es/castresana/Ktreedist/Ktreedist_v1.tar.gz
CAFE 2.2 http://downloads.sourceforge.net/project/cafehahnlab/Previous_versions/cafehahnlab-code_v2.2.tgz

Ensembl annotation
RepeatMasker, http://repeatmasker.systemsbiology.net/
RepeatModeler, http://www.repeatmasker.org/RepeatModeler/
GenBlast, http://genome.sfu.ca/genblast/
BWA, http://bio-bwa.sourceforge.net/
MUSCLE, http://www.drive5.com/muscle
BLAST, https://blast.ncbi.nlm.nih.gov/Blast.cgi
ensembl-analysis, https://github.com/Ensembl/ensembl-analysis.git
ensembl code, https://www.ensembl.org/info/docs/api/api_installation.html
exonerate, https://www.ebi.ac.uk/about/vertebrate-genomics/software/exonerate
rnafold, https://github.com/choener/RNAfold
Inferno, http://eddylab.org/infernal/

Investigation of gene co-linearity
BLAST+ 2.8.1 using Geneious version 10.2.6 (https://www.geneious.com).
Circos software http://circos.ca/software/

Ab initio repeat annotation
CARP, https://github.com/carp-te/carp-documentation RepeatModeler, http://www.repeatmasker.org/RepeatModeler/
CENSOR, which requires wu-blast and bioperl, https://girinst.org/downloads/software/censor/ RepeatMasker, http://www.repeatmasker.org/
BLASTN, https://blast.ncbi.nlm.nih.gov/Blast.cgi RepBase, https://www.girinst.org/server/RepBase/
MUSCLE, https://www.drive5.com/muscle/
MrBayes, https://nbisweden.github.io/MrBayes/download.html
FastTree, http://www.microbesonline.org/fasttree/
USEARCH, https://www.drive5.com/usearch/
HMMER, http://hmmer.org/ PILER, https://www.drive5.com/piler/

Repeat annotation of SINEs and DNA transposons
RepeatModeler version 1.0.8 http://www.repeatmasker.org/RepeatModeler/
renameRMDLconsensi.pl script https://github.com/genomicrocosm/physaliaTEcourse/blob/master/Practical2_Computational_annotation/renameRMDLconsensi.pl
repeatModelerPipeline4.pl script https://github.com/genomicrocosm/physaliaTEcourse/blob/master/Practical3_Manual_curation/repeatModelerPipeline4.pl
BLASTn version 2.2.28+ https://blast.ncbi.nlm.nih.gov/Blast.cgi?PAGE_TYPE=BlastDocs&DOC_TYPE=Download
MAFFT version 6 standalone and version 7 webserver https://mafft.cbrc.jp/alignment/software/
BioEdit version 7.2.6.1 https://bioedit.software.informer.com/download/
CENSOR webserver http://www.girinst.org/censor/index.php
RAxML version 8.0.0 in CIPRES Science Gateway https://www.phylo.org/portal2/login!input.action
MEGA version 5.2 https://www.megasoftware.net/
RepeatMasker version 4.0.7 http://www.repeatmasker.org/RMDownload.html
calcDivergenceFromAlign.pl script in RepeatMasker package http://www.repeatmasker.org/RMDownload.html
R scripts for making landscape plots https://github.com/ValentinaBoP/TuataraTELandscapes/blob/master/Tuatara_DNA_SINE_landscape_figures.Rmd

LTR analyses
Genometools, http://genometools.org/pub/genometools-1.5.8.tar.gz, used for indexing genome and running LTRharvest.
Blastn, https://ftp.ncbi.nlm.nih.gov/blast/executables/blast+/2.5.0/ncbi-blast-2.5.0+-x64-linux.tar.gz, used for blasting retroviral proteins.
MAFFT 7, https://mafft.cbrc.jp/alignment/software/, used for multiple alignment of peptide sequences.
CD-HIT-V4.6.5, https://github.com/weizhongli/cdhit, used to reduce number of similar retroelement copies in file.
RAxML 8.2, https://github.com/stamatak/standard-RAxML, used to infer phylogenetic trees under maximum likelihood.
NINJA 1.2.2, http://nimbletwist.com/software/ninja/old_distros/ninja_1.2.2.tgz, used for large-scale neighbor-joining phylogeny inference.
Inkscape 0.92, https://inkscape.org/release/inkscape-0.92/, used for preparing figures of repetitive elements.

RNA annotation
Rfam (version 13.0) covariance models, ftp://ftp.ebi.ac.uk/pub/databases/Rfam/13.0/
tRNAscan-SE (version 1.3.1), http://lowelab.ucsc.edu/software/
Infernal (version 1.1), http://eddylab.org/infernal/

Mitochondrial genome sequence and assembly
Illumina data: Bowtie 2, MaSuRCA, Minimus, Jellyfish, Geneious v10.2.4 (https://www.geneious.com).
Oxford Nanopore data: Nanopolish, Guppy, Blastn, Megablast, Discontiguous Megablast.

MHC
BLAST+ 2.3.0 https://blast.ncbi.nlm.nih.gov/Blast.cgi Geneious 9.1, (https://www.geneious.com).

Opsin gene analysis
BLAST+ 2.2.30, PAML 4.8, MEGA 5.2, PhyML 3.0, BLASTPHYME https://github.com/ryankschott/BlastPhyMe

Odorant receptors
tBLASTn in Geneious 10.0.3 (https://www.geneious.com), MAFFT (v7.338), MEGA 7.0.21, FigTree v1.4.4

Transient receptor potential (TRP) ion channel gene analysis
tBLASTn, https://blast.ncbi.nlm.nih.gov/Blast.cgi MAFFT v7.450 / https://mafft.cbrc.jp/alignment/software/
FastTree2 as implemented in the CIPRES portal https://www.phylo.org/

Selenoprotein analysis
Selenoprofiles v. 3.6, https://github.com/marco-mariotti/selenoprofiles
Secmarker v. 0.4, https://secmarker.crg.cat,

Phylogeny and evolutionary rates
LASTZ-chaining-netting/source code from the avian phylogenomics project (Zhang et al. 2014; GigaScience) https://github.com/gigascience/paper-zhang2014/tree/master/Whole_genome_alignment/
msa_view in PHAST v1.3 /http://compgen.cshl.edu/phast/oldversions.php/
RAxML v8.2.3/https://github.com/stamatak/standard-RAxML
phyloFit in PHAST v1.3 /http://compgen.cshl.edu/phast/oldversions.php/
r8s v1.8 / https://sourceforge.net/projects/r8s/
PRANK v1.7 / http://wasabiapp.org/download/prank/
FASconCAT-G v.1.02 / https://github.com/PatrickKueck/FASconCAT-G
RAxML v8.2.3/https://github.com/stamatak/standard-RAxML
ASTRAL v4 / https://github.com/smirarab/ASTRAL
MAFFT v7.450 / https://mafft.cbrc.jp/alignment/software/
AMAS / https://github.com/marekborowiec/AMAS
IQ-TREE v1.6.12 / http://www.iqtree.org/

Punctuated evolution
Time-calibrated phylogeny: BEAST v2.4.8 on the CIPRES Science Gateway: https://www.phylo.org/
Punctuated evolution: BayesTraits V3.0.2 Nov 2019: http://www.evolution.rdg.ac.uk/BayesTraitsV3.0.2/BayesTraitsV3.0.2.html
Node-density artefact: Test for Punctuational Evolution and the Node-Density Artifact. v1: http://www.evolution.reading.ac.uk/pe/index.html

Patterns of selection
Translatorx, http://translatorx.co.uk/, Translation of nucleotide sequences
MAFFT 7.310, https://mafft.cbrc.jp/alignment/software/ , amino acid alignment
PAL2NALv14, https://github.com/drostlab/orthologr/tree/master/inst/pal2nal/pal2nal.v14 , conversion of protein alignments into codon-based DNA alignments
TrimAI 1.2, http://trimal.cgenomics.org/, Alignment correction
Garli 2.0.1, https://www.nescent.org/wg_garli/Main_Page, maximum likelihood phylogenetic reconstruction
PAML 4.5, http://abacus.gene.ucl.ac.uk/software/paml.html , infer branch specific evolutionary patterns
QVALUE, https://www.bioconductor.org/packages/release/bioc/html/qvalue.html , false discovery rate correction
Kobas 2.0, http://kobas.cbi.pku.edu.cn/kobas3/?t=1, Gene set enrichment analysis
Galaxy's Stitch Gene Blocks Tool, http://www.bioinformatics.nl/galaxy
AlignmentProcessor0.12, https://github.com/WilsonSayresLab/AlignmentProcessor
PAML, CodeML, http://abacus.gene.ucl.ac.uk/software/paml.html
R v3.3.1, https://cran.r-project.org/

Reconstruction of the demographic history of the tuatara
BWA mem, http://bio-bwa.sourceforge.net/
Samtools mpileup, http://www.htslib.org/
PSMC, https://github.com/lh3/psmc

Population genomics
FastQC v0.10.1, http://www.bioinformatics.babraham.ac.uk/projects/fastqc/ QC analysis pipeline, https://github.com/AgResearch/DECONVQC
GBSX, https://github.com/GenomicsCoreLeuven/GBSX
BWA mem, http://bio-bwa.sourceforge.net/
STACKS 1.4.4, http://catchenlab.life.illinois.edu/stacks/
GATK haplotypecaller, https://gatk.broadinstitute.org/hc/en-us/articles/360037225632-HaplotypeCaller

For manuscripts utilizing custom algorithms or software that are central to the research but not yet described in published literature, software must be made available to editors/reviewers. We strongly encourage code deposition in a community repository (e.g. GitHub). See the Nature Research guidelines for submitting code & software for further information.

Data

Policy information about availability of data

All manuscripts must include a data availability statement. This statement should provide the following information, where applicable:

- Accession codes, unique identifiers, or web links for publicly available datasets
- A list of figures that have associated raw data
- A description of any restrictions on data availability

All data are freely available. The Tuatara Genome Consortium Project Whole Genome Shotgun and genome assembly are registered under the umbrella BioProject PRJNA418887 and BioSample SAMN08038466. Transcriptome read data are submitted under SRR7084910 (whole blood) together with prior data SRR485948. The transcriptome assembly is submitted to GenBank with ID GGNQ00000000.1. Illumina short-read and nanopore long read sequence are in SRAs associated with PRJNA445603. The assembly (GCA_003113815.1) described in this paper is version QEPC00000000.1 and consists of sequences QEPC01000001-QEPC01016536. Maker gene predictions are available from Zenodo, DOI: 10.5281/zenodo.1489353. The repeat library database developed for tuatara is available from Zenodo, DOI: 10.5281/zenodo.2585367. Other data for analyses in specfic sections of our paper have been uploaded to Zenodo and DOIs are clearly indicated in the paper.

Field-specific reporting

Please select the one below that is the best fit for your research. If you are not sure, read the appropriate sections before making your selection.

☐ Life sciences ☐ Behavioural & social sciences ☒ Ecological, evolutionary & environmental sciences

For a reference copy of the document with all sections, see nature.com/documents/nr-reporting-summary-flat.pdf

Ecological, evolutionary & environmental sciences study design

All studies must disclose on these points even when the disclosure is negative.

Study description	This is the study of the genome, transcriptome and methylome of one exemplar male tuatara from Lady Alice Island in the far North of New Zealand. We have subsequently compared this genome to other published genomes, while also undertaking population geneomic investigations of tuatara using 30 individuals that span the main axes of genetic diversity for tuatara .
Research sample	1 exemplar tuatara (Sphenodon punctatus) from Lady Alice Is. New Zealand. 30 other tuatara samples (approximately half male, half female) spanning three main populations (Lady Alice, Stephens, and Brothers Is.) that encompass the main axes of genetic diversity for this species.
Sampling strategy	Sampling was undertaken using venipuncture. Because this species is special to Maori and highly protected, our sampling strategy was ad hoc relying heavily on samples collected previously for other studies. While no tests of statistical power were undertaken past investigations of population structure in this species suggested that 10 samples per population was likely adequate to capture much of the variation present. The use of equal numbers of males and females provided a reasonable opportunity to explore obvious sex differences, should these be present.
Data collection	Blood samples for the population-level analysis were collected during field work for research on other projects, for example, investigating population size and genetic diversity of island populations of tuatara. Islands were searched for tuatara at night when they were active above ground. Tuatara were captured by hand, and ~1ml blood samples were obtained from the caudal artery. Samples were stored in liquid nitrogen then once back at the lab, transferred to a -80 ˚C freezer. The exemplar tuatara sample was collected during a survey trip to Lady Alice Island, but otherwise procedures were similar to earlier population samples.
Timing and spatial scale	Samples were collected at each of three sites (Lady Alice, Stephens, and Brothers Is.) from 1984 to 2011.
Data exclusions	Several samples (2 out of 30) failed in downstream genotype by sequencing and bisulphite methylation profiling due to DNA quality/ qunatity issues.
Reproducibility	Data reproducibility was verified using repeat sequencing and independent analyses using alternative pipelines. e.g. genome assembly used at least three independent pipelines, each of which had high concordance.
Randomization	There was no need to randomise our study given the focus on genomic and population genomics
Blinding	Blinding was irrelevant given the focus on genomic and population genomics

Did the study involve field work? ☒ Yes ☐ No

Field work, collection and transport

Field conditions	Data not available, but field sites are all temperate offshore Islands in New Zealand
Location	Lady Alice, Stephens, and Brothers Is. New Zealand
Access and import/export	Samples were collected with the permission and support of local iwi and the NZ DOC, under Victoria University of Wellington

Access and import/export	Animal Ethics approvals 2006R12; 2009R12; 2012R33; 22347 and held and used under permits 45462-DOA (1/9/15) and 32037-RES (11/11/11) issued by the New Zealand Department of Conservation. Samples from the exemplar Lady Alice animals were shipped from NZ to ZSD in USA using CITES Permit to Export – Permit # 13NZ000096 (25/7/13), NZ Dept. of Conservation Authority to Export – Permit # 36830-RES (11/7/2013), and CITES Import Permit # 13US727416/9 and US Federal Fish and Wildlife Permit # LE736007-0 (15/7/13). The ethics application and other permitting processes ensures minimal numbers of individuals are used in research, and that their use is justified under New Zealand and international law. All approvals issued by the Department of Conservation ensure the research complies with relevant acts of Parliament for access to collection sites and handing and research on native species of New Zealand. All Department of Conservation permits for capture, sampling, require consultation with local indigenous people affiliated with the islands.
Disturbance	Animals were handled minimally and returned to the site of capture for release.

Reporting for specific materials, systems and methods

We require information from authors about some types of materials, experimental systems and methods used in many studies. Here, indicate whether each material, system or method listed is relevant to your study. If you are not sure if a list item applies to your research, read the appropriate section before selecting a response.

Materials & experimental systems

n/a	Involved in the study	
☒	☐	Antibodies
☒	☐	Eukaryotic cell lines
☒	☐	Palaeontology
☐	☒	Animals and other organisms
☒	☐	Human research participants
☒	☐	Clinical data

Methods

n/a	Involved in the study	
☒	☐	ChIP-seq
☒	☐	Flow cytometry
☒	☐	MRI-based neuroimaging

Animals and other organisms

Policy information about studies involving animals; ARRIVE guidelines recommended for reporting animal research

Laboratory animals	This study did not involve the us of laboratory animals
Wild animals	Adult tuatara, (Sphenodon punctatus) from Lady Alice, Stephens, and Brothers Is. New Zealand were captured and blood samples taken using established venipuncture approaches. Animals were captured while active outside their burrows at night. Blood samples were taken upon capture and animals were released at their site of capture. If animals were held while others were being sampled (<1h), they were placed into a cloth bag. No animals died as a result of this study.
Field-collected samples	Bloods from one exemplar male and a further 30 animals, previously collected for other purposes, were utlised for our work. Sex ratios among samples were approximately 50:50 and equal numbers of samples were obtained from all sites. One sample was collected specifically for this study - the exemplar - but during another research project, so it did not require separate arrangements solely for this sample. All others arose from frozen samples from previous studies.
Ethics oversight	Samples were collected under Victoria University of Wellington Animal Ethics approvals 2006R12; 2009R12; 2012R33; 22347.

Note that full information on the approval of the study protocol must also be provided in the manuscript.
